# Draft genome of *Semisulcospira libertina*, a species of freshwater snail

**DOI:** 10.5808/gi.21039

**Published:** 2021-09-30

**Authors:** Jeong-An Gim, Kyung-Wan Baek, Young-Sool Hah, Ho Jin Choo, Ji-Seok Kim, Jun-Il Yoo

**Affiliations:** 1 Medical Science Research Center, Korea University Guro Hospital, Korea University College of Medicine, Seoul 08308, Korea; 2 Department of Physical Education, Gyeongsang National University, Jinju 52727, Korea; 3 Department of Orthopaedic Surgery, Gyeongsang National University Hospital, Jinju 52727, Korea; 4 Biomedical Research Institute, Gyeongsang National University Hospital, Jinju 52727, Korea; 5 South Korea 4H Association, Seoul 05269, Korea

**Keywords:** *de novo* assembly, draft genome, *Semisulcospisa libertina*

## Abstract

*Semisulcospira libertina*, a species of freshwater snail, is widespread in East Asia. It is important as a food source. Additionally, it is a vector of clonorchiasis, paragonimiasis, metagonimiasis, and other parasites. Although *S. libertina* has ecological, commercial, and clinical importance, its whole-genome has not been reported yet. Here, we revealed the genome of *S. libertina* through *de novo* assembly. We assembled the whole-genome of *S. libertina* and determined its transcriptome for the first time using Illumina NovaSeq 6000 platform. According to the *k*-mer analysis, the genome size of *S. libertina* was estimated to be 3.04 Gb. Using RepeatMasker, a total of 53.68% of repeats were identified in the genome assembly. Genome data of *S. libertina* reported in this study will be useful for identification and conservation of *S. libertina* in East Asia.

## Introduction

As a species of freshwater snail, *Semisulcospira libertina* is widespread in East Asia and it is an important food source. It is also a vector of clonorchiasis, paragonimiasis, metagonimiasis, and other parasites. It inhabits clean running waters or pools such as drainage ditches, slow flowing rivers, rice paddies, and streams. The phylogeography of *S. libertina* in Taiwan has been revealed in two studies [1,2] by its mitochondrial cytochrome c oxidase subunit I (COI) sequences. *S. libertina* belongs to genus *Semisulcospira*, a well-known group of freshwater snails. *S. libertina* can be readily identified by its nuclear seqeunce (28S ribosomal RNA) and mitochondrial sequence (16S ribosomal RNA) [3]. In genus *Semisulcospira*, mitochondrial genomes of *S. libertina* [4], *S. coreana* [5], and *S. gottsei* [6] have been reported. In Gastropoda, mitochondrial genome studies have been performed to classify species until now, as well as genomes were revealed in some species. The genome of *Biomphalaria glabrata*, a freshwater snail, has been reported [7]. Genomes of owl limpet (*Lottia gigantea*) [8] and abalones (*Haliotis discus hannai*) [9] have also been revealed. However, no study has reported whole-genome of *Semisulcospira* genus. A draft genome of *Radix auricularia* (big-ear Radix) [10] and a genome of *Conus tribblei* [11] are cases of genome sequencing in Gastropoda.

*S. libertina* has ecological, commercial, and clinical importance [12,13], thus whole-genome data of *S. libertina* could be of great help in many ways. In this study, we sequenced the whole-genome and transcriptome of *S. libertina* for the first time using Illumina NovaSeq 6000 platform. To enhance the accuracy of gene prediction, we integrated *S. libertina* transcriptome data with gene set annotation for the assembled genome. Our genomic data could provide basic knowledge for understanding genomic features of *S. libertina*. They could be used for further comparative, systemic, and functional genomic studies of freshwater snails.

## Methods

### Sample collection and nucleic acid extraction

Specimens of healthy *S. libertina* were collected from the upstream of Bukhan River basin, South Korea (37°47'32.3"N, 127°31'49.8"E) in June 2019. Morphometric characteristics such as shell length (20‒30 mm) and weight (5‒6 g) of collected *S. libertina* samples were determined. The samples were stored in a ‒80°C freezer. Freshest individuals (five for DNA and five for RNA) with the best DNA or RNA quality were studied. Genomic DNAs were extracted from muscle tissues using DNeasy Blood & Tissue Kits (Qiagen, Hilden, Germany). RNAs were extracted using Trizol reagent (Invitrogen, Carlsbad, CA, USA). The quality of RNA was comfirmed based on 28S/18S ratio. RNA integrity number (RIN) of the extracted RNA was determiend using a Tecan F-200 and an Agilent Bioanalyzer 2100 system (Agilent, Santa Clara, CA, USA). All RNAs extracted from samples had RIN values of 6.5‒7.0. One sample with the highest quality among five DNA or RNA samples was used for sequencing.

### Sequencing library construction

To construct sequencing library, high molecular weight genomic DNAs were sheared to ~500 bp using a Covaris S2 Ultrasonicator system. All DNA libraries for sequencing were constructed following Illumina’s instruction. To check the quality of the library constructed, the size of the library was detemrined with a 2200 TapeStation (Agilent). Normalized libraries were diluted with hybridization buffer. Clusters of each library were then made with a cBot system and a HiSeq Rapid Duo cBot Sample Loading Kit (Illumina, San Diego, CA, USA). Pair-end libraries were prepared following the manufacturer’s guideline (Illumina). Final library products were sequenced on an Illumina NovaSeq 6000 platform using HiSeq Rapid Paired End Cluster Kit v2 and SBS Kit V2 for 100 PE sequencing (Illumina). Raw fastq sequences are available under BioProject ID PRJNA659426.

### Filtering raw sequences for *de novo* assembly

To maintain quality of sequences, raw reads were filtered to remove the following: (1) reads presented with letter N (ambiguous bases) or poly-A motif; (2) reads with low-quality bases (below base quality 7) from the 549 bp insert size library; (3) reads with adapter contamination; (4) reads with small sizes of inserts in which read 1 and read 2 overlapped for more than 10 bp (only 10% mismatch allowed); (5) PCR duplicates (reads were considered duplicates when read 1 and read 2 of two pair-end reads were identical).

### *De novo* assembly of the *S. libertina* genome

*K*-mer size of 17-bp was estimated using SOAPec v2.01, and the best k was 77. The genome size was calculated using the following formula: genome size = total number of *k*-mer/*k*-mer depth. The size of the *S. libertina* genome was estimated to be 3.04 Gb. The genome was then assembled using qualified reads from the pair-end libraries. *De novo* assembly invovled contig construction followed by scaffolding and gap closure. In the step of contig construction, a short insert library (429 bp) was used to construct a de Brujin graph using SOAPdenovo v2.04 with default parameters [14]. All erroneous data derived from clip tips, bubbles, and connection with low coverage were eliminated. All qualified reads were then realigned with contig sequences. Reads were mapped with bowtie2 v2.2.5 using end-to-end mode and default options. Mapping was perforemd with samtools v1.2.1 and bedtools v2.26. We used benchmarking universal single-copy orthologs software (BUSCO; v2.0) to assess the genome completeness [15].

### Identification of repeat sequences

To identify repeat sequences in the genome of *S. libertina*, the follwoing two approaches were applied: (1) a homology-based approach; and (2) a *de novo*-based approach. Identification of homology-based repeat sequences was performed with RepeatMasker (v4.0.9) using Repbase libraries (2019, volume 19, issue 1) containing identified repeat sequences [16].

Identification of *de novo*-based repeat sequences was then finished with RepeatModeler v1.0.8 [16]. Simple sequence repeats (SSRs) were identified using perl script of SSR identification tool (SSRIT; ftp://ftp.gramene.org/pub/gramene/archives/software/scripts/ssr.pl). SSR target primer pairs were designed with flanking sequences of SSR using Primer 3 program (v0.4.0) [17]. These primers met the following criteria: having GC content > 50%, annealing temperature range at 55‒62°C, and primer length of 18‒26 bp in size.

### Prediction of noncoding RNAs

From *de novo* assembled *S. libertina* genome, four types of noncoding RNA (ncRNA; miRNAs, tRNAs, rRNAs, and snRNAs) were identified by searching databases as follows, tRNAscan-SE with default setting was applied to search for definite tRNA positions [18]. To detect snRNAs and miRNAs, INFERNAL v1.1.1 was used to search for putative sequences with Rfam database (release 9.1) [19]. For rRNA predictions in the *S. libertina* genome, BLAST (v2.2.29+) homology search was performed [20].

### Transcriptome sequencing

For RNA sequencing, cDNA libraries were constructed. mRNA was enriched with oligo-dT attached magnetic beads from total RNA (2 mg). Purified mRNAs were sheared into short fragments and synthesized into double-stranded cDNAs by reverse-transcription immediately. Synthesized cDNAs were subjected to end-repair, poly-A addition, and ligations with adaptors provided by a TruSeqRNA sample prep Kit (Illumina). Modified mRNA fragments were separated on bluepippin 2% agarose gel cassette. Suitable fragments were automatically purified and used as templates for PCR amplification. Final products were 400–500 bp in length and evaluated with an Agilent High Sensitivity DNA Kit (Agilent) on an Agilent Bioanalyzer 2100 system. Subsequently, the constructed libraries were sequenced using an Illumina HiSeq 2500 sequencer (Illumina). All processes were conducted by TheragenETEX Bio Institute (Suwon, Korea).

### Gene prediction and annotation

For the annotation of *S. libertina* genome, a combination of evidence-based gene prediction (RNA-sequencing [RNA-seq] and proteins) and *ab initio* gene prediction was used. First, transcript alignment was performed with STAR v2.7.0a using a set of gene model annotations [21]. From RNA-seq data, clean reads with average quality scores of higher than Q30 were aligned from all libraries and used for gene prediction using GeneMark-ET v4.29 [22]. Next, homologous proteins of other species were aligned to the genome using TBlastN v2.2.29+ with an E-value cutoff of 1E–5. Aligned protein sequences were used for the prediction of gene regions using Exonerate v2.2.0 with default parameters [20]. A final gene set of *S. libertina* was produced with AUGUSTUS v3.2.1 using default settings [23]. Gene functions were assigned according to the best alignment attained using BLASTP against UniProt database (Last modified in January 17, 2019), NCBI nr (accecced in June 28, 2019; E-value cutoff of 1E–5), and InterProScan v5.17 [24,25].

### Visualization and phylogenetic analysis

For visualization, we used R v3.6.1 and RStudio v1.2.5019 (https://cran.r-project.org/). For heatmap drawing, we used “pheatmap v1.0.10” and “heatmap3 v1.1.6” packages. From whole-mitochondrial genome and COI regions of mitochondrial DNA, the maximum likelihood tree was obtained with a Tamura-Nei model using MEGA-X v10.1.4 [26,27]. Mitochondrial DNA sequences of related species were retrieved from GenBank. Accession numbers were indicated in dendrograms.

## Results

### *De novo* assambly of *S. libertina*

A genomic DNA sample of *S. libertina* was used to construct short-insert paired end libraries. Paired end sequencing of 429 bp insert libraries generated a total of 60.99 Gb sequence data with an Illumina NovaSeq 6000 platform. Based on *k*-mer analysis, the genome size of *S. libertina* was estimated to be 3.04 Gb (3,037,193,258 bp) at a *k*-mer size of 17. The *k*-mer frequency distribution had two peaks. This is because the heterozygosity of the *S. libertina* genome is relatively high [28]. Sequence reads from paired end and mate were assembled, and gaps in scaffolds were subsequently filled with Illumina reads using GapCloser v1.12 [14]. Characteristics of the assembled genome are listed in Supplementary Table 1. The N50 size was 2,788. The total number of contigs was 748,492. Raw sequence data were deposited to NCBI SRA (PRJNA659426). Benchmarking was performed by universal single-copy orthologs software (BUSCO; v2.0) to assess the genome completeness [15]. Our assembly covered 23.0% of core genes, with 225 genes being complete genes (Supplementary Table 2).

### Gene prediction and annotation

Gene prediction and structural-annotation were carried out using homology-based search. Determination of gene set was performed using transcriptome data. First, we sought to comprehensively describe ncRNA to build better coding gene models. By homology-based Blast search, a total of 935 rRNA copies were matched with 105,942 bp, accounting for 0.01% of the genome. In addition, 572 tRNA copies were estimated using tRNAscan-SEtool [18]. Using INFERNAL [19], miRNAs with 109,716 copies (9,270,754 bp) and snRNAs with 3,797 copies (426,539 bp) were found.

A total of 61,610 gene models were then predicted. The average length of genes was calculated to be 424 bp. Gene annotation databases were used to annotate gene models, find protein sequence, and search for biological functions of annotated genes. Among 61,610 gene models, 39,949 were annotated genes. A total of 10,065, 19,659, and 37,333 genes were producted hits with UniProt, NCBI nonredundant, and InterProScan databases, respectively (Table 1). Each analysis was performed under default options.

### Repeat sequences

Repeat composition of the *S. libertina* genome was then investigated. We used homology and *de novo*-based approaches first. We then combined these two approaches. Using RepeatMasker, a total of 53.68% of repeats were identified in the genome. More than half of total repeat length was filled with unclassified repeats, accounting for 34.68% of the genome. DNA transposons accounted for 7.48% of the genome. Most sequences of retrotransposons consisted of long interspersed nuclear elements (9.54%) and long terminal repeat elements (5.58%), whereas short interspersed nuclear elements (0.97%) were present at low proportions (Table 2).

We also discovered features of SSRs to provide clues for polymorphic information of other species of the genus *Semisulcospira* and molecular markers. In the genome, a total of 35,610 copies of dinucleotide repeats were detected whereas the copy number of each hexa to deca-nucoeotide repeat was <70 (Table 3). On average, a total of 512,774 dinucleotide repeats were detected and 364.97 dinucleotide repeats were detected per million basepairs. Among dinucleotide repeats, CA had the highest frequency (6,494 copies) whereas CG had the lowest frequency (656 copies). Based on these SSR data, we predicted a total of 750,057 primer sets for SSR targets that could be used for polymorphism screening across congener species of *S. libertina*.

### Comparative analysis with related species

**Fig. 1.**
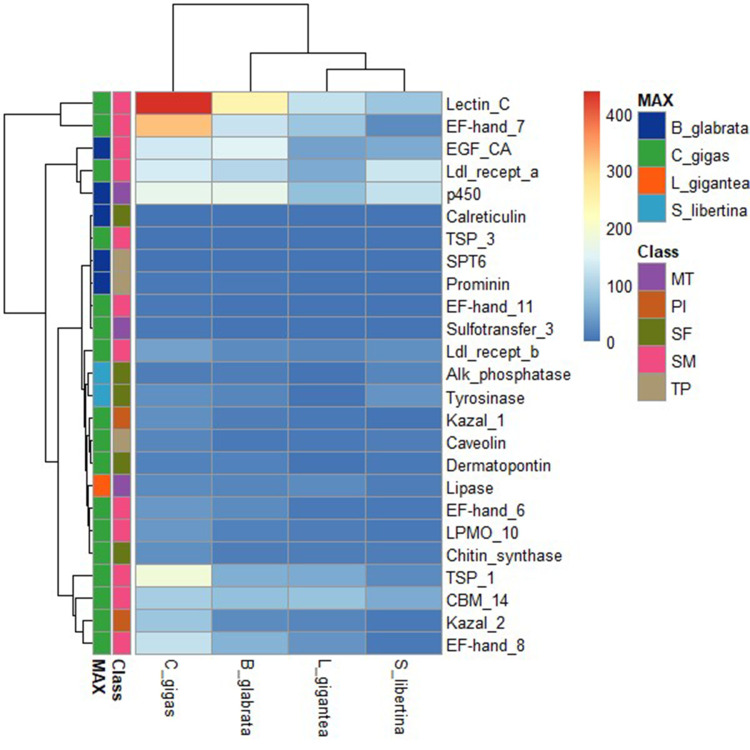
Copy number of orthologous shell formation related genes calculated with PFAM in four genomes (air-breathing freshwater snail, *Biomphalaria glabrata*; oyster, *Crassostrea gigas*; owl limpet, *Lottia gigantea*; freshwater snail, *Semisulcospira libertina*). In these four genomes, genes detected with the highest frequency were indicated with ‘MAX’ row bar. A total of 25 genes were used to depict heatmap and construct phylogenetic tree from five class (MT, metabolic transcripts; PI, protease inhibitors; SF, shell formation; SM, small matrix proteins; TP, transmembrane proteins). They are indicated as the second row bar. Full description of each gene name is shown in Supplementary Table 3.

Four genomes of similar species, owl limpet, air-breathing freshwater snail, and oyster (owl limpet, *Lottia gigantea*; air-breathing freshwater snail, *Biomphalaria glabrata*; oyster, *Crassostrea gigas*) were compared. Based on PFAM database, we compared the copy number of shell formation related genes in each genome [29,30]. In the heatmap, the number of orthologous genes in each genome was depicted for 25 genes (Table 4, Fig. 1). Shell formation-related genes were retrived from previous studies [29,30]. In these four genomes, the mostly detected gene was indicated by a ‘Top’ row bar. A total of 25 genes used to depict heatmap and phylogenetic tree from four class (MT, metabolic transcripts; PI, protease inhibitors; SF, shell formation; SM, small matrix proteins; and TP, transmembrane proteins) were indicated by a second row bar.

**Fig. 2.**
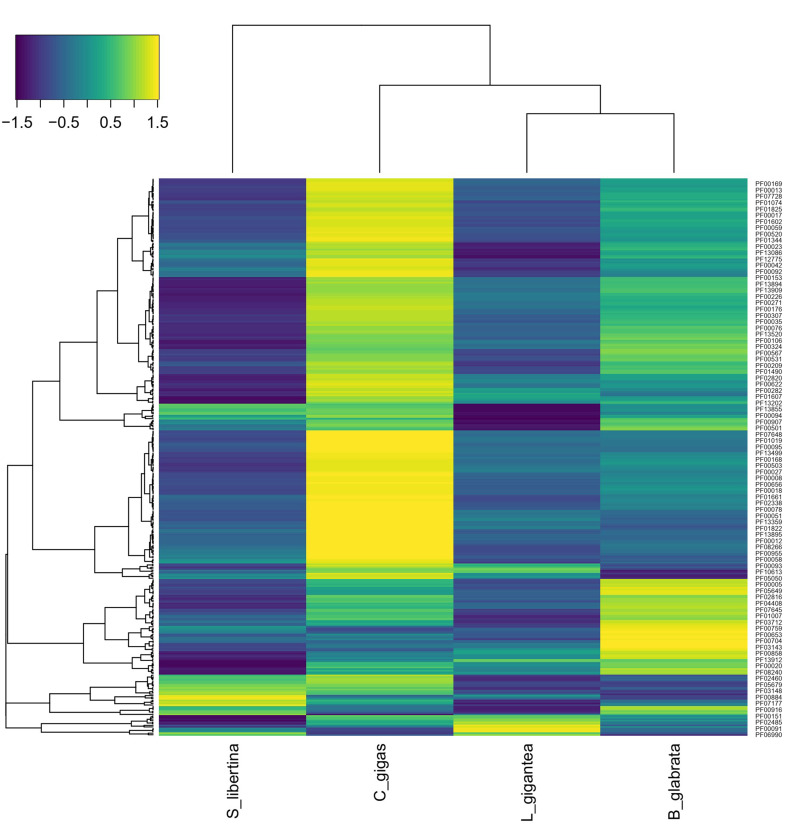
Heatmap presenting copy numbers of orthologous genes in each genome. Each unit was selected if five or more copy numbers were present in the genome (air-breathing freshwater snail, *Biomphalaria glabrata*; oyster, *Crassostrea gigas*; owl limpet, *Lottia gigantea*; freshwater snail, *Semisulcospira libertina*). In the dendrogram, *B. glabrata* and *L. gigantea* were grouped whereas *S. libertina* was outgrouped.

We also provided a table and heatmap presenting the copy number of orthologous genes in each genome (Supplementary Table 3). Fig. 2 provides enriched PFAM domains identified as copy number. In the genome of *C. gigas*, PFAM domains were overrepresented. In the genome of *S. libertina*, domain signals from PFAM had weaker patterns than in genomes of other species. Therefore, the genome of *S. libertina* was distinctively divided into genomes of other three species.

**Fig. 3.**
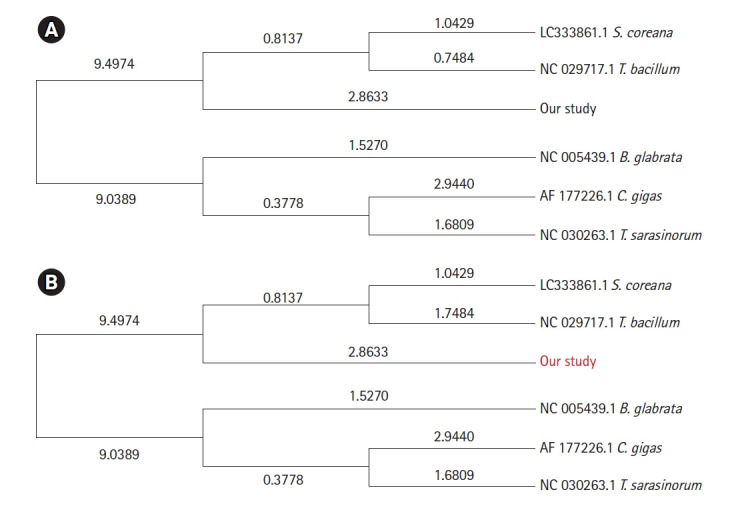
Maximum likelihood tree for whole-mitochondrial genome (A) and cytochrome c oxidase subunit I regions of mitochondrial DNA (B). These dendrograms were derived from mitochondrial genome sequences identified in the GenBank database and sequences obtained from the present study. Values at nodes indicate branch lengths. Branch length is proportional to the distance between taxa.

We also provided a maximum likelihood tree for whole-mitochondrial genome (Fig. 3A) and COI regions of mitochondrial DNA (Fig. 3B). The phylogenetic tree shown in Fig. 3 reflects the relationshp of PFAM domains (Fig. 2). Dendrograms were derived from mitochondrial genome sequences obtained from GenBank database. In the phylogenetic tree of the whole-mitochondrial genome, *S. coreana* and *Turritella bacillum* were grouped (Fig. 3A). However, for COI regions, *S. libertina* and *S. coreana* were grouped as expected (Fig. 3B). As expected, *C. gigas* and *B. glabrata* were outgrouped with *S. libertina* in both analyses (Fig. 3).

## Discussion

The genome of *S. libertina* could provide insights into freshwater shellfish biology such as extraction of useful components and shell body plan. Next-generation sequencing technologies have greatly reduced the cost of whole-genome sequencing. A huge amount of sequencing data have been accumulated and utilized to study substances such as venom and druggable tartgets. However, in comparison with vertebrate genome studies, freshwater snail genome study is still at its infancy. We tried to provide a source for genomics of freshwater snails. Because of its large genome size, we provided a draft genome in this study. Our draft genome has relatively lower sequencing depth (<20×). Therefore, validation steps by other methods such as PCR or targeted sequencing is needed in the future to obtain accurate genetic information. This draft genome could be used for further studies so that biological mechanisms could be elucidated.

Previous studies have shown that genomes of invertebrates have relatively high heterozygosity, and the genome of *S. libertina* might also show high heterozygosity, like genomes of *Dendronephthya gigantea* [31] and *Ruditapes philippinarum* [32]. The genome size of *C. tribblei* was 2.76 Gb [11], and the genome size *R. philippinarum* was 2.56 Gb [32]. The genome size of *S. libertina* was relatively larger than that of other species such as Gastropada class or *R. philippinarum*. *R. auricularia* has a relatively small genome size of 910 Mb [10]. Oyster, *Crassostrea gigas*, has a smaller genome size of 637 Mb [30]. Freshwater snail *B. glabrata* has a genome size of approximately 916 Mb [7]. The genome size of *S. libertina* is very large compared to other similar species, and it is similar to that of humans (3.10 Gb). Evolutionary and phylogenetic approaches to large genome sizes will be needed as future studies.

We calculated the copy number of orthologous genes based on PFAM dataset (Supplementary Table 3). The genome of *C. gigas* had the highest copy number for shell formation related genes. Similar copy number patterns were detected for genomes of *S. libertina* and *B. glabrata*. The genome of *B. glabrata* has different patterns of shell formation proteomes compared to the genome of *C. gigas* [7]. In the genome of *C. gigas*, PIs are highly abundant in shells. The copy number of PIs in four genomes had similar patterns in our analysis. In the genome of *C. gigas*, lectin C-type domain, EF-hand domain pair, and thrombospondin type 1 domain have dramatically higher copy numbers. Lectin C-type‒containing proteins are highly expressed in the digestive gland of *C. gigas* [30]. The copy number was also highly detected in our genome. Two genes (alkaline phosphatase and tyrosinase) related to shell formation showed the highest copy number in *S. libertina* among the four species. It means that freshwater snails could have slightly different copy numbers for shellfish metabolism.

Mitochondrial DNA sequences and COI sequences are useful for species identification. This is because each species has specific patterns in their sequences. We obtained two phylogenetic trees from whole mitochondrial and COI sequences. These trees showed slightly different patterns. COI seqeucnes tended to be more accurate evolutionarily and taxonomically than whole mitochondrial sequence. Thus, COI sequences are used to confirm species identification and geological distribution of *Semisulcospira* genus.

One of the limitations of this study was our assembly with a total length of 1.4 Gb, but the total genome size was 3 Gb. About 46.7% of the assembled sequences are available. If the complete genome is provided through additional sequencing to the sequence provided by us, it is expected to be of great help in genomics studies on Gastropoda species [33]. The sequence information in this study is incomplete, and it will not be easy to profile the genome charasteristics. Based on our study, evolutionary or phylogenetic studies in similar species could be performed by comparing gene family diversity of complete genes.

Here, we identified gene sets of *S. libertina* predicted with *de novo* genome assembly data for the first time. These results may provide clues for ecological studies of freshwater environments and immunological studies of secreted materials of *S. libertina*. Our study may also provide useful information for better understanding of the evolutionary relationship among Gastropoda species.

## Figures and Tables

**Table 1.** Results of gene prediction for the genome of *Semisulcospira libertina*

Parameter	Value
Total No. of gene models predicted	61,610
Annotated gene	39,949
Uniprot	10,065
NCBI nonredundant	19,659
InterProScan	37,333
Average gene length (bp)	424
Average of GC content (%)	53.68

**Table 2.** Number, length, and proportion of repetitive elements in the genome of *Semisulcospira libertina*

Type	No. of Elements	Length (bp)	% in genome
Retrotransposons	1,190,727	224,533,654	15.98
SINEs	102,400	13,600,944	0.97
LINEs	784,492	134,091,004	9.54
LTR elements	303,708	78,375,018	5.58
Retroposon	127	5,771	0.00
DNA transposons	596,425	105,094,630	7.48
DNA	507,157	80,734,772	5.75
RC	89,191	24,777,502	1.76
Other	77	7,150	0.00
Inserted sequence	9	437	0.00
Segmental duplication	3	134	0.00
Unclassified	3,711,311	487,237,955	34.68
Small RNA	3505	444,780	0.03
Satellites	6767	901,035	0.06
Simple repeats	847777	43,050,705	3.06
Low complexity	79335	4,196,255	0.30
Total		832,215,362	59.23

**Table 3.** Summary of simple sequence repeats distribution in the genome of *Semisulcospira libertina*

Repeat type	Frequency	Frequency per million
2	512,774	364.97
3	132,734	94.47
4	86,955	61.89
5	14,883	10.59
6	1,590	1.13
7	241	0.17
8	374	0.27
9	259	0.18
10	247	0.18

**Table 4.** Shell formation related genes (ID and description were obtained from PFAM; species with the highest copy number in four genomes is indicated the in top column)

ID	Description	Highest copy number
Shell formation proteins [30]		
PF00245	Alkaline phosphatase	*S_libertina*
PF00262	Calreticulin	*B_glabrata and C_gigas*
PF03142	Chitin synthase	*C_gigas*
PF14704	Dermatopontin	*C_gigas*
PF00264	Tyrosinase	*S_libertina*
Metabolic transcripts [29]		
PF00067	Cytochrome P450	*C_gigas*
PF00151	Lipase	*L_gigantea*
PF13469	Sulfotransferase family	*C_gigas*
Protease inhibitors [29]		
PF00050	Kazal-type serine protease inhibitor domain	*C_gigas*
PF07648	Kazal-type serine protease inhibitor domain	*C_gigas*
Small matrix proteins [29]		
PF00057	Low-density lipoprotein receptor domain class A	*C_gigas*
PF00058	Low-density lipoprotein receptor repeat class B	*C_gigas*
PF00059	Lectin C-type domain	*C_gigas*
PF00090	Thrombospondin type 1 domain	*C_gigas*
PF01607	Chitin binding Peritrophin-A domain	*C_gigas*
PF02412	Thrombospondin type 3 repeat	*C_gigas*
PF03067	Chitin binding domain	*C_gigas*
PF07645	Calcium-binding EGF domain	*B_glabrata*
PF08976	EF-hand domain	*C_gigas*
PF13405	EF-hand domain	*C_gigas*
PF13499	EF-hand domain pair	*C_gigas*
PF13833	EF-hand domain pair	*C_gigas*
Transmembrane proteins [29]		
PF01146	Caveolin	*C_gigas*
PF05478	Prominin	*C_gigas*
PF14878	Death-like domain of SPT6	*B_glabrata*

## Supplementary Materials

Supplementary Table 1. Summary of genome assembly (k = 77 selected)

Supplementary Table 2. Summary of benchmarking universal single-copy orthologs (BUSCO) analysis (lineage dataset was used as metazoa_odb9; and number of BUSCOs was 978)

Supplementary Table 3. Enriched PFAM domains identified as copy number in four genomes
