# Supplementary material for: Draft genome of Semisulcospira libertina, a species of freshwater snail
Source: Genomics Inform. 2021 Sep 30;19(3):e32. doi: 10.5808/gi.21039 (PMC8510874; doi:10.5808/gi.21039)
Supplement: Supplementary Table 1. — Summary of genome assembly (k = 77 selected) [file gi-21039suppl1.pdf]

Supplementary Table 1. Summary of genome assembly (k = 77 selected)

k value	Contigs	No. of bases	Longest	Shortest	N50	N90
67	720,471	1,366,689,130	47,812	500	2,804	789
**77**	**748,492**	**1,404,984,258**	**53,068**	**500**	**2,788**	**778**
87	764,612	1,387,429,382	52,950	500	2,650	757
97	743,548	1,302,683,958	45,850	500	2,493	744
107	682,929	1,070,198,162	36,901	500	2,078	707
117	531,204	667,859,072	22,138	500	1,487	632
127	133,321	112,175,541	15,611	500	875	532
