# Supplementary material for: Draft genome of Semisulcospira libertina, a species of freshwater snail
Source: Genomics Inform. 2021 Sep 30;19(3):e32. doi: 10.5808/gi.21039 (PMC8510874; doi:10.5808/gi.21039)
Supplement: Supplementary Table 2. — Summary of benchmarking universal single-copy orthologs (BUSCO) analysis (lineage dataset was used as metazoa_odb9; and number of BUSCOs was 978) [file gi-21039suppl2.pdf]

Supplementary Table 2. Summary of benchmarking universal single-copy orthologs (BUSCO) analysis (lineage dataset was used as metazoa_odb9; and number of BUSCOs was 978)

Type	Count	Ratio (%)
Complete BUSCOs	225	23
Complete and single-copy BUSCOs	223	22.8
Complete and duplicated BUSCOs	2	0.2
Fragmented BUSCOs	462	47.2
Missing BUSCOs	291	29.8
