# Supplementary material for: Draft genome of Semisulcospira libertina, a species of freshwater snail
Source: Genomics Inform. 2021 Sep 30;19(3):e32. doi: 10.5808/gi.21039 (PMC8510874; doi:10.5808/gi.21039)
Supplement: Supplementary Table 3. — Enriched PFAM domains identified as copy number in four genomes [file gi-21039suppl3.pdf]

Supplementary Table 3. Enriched PFAM domains identified as copy number in four genomes

PFAM_ACC	PFAM_Name	Description	L_gigantea	B_glabrata	C_gigas	S_libertina
PF00001	7tm_1	7 transmembrane receptor (rhodopsin family)	275	527	626	641
PF00002	7tm_2	7 transmembrane receptor (Secretin family)	58	94	117	42
PF00003	7tm_3	7 transmembrane sweet-taste receptor of 3 GCPR	17	35	16	21
PF00004	AAA	ATPase family associated with various cellular activities (AAA)	40	80	78	18
PF00005	ABC_tran	ABC transporter	48	152	109	36
PF00006	ATP-synt_ab	ATP synthase alpha/beta family, nucleotide-binding domain	4	8	7	5
PF00007	Cys_knot	Cystine-knot domain	2	2	2	4
PF00008	EGF	EGF-like domain	69	103	241	39
PF00009	GTP_EFTU	Elongation factor Tu GTP binding domain	17	31	21	6
PF00010	HLH	Helix-loop-helix DNA-binding domain	77	93	112	34
PF00011	HSP20	Hsp20/alpha crystallin family	10	18	16	11
PF00012	HSP70	Hsp70 protein	16	28	137	22
PF00013	KH_1	KH domain	15	37	72	6
PF00014	Kunitz_BPTI	Kunitz/Bovine pancreatic trypsin inhibitor domain	27	16	64	13
PF00017	SH2	SH2 domain	26	91	168	14
PF00018	SH3_1	SH3 domain	33	74	175	12
PF00019	TGF_beta	Transforming growth factor beta like domain	10	15	20	11
PF00020	TNFR_c6	TNFR/NGFR cysteine-rich region	16	23	20	5
PF00021	UPAR_LY6	u-PAR/Ly-6 domain	3	1	5	0
PF00022	Actin	Actin	20	33	44	20
PF00023	Ank	Ankyrin repeat	17	98	136	55
PF00024	PAN_1	PAN domain	55	25	99	47
PF00025	Arf	ADP-ribosylation factor family	24	33	43	10
PF00026	Asp	Eukaryotic aspartyl protease	5	5	3	2
PF00027	cNMP_binding	Cyclic nucleotide-binding domain	36	36	103	9
PF00028	Cadherin	Cadherin domain	57	127	168	107
PF00031	Cystatin	Cystatin domain	2	11	11	0
PF00032	Cytochrom_B_C	Cytochrome b(C-terminal)/b6/petD	0	2	1	0
PF00033	Cytochrome_B	Cytochrome b/b6/petB	0	1	0	0
PF00034	Cytochrom_C	Cytochrome c	1	3	1	0
PF00035	dsrm	Double-stranded RNA binding motif	11	26	33	7
PF00036	EF-hand_1	EF hand	6	12	37	14
PF00037	Fer4	4Fe-4S binding domain	0	3	2	0
PF00038	Filament	Intermediate filament protein	6	17	11	5
PF00040	fn2	Fibronectin type II domain	1	0	0	0
PF00041	fn3	Fibronectin type III domain	62	115	273	85
PF00042	Globin	Globin	14	21	28	17
PF00043	GST_C	Glutathione S-transferase, C-terminal domain	19	30	31	4
PF00044	Gp_dh_N	Glyceraldehyde 3-phosphate dehydrogenase, NAD binding domain	1	4	1	0
PF00045	Hemopexin	Hemopexin	6	2	8	0
PF00046	Homeodomain	Homeobox domain	133	113	190	51
PF00047	ig	Immunoglobulin domain	11	40	53	16
PF00049	Insulin	Insulin/IGF/Relaxin family	3	2	5	1
PF00050	Kazal_1	Kazal-type serine protease inhibitor domain	7	10	27	1
PF00051	Kringle	Kringle domain	20	14	57	10
PF00052	NA	Laminin B (Domain IV)	6	5	13	2
PF00053	Laminin_EGF	Laminin EGF domain	22	46	69	12
PF00054	Laminin_G_1	Laminin G domain	6	12	27	9
PF00055	Laminin_N	Laminin N-terminal (Domain VI)	10	10	12	7
PF00056	Ldh_1_N	lactate/malate dehydrogenase, NAD binding domain	3	3	3	2
PF00057	Ldl_recept_a	Low-density lipoprotein receptor domain class A	53	108	138	129
PF00058	Ldl_recept_b	Low-density lipoprotein receptor repeat class B	20	23	47	30
PF00059	Lectin_C	Lectin C-type domain	119	243	438	84
PF00060	Lig_chan	Ligand-gated ion channel	48	32	49	40
PF00061	Lipocalin	Lipocalin / cytosolic fatty-acid binding protein family	11	3	22	2
PF00062	Lys	C-type lysozyme/alpha-lactalbumin family	1	0	0	0
PF00063	Myosin_head	Myosin head (motor domain)	37	56	120	27
PF00066	Notch	LNR domain	2	3	7	0
PF00067	p450	Cytochrome P450	75	166	166	122
PF00068	Phospholip_A2_1	Phospholipase A2	1	10	3	1
PF00069	Pkinase	Protein kinase domain	226	543	619	102
PF00070	NA	Pyridine nucleotide-disulphide oxidoreductase	0	0	0	2
PF00071	Ras	Ras family	97	178	272	86
PF00072	Response_reg	Response regulator receiver domain	0	2	2	0
PF00075	RNase_H	RNase H	1	4	1	1
PF00076	RRM_1	RNA recognition motif. (a.k.a. RRM, RBD, or RNP domain)	113	272	315	41
PF00077	RVP	Retroviral aspartyl protease	0	2	3	1
PF00078	RVT_1	Reverse transcriptase (RNA-dependent DNA polymerase)	6	20	78	5
PF00079	Serpin	Serpin (serine protease inhibitor)	8	65	4	15
PF00080	Sod_Cu	Copper/zinc superoxide dismutase (SODC)	9	11	15	1
PF00081	Sod_Fe_N	Iron/manganese superoxide dismutases, alpha-hairpin domain	1	1	1	1
PF00082	Peptidase_S8	Subtilase family	8	12	32	3
PF00083	Sugar_tr	Sugar (and other) transporter	57	81	72	24
PF00084	Sushi	Sushi repeat (SCR repeat)	93	32	130	26
PF00085	Thioredoxin	Thioredoxin	23	40	52	6

PF00086	Thyroglobulin_1	Thyroglobulin type-1 repeat	4	10	21	6
PF00088	Trefoil	Trefoil (P-type) domain	6	3	2	3
PF00089	Trypsin	Trypsin	57	98	42	33
PF00090	TSP_1	Thrombospondin type 1 domain	55	58	187	26
PF00091	Tubulin	Tubulin/FtsZ family, GTPase domain	92	33	18	43
PF00092	VWA	von Willebrand factor type A domain	65	115	215	98
PF00093	VWC	von Willebrand factor type C domain	16	9	22	9
PF00094	VWD	von Willebrand factor type D domain	15	41	52	38
PF00095	WAP	WAP-type (Whey Acidic Protein) 'four-disulfide core'	16	15	65	7
PF00096	zf-C2H2	Zinc finger, C2H2 type	195	138	155	270
PF00097	zf-C3HC4	Zinc finger, C3HC4 type (RING finger)	70	73	146	50
PF00098	zf-CCHC	Zinc knuckle	39	34	58	20
PF00100	Zona_pellucida	Zona pellucida-like domain	9	5	12	8
PF00102	Y_phosphatase	Protein-tyrosine phosphatase	48	107	218	31
PF00104	Hormone_recep	Ligand-binding domain of nuclear hormone receptor	30	70	134	26
PF00105	zf-C4	Zinc finger, C4 type (two domains)	33	79	146	17
PF00106	adh_short	short chain dehydrogenase	76	124	137	27
PF00107	ADH_zinc_N	Zinc-binding dehydrogenase	18	25	23	4
PF00108	Thiolase_N	Thiolase, N-terminal domain	8	12	8	0
PF00109	ketoacyl-synt	Beta-ketoacyl synthase, N-terminal domain	10	13	5	15
PF00110	wnt	wnt family	12	22	27	23
PF00111	Fer2	2Fe-2S iron-sulfur cluster binding domain	8	12	7	2
PF00112	Peptidase_C1	Papain family cysteine protease	22	34	32	6
PF00113	Enolase_C	Enolase, C-terminal TIM barrel domain	2	6	6	4
PF00115	COX1	Cytochrome C and Quinol oxidase polypeptide I	0	2	1	0
PF00116	COX2	Cytochrome C oxidase subunit II, periplasmic domain	0	2	1	0
PF00117	GATase	Glutamine amidotransferase class-I	4	6	8	0
PF00118	Cpn60_TCP1	TCP-1/cpn60 chaperonin family	13	16	22	15
PF00119	ATP-synt_A	ATP synthase A chain	0	1	1	0
PF00120	Gln-synt_C	Glutamine synthetase, catalytic domain	2	11	7	9
PF00121	TIM	Triosephosphate isomerase	1	1	1	0
PF00122	E1-E2_ATPase	E1-E2 ATPase	12	44	60	3
PF00125	Histone	Core histone H2A/H2B/H3/H4	72	12	30	8
PF00126	HTH_1	Bacterial regulatory helix-turn-helix protein, lysR family	0	0	1	0
PF00128	Alpha-amylase	Alpha amylase, catalytic domain	9	20	17	3
PF00130	C1_1	Phorbol esters/diacylglycerol binding domain (C1 domain)	20	60	143	0
PF00131	Metallothio	Metallothionein	0	0	2	0
PF00132	Hexapep	Bacterial transferase hexapeptide (six repeats)	6	10	15	0
PF00133	tRNA-synt_1	tRNA synthetases class I (I, L, M and V)	6	21	11	3
PF00134	Cyclin_N	Cyclin, N-terminal domain	20	28	38	4
PF00135	COesterase	Carboxylesterase family	26	38	56	75
PF00136	DNA_pol_B	DNA polymerase family B	4	7	10	4
PF00137	ATP-synt_C	ATP synthase subunit C	3	3	6	1
PF00144	Beta-lactamase	Beta-lactamase	9	14	33	15
PF00145	DNA_methylase	C-5 cytosine-specific DNA methylase	3	3	6	0
PF00146	NADHdh	NADH dehydrogenase	0	2	1	0
PF00147	Fibrinogen_C	Fibrinogen beta and gamma chains, C-terminal globular domain	69	76	192	170
PF00149	Metallophos	Calcineurin-like phosphoesterase	35	65	69	7
PF00150	Cellulase	Cellulase (glycosyl hydrolase family 5)	0	2	0	0
PF00151	Lipase	Lipase	23	19	23	13
PF00152	tRNA-synt_2	tRNA synthetases class II (D, K and N)	5	12	12	1
PF00153	Mito_carr	Mitochondrial carrier protein	35	54	64	19
PF00154	RecA	recA bacterial DNA recombination protein	0	2	0	0
PF00155	Aminotran_1_2	Aminotransferase class I and II	20	29	37	7
PF00156	Pribosyltran	Phosphoribosyl transferase domain	4	7	6	0
PF00157	Pou	Pou domain - N-terminal to homeobox domain	4	7	11	3
PF00159	Hormone_3	Pancreatic hormone peptide	1	1	2	0
PF00160	Pro_isomerase	Cyclophilin type peptidyl-prolyl cis-trans isomerase/CLD	18	22	18	0
PF00162	PGK	Phosphoglycerate kinase	1	12	2	1
PF00163	Ribosomal_S4	Ribosomal protein S4/S9 N-terminal domain	1	1	2	0
PF00164	Ribosom_S12_S23	Ribosomal protein S12/S23	2	3	2	0
PF00166	Cpn10	Chaperonin 10 Kd subunit	1	1	1	0
PF00167	FGF	Fibroblast growth factor	2	6	11	1
PF00168	C2	C2 domain	73	122	256	23
PF00169	PH	PH domain	71	160	334	5
PF00170	bZIP_1	bZIP transcription factor	17	19	29	3
PF00171	Aldedh	Aldehyde dehydrogenase family	16	36	32	16
PF00173	Cyt-b5	Cytochrome b5-like Heme/Steroid binding domain	14	17	26	8
PF00174	Oxidored_molyb	Oxidoreductase molybdopterin binding domain	1	1	1	1
PF00175	NAD_binding_1	Oxidoreductase NAD-binding domain	8	13	24	1
PF00176	SNF2_N	SNF2 family N-terminal domain	24	47	71	6
PF00177	Ribosomal_S7	Ribosomal protein S7p/S5e	2	5	2	2
PF00178	Ets	Ets-domain	10	22	46	14
PF00179	UQ_con	Ubiquitin-conjugating enzyme	38	36	52	9
PF00180	Iso_dh	Isocitrate/isopropylmalate dehydrogenase	5	7	11	3
PF00181	Ribosomal_L2	Ribosomal Proteins L2, RNA binding domain	2	4	2	0
PF00183	HSP90	Hsp90 protein	3	4	6	5

PF00184	Hormone_5	Neurohypophysial hormones, C-terminal Domain	1	0	1	0
PF00185	OTCace	Aspartate/ornithine carbamoyltransferase, Asp/Orn binding domain	2	3	5	0
PF00186	DHFR_1	Dihydrofolate reductase	2	2	3	1
PF00188	CAP	Cysteine-rich secretory protein family	17	38	47	3
PF00189	Ribosomal_S3_C	Ribosomal protein S3, C-terminal domain	1	2	2	1
PF00191	Annexin	Annexin	16	12	23	8
PF00194	Carb_anhydrase	Eukaryotic-type carbonic anhydrase	17	20	47	15
PF00198	2-oxoacid_dh	2-oxoacid dehydrogenases acyltransferase (catalytic domain)	2	7	4	2
PF00199	Catalase	Catalase	1	3	8	1
PF00200	Disintegrin	Disintegrin	6	26	9	6
PF00201	UDPGT	UDP-glucoronosyl and UDP-glucosyl transferase	8	16	0	14
PF00202	Aminotran_3	Aminotransferase class-III	5	11	9	4
PF00203	Ribosomal_S19	Ribosomal protein S19	2	2	1	0
PF00204	DNA_gyraseB	DNA gyrase B	2	6	2	1
PF00205	TPP_enzyme_M	Thiamine pyrophosphate enzyme, central domain	3	1	2	0
PF00206	Lyase_1	Lyase	3	11	4	1
PF00207	A2M	Alpha-2-macroglobulin family	13	19	14	2
PF00208	ELFV_dehydrog	Glutamate/Leucine/Phenylalanine/Valine dehydrogenase	1	2	3	0
PF00209	SNF	Sodium:neurotransmitter symporter family	43	66	73	49
PF00210	Ferritin	Ferritin-like domain	5	9	8	0
PF00211	Guanylate_cyc	Adenylate and Guanylate cyclase catalytic domain	48	49	77	25
PF00213	OSCP	ATP synthase delta (OSCP) subunit	1	1	1	0
PF00215	OMPdecase	Orotidine 5'-phosphate decarboxylase / HUMPS family	1	1	1	0
PF00217	ATP-gua_Ptrans	ATP:guanido phosphotransferase, C-terminal catalytic domain	5	8	2	5
PF00219	IGFBP	Insulin-like growth factor binding protein	6	2	5	3
PF00221	Lyase_aromatic	Aromatic amino acid lyase	1	1	1	1
PF00224	PK	Pyruvate kinase, barrel domain	2	13	4	3
PF00225	Kinesin	Kinesin motor domain	41	66	133	17
PF00226	DnaJ	DnaJ domain	33	53	73	5
PF00227	Proteasome	Proteasome subunit	16	17	18	1
PF00229	TNF	TNF(Tumour Necrosis Factor) family	6	35	46	4
PF00230	MIP	Major intrinsic protein	16	19	30	8
PF00231	ATP-synt	ATP synthase	1	2	3	1
PF00232	Glyco_hydro_1	Glycosyl hydrolase family 1	7	9	3	96
PF00233	PDEase_I	3'5'-cyclic nucleotide phosphodiesterase	14	28	89	4
PF00235	Profilin	Profilin	7	9	10	10
PF00237	Ribosomal_L22	Ribosomal protein L22p/L17e	2	5	3	0
PF00238	Ribosomal_L14	Ribosomal protein L14p/L23e	2	4	2	1
PF00240	ubiquitin	Ubiquitin family	36	47	41	4
PF00241	Cofilin_ADF	Cofilin/tropomyosin-type actin-binding protein	6	14	18	2
PF00243	NGF	Nerve growth factor family	2	3	2	1
PF00244	41701	14-3-3 protein	4	4	4	4
PF00245	Alk_phosphatase	Alkaline phosphatase	2	12	13	21
PF00246	Peptidase_M14	Zinc carboxypeptidase	12	24	45	10
PF00248	Aldo_ket_red	Aldo/keto reductase family	21	15	17	5
PF00249	Myb_DNA-binding	Myb-like DNA-binding domain	14	36	35	0
PF00250	Forkhead	Forkhead domain	30	24	38	16
PF00252	Ribosomal_L16	Ribosomal protein L16p/L10e	2	5	4	2
PF00253	Ribosomal_S14	Ribosomal protein S14p/S29e	2	0	1	0
PF00254	FKBP_C	FKBP-type peptidyl-prolyl cis-trans isomerase	13	18	25	0
PF00255	GSHPx	Glutathione peroxidase	4	9	8	4
PF00258	Flavodoxin_1	Flavodoxin	5	8	14	1
PF00261	Tropomyosin	Tropomyosin	1	18	16	0
PF00262	Calreticulin	Calreticulin family	2	4	4	0
PF00264	Tyrosinase	Common central domain of tyrosinase	3	21	30	34
PF00265	TK	Thymidine kinase	1	1	2	0
PF00266	Aminotran_5	Aminotransferase class-V	13	17	17	2
PF00268	Ribonuc_red_sm	Ribonucleotide reductase, small chain	1	2	1	3
PF00270	DEAD	DEAD/DEAH box helicase	53	86	126	7
PF00271	Helicase_C	Helicase conserved C-terminal domain	80	135	191	20
PF00274	Glycolytic	Fructose-bisphosphate aldolase class-I	1	4	5	1
PF00275	EPSP_synthase	EPSP synthase (3-phosphoshikimate 1-carboxyvinyltransferase)	0	1	0	0
PF00276	Ribosomal_L23	Ribosomal protein L23	2	3	4	0
PF00277	SAA	Serum amyloid A protein	0	0	1	0
PF00278	Orn_DAP_Arg_deC	Pyridoxal-dependent decarboxylase, C-terminal sheet domain	2	4	5	3
PF00280	potato_inhibit	Potato inhibitor I family	1	0	0	0
PF00281	Ribosomal_L5	Ribosomal protein L5	1	2	1	1
PF00282	Pyridoxal_deC	Pyridoxal-dependent decarboxylase conserved domain	13	12	18	6
PF00285	Citrate_synt	Citrate synthase	2	6	7	1
PF00287	Na_K-ATPase	Sodium / potassium ATPase beta chain	3	7	2	4
PF00288	GHMP_kinases_N	GHMP kinases N terminal domain	5	9	6	1
PF00289	Biotin_carb_N	Carbamoyl-phosphate synthase L chain, N-terminal domain	4	5	9	0
PF00290	Trp_syntA	Tryptophan synthase alpha chain	0	0	1	0
PF00291	PALP	Pyridoxal-phosphate dependent enzyme	11	10	25	8
PF00292	PAX	'Paired box' domain	7	5	33	4
PF00293	NUDIX	NUDIX domain	17	46	21	1
PF00294	PfkB	pfkB family carbohydrate kinase	6	10	6	4

PF00297	Ribosomal_L3	Ribosomal protein L3	2	2	3	1
PF00298	Ribosomal_L11	Ribosomal protein L11, RNA binding domain	1	4	4	1
PF00300	His_Phos_1	Histidine phosphatase superfamily (branch 1)	5	22	18	0
PF00303	Thymidylat_synt	Thymidylate synthase	1	3	1	0
PF00305	Lipoxygenase	Lipoxygenase	4	9	18	7
PF00306	ATP-synt_ab_C	ATP synthase alpha/beta chain, C terminal domain	4	5	8	1
PF00307	CH	Calponin homology (CH) domain	39	115	169	10
PF00308	NA	Bacterial dnaA protein	0	2	0	0
PF00310	GATase_2	Glutamine amidotransferases class-II	1	2	4	1
PF00312	Ribosomal_S15	Ribosomal protein S15	2	2	3	0
PF00313	CSD	'Cold-shock' DNA-binding domain	4	6	7	1
PF00314	Thaumatin	Thaumatin family	0	0	1	0
PF00316	FBPase	Fructose-1-6-bisphosphatase	0	1	1	1
PF00317	Ribonuc_red_lgN	Ribonucleotide reductase, all-alpha domain	1	2	3	0
PF00318	Ribosomal_S2	Ribosomal protein S2	2	4	2	1
PF00319	SRF-TF	SRF-type transcription factor (DNA-binding and dimerisation domain)	3	4	5	1
PF00320	GATA	GATA zinc finger	4	16	15	2
PF00324	AA_permease	Amino acid permease	7	10	10	5
PF00326	Peptidase_S9	Prolyl oligopeptidase family	5	10	30	3
PF00327	Ribosomal_L30	Ribosomal protein L30p/L7e	1	2	2	0
PF00328	His_Phos_2	Histidine phosphatase superfamily (branch 2)	7	15	8	3
PF00329	Complex1_30kDa	Respiratory-chain NADH dehydrogenase, 30 Kd subunit	1	1	3	0
PF00330	Aconitase	Aconitase family (aconitate hydratase)	2	3	6	2
PF00331	Glyco_hydro_10	Glycosyl hydrolase family 10	13	8	7	14
PF00333	Ribosomal_S5	Ribosomal protein S5, N-terminal domain	2	3	3	1
PF00334	NDK	Nucleoside diphosphate kinase	6	13	27	4
PF00335	Tetraspanin	Tetraspanin family	39	53	129	7
PF00337	Gal-bind_lectin	Galactoside-binding lectin	2	24	24	5
PF00338	Ribosomal_S10	Ribosomal protein S10p/S20e	3	6	3	0
PF00339	Arrestin_N	Arrestin (or S-antigen), N-terminal domain	18	34	53	8
PF00341	PDGF	PDGF/VEGF domain	2	1	8	3
PF00342	PGI	Phosphoglucose isomerase	1	2	2	0
PF00343	Phosphorylase	Carbohydrate phosphorylase	1	2	5	4
PF00344	SecY	SecY translocase	1	1	3	1
PF00346	Complex1_49kDa	Respiratory-chain NADH dehydrogenase, 49 Kd subunit	1	2	1	1
PF00347	Ribosomal_L6	Ribosomal protein L6	1	4	3	1
PF00348	polyprenyl_synt	Polyprenyl synthetase	4	7	4	0
PF00349	Hexokinase_1	Hexokinase	3	4	7	0
PF00350	Dynamin_N	Dynamin family	8	10	48	4
PF00351	Biopterin_H	Biopterin-dependent aromatic amino acid hydroxylase	4	4	7	3
PF00352	TBP	Transcription factor TFIID (or TATA-binding protein, TBP)	1	2	2	0
PF00354	Pentaxin	Pentaxin family	2	1	2	4
PF00355	Rieske	Rieske [2Fe-2S] domain	6	14	12	0
PF00357	Integrin_alpha	Integrin alpha cytoplasmic region	1	0	0	0
PF00358	PTS_EIIA_1	phosphoenolpyruvate-dependent sugar phosphotransferase system, EIIA 1	0	1	0	0
PF00359	PTS_EIIA_2	Phosphoenolpyruvate-dependent sugar phosphotransferase system, EIIA 2	1	1	5	0
PF00361	Proton_antipo_M	Proton-conducting membrane transporter	0	5	3	0
PF00362	Integrin_beta	Integrin, beta chain	3	6	13	0
PF00364	Biotin_lipoyl	Biotin-requiring enzyme	7	10	12	3
PF00365	PFK	Phosphofructokinase	3	6	2	3
PF00366	Ribosomal_S17	Ribosomal protein S17	1	3	1	0
PF00367	PTS_EIIB	phosphotransferase system, EIIB	0	1	0	0
PF00368	HMG-CoA_red	Hydroxymethylglutaryl-coenzyme A reductase	1	1	1	1
PF00370	FGGY_N	FGGY family of carbohydrate kinases, N-terminal domain	5	7	6	1
PF00373	FERM_M	FERM central domain	21	68	112	5
PF00375	SDF	Sodium:dicarboxylate symporter family	8	18	31	16
PF00378	ECH_1	Enoyl-CoA hydratase/isomerase	14	22	20	4
PF00380	Ribosomal_S9	Ribosomal protein S9/S16	1	3	2	0
PF00381	PTS-HPr	PTS HPr component phosphorylation site	0	0	1	0
PF00382	TFIIB	Transcription factor TFIIB repeat	3	2	3	0
PF00383	dCMP_cyt_deam_1	Cytidine and deoxycytidylate deaminase zinc-binding region	4	6	6	0
PF00384	Molybdopterin	Molybdopterin oxidoreductase	1	3	2	1
PF00385	Chromo	Chromo (CHRromatin Organisation MOdifier) domain	5	16	41	4
PF00386	C1q	C1q domain	7	48	376	266
PF00387	PI-PLC-Y	Phosphatidylinositol-specific phospholipase C, Y domain	5	12	36	2
PF00388	PI-PLC-X	Phosphatidylinositol-specific phospholipase C, X domain	6	13	32	3
PF00389	2-Hacid_dh	D-isomer specific 2-hydroxyacid dehydrogenase, catalytic domain	7	15	13	3
PF00390	malic	Malic enzyme, N-terminal domain	3	3	2	1
PF00391	PEP-utilizers	PEP-utilising enzyme, mobile domain	0	3	1	0
PF00393	6PGD	6-phosphogluconate dehydrogenase, C-terminal domain	1	1	1	1
PF00394	Cu-oxidase	Multicopper oxidase	6	18	45	15
PF00396	Granulin	Granulin	3	4	5	1
PF00397	WW	WW domain	21	34	61	1
PF00398	RrnaAD	Ribosomal RNA adenine dimethylase	3	7	3	0
PF00400	WD40	WD domain, G-beta repeat	177	265	394	34
PF00402	NA	Calponin family repeat	4	9	11	3
PF00403	HMA	Heavy-metal-associated domain	3	8	8	2

PF00405	Transferrin	Transferrin	2	9	1	3
PF00406	ADK	Adenylate kinase	12	13	21	3
PF00408	PGM_PMM_IV	Phosphoglucomutase/phosphomannomutase, C-terminal domain	3	4	6	0
PF00410	Ribosomal_S8	Ribosomal protein S8	1	1	1	0
PF00411	Ribosomal_S11	Ribosomal protein S11	2	5	4	1
PF00412	LIM	LIM domain	33	48	107	17
PF00413	Peptidase_M10	Matrixin	8	5	8	5
PF00415	RCC1	Regulator of chromosome condensation (RCC1) repeat	18	33	31	12
PF00416	Ribosomal_S13	Ribosomal protein S13/S18	2	3	1	1
PF00418	Tubulin-binding	Tau and MAP protein, tubulin-binding repeat	4	2	3	1
PF00428	Ribosomal_60s	60s Acidic ribosomal protein	2	4	5	1
PF00431	CUB	CUB domain	65	127	158	101
PF00432	Prenyltrans	Prenyltransferase and squalene oxidase repeat	3	4	4	3
PF00433	Pkinase_C	Protein kinase C terminal domain	11	17	25	2
PF00435	Spectrin	Spectrin repeat	13	51	56	30
PF00436	SSB	Single-strand binding protein family	1	1	1	0
PF00437	T2SSE	Type II/IV secretion system protein	1	0	0	1
PF00438	S-AdoMet_synt_N	S-adenosylmethionine synthetase, N-terminal domain	2	3	6	1
PF00439	Bromodomain	Bromodomain	22	43	83	3
PF00441	Acyl-CoA_dh_1	Acyl-CoA dehydrogenase, C-terminal domain	14	24	13	5
PF00443	UCH	Ubiquitin carboxyl-terminal hydrolase	34	54	64	12
PF00444	Ribosomal_L36	Ribosomal protein L36	1	0	0	0
PF00445	Ribonuclease_T2	Ribonuclease T2 family	3	1	10	0
PF00447	HSF_DNA-bind	HSF-type DNA-binding	3	2	2	1
PF00448	SRP54	SRP54-type protein, GTPase domain	2	6	2	2
PF00449	Urease_alpha	Urease alpha-subunit, N-terminal domain	1	1	0	1
PF00450	Peptidase_S10	Serine carboxypeptidase	6	26	5	2
PF00452	Bcl-2	Apoptosis regulator proteins, Bcl-2 family	7	22	15	4
PF00453	Ribosomal_L20	Ribosomal protein L20	1	2	2	0
PF00454	PI3_PI4_kinase	Phosphatidylinositol 3- and 4-kinase	15	23	25	6
PF00456	Transketolase_N	Transketolase, thiamine diphosphate binding domain	1	2	2	1
PF00458	WHEP-TRS	WHEP-TRS domain	3	3	7	1
PF00459	Inositol_P	Inositol monophosphatase family	4	7	4	2
PF00460	Flg_bb_rod	Flagella basal body rod protein	0	0	1	0
PF00462	Glutaredoxin	Glutaredoxin	8	13	17	0
PF00464	SHMT	Serine hydroxymethyltransferase	3	4	2	1
PF00465	Fe-ADH	Iron-containing alcohol dehydrogenase	1	2	2	1
PF00466	Ribosomal_L10	Ribosomal protein L10	3	2	3	0
PF00467	KOW	KOW motif	2	6	3	1
PF00472	RF-1	RF-1 domain	2	8	3	0
PF00474	NA	Sodium:solute symporter family	36	43	60	16
PF00476	DNA_pol_A	DNA polymerase family A	3	4	6	1
PF00478	IMPDH	IMP dehydrogenase / GMP reductase domain	2	12	12	0
PF00479	G6PD_N	Glucose-6-phosphate dehydrogenase, NAD binding domain	2	5	4	0
PF00481	PP2C	Protein phosphatase 2C	13	22	23	12
PF00483	NTP_transferase	Nucleotidyl transferase	4	6	12	0
PF00484	Pro_CA	Carbonic anhydrase	1	8	1	0
PF00485	PRK	Phosphoribulokinase / Uridine kinase family	3	5	6	1
PF00487	FA_desaturase	Fatty acid desaturase	9	18	14	2
PF00488	MutS_V	MutS domain V	5	8	10	1
PF00490	ALAD	Delta-aminolevulinic acid dehydratase	1	1	1	1
PF00491	Arginase	Arginase family	4	9	2	2
PF00493	MCM	MCM2/3/5 family	8	10	15	6
PF00494	SQS_PSY	Squalene/phytoene synthase	2	2	1	0
PF00497	SBP_bac_3	Bacterial extracellular solute-binding proteins, family 3	6	12	6	2
PF00498	FHA	FHA domain	21	34	75	3
PF00501	AMP-binding	AMP-binding enzyme	35	74	68	53
PF00503	G-alpha	G-protein alpha subunit	9	10	14	7
PF00505	HMG_box	HMG (high mobility group) box	26	52	73	8
PF00507	Oxidored_q4	NADH-ubiquinone/plastoquinone oxidoreductase, chain 3	0	1	1	0
PF00510	COX3	Cytochrome c oxidase subunit III	0	1	1	0
PF00514	Arm	Armadillo/beta-catenin-like repeat	14	21	41	9
PF00515	TPR_1	Tetratricopeptide repeat	36	72	69	5
PF00520	Ion_trans	Ion transport protein	65	161	292	61
PF00521	DNA_topoisoIV	DNA gyrase/topoisomerase IV, subunit A	2	6	1	2
PF00528	BPD_transp_1	Binding-protein-dependent transport system inner membrane component	0	0	1	0
PF00530	SRCR	Scavenger receptor cysteine-rich domain	22	18	80	71
PF00531	Death	Death domain	25	68	72	18
PF00533	BRCT	BRCA1 C Terminus (BRCT) domain	7	16	14	1
PF00534	Glycos_transf_1	Glycosyl transferases group 1	5	7	7	2
PF00535	Glycos_transf_2	Glycosyl transferase family 2	29	43	36	5
PF00536	SAM_1	SAM domain (Sterile alpha motif)	33	62	114	12
PF00538	Linker_histone	linker histone H1 and H5 family	15	14	16	15
PF00542	Ribosomal_L12	Ribosomal protein L7/L12 C-terminal domain	1	3	1	0
PF00547	Urease_gamma	Urease, gamma subunit	1	1	0	0
PF00549	Ligase_CoA	CoA-ligase	4	8	8	1
PF00550	PP-binding	Phosphopantetheine attachment site	5	14	8	7

PF00551	Formyl_trans_N	Formyl transferase	3	8	8	1
PF00553	CBM_2	Cellulose binding domain	2	8	0	0
PF00554	RHD_DNA_bind	Rel homology DNA-binding domain	4	4	8	2
PF00557	Peptidase_M24	Metallopeptidase family M24	9	31	17	3
PF00558	Vpu	Vpu protein	0	0	1	0
PF00560	LRR_1	Leucine Rich Repeat	19	34	41	28
PF00561	Abhydrolase_1	alpha/beta hydrolase fold	14	36	23	4
PF00562	RNA_pol_Rpb2_6	RNA polymerase Rpb2, domain 6	3	7	5	2
PF00564	PB1	PB1 domain	5	13	11	2
PF00565	SNase	Staphylococcal nuclease homologue	1	1	1	0
PF00566	RabGAP-TBC	Rab-GTPase-TBC domain	27	64	77	7
PF00567	TUDOR	Tudor domain	14	29	28	13
PF00568	WH1	WH1 domain	6	9	28	1
PF00569	ZZ	Zinc finger, ZZ type	12	35	45	2
PF00570	HRDC	HRDC domain	1	5	6	0
PF00571	CBS	CBS domain	7	22	56	0
PF00572	Ribosomal_L13	Ribosomal protein L13	2	2	2	0
PF00573	Ribosomal_L4	Ribosomal protein L4/L1 family	2	3	3	0
PF00574	CLP_protease	Clp protease	1	2	1	0
PF00575	S1	S1 RNA binding domain	6	7	10	2
PF00576	Transthyretin	HIUase/Transthyretin family	0	0	7	0
PF00578	AhpC-TSA	AhpC/TSA family	4	7	3	0
PF00579	tRNA-synt_1b	tRNA synthetases class I (W and Y)	4	11	7	0
PF00580	UvrD-helicase	UvrD/REP helicase N-terminal domain	1	2	8	0
PF00581	Rhodanese	Rhodanese-like domain	41	22	36	6
PF00582	Usp	Universal stress protein family	14	36	50	6
PF00583	Acetyltransf_1	Acetyltransferase (GNAT) family	13	23	36	3
PF00584	SecE	SecE/Sec61-gamma subunits of protein translocation complex	1	0	0	0
PF00586	AIRS	AIR synthase related protein, N-terminal domain	2	3	3	0
PF00587	tRNA-synt_2b	tRNA synthetase class II core domain (G, H, P, S and T)	8	13	10	1
PF00588	SpoU_methylase	SpoU rRNA Methylase family	0	5	4	2
PF00589	Phage_integrase	Phage integrase family	6	1	17	0
PF00590	TP_methylase	Tetrapyrrole (Corrin/Porphyrin) Methylases	1	1	1	0
PF00591	Glycos_transf_3	Glycosyl transferase family, a/b domain	1	4	3	1
PF00595	PDZ	PDZ domain (Also known as DHR or GLGF)	79	182	340	20
PF00596	Aldolase_II	Class II Aldolase and Adducin N-terminal domain	2	4	14	1
PF00605	IRF	Interferon regulatory factor transcription factor	2	21	10	1
PF00609	NA	Diacylglycerol kinase accessory domain	4	4	31	0
PF00610	DEP	Domain found in Dishevelled, Egl-10, and Pleckstrin (DEP)	11	22	53	3
PF00611	FCH	Fes/CIP4, and EFC/F-BAR homology domain	8	19	46	1
PF00612	IQ	IQ calmodulin-binding motif	29	75	142	11
PF00613	PI3Ka	Phosphoinositide 3-kinase family, accessory domain (PIK domain)	5	10	12	2
PF00614	PLDc	Phospholipase D Active site motif	2	4	8	0
PF00615	RGS	Regulator of G protein signaling domain	14	34	80	2
PF00616	RasGAP	GTPase-activator protein for Ras-like GTPase	6	19	41	0
PF00617	RasGEF	RasGEF domain	14	37	77	2
PF00618	RasGEF_N	RasGEF N-terminal motif	7	26	51	3
PF00619	CARD	Caspase recruitment domain	22	45	68	16
PF00620	RhoGAP	RhoGAP domain	27	97	179	13
PF00621	RhoGEF	RhoGEF domain	31	63	193	8
PF00622	SPRY	SPRY domain	18	25	47	5
PF00623	RNA_pol_Rpb1_2	RNA polymerase Rpb1, domain 2	3	5	3	3
PF00625	Guanylate_kin	Guanylate kinase	12	28	66	1
PF00626	Gelsolin	Gelsolin repeat	11	13	45	3
PF00627	UBA	UBA/TS-N domain	12	19	26	4
PF00628	PHD	PHD-finger	32	48	101	3
PF00629	MAM	MAM domain, meprin/A5/mu	16	35	86	78
PF00630	Filamin	Filamin/ABP280 repeat	10	23	60	11
PF00631	G-gamma	GGL domain	4	6	21	5
PF00632	HECT	HECT-domain (ubiquitin-transferase)	20	33	43	18
PF00633	HHH	Helix-hairpin-helix motif	1	6	2	0
PF00634	BRCA2	BRCA2 repeat	1	1	1	1
PF00635	Motile_Sperm	MSP (Major sperm protein) domain	3	7	10	1
PF00636	Ribonuclease_3	Ribonuclease III domain	2	2	5	2
PF00637	Clathrin	Region in Clathrin and VPS	9	7	11	4
PF00638	Ran_BP1	RanBP1 domain	4	4	10	2
PF00639	Rotamase	PPIC-type PPIASE domain	0	1	1	0
PF00640	PID	Phosphotyrosine interaction domain (PTB/PID)	15	35	84	1
PF00641	zf-RanBP	Zn-finger in Ran binding protein and others	5	16	29	5
PF00642	zf-CCCH	Zinc finger C-x8-C-x5-C-x3-H type (and similar)	19	29	46	5
PF00643	zf-B_box	B-box zinc finger	45	47	611	84
PF00644	PARP	Poly(ADP-ribose) polymerase catalytic domain	17	64	68	14
PF00645	zf-PARP	Poly(ADP-ribose) polymerase and DNA-Ligase Zn-finger region	2	2	10	1
PF00646	F-box	F-box domain	6	13	11	4
PF00647	NA	Elongation factor 1 gamma, conserved domain	1	1	2	0
PF00648	Peptidase_C2	Calpain family cysteine protease	14	50	61	11
PF00650	CRAL_TRIO	CRAL/TRIO domain	8	27	17	3

PF00651	BTB	BTB/POZ domain	77	197	328	78
PF00652	Ricin_B_lectin	Ricin-type beta-trefoil lectin domain	13	30	26	1
PF00653	BIR	Inhibitor of Apoptosis domain	23	171	68	25
PF00654	Voltage_CLC	Voltage gated chloride channel	6	11	23	3
PF00656	Peptidase_C14	Caspase domain	18	32	76	13
PF00657	Lipase_GDSL	GDSL-like Lipase/Acylhydrolase	6	5	4	0
PF00658	PABP	Poly-adenylate binding protein, unique domain	2	4	4	2
PF00659	POLO_box	POLO box duplicated region	7	7	5	2
PF00662	Proton_antipo_N	NADH-Ubiquinone oxidoreductase (complex I), chain 5 N-terminus	0	1	0	0
PF00664	ABC_membrane	ABC transporter transmembrane region	22	63	48	22
PF00665	rve	Integrase core domain	0	43	117	1
PF00667	FAD_binding_1	FAD binding domain	4	7	13	2
PF00668	Condensation	Condensation domain	3	1	0	1
PF00669	Flagellin_N	Bacterial flagellin N-terminal helical region	0	0	1	0
PF00670	AdoHcyase_NAD	S-adenosyl-L-homocysteine hydrolase, NAD binding domain	2	6	9	2
PF00673	Ribosomal_L5_C	ribosomal L5P family C-terminus	1	2	1	0
PF00675	Peptidase_M16	Insulinase (Peptidase family M16)	7	14	7	0
PF00676	E1_dh	Dehydrogenase E1 component	6	6	11	6
PF00679	EFG_C	Elongation factor G C-terminus	6	9	8	3
PF00681	Plectin	Plectin repeat	1	1	9	3
PF00682	HMGL-like	HMGL-like	2	1	10	2
PF00683	NA	TB domain	1	1	2	2
PF00684	NA	DnaJ central domain	3	5	4	1
PF00685	Sulfotransfer_1	Sulfotransferase domain	37	71	85	52
PF00686	CBM_20	Starch binding domain	9	5	8	1
PF00687	Ribosomal_L1	Ribosomal protein L1p/L10e family	4	7	4	0
PF00688	TGFb_propeptide	TGF-beta propeptide	9	16	18	6
PF00689	Cation_ATPase_C	Cation transporting ATPase, C-terminus	5	10	5	6
PF00690	Cation_ATPase_N	Cation transporter/ATPase, N-terminus	6	13	22	2
PF00691	OmpA	OmpA family	0	1	0	0
PF00692	dUTPase	dUTPase	1	1	3	0
PF00694	Aconitase_C	Aconitase C-terminal domain	2	4	6	1
PF00696	AA_kinase	Amino acid kinase family	2	9	2	1
PF00698	Acyl_transf_1	Acyl transferase domain	7	7	4	5
PF00699	Urease_beta	Urease beta subunit	1	1	0	0
PF00700	Flagellin_C	Bacterial flagellin C-terminal helical region	0	0	1	0
PF00701	DHDPS	Dihydrodipicolinate synthetase family	3	4	6	0
PF00702	Hydrolase	haloacid dehalogenase-like hydrolase	3	14	14	3
PF00703	Glyco_hydro_2	Glycosyl hydrolases family 2	4	2	5	0
PF00704	Glyco_hydro_18	Glycosyl hydrolases family 18	25	65	25	27
PF00705	PCNA_N	Proliferating cell nuclear antigen, N-terminal domain	1	1	1	0
PF00707	IF3_C	Translation initiation factor IF-3, C-terminal domain	0	3	0	1
PF00708	Acylphosphatase	Acylphosphatase	2	2	3	0
PF00709	Adenylsucc_synt	Adenylosuccinate synthetase	1	2	1	1
PF00710	Asparaginase	Asparaginase	1	10	7	1
PF00717	Peptidase_S24	Peptidase S24-like	2	1	5	0
PF00719	Pyrophosphatase	Inorganic pyrophosphatase	1	1	3	0
PF00722	Glyco_hydro_16	Glycosyl hydrolases family 16	10	10	4	0
PF00723	Glyco_hydro_15	Glycosyl hydrolases family 15	2	12	15	0
PF00725	3HCDH	3-hydroxyacyl-CoA dehydrogenase, C-terminal domain	5	7	4	1
PF00728	Glyco_hydro_20	Glycosyl hydrolase family 20, catalytic domain	11	21	27	7
PF00730	HhH-GPD	HhH-GPD superfamily base excision DNA repair protein	2	9	5	1
PF00731	AIRC	AIR carboxylase	1	1	1	0
PF00732	GMC_oxred_N	GMC oxidoreductase	3	23	28	9
PF00733	Asn_synthase	Asparagine synthase	3	3	2	3
PF00735	Septin	Septin	4	15	25	0
PF00736	EF1_GNE	EF-1 guanine nucleotide exchange domain	1	4	6	0
PF00743	FMO-like	Flavin-binding monooxygenase-like	5	9	18	20
PF00749	tRNA-synt_1c	tRNA synthetases class I (E and Q), catalytic domain	3	4	4	0
PF00750	tRNA-synt_1d	tRNA synthetases class I (R)	2	3	4	0
PF00751	DM	DM DNA binding domain	4	5	4	3
PF00752	XPG_N	XPG N-terminal domain	6	7	8	0
PF00753	Lactamase_B	Metallo-beta-lactamase superfamily	5	12	11	0
PF00754	F5_F8_type_C	F5/8 type C domain	39	32	114	17
PF00755	Carn_acyltransf	Choline/Carnitine o-acyltransferase	8	21	14	10
PF00756	Esterase	Putative esterase	1	2	1	0
PF00757	Furin-like	Furin-like cysteine rich region	6	5	13	0
PF00759	Glyco_hydro_9	Glycosyl hydrolase family 9	13	50	9	23
PF00762	Ferrochelatase	Ferrochelatase	1	1	1	0
PF00763	THF_DHG_CYH	Tetrahydrofolate dehydrogenase/cyclohydrolase, catalytic domain	2	7	2	1
PF00764	Arginosuc_synth	Arginosuccinate synthase	1	2	1	1
PF00766	ETF_alpha	Electron transfer flavoprotein FAD-binding domain	1	1	1	0
PF00769	ERM	Ezrin/radixin/moesin family	3	3	9	0
PF00770	Peptidase_C5	Adenovirus endoprotease	12	0	0	0
PF00772	DnaB	DnaB-like helicase N terminal domain	0	1	0	0
PF00773	RNB	RNB domain	7	8	19	12
PF00775	Dioxygenase_C	Dioxygenase	0	0	3	0

PF00778	DIX	DIX domain	4	12	15	0
PF00779	BTK	BTK motif	2	6	4	0
PF00780	NA	CNH domain	7	13	24	1
PF00781	DAGK_cat	Diacylglycerol kinase catalytic domain	9	17	45	1
PF00782	DSPc	Dual specificity phosphatase, catalytic domain	25	45	64	11
PF00784	MyTH4	MyTH4 domain	5	12	56	0
PF00786	PBD	P21-Rho-binding domain	4	12	13	0
PF00787	PX	PX domain	27	44	49	3
PF00788	RA	Ras association (RalGDS/AF-6) domain	13	47	129	5
PF00789	UBX	UBX domain	11	18	21	3
PF00790	VHS	VHS domain	4	15	11	0
PF00791	ZU5	ZU5 domain	5	29	14	22
PF00792	PI3K_C2	Phosphoinositide 3-kinase C2	3	9	6	0
PF00794	PI3K_rbd	PI3-kinase family, ras-binding domain	2	7	4	0
PF00795	CN_hydrolase	Carbon-nitrogen hydrolase	7	9	6	0
PF00797	Acetyltransf_2	N-acetyltransferase	4	4	0	0
PF00801	PKD	PKD domain	4	5	8	10
PF00804	Syntaxin	Syntaxin	3	5	6	2
PF00805	Pentapeptide	Pentapeptide repeats (8 copies)	1	1	4	1
PF00806	PUF	Pumilio-family RNA binding repeat	1	7	3	3
PF00808	CBFD_NFYB_HMF	Histone-like transcription factor (CBF/NF-Y) and archaeal histone	6	13	8	0
PF00809	Pterin_bind	Pterin binding enzyme	1	1	1	2
PF00810	ER_lumen_recept	ER lumen protein retaining receptor	1	2	2	1
PF00811	Ependymin	Ependymin	16	13	32	2
PF00812	Ephrin	Ephrin	1	1	2	0
PF00814	Peptidase_M22	Glycoprotease family	2	5	3	0
PF00817	IMS	impB/mucB/samB family	5	6	4	2
PF00821	PEPCK_GTP	Phosphoenolpyruvate carboxykinase	2	4	7	5
PF00822	PMP22_Claudin	PMP-22/EMP/MP20/Claudin family	17	26	44	2
PF00827	Ribosomal_L15e	Ribosomal L15	1	1	1	0
PF00828	Ribosomal_L27A	Ribosomal protein L18e/L15	2	4	3	1
PF00829	Ribosomal_L21p	Ribosomal prokaryotic L21 protein	1	2	1	0
PF00831	Ribosomal_L29	Ribosomal L29 protein	1	2	1	0
PF00832	Ribosomal_L39	Ribosomal L39 protein	1	0	0	0
PF00833	Ribosomal_S17e	Ribosomal S17	1	2	1	0
PF00834	Ribul_P_3_epim	Ribulose-phosphate 3 epimerase family	1	2	1	1
PF00835	SNAP-25	SNAP-25 family	1	1	7	0
PF00836	Stathmin	Stathmin family	11	7	12	2
PF00837	NA	Iodothyronine deiodinase	3	0	1	1
PF00838	TCTP	Translationally controlled tumour protein	1	1	2	0
PF00839	NA	Cysteine rich repeat	1	3	2	4
PF00842	Ala_racemase_C	Alanine racemase, C-terminal domain	1	0	1	0
PF00849	PseudoU_synth_2	RNA pseudouridylate synthase	3	7	5	0
PF00850	Hist_deacetyl	Histone deacetylase domain	7	11	30	4
PF00852	Glyco_transf_10	Glycosyltransferase family 10 (fucosyltransferase) C-term	21	45	26	25
PF00853	Runt	Runt domain	1	1	3	1
PF00854	PTR2	POT family	3	14	6	5
PF00855	PWWP	PWWP domain	12	18	33	2
PF00856	SET	SET domain	31	35	73	4
PF00857	Isochorismatase	Isochorismatase family	4	7	6	0
PF00858	ASC	Amiloride-sensitive sodium channel	30	35	28	22
PF00859	NA	CTF/NF-I family transcription modulation region	0	0	4	0
PF00860	NA	Permease family	13	10	33	10
PF00861	Ribosomal_L18p	Ribosomal L18p/L5e family	2	1	4	0
PF00864	P2X_receptor	ATP P2X receptor	1	4	2	0
PF00867	XPG_I	XPG I-region	5	7	8	1
PF00868	Transglut_N	Transglutaminase family	4	10	10	5
PF00870	P53	P53 DNA-binding domain	1	4	5	0
PF00875	DNA_photolyase	DNA photolyase	5	10	9	1
PF00876	Innexin	Innexin	12	38	55	30
PF00878	CIMR	Cation-independent mannose-6-phosphate receptor repeat	0	1	2	3
PF00880	NA	Nebulin repeat	1	6	15	0
PF00882	Zn_dep_PLPC	Zinc dependent phospholipase C	0	1	3	0
PF00883	Peptidase_M17	Cytosol aminopeptidase family, catalytic domain	3	6	5	1
PF00884	Sulfatase	Sulfatase	26	19	26	46
PF00886	Ribosomal_S16	Ribosomal protein S16	1	3	2	0
PF00887	ACBP	Acyl CoA binding protein	5	4	9	0
PF00888	Cullin	Cullin family	7	7	12	6
PF00889	EF_TS	Elongation factor TS	1	3	1	0
PF00890	FAD_binding_2	FAD binding domain	3	6	1	3
PF00891	Methyltransf_2	O-methyltransferase	2	0	1	0
PF00892	NA	EamA-like transporter family	1	1	4	0
PF00899	ThiF	ThiF family	9	10	18	1
PF00900	Ribosomal_S4e	Ribosomal family S4e	1	1	1	1
PF00902	TatC	Sec-independent protein translocase protein (TatC)	0	1	0	0
PF00903	Glyoxalase	Glyoxalase/Bleomycin resistance protein/Dioxygenase superfamily	4	5	3	0
PF00905	Transpeptidase	Penicillin binding protein transpeptidase domain	0	2	1	0

PF00907	T-box	T-box	11	20	20	16
PF00909	Ammonium_transp	Ammonium Transporter Family	8	14	13	10
PF00910	RNA_helicase	RNA helicase	0	0	0	1
PF00916	Sulfate_transp	Sulfate permease family	12	25	17	22
PF00917	MATH	MATH domain	6	5	9	1
PF00919	NA	Uncharacterized protein family UPF0004	2	2	3	0
PF00923	TAL_FSA	Transaldolase	1	1	3	1
PF00926	DHBP_synthase	3,4-dihydroxy-2-butanone 4-phosphate synthase	0	0	1	0
PF00927	Transglut_C	Transglutaminase family, C-terminal ig like domain	4	9	6	2
PF00928	Adap_comp_sub	Adaptor complexes medium subunit family	6	10	13	2
PF00929	RNase_T	Exonuclease	10	21	41	1
PF00930	DPPIV_N	Dipeptidyl peptidase IV (DPP IV) N-terminal region	2	3	21	0
PF00931	NB-ARC	NB-ARC domain	0	0	7	0
PF00932	LTD	Lamin Tail Domain	1	6	6	0
PF00933	Glyco_hydro_3	Glycosyl hydrolase family 3 N terminal domain	20	8	2	5
PF00935	Ribosomal_L44	Ribosomal protein L44	1	3	1	0
PF00939	Na_sulph_symp	Sodium:sulfate symporter transmembrane region	5	12	21	4
PF00940	RNA_pol	DNA-dependent RNA polymerase	1	1	1	0
PF00941	FAD_binding_5	FAD binding domain in molybdopterin dehydrogenase	6	14	4	0
PF00953	Glycos_transf_4	Glycosyl transferase family 4	1	2	2	1
PF00954	S_locus_glycop	S-locus glycoprotein domain	0	0	0	1
PF00955	HCO3_cotransp	HCO3- transporter family	6	10	34	12
PF00956	NAP	Nucleosome assembly protein (NAP)	3	5	5	0
PF00957	Synaptobrevin	Synaptobrevin	7	13	30	0
PF00958	GMP_synt_C	GMP synthase C terminal domain	1	2	1	0
PF00962	A_deaminase	Adenosine/AMP deaminase	6	17	21	1
PF00965	TIMP	Tissue inhibitor of metalloproteinase	0	0	38	0
PF00970	FAD_binding_6	Oxidoreductase FAD-binding domain	3	5	10	0
PF00975	Thioesterase	Thioesterase domain	2	4	6	0
PF00984	UDPG_MGDP_dh	UDP-glucose/GDP-mannose dehydrogenase family, central domain	1	2	3	0
PF00986	DNA_gyraseB_C	DNA gyrase B subunit, carboxyl terminus	0	0	1	0
PF00988	CPSase_sm_chain	Carbamoyl-phosphate synthase small chain, CPSase domain	2	2	4	0
PF00989	PAS	PAS fold	8	13	24	1
PF00992	Troponin	Troponin	2	15	19	1
PF00994	MoCF_biosynth	Probable molybdopterin binding domain	4	6	3	0
PF00995	Sec1	Sec1 family	6	9	9	4
PF00996	GDI	GDP dissociation inhibitor	3	5	3	1
PF00999	Na_H_Exchanger	Sodium/hydrogen exchanger family	11	22	43	5
PF01000	RNA_pol_A_bac	RNA polymerase Rpb3/RpoA insert domain	2	3	5	0
PF01007	IRK	Inward rectifier potassium channel	5	17	15	9
PF01008	IF-2B	Initiation factor 2 subunit family	4	5	6	2
PF01011	PQQ	PQQ enzyme repeat	1	0	0	0
PF01012	ETF	Electron transfer flavoprotein domain	2	3	2	1
PF01014	Uricase	Uricase	0	0	1	0
PF01015	Ribosomal_S3Ae	Ribosomal S3Ae family	1	2	3	1
PF01016	Ribosomal_L27	Ribosomal L27 protein	1	2	1	1
PF01017	STAT_alpha	STAT protein, all-alpha domain	1	5	0	0
PF01018	GTP1_OBG	GTP1/OBG	2	3	2	0
PF01019	G_glu_transpept	Gamma-glutamyltranspeptidase	7	7	14	5
PF01020	Ribosomal_L40e	Ribosomal L40e family	0	1	1	0
PF01025	NA	GrpE	1	1	2	1
PF01026	TatD_DNase	TatD related DNase	3	4	13	2
PF01027	Bax1-I	Inhibitor of apoptosis-promoting Bax1	5	10	10	0
PF01028	Topoisom_I	Eukaryotic DNA topoisomerase I, catalytic core	1	1	3	1
PF01030	Recep_L_domain	Receptor L domain	9	5	11	4
PF01031	Dynamin_M	Dynamin central region	2	4	5	1
PF01033	Somatomedin_B	Somatomedin B domain	14	2	2	3
PF01034	NA	Syndecan domain	2	1	8	1
PF01035	DNA_binding_1	6-O-methylguanine DNA methyltransferase, DNA binding domain	1	9	2	1
PF01039	Carboxyl_trans	Carboxyl transferase domain	5	5	13	3
PF01040	UbiA	UbiA prenyltransferase family	3	6	5	2
PF01041	DegT_DnrJ_EryC1	DegT/DnrJ/EryC1/StrS aminotransferase family	1	2	3	1
PF01042	Ribonuc_L-PSP	Endoribonuclease L-PSP	2	4	2	0
PF01043	SecA_PP_bind	SecA preprotein cross-linking domain	0	1	0	0
PF01044	Vinculin	Vinculin family	3	11	19	6
PF01048	PNP_UDP_1	Phosphorylase superfamily	7	10	11	2
PF01049	NA	Cadherin cytoplasmic region	1	2	4	2
PF01053	Cys_Met_Meta_PP	Cys/Met metabolism PLP-dependent enzyme	2	3	5	3
PF01055	Glyco_hydro_31	Glycosyl hydrolases family 31	19	15	14	16
PF01056	Myc_N	Myc amino-terminal region	0	0	1	0
PF01058	Oxidored_q6	NADH ubiquinone oxidoreductase, 20 Kd subunit	1	0	1	1
PF01059	Oxidored_q5_N	NADH-ubiquinone oxidoreductase chain 4, amino terminus	0	0	1	0
PF01061	ABC2_membrane	ABC-2 type transporter	10	21	25	1
PF01062	Bestrophin	Bestrophin, RFP-TM, chloride channel	1	5	7	0
PF01063	Aminotran_4	Amino-transferase class IV	1	6	1	0
PF01064	Activin_recp	Activin types I and II receptor domain	4	4	6	0
PF01066	CDP-OH_P_transf	CDP-alcohol phosphatidyltransferase	5	5	6	0

PF01067	Calpain_III	Calpain large subunit, domain III	8	29	33	6
PF01068	DNA_ligase_A_M	ATP dependent DNA ligase domain	4	7	11	1
PF01070	FMN_dh	FMN-dependent dehydrogenase	1	0	0	0
PF01071	GARS_A	Phosphoribosylglycinamide synthetase, ATP-grasp (A) domain	1	2	2	0
PF01073	3Beta_HSD	3-beta hydroxysteroid dehydrogenase/isomerase family	4	13	17	6
PF01074	Glyco_hydro_38N	Glycosyl hydrolases family 38 N-terminal domain	6	13	18	7
PF01077	NIR_SIR	Nitrite and sulphite reductase 4Fe-4S domain	0	0	1	0
PF01079	Hint	Hint module	7	4	6	1
PF01080	Presenilin	Presenilin	1	3	3	1
PF01082	Cu2_monooxygen	Copper type II ascorbate-dependent monooxygenase, N-terminal domain	15	28	21	0
PF01084	Ribosomal_S18	Ribosomal protein S18	2	4	4	0
PF01085	HH_signal	Hedgehog amino-terminal signalling domain	1	2	8	3
PF01086	Clathrin_lg_ch	Clathrin light chain	1	5	2	0
PF01087	GalP_UDP_transf	Galactose-1-phosphate uridyl transferase, N-terminal domain	1	1	3	0
PF01088	Peptidase_C12	Ubiquitin carboxyl-terminal hydrolase, family 1	3	5	6	0
PF01090	Ribosomal_S19e	Ribosomal protein S19e	1	2	2	1
PF01091	NA	PTN/MK heparin-binding protein family, C-terminal domain	0	4	1	0
PF01092	Ribosomal_S6e	Ribosomal protein S6e	1	1	1	0
PF01094	ANF_receptor	Receptor family ligand binding region	55	89	76	55
PF01096	TFIIS_C	Transcription factor S-II (TFIIS)	3	4	4	0
PF01103	Bac_surface_Ag	Surface antigen	1	1	1	0
PF01105	EMP24_GP25L	emp24/gp25L/p24 family/GOLD	8	9	11	4
PF01106	NifU	NifU-like domain	1	1	2	0
PF01111	CKS	Cyclin-dependent kinase regulatory subunit	1	2	3	0
PF01112	Asparaginase_2	Asparaginase	3	4	7	1
PF01115	F_actin_cap_B	F-actin capping protein, beta subunit	1	2	0	1
PF01116	F_bP_aldolase	Fructose-bisphosphate aldolase class-II	0	1	0	0
PF01117	Aerolysin	Aerolysin toxin	0	25	0	0
PF01118	Semialdhyde_dh	Semialdehyde dehydrogenase, NAD binding domain	0	1	0	0
PF01119	DNA_mis_repair	DNA mismatch repair protein, C-terminal domain	5	5	6	1
PF01120	Alpha_L_fucos	Alpha-L-fucosidase	15	12	11	9
PF01121	CoaE	Dephospho-CoA kinase	2	4	2	2
PF01122	Cobalamin_bind	Eukaryotic cobalamin-binding protein	0	0	4	0
PF01124	MAPEG	MAPEG family	8	5	9	1
PF01125	G10	G10 protein	1	1	1	0
PF01126	Heme_oxygenase	Heme oxygenase	2	0	0	0
PF01127	Sdh_cyt	Succinate dehydrogenase/Fumarate reductase transmembrane subunit	1	2	2	0
PF01128	IspD	2-C-methyl-D-erythritol 4-phosphate cytidylyltransferase	1	3	1	0
PF01129	ART	NAD:arginine ADP-ribosyltransferase	0	0	1	0
PF01130	CD36	CD36 family	2	11	9	0
PF01131	Topoisom_bac	DNA topoisomerase	2	3	5	0
PF01133	NA	Enhancer of rudimentary	1	1	1	0
PF01134	GIDA	Glucose inhibited division protein A	1	4	1	0
PF01135	PCMT	Protein-L-isoaspartate(D-aspartate) O-methyltransferase (PCMT)	2	6	4	3
PF01137	RTC	RNA 3'-terminal phosphate cyclase	2	4	2	0
PF01138	RNase_PH	3' exoribonuclease family, domain 1	6	8	7	2
PF01139	RtcB	tRNA-splicing ligase RtcB	1	1	4	3
PF01142	TruD	tRNA pseudouridine synthase D (TruD)	1	2	2	2
PF01144	CoA_trans	Coenzyme A transferase	1	2	1	0
PF01145	Band_7	SPFH domain / Band 7 family	11	14	50	4
PF01146	NA	Caveolin	7	7	18	9
PF01148	CTP_transf_1	Cytidylyltransferase family	1	6	2	1
PF01149	Fapy_DNA_glyco	Formamidopyrimidine-DNA glycosylase N-terminal domain	1	3	1	0
PF01150	GDA1_CD39	GDA1/CD39 (nucleoside phosphatase) family	3	10	14	5
PF01151	NA	GNS1/SUR4 family	12	18	20	7
PF01153	Glypican	Glypican	4	2	4	5
PF01154	HMG_CoA_synt_N	Hydroxymethylglutaryl-coenzyme A synthase N terminal	1	1	1	1
PF01156	IU_nuc_hydro	Inosine-uridine preferring nucleoside hydrolase	3	3	7	1
PF01157	Ribosomal_L21e	Ribosomal protein L21e	1	1	4	0
PF01158	Ribosomal_L36e	Ribosomal protein L36e	1	1	1	0
PF01159	Ribosomal_L6e	Ribosomal protein L6e	1	1	2	2
PF01161	PBP	Phosphatidylethanolamine-binding protein	3	4	4	1
PF01163	RIO1	RIO1 family	3	5	4	1
PF01165	Ribosomal_S21	Ribosomal protein S21	0	0	1	0
PF01166	TSC22	TSC-22/dip/bun family	1	2	1	1
PF01167	Tub	Tub family	2	4	5	1
PF01168	Ala_racemase_N	Alanine racemase, N-terminal domain	3	6	2	0
PF01169	NA	Uncharacterized protein family UPF0016	1	1	1	1
PF01170	UPF0020	Putative RNA methylase family UPF0020	3	5	3	0
PF01171	ATP_bind_3	PP-loop family	2	1	3	0
PF01172	SBDS	Shwachman-Bodian-Diamond syndrome (SBDS) protein	1	1	1	0
PF01174	SNO	SNO glutamine amidotransferase family	1	0	4	1
PF01175	Urocanase	Urocanase	1	1	1	0
PF01176	eIF-1a	Translation initiation factor 1A / IF-1	2	5	2	0
PF01177	Asp_Glu_race	Asp/Glu/Hydantoin racemase	0	0	1	0
PF01179	Cu_amine_oxid	Copper amine oxidase, enzyme domain	9	23	18	8
PF01180	DHO_dh	Dihydroorotate dehydrogenase	2	4	2	0

PF01182	Glucosamine_iso	Glucosamine-6-phosphate isomerases/6-phosphogluconolactonase	3	8	4	2
PF01184	Gpr1_Fun34_YaaH	GPR1/FUN34/yaaH family	0	1	0	1
PF01186	Lysyl_oxidase	Lysyl oxidase	1	0	1	2
PF01187	MIF	Macrophage migration inhibitory factor (MIF)	4	8	2	1
PF01189	Methyltr_RsmB-F	16S rRNA methyltransferase RsmF	6	6	14	0
PF01191	RNA_pol_Rpb5_C	RNA polymerase Rpb5, C-terminal domain	1	4	1	0
PF01192	RNA_pol_Rpb6	RNA polymerase Rpb6	2	3	1	0
PF01193	RNA_pol_L	RNA polymerase Rpb3/Rpb11 dimerisation domain	2	3	5	1
PF01194	RNA_pol_N	RNA polymerases N / 8 kDa subunit	1	0	0	0
PF01195	Pept_tRNA_hydro	Peptidyl-tRNA hydrolase	1	2	1	0
PF01196	Ribosomal_L17	Ribosomal protein L17	1	2	1	0
PF01198	Ribosomal_L31e	Ribosomal protein L31e	1	2	1	0
PF01199	Ribosomal_L34e	Ribosomal protein L34e	1	2	1	0
PF01200	Ribosomal_S28e	Ribosomal protein S28e	1	0	1	0
PF01201	Ribosomal_S8e	Ribosomal protein S8e	2	3	2	0
PF01202	SKI	Shikimate kinase	2	9	5	1
PF01204	Trehalase	Trehalase	0	0	1	0
PF01205	NA	Uncharacterized protein family UPF0029	1	1	17	0
PF01207	Dus	Dihydrouridine synthase (Dus)	4	12	5	2
PF01208	URO-D	Uroporphyrinogen decarboxylase (URO-D)	1	1	1	0
PF01209	Ubie_methyltran	ubiE/COQ5 methyltransferase family	1	2	1	0
PF01210	NAD_Gly3P_dh_N	NAD-dependent glycerol-3-phosphate dehydrogenase N-terminus	2	4	2	1
PF01212	Beta_elim_lyase	Beta-eliminating lyase	1	2	3	1
PF01213	CAP_N	Adenylate cyclase associated (CAP) N terminal	1	1	3	0
PF01214	CK_II_beta	Casein kinase II regulatory subunit	1	2	3	0
PF01215	COX5B	Cytochrome c oxidase subunit Vb	1	1	4	0
PF01217	Clat_adaptor_s	Clathrin adaptor complex small chain	7	18	17	0
PF01218	Coprogen_oxidas	Coproporphyrinogen III oxidase	1	1	1	1
PF01221	Dynein_light	Dynein light chain type 1	8	2	2	2
PF01222	NA	Ergosterol biosynthesis ERG4/ERG24 family	2	19	4	2
PF01223	Endonuclease_NS	DNA/RNA non-specific endonuclease	1	3	3	0
PF01225	Mur_ligase	Mur ligase family, catalytic domain	0	1	0	0
PF01227	GTP_cyclohydroI	GTP cyclohydrolase I	1	3	1	1
PF01228	Gly_radical	Glycine radical	0	1	0	0
PF01229	Glyco_hydro_39	Glycosyl hydrolases family 39	1	3	0	0
PF01230	HIT	HIT domain	3	10	3	0
PF01231	IDO	Indoleamine 2,3-dioxygenase	4	9	1	0
PF01233	NMT	Myristoyl-CoA:protein N-myristoyltransferase, N-terminal domain	1	2	1	0
PF01234	NNMT_PNMT_TEMT	NNMT/PNMT/TEMT family	1	0	0	0
PF01237	Oxysterol_BP	Oxysterol-binding protein	6	16	22	3
PF01238	PMI_typeI	Phosphomannose isomerase type I	1	1	1	0
PF01239	PPTA	Protein prenyltransferase alpha subunit repeat	3	3	3	0
PF01242	PTPS	6-pyruvoyl tetrahydropterin synthase	1	7	1	0
PF01243	Putative_PNPOx	Pyridoxamine 5'-phosphate oxidase	1	2	1	0
PF01244	Peptidase_M19	Membrane dipeptidase (Peptidase family M19)	2	8	3	1
PF01245	Ribosomal_L19	Ribosomal protein L19	1	1	0	0
PF01246	Ribosomal_L24e	Ribosomal protein L24e	2	3	2	1
PF01247	Ribosomal_L35Ae	Ribosomal protein L35Ae	2	1	1	0
PF01248	Ribosomal_L7Ae	Ribosomal protein L7Ae/L30e/S12e/Gadd45 family	9	9	13	1
PF01249	Ribosomal_S21e	Ribosomal protein S21e	1	2	3	0
PF01250	Ribosomal_S6	Ribosomal protein S6	1	1	1	0
PF01251	Ribosomal_S7e	Ribosomal protein S7e	1	2	1	0
PF01253	SUI1	Translation initiation factor SUI1	4	6	3	0
PF01255	Prenyltransf	Putative undecaprenyl diphosphate synthase	2	5	3	0
PF01256	Carb_kinase	Carbohydrate kinase	1	3	1	0
PF01257	2Fe-2S_thioredx	Thioredoxin-like [2Fe-2S] ferredoxin	1	1	1	0
PF01258	zf-dskA_traR	Prokaryotic dksA/traR C4-type zinc finger	0	1	1	0
PF01259	SAICAR_synt	SAICAR synthetase	1	1	1	1
PF01261	AP_endonuc_2	Xylose isomerase-like TIM barrel	2	3	5	0
PF01262	AlaDh_PNT_C	Alanine dehydrogenase/PNT, C-terminal domain	2	2	1	1
PF01263	Aldose_epim	Aldose 1-epimerase	3	6	17	2
PF01264	Chorismate_synt	Chorismate synthase	0	0	1	0
PF01265	NA	Cytochrome c/c1 heme lyase	1	1	2	1
PF01266	DAO	FAD dependent oxidoreductase	13	18	26	2
PF01267	F-actin_cap_A	F-actin capping protein alpha subunit	1	1	2	1
PF01268	FTHFS	Formate--tetrahydrofolate ligase	1	1	1	2
PF01269	Fibrillarin	Fibrillarin	1	2	3	0
PF01272	GreA_GreB	Transcription elongation factor, GreA/GreB, C-term	0	2	0	0
PF01273	LBP_BPI_CETP	LBP / BPI / CETP family, N-terminal domain	1	7	4	0
PF01274	Malate_synthase	Malate synthase	0	0	5	0
PF01275	NA	Myelin proteolipid protein (PLP or lipophilin)	2	1	15	0
PF01280	Ribosomal_L19e	Ribosomal protein L19e	1	4	1	0
PF01281	Ribosomal_L9_N	Ribosomal protein L9, N-terminal domain	1	0	1	0
PF01282	Ribosomal_S24e	Ribosomal protein S24e	1	1	3	0
PF01283	Ribosomal_S26e	Ribosomal protein S26e	1	2	1	1
PF01284	NA	Membrane-associating domain	5	7	4	0
PF01285	TEA	TEA/ATTS domain family	1	2	7	1

PF01286	XPA_N	XPA protein N-terminal	1	1	2	0
PF01287	eIF-5a	Eukaryotic elongation factor 5A hypusine, DNA-binding OB fold	2	1	3	0
PF01290	Thymosin	Thymosin beta-4 family	2	4	9	0
PF01294	Ribosomal_L13e	Ribosomal protein L13e	1	1	1	2
PF01299	Lamp	Lysosome-associated membrane glycoprotein (Lamp)	1	2	6	0
PF01300	Sua5_yciO_yrdC	Telomere recombination	3	5	3	4
PF01301	Glyco_hydro_35	Glycosyl hydrolases family 35	2	2	11	1
PF01302	CAP_GLY	CAP-Gly domain	11	23	62	2
PF01315	Ald_Xan_dh_C	Aldehyde oxidase and xanthine dehydrogenase, a/b hammerhead domain	6	15	5	1
PF01321	Creatinase_N	Creatinase/Prolidase N-terminal domain	2	11	5	0
PF01323	DSBA	DSBA-like thioredoxin domain	2	2	1	0
PF01326	PPDK_N	Pyruvate phosphate dikinase, PEP/pyruvate binding domain	0	2	3	0
PF01327	Pep_deformylase	Polypeptide deformylase	0	1	0	0
PF01329	Pterin_4a	Pterin 4 alpha carbinolamine dehydratase	1	1	1	0
PF01331	mRNA_cap_enzyme	mRNA capping enzyme, catalytic domain	3	1	4	0
PF01335	DED	Death effector domain	5	6	17	1
PF01336	tRNA_anti-codon	OB-fold nucleic acid binding domain	5	10	11	0
PF01342	SAND	SAND domain	3	3	4	2
PF01343	Peptidase_S49	Peptidase family S49	0	1	1	0
PF01344	Kelch_1	Kelch motif	42	70	114	49
PF01347	NA	Lipoprotein amino terminal region	6	9	10	8
PF01351	RNase_HII	Ribonuclease HII	1	2	1	0
PF01357	Pollen_allerg_1	Pollen allergen	0	1	0	1
PF01359	NA	Transposase (partial DDE domain)	0	0	3	0
PF01363	FYVE	FYVE zinc finger	20	50	45	3
PF01365	RYDR_ITPR	RIH domain	5	15	19	2
PF01367	5_3_exonuc	5'-3' exonuclease, C-terminal SAM fold	0	1	1	0
PF01368	DHH	DHH family	1	0	1	0
PF01369	Sec7	Sec7 domain	4	13	21	2
PF01370	Epimerase	NAD dependent epimerase/dehydratase family	8	11	14	5
PF01379	Porphobil_deam	Porphobilinogen deaminase, dipyromethane cofactor binding domain	1	2	3	0
PF01380	SIS	SIS domain	1	6	7	1
PF01381	HTH_3	Helix-turn-helix	1	0	1	0
PF01384	NA	Phosphate transporter family	1	8	7	0
PF01388	ARID	ARID/BRIGHT DNA binding domain	6	22	20	0
PF01390	SEA	SEA domain	7	21	61	0
PF01391	Collagen	Collagen triple helix repeat (20 copies)	27	35	54	8
PF01392	Fz	Fz domain	10	23	35	10
PF01393	Chromo_shadow	Chromo shadow domain	1	2	3	0
PF01394	Clathrin_propel	Clathrin propeller repeat	1	1	4	0
PF01396	zf-C4_Topoisom	Topoisomerase DNA binding C4 zinc finger	0	2	4	0
PF01398	JAB	JAB1/Mov34/MPN/PAD-1 ubiquitin protease	11	13	14	1
PF01399	PCI	PCI domain	15	19	24	2
PF01400	Astacin	Astacin (Peptidase family M12A)	20	41	70	11
PF01401	Peptidase_M2	Angiotensin-converting enzyme	5	12	8	4
PF01403	Sema	Sema domain	7	14	33	4
PF01404	Ephrin_lbd	Ephrin receptor ligand binding domain	1	1	6	1
PF01406	tRNA-synt_1e	tRNA synthetases class I (C) catalytic domain	2	6	4	1
PF01408	GFO_IDH_MocA	Oxidoreductase family, NAD-binding Rossmann fold	6	5	13	0
PF01409	tRNA-synt_2d	tRNA synthetases class II core domain (F)	1	2	7	1
PF01410	COLFI	Fibrillar collagen C-terminal domain	3	3	3	2
PF01411	tRNA-synt_2c	tRNA synthetases class II (A)	5	9	5	0
PF01412	ArfGap	Putative GTPase activating protein for Arf	10	27	59	0
PF01413	C4	C-terminal tandem repeated domain in type 4 procollagen	4	3	4	2
PF01414	DSL	Delta serrate ligand	5	18	27	4
PF01416	PseudoU_synth_1	tRNA pseudouridine synthase	3	7	5	0
PF01417	ENTH	ENTH domain	2	17	12	1
PF01421	Reprolysin	Reprolysin (M12B) family zinc metalloprotease	12	17	35	0
PF01422	NA	NF-X1 type zinc finger	2	2	2	0
PF01423	LSM	LSM domain	17	15	23	2
PF01424	R3H	R3H domain	8	8	10	2
PF01425	Amidase	Amidase	4	19	12	9
PF01426	BAH	BAH domain	6	19	32	3
PF01428	zf-AN1	AN1-like Zinc finger	4	12	10	1
PF01429	MBD	Methyl-CpG binding domain	4	8	22	0
PF01431	Peptidase_M13	Peptidase family M13	15	22	25	2
PF01432	Peptidase_M3	Peptidase family M3	3	5	4	3
PF01433	Peptidase_M1	Peptidase family M1	12	31	25	1
PF01434	Peptidase_M41	Peptidase family M41	3	7	8	2
PF01435	Peptidase_M48	Peptidase family M48	3	4	4	1
PF01436	NHL	NHL repeat	14	32	97	13
PF01437	PSI	Plexin repeat	6	15	24	2
PF01442	Apolipoprotein	Apolipoprotein A1/A4/E domain	1	1	0	0
PF01447	Peptidase_M4	Thermolysin metallopeptidase, catalytic domain	1	1	1	4
PF01448	ELM2	ELM2 domain	3	4	13	0
PF01451	LMWPc	Low molecular weight phosphotyrosine protein phosphatase	1	0	2	0
PF01454	MAGE	MAGE family	1	2	1	0

PF01457	NA	Leishmanolysin	2	1	2	0
PF01458	UPF0051	Uncharacterized protein family (UPF0051)	0	1	0	0
PF01459	Porin_3	Eukaryotic porin	2	5	4	0
PF01462	LRRNT	Leucine rich repeat N-terminal domain	4	1	4	3
PF01463	LRRCT	Leucine rich repeat C-terminal domain	1	1	0	4
PF01464	SLT	Transglycosylase SLT domain	0	1	0	0
PF01465	GRIP	GRIP domain	3	9	5	0
PF01466	Skp1	Skp1 family, dimerisation domain	1	1	2	0
PF01467	CTP_transf_like	Cytidylyltransferase-like	4	9	12	0
PF01469	NA	Pentapeptide repeats (8 copies)	1	2	0	1
PF01470	Peptidase_C15	Pyroglutamyl peptidase	1	1	1	0
PF01471	PG_binding_1	Putative peptidoglycan binding domain	5	2	6	1
PF01472	PUA	PUA domain	2	5	2	0
PF01476	LysM	LysM domain	1	6	8	0
PF01477	PLAT	PLAT/LH2 domain	29	48	68	19
PF01479	S4	S4 domain	3	3	2	0
PF01480	PWI	PWI domain	3	7	12	1
PF01483	P_proprotein	Proprotein convertase P-domain	6	10	27	3
PF01484	NA	Nematode cuticle collagen N-terminal domain	0	2	0	0
PF01485	IBR	IBR domain, a half RING-finger domain	24	19	23	8
PF01488	Shikimate_DH	Shikimate / quinate 5-dehydrogenase	0	0	1	0
PF01490	NA	Transmembrane amino acid transporter protein	8	24	27	7
PF01491	Frataxin_Cyay	Frataxin-like domain	1	1	2	1
PF01493	GXGXG	GXGXG motif	1	2	4	0
PF01494	FAD_binding_3	FAD binding domain	2	4	4	0
PF01496	V_ATPase_I	V-type ATPase 116kDa subunit family	2	7	14	1
PF01498	HTH_Tnp_Tc3_2	Transposase	0	0	1	1
PF01500	NA	Keratin, high sulfur B2 protein	0	1	0	0
PF01501	Glyco_transf_8	Glycosyl transferase family 8	3	11	6	1
PF01504	PIP5K	Phosphatidylinositol-4-phosphate 5-Kinase	4	17	10	1
PF01505	Vault	Major Vault Protein repeat	2	5	3	0
PF01507	PAPS_reduct	Phosphoadenosine phosphosulfate reductase family	1	3	1	0
PF01509	TruB_N	TruB family pseudouridylate synthase (N terminal domain)	2	2	3	0
PF01510	Amidase_2	N-acetylmuramoyl-L-alanine amidase	6	9	14	2
PF01512	Complex1_51K	Respiratory-chain NADH dehydrogenase 51 Kd subunit	1	2	1	0
PF01513	NAD_kinase	ATP-NAD kinase	2	5	5	0
PF01515	PTA_PTB	Phosphate acetyl/butaryl transferase	0	1	0	0
PF01521	Fe-S_biosyn	Iron-sulphur cluster biosynthesis	1	2	2	0
PF01522	Polysacc_deac_1	Polysaccharide deacetylase	6	6	5	2
PF01529	NA	DHHC palmitoyltransferase	20	42	53	6
PF01530	zf-C2HC	Zinc finger, C2HC type	3	5	13	1
PF01531	NA	Glycosyl transferase family 11	13	13	38	9
PF01532	Glyco_hydro_47	Glycosyl hydrolase family 47	6	7	5	4
PF01534	Frizzled	Frizzled/Smoothened family membrane region	5	6	16	6
PF01535	PPR	PPR repeat	2	1	2	0
PF01536	SAM_decarbox	Adenosylmethionine decarboxylase	1	1	3	0
PF01541	GIY-YIG	GIY-YIG catalytic domain	1	2	2	0
PF01545	NA	Cation efflux family	11	30	13	2
PF01546	Peptidase_M20	Peptidase family M20/M25/M40	4	4	4	0
PF01549	ShK	ShK domain-like	13	36	111	7
PF01551	Peptidase_M23	Peptidase family M23	0	1	3	0
PF01553	Acyltransferase	Acyltransferase	13	22	21	1
PF01554	MatE	MatE	6	0	5	0
PF01556	DnaJ_C	DnaJ C terminal domain	6	9	7	3
PF01557	FAA_hydrolase	Fumarylacetoacetate (FAA) hydrolase family	5	2	11	4
PF01558	POR	Pyruvate ferredoxin/flavodoxin oxidoreductase	0	1	0	0
PF01562	NA	Reprolysin family propeptide	7	31	29	6
PF01564	Spermine_synth	Spermine/spermidine synthase	2	8	7	0
PF01565	FAD_binding_4	FAD binding domain	8	10	14	0
PF01566	Nramp	Natural resistance-associated macrophage protein	2	5	2	4
PF01568	Molydop_binding	Molydopterin dinucleotide binding domain	0	0	1	0
PF01569	PAP2	PAP2 superfamily	10	20	18	1
PF01571	GCV_T	Aminomethyltransferase folate-binding domain	5	8	6	1
PF01575	MaoC_dehydratas	MaoC like domain	2	1	2	0
PF01576	Myosin_tail_1	Myosin tail	3	27	20	12
PF01581	NA	FMRFamide related peptide family	1	3	1	3
PF01582	TIR	TIR domain	45	49	87	26
PF01583	APS_kinase	Adenylylsulphate kinase	1	1	5	1
PF01585	G-patch	G-patch domain	13	17	23	8
PF01586	NA	Myogenic Basic domain	1	1	1	0
PF01588	tRNA_bind	Putative tRNA binding domain	2	8	2	1
PF01590	GAF	GAF domain	9	11	39	3
PF01591	6PF2K	6-phosphofructo-2-kinase	1	3	8	0
PF01592	NifU_N	NifU-like N terminal domain	1	0	1	0
PF01593	Amino_oxidase	Flavin containing amine oxidoreductase	11	23	24	5
PF01594	NA	Domain of unknown function DUF20	1	0	1	0
PF01595	NA	Domain of unknown function DUF21	3	3	3	1

PF01596	Methyltransf_3	O-methyltransferase	2	2	6	2
PF01597	GCV_H	Glycine cleavage H-protein	2	2	2	0
PF01599	Ribosomal_S27	Ribosomal protein S27a	0	2	1	0
PF01602	Adaptin_N	Adaptin N terminal region	8	14	19	8
PF01603	B56	Protein phosphatase 2A regulatory B subunit (B56 family)	2	9	19	4
PF01607	CBM_14	Chitin binding Peritrophin-A domain	80	76	93	54
PF01608	NA	I/LWEQ domain	2	12	8	0
PF01612	DNA_pol_A_exo1	3'-5' exonuclease	6	10	27	3
PF01613	Flavin_Reduct	Flavin reductase like domain	1	1	1	0
PF01619	Pro_dh	Proline dehydrogenase	2	4	3	1
PF01624	MutS_I	MutS domain I	3	3	4	1
PF01625	PMSR	Peptide methionine sulfoxide reductase	1	1	5	1
PF01628	HrcA	HrcA protein C terminal domain	0	1	0	0
PF01630	Glyco_hydro_56	Hyaluronidase	1	1	4	5
PF01632	Ribosomal_L35p	Ribosomal protein L35	1	1	1	0
PF01633	Choline_kinase	Choline/ethanolamine kinase	2	5	2	1
PF01636	APH	Phosphotransferase enzyme family	4	7	12	0
PF01637	NA	Archaeal ATPase	2	0	2	0
PF01640	Peptidase_C10	Peptidase C10 family	1	0	0	0
PF01641	SelR	SelR domain	3	5	9	0
PF01642	MM_CoA_mutase	Methylmalonyl-CoA mutase	1	1	1	0
PF01643	Acyl-ACP_TE	Acyl-ACP thioesterase	3	9	9	2
PF01645	Glu_synthase	Conserved region in glutamate synthase	3	2	4	0
PF01648	ACPS	4'-phosphopantetheinyl transferase superfamily	1	1	0	0
PF01650	Peptidase_C13	Peptidase C13 family	2	3	3	0
PF01652	IF4E	Eukaryotic initiation factor 4E	3	4	6	0
PF01654	Cyt_bd_oxida_I	Cytochrome bd terminal oxidase subunit I	0	1	0	0
PF01655	Ribosomal_L32e	Ribosomal protein L32	0	2	2	0
PF01658	Inos-1-P_synth	Myo-inositol-1-phosphate synthase	1	1	2	3
PF01661	Macro	Macro domain	9	28	77	19
PF01663	Phosphodiest	Type I phosphodiesterase / nucleotide pyrophosphatase	8	17	18	14
PF01667	Ribosomal_S27e	Ribosomal protein S27	1	1	1	0
PF01668	SmpB	SmpB protein	0	1	0	0
PF01676	Metalloenzyme	Metalloenzyme superfamily	0	1	0	0
PF01678	DAP_epimerase	Diaminopimelate epimerase	1	5	0	1
PF01680	SOR_SNZ	SOR/SNZ family	1	0	1	0
PF01682	NA	DB module	1	0	3	0
PF01683	NA	EB module	1	27	44	29
PF01687	Flavokinase	Riboflavin kinase	1	2	1	1
PF01693	Cauli_VI	Caulimovirus viroplasmin	0	0	2	1
PF01694	Rhomboid	Rhomboid family	8	17	13	5
PF01697	NA	Glycosyltransferase family 92	14	27	18	27
PF01699	Na_Ca_ex	Sodium/calcium exchanger protein	10	19	42	6
PF01702	TGT	Queuine tRNA-ribosyltransferase	1	7	2	1
PF01704	UDPGP	UTP--glucose-1-phosphate uridylyltransferase	2	2	7	0
PF01709	Transcrip_reg	Transcriptional regulator	1	2	7	1
PF01712	dNK	Deoxynucleoside kinase	4	4	7	1
PF01713	Smr	Smr domain	0	10	1	0
PF01715	IPPT	IPP transferase	1	1	1	0
PF01722	BolA	BolA-like protein	3	4	4	0
PF01725	Ham1p_like	Ham1 family	0	2	2	0
PF01728	FtsJ	FtsJ-like methyltransferase	5	5	5	2
PF01729	QRPTase_C	Quinolinate phosphoribosyl transferase, C-terminal domain	1	2	1	0
PF01730	UreF	UreF	1	0	0	0
PF01731	Arylesterase	Arylesterase	1	5	6	1
PF01733	Nucleoside_tran	Nucleoside transporter	3	5	2	1
PF01734	Patatin	Patatin-like phospholipase	12	18	17	2
PF01735	PLA2_B	Lysophospholipase catalytic domain	1	4	16	2
PF01738	DLH	Dienelactone hydrolase family	3	5	3	0
PF01740	STAS	STAS domain	9	18	17	11
PF01743	PolyA_pol	Poly A polymerase head domain	1	2	1	1
PF01744	NA	GLTT repeat (6 copies)	0	0	0	1
PF01746	tRNA_m1G_MT	tRNA (Guanine-1)-methyltransferase	2	3	3	1
PF01747	ATP-sulfurylase	ATP-sulfurylase	1	1	5	1
PF01749	IBB	Importin beta binding domain	4	3	3	0
PF01751	Toprim	Toprim domain	3	9	7	1
PF01753	zf-MYND	MYND finger	18	39	53	6
PF01754	zf-A20	A20-like zinc finger	3	6	8	3
PF01755	NA	Glycosyltransferase family 25 (LPS biosynthesis protein)	1	1	2	0
PF01756	ACOX	Acyl-CoA oxidase	4	9	9	4
PF01757	NA	Acyltransferase family	6	12	11	0
PF01758	SBF	Sodium Bile acid symporter family	2	2	19	4
PF01759	NTR	UNC-6/NTR/C345C module	4	5	13	1
PF01762	NA	Galactosyltransferase	37	90	70	53
PF01764	Lipase_3	Lipase (class 3)	1	4	7	0
PF01765	RRF	Ribosome recycling factor	1	2	1	0
PF01769	NA	Divalent cation transporter	1	1	2	0

PF01770	NA	Reduced folate carrier	1	0	0	0
PF01771	Viral_alk_exo	Herpesvirus alkaline exonuclease	1	0	10	4
PF01773	Nucleos_tra2_N	Na+ dependent nucleoside transporter N-terminus	6	11	17	1
PF01774	NA	UreD urease accessory protein	1	1	0	0
PF01775	Ribosomal_L18A	Ribosomal L18ae/LX protein domain	1	2	1	0
PF01776	Ribosomal_L22e	Ribosomal L22e protein family	1	1	1	0
PF01777	Ribosomal_L27e	Ribosomal L27e protein family	1	0	1	0
PF01778	Ribosomal_L28e	Ribosomal L28e protein family	2	2	2	1
PF01779	Ribosomal_L29e	Ribosomal L29e protein family	1	0	0	0
PF01780	Ribosomal_L37ae	Ribosomal L37ae protein family	1	1	2	0
PF01781	Ribosomal_L38e	Ribosomal L38e protein family	1	0	0	0
PF01783	Ribosomal_L32p	Ribosomal L32p protein family	1	2	2	0
PF01784	NIF3	NIF3 (NGG1p interacting factor 3)	1	2	1	2
PF01786	AOX	Alternative oxidase	1	3	2	0
PF01791	DeoC	DeoC/LacD family aldolase	1	1	1	0
PF01794	Ferric_reduct	Ferric reductase like transmembrane component	8	10	17	1
PF01795	NA	MraW methylase family	1	2	1	1
PF01798	Nop	snoRNA binding domain, fibrillarin	4	3	5	0
PF01799	Fer2_2	[2Fe-2S] binding domain	7	13	4	1
PF01803	NA	LIM-domain binding protein	1	2	2	0
PF01805	Surp	Surp module	4	4	5	1
PF01808	AICARFT_IMPCHas	AICARFT/IMPCHase bienzyme	1	2	1	0
PF01812	5-FTHF_cyc-lig	5-formyltetrahydrofolate cyclo-ligase family	1	3	3	1
PF01813	ATP-synt_D	ATP synthase subunit D	1	1	1	0
PF01814	Hemerythrin	Hemerythrin HHE cation binding domain	0	0	1	0
PF01822	WSC	WSC domain	11	6	32	6
PF01823	MACPF	MAC/Perforin domain	3	23	16	11
PF01825	NA	GPCR proteolysis site, GPS, motif	13	36	49	9
PF01826	TIL	Trypsin Inhibitor like cysteine rich domain	5	6	10	10
PF01833	TIG	IPT/TIG domain	11	21	55	2
PF01834	XRCC1_N	XRCC1 N terminal domain	4	4	3	0
PF01835	MG2	MG2 domain	13	16	15	0
PF01839	FG-GAP	FG-GAP repeat	5	10	24	2
PF01841	Transglut_core	Transglutaminase-like superfamily	9	16	55	1
PF01842	ACT	ACT domain	4	6	4	0
PF01843	DIL	DIL domain	3	2	7	1
PF01844	HNH	HNH endonuclease	1	4	1	0
PF01846	FF	FF domain	3	5	4	1
PF01847	VHL	von Hippel-Lindau disease tumour suppressor protein	1	1	1	1
PF01849	NAC	NAC domain	2	4	2	0
PF01851	PC_rep	Proteasome/cyclosome repeat	2	2	3	1
PF01852	START	START domain	7	12	14	0
PF01853	MOZ_SAS	MOZ/SAS family	4	6	5	3
PF01855	POR_N	Pyruvate flavodoxin/ferredoxin oxidoreductase, thiamine diP-bdg	0	1	0	0
PF01857	RB_B	Retinoblastoma-associated protein B domain	2	2	2	1
PF01858	RB_A	Retinoblastoma-associated protein A domain	2	2	2	0
PF01866	Diphthamide_syn	Putative diphthamide synthesis protein	2	3	4	1
PF01868	UPF0086	Domain of unknown function UPF0086	1	1	3	0
PF01869	BcrAD_BadFG	BadF/BadG/BcrA/BcrD ATPase family	1	0	1	0
PF01871	AMMECR1	AMMECR1	1	1	1	1
PF01873	eIF-5_eIF-2B	Domain found in IF2B/IF5	2	1	2	0
PF01875	NA	Memo-like protein	1	4	2	0
PF01876	RNase_P_p30	RNase P subunit p30	1	1	1	0
PF01878	EVE	EVE domain	1	1	0	0
PF01883	FeS_assembly_P	Domain of unknown function DUF59	1	2	2	0
PF01885	PTS_2-RNA	RNA 2'-phosphotransferase, Tpt1 / KptA family	2	2	13	0
PF01894	UPF0047	Uncharacterised protein family UPF0047	1	1	1	0
PF01896	DNA_primase_S	Eukaryotic and archaeal DNA primase small subunit	0	1	2	0
PF01900	RNase_P_Rpp14	Rpp14/Pop5 family	1	2	1	0
PF01902	Diphthami_syn_2	Diphthamide synthase	1	2	1	0
PF01907	Ribosomal_L37e	Ribosomal protein L37e	1	0	1	0
PF01909	NTP_transf_2	Nucleotidyltransferase domain	3	3	13	2
PF01912	eIF-6	eIF-6 family	1	1	2	0
PF01915	Glyco_hydro_3_C	Glycosyl hydrolase family 3 C-terminal domain	20	6	2	3
PF01916	DS	Deoxyhypusine synthase	1	1	1	1
PF01918	Alba	Alba	1	4	1	0
PF01920	Prefoldin_2	Prefoldin subunit	5	10	5	2
PF01922	SRP19	SRP19 protein	1	1	2	0
PF01923	Cob_adeno_trans	Cobalamin adenosyltransferase	1	1	1	0
PF01926	MMR_HSR1	50S ribosome-binding GTPase	17	32	52	1
PF01927	NA	Mut7-C RNAse domain	1	3	5	2
PF01928	CYTH	CYTH domain	2	6	10	2
PF01929	Ribosomal_L14e	Ribosomal protein L14	1	3	1	0
PF01931	NTPase_I-T	Protein of unknown function DUF84	0	4	1	0
PF01936	NYN	NYN domain	1	1	1	0
PF01937	DUF89	Protein of unknown function DUF89	2	2	2	2
PF01938	TRAM	TRAM domain	2	2	4	0

PF01940	NA	Integral membrane protein DUF92	1	1	1	1
PF01946	Thi4	Thi4 family	0	1	0	0
PF01951	Archease	Archease protein family (MTH1598/TM1083)	1	3	2	0
PF01956	EMC3_TMCO1	Integral membrane protein DUF106	2	2	2	0
PF01958	DUF108	Domain of unknown function DUF108	1	1	1	0
PF01963	NA	TraB family	2	4	3	2
PF01964	ThiC_Rad_SAM	Radical SAM ThiC family	0	0	1	0
PF01965	DJ-1_PfpI	DJ-1/PfpI family	3	6	8	0
PF01966	HD	HD domain	3	10	13	2
PF01967	MoaC	MoaC family	1	1	1	1
PF01968	Hydantoinase_A	Hydantoinase/oxoprolinase	2	6	3	0
PF01974	tRNA_int_endo	tRNA intron endonuclease, catalytic C-terminal domain	2	2	2	0
PF01979	Amidohydro_1	Amidohydrolase family	7	12	20	3
PF01980	NA	Uncharacterised protein family UPF0066	1	2	3	0
PF01981	NA	Peptidyl-tRNA hydrolase PTH2	2	7	5	2
PF01984	dsDNA_bind	Double-stranded DNA-binding domain	0	1	1	0
PF01990	ATP-synt_F	ATP synthase (F/14-kDa) subunit	1	1	1	0
PF01991	vATP-synt_E	ATP synthase (E/31 kDa) subunit	1	1	1	0
PF01992	vATP-synt_AC39	ATP synthase (C/AC39) subunit	1	1	1	1
PF01997	Translin	Translin family	2	2	3	2
PF02002	TFIIE_alpha	TFIIE alpha subunit	1	5	1	0
PF02005	TRM	N2,N2-dimethylguanosine tRNA methyltransferase	2	1	3	1
PF02008	zf-CXXC	CXXC zinc finger domain	4	4	7	1
PF02010	NA	REJ domain	8	15	23	12
PF02014	Reeler	Reeler domain	16	53	54	3
PF02017	CIDE-N	CIDE-N domain	3	2	7	1
PF02018	CBM_4_9	Carbohydrate binding domain	1	2	5	0
PF02019	WIF	WIF domain	1	2	4	0
PF02020	W2	eIF4-gamma/eIF5/eIF2-epsilon	5	10	20	1
PF02023	NA	SCAN domain	2	10	13	10
PF02026	RyR	RyR domain	1	2	8	0
PF02033	RBFA	Ribosome-binding factor A	1	1	1	0
PF02036	SCP2	SCP-2 sterol transfer family	4	6	9	2
PF02037	SAP	SAP domain	8	14	23	2
PF02045	CBFB_NFYA	CCAAT-binding transcription factor (CBF-B/NF-YA) subunit B	2	7	8	0
PF02046	COX6A	Cytochrome c oxidase subunit VIa	0	2	0	0
PF02055	Glyco_hydro_30	O-Glycosyl hydrolase family 30	2	1	4	1
PF02057	Glyco_hydro_59	Glycosyl hydrolase family 59	6	7	1	0
PF02060	ISK_Channel	Slow voltage-gated potassium channel	1	1	0	0
PF02064	MAS20	MAS20 protein import receptor	1	1	1	0
PF02077	NA	SURF4 family	1	2	1	1
PF02078	Synapsin	Synapsin, N-terminal domain	1	0	1	0
PF02089	Palm_thioest	Palmitoyl protein thioesterase	2	5	6	0
PF02091	tRNA-synt_2e	Glycyl-tRNA synthetase alpha subunit	0	1	0	0
PF02092	NA	Glycyl-tRNA synthetase beta subunit	0	1	0	0
PF02095	NA	Extensin-like protein repeat	1	0	0	0
PF02096	60KD_IMP	60Kd inner membrane protein	2	2	3	0
PF02099	Josephin	Josephin	2	2	2	1
PF02100	ODC_AZ	Ornithine decarboxylase antizyme	1	1	1	0
PF02101	NA	Ocular albinism type 1 protein	2	0	2	0
PF02104	NA	SURF1 family	1	1	2	0
PF02106	NA	Fanconi anaemia group C protein	0	1	0	0
PF02109	DAD	DAD family	1	0	0	0
PF02114	Phosducin	Phosducin	1	3	4	0
PF02115	Rho_GDI	RHO protein GDP dissociation inhibitor	1	2	3	2
PF02121	IP_trans	Phosphatidylinositol transfer protein	3	4	13	2
PF02126	PTE	Phosphotriesterase family	2	1	1	1
PF02127	Peptidase_M18	Aminopeptidase I zinc metalloprotease (M18)	1	1	1	1
PF02130	NA	Uncharacterized protein family UPF0054	0	1	1	0
PF02135	zf-TAZ	TAZ zinc finger	1	1	9	1
PF02136	NTF2	Nuclear transport factor 2 (NTF2) domain	4	3	6	0
PF02137	A_deamin	Adenosine-deaminase (editase) domain	5	14	14	9
PF02138	Beach	Beige/BEACH domain	8	10	23	4
PF02140	Gal_Lectin	Galactose binding lectin domain	2	4	53	3
PF02141	DENN	DENN (AEX-3) domain	7	34	36	2
PF02142	MGS	MGS-like domain	3	3	6	1
PF02144	Rad1	Repair protein Rad1/Rec1/Rad17	2	3	1	1
PF02145	NA	Rap/ran-GAP	5	19	39	1
PF02146	SIR2	Sir2 family	8	12	15	4
PF02148	zf-UBP	Zn-finger in ubiquitin-hydrolases and other protein	10	9	15	1
PF02149	NA	Kinase associated domain 1	2	4	12	2
PF02150	RNA_POL_M_15KD	RNA polymerases M/15 Kd subunit	2	2	2	0
PF02151	UVR	UvrB/uvrC motif	1	3	0	1
PF02156	Glyco_hydro_26	Glycosyl hydrolase family 26	4	0	0	1
PF02157	Man-6-P_recep	Mannose-6-phosphate receptor	2	6	5	0
PF02158	NA	Neuregulin family	0	2	0	0
PF02163	Peptidase_M50	Peptidase family M50	1	3	1	0

PF02167	Cytochrom_C1	Cytochrome C1 family	1	1	1	1
PF02170	PAZ	PAZ domain	4	9	13	1
PF02171	Piwi	Piwi domain	4	8	13	3
PF02172	KIX	KIX domain	1	1	9	0
PF02173	NA	pKID domain	0	1	3	0
PF02174	IRS	PTB domain (IRS-1 type)	6	16	18	2
PF02176	zf-TRAF	TRAF-type zinc finger	3	16	38	1
PF02177	NA	Amyloid A4 N-terminal heparin-binding	1	1	0	0
PF02178	NA	AT hook motif	1	1	1	0
PF02179	BAG	BAG domain	1	1	5	1
PF02180	BH4	Bcl-2 homology region 4	1	0	1	0
PF02181	FH2	Formin Homology 2 Domain	10	33	53	4
PF02182	SAD_SRA	SAD/SRA domain	2	2	3	0
PF02184	HAT	HAT (Half-A-TPR) repeat	1	1	1	0
PF02185	HR1	Hr1 repeat	2	4	3	2
PF02186	TFIIE_beta	TFIIE beta subunit core domain	1	1	2	0
PF02187	GAS2	Growth-Arrest-Specific Protein 2 Domain	3	21	28	0
PF02188	GoLoco	GoLoco motif	1	6	7	2
PF02190	LON_substr_bdg	ATP-dependent protease La (LON) substrate-binding domain	4	5	6	1
PF02191	OLF	Olfactomedin-like domain	3	2	3	3
PF02192	PI3K_p85B	PI3-kinase family, p85-binding domain	2	7	3	1
PF02194	NA	PXA domain	5	5	5	1
PF02195	ParBc	ParB-like nuclease domain	1	2	1	0
PF02196	RBD	Raf-like Ras-binding domain	2	14	24	0
PF02197	RIIa	Regulatory subunit of type II PKA R-subunit	5	5	17	2
PF02198	SAM_PNT	Sterile alpha motif (SAM)/Pointed domain	5	12	27	2
PF02199	NA	Saposin A-type domain	2	4	3	0
PF02201	SWIB	SWIB/MDM2 domain	5	12	14	0
PF02204	VPS9	Vacuolar sorting protein 9 (VPS9) domain	6	15	24	4
PF02205	WH2	WH2 motif	3	6	9	1
PF02207	zf-UBR	Putative zinc finger in N-recognin (UBR box)	5	6	9	0
PF02208	NA	Sorbin homologous domain	1	0	25	0
PF02209	VHP	Villin headpiece domain	5	6	23	0
PF02210	Laminin_G_2	Laminin G domain	27	39	65	23
PF02212	GED	Dynamin GTPase effector domain	2	4	2	2
PF02213	GYF	GYF domain	2	2	3	0
PF02214	BTB_2	BTB/POZ domain	33	63	73	20
PF02218	NA	Repeat in HS1/Cortactin	1	7	4	0
PF02219	MTHFR	Methylenetetrahydrofolate reductase	1	7	1	3
PF02221	E1_DerP2_DerF2	ML domain	13	18	29	2
PF02223	Thymidylate_kin	Thymidylate kinase	2	1	4	1
PF02225	PA	PA domain	8	14	21	3
PF02229	PC4	Transcriptional Coactivator p15 (PC4)	1	2	5	0
PF02230	Abhydrolase_2	Phospholipase/Carboxylesterase	2	4	4	0
PF02233	PNTB	NAD(P) transhydrogenase beta subunit	1	1	1	2
PF02234	CDI	Cyclin-dependent kinase inhibitor	1	1	0	1
PF02237	BPL_C	Biotin protein ligase C terminal domain	1	1	4	0
PF02238	COX7a	Cytochrome c oxidase subunit VIIa	0	1	0	0
PF02244	Propep_M14	Carboxypeptidase activation peptide	2	1	9	0
PF02245	Pur_DNA_glyco	Methylpurine-DNA glycosylase (MPG)	0	3	4	2
PF02251	PA28_alpha	Proteasome activator pa28 alpha subunit	1	1	1	0
PF02252	PA28_beta	Proteasome activator pa28 beta subunit	1	1	1	0
PF02256	Fe_hyd_SSU	Iron hydrogenase small subunit	1	1	1	1
PF02257	NA	RFX DNA-binding domain	4	20	13	0
PF02259	FAT	FAT domain	4	8	11	2
PF02260	FATC	FATC domain	6	4	6	0
PF02262	Cbl_N	CBL proto-oncogene N-terminal domain 1	0	2	1	1
PF02263	GBP	Guanylate-binding protein, N-terminal domain	1	3	9	0
PF02267	Rib_hydrolayse	ADP-ribosyl cyclase	2	2	1	0
PF02268	TFIIA_gamma_N	Transcription initiation factor IIA, gamma subunit, helical domain	1	4	1	0
PF02269	TFIID-18kDa	Transcription initiation factor IID, 18kD subunit	2	3	2	0
PF02270	TFIIF_beta	Transcription initiation factor IIF, beta subunit	2	1	1	0
PF02271	UCR_14kD	Ubiquinol-cytochrome C reductase complex 14kD subunit	1	0	2	1
PF02272	DHHA1	DHHA1 domain	1	1	2	0
PF02274	Amidinotransf	Amidinotransferase	2	2	3	0
PF02275	CBAH	Linear amide C-N hydrolases, choloylglycine hydrolase family	3	3	2	0
PF02284	COX5A	Cytochrome c oxidase subunit Va	1	2	1	0
PF02290	SRP14	Signal recognition particle 14kD protein	1	1	1	0
PF02291	TFIID-31kDa	Transcription initiation factor IID, 31kD subunit	1	1	2	0
PF02295	z-alpha	Adenosine deaminase z-alpha domain	0	1	1	0
PF02296	Alpha_adaptin_C	Alpha adaptin AP2, C-terminal domain	1	1	2	0
PF02297	COX6B	Cytochrome oxidase c subunit VIb	2	3	2	0
PF02301	HORMA	HORMA domain	3	10	5	0
PF02310	B12-binding	B12 binding domain	2	3	2	0
PF02312	CBF_beta	Core binding factor beta subunit	1	1	2	0
PF02317	Octopine_DH	NAD/NADP octopine/nopaline dehydrogenase, alpha-helical domain	2	0	5	4
PF02318	FYVE_2	FYVE-type zinc finger	5	9	23	1

PF02319	NA	E2F/DP family winged-helix DNA-binding domain	3	9	10	0
PF02320	UCR_hinge	Ubiquinol-cytochrome C reductase hinge protein	1	1	1	0
PF02323	NA	Egg-laying hormone precursor	2	1	1	1
PF02330	MAM33	Mitochondrial glycoprotein	1	1	2	0
PF02338	OTU	OTU-like cysteine protease	10	21	54	12
PF02342	TerD	TerD domain	0	0	1	0
PF02347	GDC-P	Glycine cleavage system P-protein	1	1	2	0
PF02351	GDNF	GDNF/GAS1 domain	1	1	1	0
PF02353	CMAS	Mycolic acid cyclopropane synthetase	1	1	1	1
PF02359	CDC48_N	Cell division protein 48 (CDC48), N-terminal domain	1	1	2	0
PF02366	NA	Dolichyl-phosphate-mannose-protein mannosyltransferase	2	4	2	0
PF02368	Big_2	Bacterial Ig-like domain (group 2)	1	1	0	0
PF02373	JmjC	JmjC domain, hydroxylase	7	22	16	2
PF02374	ArsA_ATPase	Anion-transporting ATPase	1	1	1	0
PF02375	JmjN	jmjN domain	3	6	6	0
PF02376	CUT	CUT domain	3	8	14	4
PF02377	NA	Dishevelled specific domain	1	3	6	0
PF02378	NA	Phosphotransferase system, EIIC	0	1	0	0
PF02383	Syja_N	SacI homology domain	4	5	5	2
PF02389	NA	Cornifin (SPRR) family	6	9	0	6
PF02390	Methyltransf_4	Putative methyltransferase	1	1	1	0
PF02391	MoaE	MoaE protein	1	1	4	0
PF02403	Seryl_tRNA_N	Seryl-tRNA synthetase N-terminal domain	1	1	2	0
PF02405	NA	Permease MlaE	0	1	1	0
PF02407	Viral_Rep	Putative viral replication protein	0	0	1	2
PF02410	RsfS	Ribosomal silencing factor during starvation	0	2	1	0
PF02412	TSP_3	Thrombospondin type 3 repeat	1	1	4	1
PF02416	MttA_Hcf106	mttA/Hcf106 family	0	1	0	0
PF02423	OCD_Mu_crystall	Ornithine cyclodeaminase/mu-crystallin family	1	1	1	0
PF02434	Fringe	Fringe-like	13	19	15	12
PF02436	PYC_OADA	Conserved carboxylase domain	1	1	6	1
PF02437	Ski_Sno	SKI/SNO/DAC family	4	4	5	2
PF02439	NA	Adenovirus E3 region protein CR2	1	1	0	0
PF02441	Flavoprotein	Flavoprotein	1	1	1	0
PF02450	LCAT	Lecithin:cholesterol acyltransferase	1	2	1	2
PF02453	NA	Reticulon	3	5	9	1
PF02456	NA	Adenovirus IVa2 protein	1	0	0	0
PF02460	Patched	Patched family	10	13	21	19
PF02463	SMC_N	RecF/RecN/SMC N terminal domain	5	7	8	1
PF02466	Tim17	Tim17/Tim22/Tim23/Pmp24 family	5	4	5	0
PF02469	Fasciclin	Fasciclin domain	9	20	23	15
PF02475	Met_10	Met-10+ like-protein	2	3	4	1
PF02485	Branch	Core-2/I-Branching enzyme	21	16	18	13
PF02487	NA	CLN3 protein	1	7	3	0
PF02492	NA	CobW/HypB/UreG, nucleotide-binding domain	1	5	4	1
PF02493	MORN	MORN repeat	10	22	40	3
PF02494	NA	HYR domain	5	7	24	8
PF02504	NA	Fatty acid synthesis protein	0	1	0	0
PF02515	CoA_transf_3	CoA-transferase family III	2	12	6	1
PF02516	STT3	Oligosaccharyl transferase STT3 subunit	2	3	2	0
PF02517	CPBP	CAAX protease self-immunity	2	1	1	0
PF02518	HATPase_c	Histidine kinase-, DNA gyrase B-, and HSP90-like ATPase	9	13	13	3
PF02534	T4SS-DNA_transf	Type IV secretory system Conjugative DNA transfer	0	1	0	0
PF02535	NA	ZIP Zinc transporter	14	34	42	12
PF02536	mTERF	mTERF	2	2	2	1
PF02538	Hydantoinase_B	Hydantoinase B/oxoprolinase	2	1	2	0
PF02540	NAD_synthase	NAD synthase	2	3	2	0
PF02544	NA	3-oxo-5-alpha-steroid 4-dehydrogenase	2	7	7	1
PF02545	Maf	Maf-like protein	1	1	1	0
PF02550	AcetylCoA_hydro	Acetyl-CoA hydrolase/transferase N-terminal domain	1	0	3	0
PF02551	NA	Acyl-CoA thioesterase	0	1	0	0
PF02562	NA	PhoH-like protein	0	1	0	3
PF02567	PhzC-PhzF	Phenazine biosynthesis-like protein	2	1	1	0
PF02568	ThiI	Thiamine biosynthesis protein (ThiI)	0	1	0	0
PF02574	S-methyl_trans	Homocysteine S-methyltransferase	6	2	11	2
PF02576	DUF150	Putative ribosome maturation factor RimP	0	1	0	0
PF02578	Cu-oxidase_4	Multi-copper polyphenol oxidoreductase laccase	0	2	1	1
PF02580	Tyr_Deacylase	D-Tyr-tRNA(Tyr) deacylase	2	1	2	0
PF02582	NA	Uncharacterised ACR, YagE family COG1723	1	1	1	1
PF02585	PIG-L	GlcNAc-PI de-N-acetylase	1	2	0	0
PF02586	SRAP	SOS response associated peptidase (SRAP)	1	2	3	0
PF02594	NA	Uncharacterised ACR, YggU family COG1872	0	1	3	0
PF02598	Methyltrn_RNA_3	Putative RNA methyltransferase	1	1	1	1
PF02601	NA	Exonuclease VII, large subunit	0	0	1	0
PF02602	HEM4	Uroporphyrinogen-III synthase HemD	1	0	3	1
PF02603	Hpr_kinase_N	HPr Serine kinase N terminus	0	1	0	0
PF02607	B12-binding_2	B12 binding domain	1	2	1	1

PF02615	Ldh_2	Malate/L-lactate dehydrogenase	1	5	6	1
PF02617	ClpS	ATP-dependent Clp protease adaptor protein ClpS	1	1	3	0
PF02627	CMD	Carboxymuconolactone decarboxylase family	0	5	2	0
PF02628	COX15-CtaA	Cytochrome oxidase assembly protein	1	1	1	0
PF02629	CoA_binding	CoA binding domain	2	6	5	0
PF02630	SCO1-SenC	SCO1/SenC	4	2	1	0
PF02636	Methyltransf_28	Putative S-adenosyl-L-methionine-dependent methyltransferase	1	1	1	0
PF02637	GatB_Yqey	GatB domain	1	2	2	0
PF02638	NA	Glycosyl hydrolase-like 10	6	2	1	0
PF02646	NA	RmuC family	0	1	0	0
PF02661	Fic	Fic/DOC family	1	1	1	1
PF02666	PS_Dcarbxylase	Phosphatidylserine decarboxylase	1	2	2	1
PF02668	TauD	Taurine catabolism dioxygenase TauD, TfdA family	5	11	9	1
PF02671	PAH	Paired amphipathic helix repeat	1	3	6	1
PF02676	TYW3	Methyltransferase TYW3	1	2	2	0
PF02678	Pirin	Pirin	1	4	4	0
PF02686	Glu-tRNAGln	Glu-tRNAGln amidotransferase C subunit	1	0	0	0
PF02690	NA	Na+/Pi-cotransporter	3	5	7	6
PF02696	UPF0061	Uncharacterized ACR, YdiU/UPF0061 family	2	4	2	0
PF02698	NA	DUF218 domain	1	2	1	1
PF02709	Glyco_transf_7C	N-terminal domain of galactosyltransferase	11	47	31	2
PF02714	NA	Calcium-dependent channel, 7TM region, putative phosphate	1	8	8	0
PF02724	CDC45	CDC45-like protein	2	3	1	0
PF02727	Cu_amine_oxidN2	Copper amine oxidase, N2 domain	6	8	7	3
PF02728	Cu_amine_oxidN3	Copper amine oxidase, N3 domain	1	2	4	0
PF02729	OTCace_N	Aspartate/ornithine carbamoyltransferase, carbamoyl-P binding domain	2	3	6	0
PF02731	SKIP_SNW	SKIP/SNW domain	1	1	1	0
PF02732	ERCC4	ERCC4 domain	2	4	8	1
PF02733	Dak1	Dak1 domain	2	1	2	1
PF02734	Dak2	DAK2 domain	2	1	2	0
PF02735	Ku	Ku70/Ku80 beta-barrel domain	2	2	4	0
PF02736	Myosin_N	Myosin N-terminal SH3-like domain	5	18	9	2
PF02737	3HCDH_N	3-hydroxyacyl-CoA dehydrogenase, NAD binding domain	6	9	6	5
PF02738	Ald_Xan_dh_C2	Molybdopterin-binding domain of aldehyde dehydrogenase	8	21	6	11
PF02739	5_3_exonuc_N	5'-3' exonuclease, N-terminal resolvase-like domain	0	2	1	0
PF02743	dCache_1	Cache domain	4	5	20	0
PF02744	GalP_UDP_tr_C	Galactose-1-phosphate uridyl transferase, C-terminal domain	1	1	1	0
PF02746	MR_MLE_N	Mandelate racemase / muconate lactonizing enzyme, N-terminal domain	2	3	6	0
PF02747	PCNA_C	Proliferating cell nuclear antigen, C-terminal domain	1	1	1	0
PF02749	QRPTase_N	Quinolinate phosphoribosyl transferase, N-terminal domain	1	2	1	0
PF02750	Synapsin_C	Synapsin, ATP binding domain	1	1	1	1
PF02751	TFIIA_gamma_C	Transcription initiation factor IIA, gamma subunit	1	4	1	0
PF02752	Arrestin_C	Arrestin (or S-antigen), C-terminal domain	15	32	51	3
PF02755	RPEL	RPEL repeat	3	5	12	0
PF02759	RUN	RUN domain	12	28	35	1
PF02761	Cbl_N2	CBL proto-oncogene N-terminus, EF hand-like domain	1	2	1	0
PF02762	Cbl_N3	CBL proto-oncogene N-terminus, SH2-like domain	1	2	1	0
PF02765	POT1	Telomeric single stranded DNA binding POT1/CDC13	0	1	1	0
PF02769	AIRS_C	AIR synthase related protein, C-terminal domain	3	4	8	2
PF02770	Acyl-CoA_dh_M	Acyl-CoA dehydrogenase, middle domain	16	23	17	1
PF02771	Acyl-CoA_dh_N	Acyl-CoA dehydrogenase, N-terminal domain	11	14	12	2
PF02772	S-AdoMet_synt_M	S-adenosylmethionine synthetase, central domain	2	4	7	2
PF02773	S-AdoMet_synt_C	S-adenosylmethionine synthetase, C-terminal domain	2	3	7	1
PF02775	TPP_enzyme_C	Thiamine pyrophosphate enzyme, C-terminal TPP binding domain	4	4	6	0
PF02776	TPP_enzyme_N	Thiamine pyrophosphate enzyme, N-terminal TPP binding domain	4	4	6	0
PF02777	Sod_Fe_C	Iron/manganese superoxide dismutases, C-terminal domain	1	1	1	1
PF02778	tRNA_int_endo_N	tRNA intron endonuclease, N-terminal domain	1	1	1	0
PF02779	Transket_pyr	Transketolase, pyrimidine binding domain	7	8	13	1
PF02780	Transketolase_C	Transketolase, C-terminal domain	4	3	7	1
PF02781	G6PD_C	Glucose-6-phosphate dehydrogenase, C-terminal domain	2	5	4	3
PF02782	FGGY_C	FGGY family of carbohydrate kinases, C-terminal domain	4	6	9	0
PF02784	Orn_Arg_deC_N	Pyridoxal-dependent decarboxylase, pyridoxal binding domain	2	4	6	1
PF02785	Biotin_carb_C	Biotin carboxylase C-terminal domain	5	4	9	1
PF02786	CPSase_L_D2	Carbamoyl-phosphate synthase L chain, ATP binding domain	6	10	14	4
PF02787	CPSase_L_D3	Carbamoyl-phosphate synthetase large chain, oligomerisation domain	2	3	5	3
PF02789	Peptidase_M17_N	Cytosol aminopeptidase family, N-terminal domain	1	2	1	0
PF02790	COX2_TM	Cytochrome C oxidase subunit II, transmembrane domain	0	2	1	0
PF02791	NA	DDT domain	2	4	17	1
PF02792	Mago_nashi	Mago nashi protein	1	2	1	0
PF02793	HRM	Hormone receptor domain	12	27	63	4
PF02798	GST_N	Glutathione S-transferase, N-terminal domain	14	31	30	0
PF02799	NMT_C	Myristoyl-CoA:protein N-myristoyltransferase, C-terminal domain	1	2	1	0
PF02800	Gp_dh_C	Glyceraldehyde 3-phosphate dehydrogenase, C-terminal domain	1	7	1	3
PF02801	Ketoacyl-synt_C	Beta-ketoacyl synthase, C-terminal domain	9	9	4	4
PF02803	Thiolase_C	Thiolase, C-terminal domain	7	11	9	0
PF02806	Alpha-amylase_C	Alpha amylase, C-terminal all-beta domain	3	10	5	0
PF02807	ATP-gua_PtransN	ATP:guanido phosphotransferase, N-terminal domain	5	5	2	3

PF02809	UIM	Ubiquitin interaction motif	3	7	10	0
PF02812	ELFV_dehydrog_N	Glu/Leu/Phe/Val dehydrogenase, dimerisation domain	1	2	3	1
PF02815	MIR	MIR domain	7	18	22	1
PF02816	Alpha_kinase	Alpha-kinase family	8	16	15	6
PF02817	E3_binding	e3 binding domain	1	5	3	0
PF02819	NA	Spider toxin	0	0	1	0
PF02820	MBT	mbt repeat	7	8	10	5
PF02822	Antistasin	Antistasin family	10	25	14	2
PF02823	ATP-synt_DE_N	ATP synthase, Delta/Epsilon chain, beta-sandwich domain	1	2	1	0
PF02824	TGS	TGS domain	3	6	5	0
PF02825	WWE	WWE domain	5	14	70	8
PF02826	2-Hacid_dh_C	D-isomer specific 2-hydroxyacid dehydrogenase, NAD binding domain	9	19	17	8
PF02827	PKI	cAMP-dependent protein kinase inhibitor	1	0	0	0
PF02828	NA	L27 domain	4	16	18	0
PF02833	DHHA2	DHHA2 domain	1	0	1	0
PF02836	Glyco_hydro_2_C	Glycosyl hydrolases family 2, TIM barrel domain	6	5	6	0
PF02837	Glyco_hydro_2_N	Glycosyl hydrolases family 2, sugar binding domain	7	4	5	0
PF02838	Glyco_hydro_20b	Glycosyl hydrolase family 20, domain 2	5	7	12	2
PF02840	Prp18	Prp18 domain	1	1	2	0
PF02841	GBP_C	Guanylate-binding protein, C-terminal domain	1	0	4	0
PF02843	GARS_C	Phosphoribosylglycinamide synthetase, C domain	1	2	2	0
PF02844	GARS_N	Phosphoribosylglycinamide synthetase, N domain	1	2	2	0
PF02845	CUE	CUE domain	6	5	17	1
PF02847	MA3	MA3 domain	5	18	22	0
PF02852	Pyr_redox_dim	Pyridine nucleotide-disulphide oxidoreductase, dimerisation domain	5	5	6	0
PF02854	MIF4G	MIF4G domain	8	27	24	2
PF02862	NA	DDHD domain	3	6	13	2
PF02864	STAT_bind	STAT protein, DNA binding domain	2	11	3	0
PF02865	STAT_int	STAT protein, protein interaction domain	1	6	0	0
PF02866	Ldh_1_C	lactate/malate dehydrogenase, alpha/beta C-terminal domain	3	4	4	0
PF02867	Ribonuc_red_lgC	Ribonucleotide reductase, barrel domain	1	2	3	2
PF02868	Peptidase_M4_C	Thermolysin metallopeptidase, alpha-helical domain	1	1	1	7
PF02872	5_nucleotid_C	5'-nucleotidase, C-terminal domain	3	3	11	6
PF02874	ATP-synt_ab_N	ATP synthase alpha/beta family, beta-barrel domain	4	5	7	0
PF02877	PARP_reg	Poly(ADP-ribose) polymerase, regulatory domain	4	8	7	1
PF02878	PGM_PMM_I	Phosphoglucomutase/phosphomannomutase, alpha/beta/alpha domain I	3	4	7	2
PF02879	PGM_PMM_II	Phosphoglucomutase/phosphomannomutase, alpha/beta/alpha domain II	2	3	5	0
PF02880	PGM_PMM_III	Phosphoglucomutase/phosphomannomutase, alpha/beta/alpha domain III	2	4	5	0
PF02881	SRP54_N	SRP54-type protein, helical bundle domain	2	3	2	0
PF02882	THF_DHG_CYH_C	Tetrahydrofolate dehydrogenase/cyclohydrolase, NAD(P)-binding domain	2	7	2	2
PF02883	Alpha_adaptinC2	Adaptin C-terminal domain	3	6	14	0
PF02885	Glycos_trans_3N	Glycosyl transferase family, helical bundle domain	1	4	2	0
PF02886	LBP_BPI_CETP_C	LBP / BPI / CETP family, C-terminal domain	1	11	2	1
PF02887	PK_C	Pyruvate kinase, alpha/beta domain	2	11	4	2
PF02888	CaMBD	Calmodulin binding domain	3	1	25	0
PF02889	Sec63	Sec63 Brl domain	7	6	10	1
PF02891	zf-MIZ	MIZ/SP-RING zinc finger	2	10	10	0
PF02892	zf-BED	BED zinc finger	0	4	5	0
PF02893	GRAM	GRAM domain	7	14	19	0
PF02894	GFO_IDH_MocA_C	Oxidoreductase family, C-terminal alpha/beta domain	3	1	10	2
PF02896	PEP-utilizers_C	PEP-utilising enzyme, TIM barrel domain	0	1	0	0
PF02897	Peptidase_S9_N	Prolyl oligopeptidase, N-terminal beta-propeller domain	1	1	2	2
PF02898	NO_synthase	Nitric oxide synthase, oxygenase domain	1	3	7	2
PF02900	LigB	Catalytic LigB subunit of aromatic ring-opening dioxygenase	0	0	1	0
PF02901	PFL-like	Pyruvate formate lyase-like	0	1	0	0
PF02902	Peptidase_C48	Ulp1 protease family, C-terminal catalytic domain	5	5	17	1
PF02906	Fe_hyd_lg_C	Iron only hydrogenase large subunit, C-terminal domain	1	1	2	2
PF02910	Succ_DH_flav_C	Fumarate reductase flavoprotein C-term	1	2	1	0
PF02911	Formyl_trans_C	Formyl transferase, C-terminal domain	2	6	5	0
PF02913	FAD-oxidase_C	FAD linked oxidases, C-terminal domain	3	3	4	2
PF02919	Topoisom_I_N	Eukaryotic DNA topoisomerase I, DNA binding fragment	1	1	2	1
PF02921	UCR_TM	Ubiquinol cytochrome reductase transmembrane region	1	2	1	0
PF02922	CBM_48	Carbohydrate-binding module 48 (Isoamylase N-terminal domain)	1	1	1	0
PF02926	THUMP	THUMP domain	2	3	2	2
PF02928	zf-C5HC2	C5HC2 zinc finger	2	5	4	0
PF02931	Neur_chan_LBD	Neurotransmitter-gated ion-channel ligand binding domain	87	107	236	56
PF02932	Neur_chan_memb	Neurotransmitter-gated ion-channel transmembrane region	74	84	211	45
PF02933	CDC48_2	Cell division protein 48 (CDC48), domain 2	1	2	2	0
PF02934	GatB_N	GatB/GatE catalytic domain	1	3	2	0
PF02935	COX7C	Cytochrome c oxidase subunit VIIc	0	7	0	0
PF02936	COX4	Cytochrome c oxidase subunit IV	2	2	1	1
PF02937	COX6C	Cytochrome c oxidase subunit VIc	0	0	0	1
PF02938	GAD	GAD domain	0	1	0	0
PF02939	UcrQ	UcrQ family	1	1	0	0
PF02944	NA	BESS motif	0	1	0	0
PF02945	Endonuclease_7	Recombination endonuclease VII	0	3	3	0
PF02946	GTF2I	GTF2I-like repeat	0	0	1	0

PF02949	7tm_6	7tm Odorant receptor	2	1	0	0
PF02953	NA	Tim10/DDP family zinc finger	6	1	6	3
PF02961	BAF	Barrier to autointegration factor	4	2	3	0
PF02965	Met_synt_B12	Vitamin B12 dependent methionine synthase, activation domain	1	1	1	0
PF02966	DIM1	Mitosis protein DIM1	2	3	3	1
PF02969	TAF	TATA box binding protein associated factor (TAF)	2	5	3	1
PF02970	TBCA	Tubulin binding cofactor A	1	0	1	0
PF02971	FTCD	Formiminotransferase domain	1	1	3	2
PF02978	SRP_SPB	Signal peptide binding domain	1	3	1	0
PF02984	Cyclin_C	Cyclin, C-terminal domain	10	14	23	0
PF02985	HEAT	HEAT repeat	7	26	12	2
PF02990	NA	Endomembrane protein 70	5	5	5	1
PF02991	Atg8	Autophagy protein Atg8 ubiquitin like	5	3	5	0
PF02992	NA	Transposase family tnp2	0	0	7	0
PF02995	NA	Protein of unknown function (DUF229)	14	41	33	16
PF02996	Prefoldin	Prefoldin subunit	4	3	5	0
PF03000	NA	NPH3 family	0	1	1	1
PF03006	HlyIII	Haemolysin-III related	8	7	21	5
PF03007	NA	Wax ester synthase-like Acyl-CoA acyltransferase domain	0	0	3	1
PF03008	NA	Archaea bacterial proteins of unknown function	0	0	1	0
PF03009	GDPD	Glycerophosphoryl diester phosphodiesterase family	4	10	8	0
PF03015	NA	Male sterility protein	2	1	3	0
PF03016	NA	Exostosin family	3	13	5	2
PF03020	LEM	LEM domain	1	4	4	2
PF03022	MRJP	Major royal jelly protein	0	9	1	2
PF03024	Folate_rec	Folate receptor family	4	3	3	1
PF03028	Dynein_heavy	Dynein heavy chain and region D6 of dynein motor	18	44	73	97
PF03029	ATP_bind_1	Conserved hypothetical ATP binding protein	3	3	3	1
PF03031	NIF	NLI interacting factor-like phosphatase	6	10	7	1
PF03034	NA	Phosphatidyl serine synthase	2	3	4	1
PF03036	NA	Perilipin family	1	1	2	0
PF03045	DAN	DAN domain	2	2	2	4
PF03051	Peptidase_C1_2	Peptidase C1-like family	0	0	1	0
PF03054	NA	tRNA methyl transferase	1	2	1	0
PF03055	RPE65	Retinal pigment epithelial membrane protein	5	4	3	15
PF03061	4HBT	Thioesterase superfamily	2	5	2	0
PF03062	MBOAT	MBOAT, membrane-bound O-acyltransferase family	7	18	17	1
PF03066	Nucleoplasmin	Nucleoplasmin	1	5	2	0
PF03067	LPMO_10	Chitin binding domain	13	14	38	6
PF03071	GNT-I	GNT-I family	3	5	7	1
PF03073	TspO_MBR	TspO/MBR family	1	1	2	2
PF03074	NA	Glutamate-cysteine ligase	1	3	1	0
PF03079	ARD	ARD/ARD' family	1	2	1	1
PF03081	Exo70	Exo70 exocyst complex subunit	1	4	2	2
PF03083	MtN3_slv	Sugar efflux transporter for intercellular exchange	1	7	3	0
PF03088	Str_synth	Strictosidine synthase	2	12	3	1
PF03091	CutA1	CutA1 divalent ion tolerance protein	1	4	1	0
PF03095	PTPA	Phosphotyrosyl phosphate activator (PTPA) protein	1	3	2	0
PF03096	Ndr	Ndr family	1	4	8	0
PF03097	BRO1	BRO1-like domain	4	4	10	2
PF03098	An_peroxidase	Animal haem peroxidase	24	23	65	34
PF03099	BPL_LplA_LipB	Biotin/lipoate A/B protein ligase family	3	8	6	1
PF03101	NA	FAR1 DNA-binding domain	0	5	0	0
PF03104	DNA_pol_B_exo1	DNA polymerase family B, exonuclease domain	5	5	14	2
PF03105	SPX	SPX domain	1	1	5	0
PF03109	ABC1	ABC1 family	3	2	6	0
PF03114	BAR	BAR domain	6	10	22	0
PF03118	RNA_pol_A_CTD	Bacterial RNA polymerase, alpha chain C terminal domain	0	1	0	0
PF03121	NA	Herpesviridae UL52/UL70 DNA primase	1	0	3	0
PF03124	NA	EXS family	1	1	5	0
PF03126	NA	Plus-3 domain	1	1	1	0
PF03127	GAT	GAT domain	3	7	9	0
PF03128	NA	CXCXC repeat	0	3	2	2
PF03129	HGTP_anticodon	Anticodon binding domain	7	13	13	0
PF03130	HEAT_PBS	PBS lyase HEAT-like repeat	0	2	1	0
PF03131	bZIP_Maf	bZIP Maf transcription factor	3	7	9	3
PF03133	TTL	Tubulin-tyrosine ligase family	14	46	90	9
PF03134	NA	TB2/DP1, HVA22 family	2	8	4	0
PF03137	NA	Organic Anion Transporter Polypeptide (OATP) family	9	31	42	42
PF03142	NA	Chitin synthase	13	11	29	9
PF03143	GTP_EFTU_D3	Elongation factor Tu C-terminal domain	6	10	6	5
PF03144	GTP_EFTU_D2	Elongation factor Tu domain 2	12	19	11	6
PF03145	Sina	Seven in absentia protein family	2	2	1	1
PF03146	NtA	Agrin NtA domain	1	1	6	1
PF03147	FDX-ACB	Ferredoxin-fold anticodon binding domain	2	1	7	1
PF03148	NA	Tektin family	10	9	15	16
PF03151	NA	Triose-phosphate Transporter family	14	24	9	4

PF03152	UFD1	Ubiquitin fusion degradation protein UFD1	1	2	1	1
PF03153	TFIIA	Transcription factor IIA, alpha/beta subunit	1	1	2	0
PF03154	NA	Atrophin-1 family	0	4	4	2
PF03155	NA	ALG6, ALG8 glycosyltransferase family	2	4	3	0
PF03157	NA	High molecular weight glutenin subunit	1	0	0	0
PF03159	XRN_N	XRN 5'-3' exonuclease N-terminus	0	3	3	0
PF03160	Calx-beta	Calx-beta domain	9	12	39	10
PF03164	NA	Trafficking protein Mon1	1	1	1	0
PF03165	MH1	MH1 domain	5	19	12	4
PF03166	MH2	MH2 domain	4	12	10	4
PF03167	UDG	Uracil DNA glycosylase superfamily	4	3	5	0
PF03171	2OG-FeII_Oxy	2OG-Fe(II) oxygenase superfamily	8	1	10	3
PF03173	CHB_HEX	Putative carbohydrate binding domain	4	9	12	4
PF03174	CHB_HEX_C	Chitobiase/beta-hexosaminidase C-terminal domain	1	1	3	1
PF03175	DNA_pol_B_2	DNA polymerase type B, organellar and viral	178	0	3	0
PF03177	Nucleoporin_C	Non-repetitive/WGA-negative nucleoporin C-terminal	2	3	2	0
PF03178	CPSF_A	CPSF A subunit region	3	3	4	1
PF03179	V-ATPase_G	Vacuolar (H+)-ATPase G subunit	1	1	1	0
PF03184	NA	DDE superfamily endonuclease	9	24	24	2
PF03188	Cytochrom_B561	Eukaryotic cytochrome b561	8	23	27	1
PF03189	Otopetrin	Otopetrin	6	8	7	5
PF03190	NA	Protein of unknown function, DUF255	1	1	2	0
PF03194	NA	LUC7 N_terminus	2	3	4	0
PF03199	GSH_synthase	Eukaryotic glutathione synthase	3	1	1	0
PF03200	NA	Glycosyl hydrolase family 63 C-terminal domain	3	4	2	1
PF03208	NA	PRA1 family protein	2	2	3	0
PF03215	NA	Rad17 cell cycle checkpoint protein	1	5	2	0
PF03221	HTH_Tnp_Tc5	Tc5 transposase DNA-binding domain	5	25	10	2
PF03222	NA	Tryptophan/tyrosine permease family	0	0	1	0
PF03223	V-ATPase_C	V-ATPase subunit C	1	1	2	0
PF03224	V-ATPase_H_N	V-ATPase subunit H	2	2	4	0
PF03226	Yippee-Mis18	Yippee zinc-binding/DNA-binding /Mis18, centromere assembly	3	5	4	2
PF03227	NA	Gamma interferon inducible lysosomal thiol reductase (GILT)	2	1	3	1
PF03232	NA	Ubiquinone biosynthesis protein COQ7	1	1	1	0
PF03234	CDC37_N	Cdc37 N terminal kinase binding	1	1	0	0
PF03248	NA	Rer1 family	1	1	3	0
PF03250	NA	Tropomodulin	1	0	1	0
PF03256	ANAPC10	Anaphase-promoting complex, subunit 10 (APC10)	5	6	7	1
PF03259	Robl_LC7	Roadblock/LC7 domain	2	6	5	0
PF03261	CDK5_activator	Cyclin-dependent kinase 5 activator protein	1	1	2	1
PF03265	DNase_II	Deoxyribonuclease II	2	2	22	1
PF03266	NA	NTPase	1	2	1	0
PF03268	NA	Caenorhabditis protein of unknown function, DUF267	0	0	0	1
PF03271	EB1	EB1-like C-terminal motif	1	3	9	0
PF03281	Mab-21	Mab-21 protein	2	3	145	9
PF03283	PAE	Pectinacetylesterase	2	3	3	2
PF03291	Pox_MCEL	mRNA capping enzyme	1	1	2	1
PF03297	Ribosomal_S25	S25 ribosomal protein	1	2	1	1
PF03298	NA	Stanniocalcin family	2	1	2	0
PF03299	NA	Transcription factor AP-2	1	3	4	1
PF03301	Trp_dioxygenase	Tryptophan 2,3-dioxygenase	2	4	3	0
PF03308	MeaB	ArgK protein	1	2	1	0
PF03311	Cornichon	Cornichon protein	2	3	5	0
PF03313	NA	Serine dehydratase alpha chain	0	0	1	0
PF03315	SDH_beta	Serine dehydratase beta chain	0	0	1	0
PF03318	ETX_MTX2	Clostridium epsilon toxin ETX/Bacillus mosquitocidal toxin MTX2	1	5	6	4
PF03321	GH3	GH3 auxin-responsive promoter	2	1	2	3
PF03328	HpcH_HpaI	HpcH/HpaI aldolase/citrate lyase family	1	3	1	0
PF03332	PMM	Eukaryotic phosphomannomutase	1	1	2	1
PF03343	SART-1	SART-1 family	1	2	2	0
PF03344	NA	Daxx N-terminal Rassf1C-interacting domain	0	0	7	0
PF03345	DDOST_48kD	Oligosaccharyltransferase 48 kDa subunit beta	1	2	1	0
PF03348	Serinc	Serine incorporator (Serinc)	2	9	5	2
PF03351	NA	DOMON domain	11	23	28	1
PF03357	Snf7	Snf7	9	15	13	4
PF03358	FMN_red	NADPH-dependent FMN reductase	1	2	3	0
PF03359	GKAP	Guanylate-kinase-associated protein (GKAP) protein	2	2	13	2
PF03360	Glyco_transf_43	Glycosyltransferase family 43	2	5	2	0
PF03364	Polyketide_cyc	Polyketide cyclase / dehydrase and lipid transport	1	1	1	1
PF03366	YEATS	YEATS family	3	5	4	1
PF03367	NA	ZPR1 zinc-finger domain	1	3	2	0
PF03368	NA	Dicer dimerisation domain	1	1	3	0
PF03370	CBM_21	Carbohydrate/starch-binding module (family 21)	1	7	4	2
PF03371	PRP38	PRP38 family	2	6	5	0
PF03372	Exo_endo_phos	Endonuclease/Exonuclease/phosphatase family	47	66	60	1
PF03378	NA	CAS/CSE protein, C-terminus	1	1	1	0
PF03381	CDC50	LEM3 (ligand-effect modulator 3) family / CDC50 family	1	5	3	2

PF03382	NA	Mycoplasma protein of unknown function, DUF285	0	0	0	1
PF03383	NA	Caenorhabditis serpentine receptor-like protein, class xa	0	0	0	1
PF03385	NA	Protein of unknown function, DUF288	15	4	0	0
PF03388	Lectin_leg-like	Legume-like lectin family	2	3	2	0
PF03398	Ist1	Regulator of Vps4 activity in the MVB pathway	2	1	1	2
PF03399	SAC3_GANP	SAC3/GANP/Nin1/mts3/eIF-3 p25 family	3	13	5	2
PF03403	PAF-AH_p_II	Platelet-activating factor acetylhydrolase, isoform II	0	1	1	0
PF03404	Mo-co_dimer	Mo-co oxidoreductase dimerisation domain	1	1	1	1
PF03407	NA	Nucleotide-diphospho-sugar transferase	0	0	9	4
PF03413	PepSY	Peptidase propeptide and YPEB domain	0	0	1	4
PF03416	NA	Peptidase family C54	2	2	2	0
PF03417	NA	Acyl-coenzyme A:6-aminopenicillanic acid acyl-transferase	2	3	0	1
PF03422	CBM_6	Carbohydrate binding module (family 6)	1	0	0	0
PF03435	Sacchrp_dh_NADP	Saccharopine dehydrogenase NADP binding domain	2	2	2	1
PF03437	NA	BtpA family	1	1	3	0
PF03439	Spt5-NGN	Early transcription elongation factor of RNA pol II, NGN section	1	1	0	0
PF03441	FAD_binding_7	FAD binding domain of DNA photolyase	5	11	10	1
PF03446	NAD_binding_2	NAD binding domain of 6-phosphogluconate dehydrogenase	4	3	4	0
PF03447	NAD_binding_3	Homoserine dehydrogenase, NAD binding domain	1	0	1	0
PF03448	MgtE_N	MgtE intracellular N domain	0	2	0	5
PF03449	GreA_GreB_N	Transcription elongation factor, N-terminal	0	2	0	0
PF03450	CO_deh_flav_C	CO dehydrogenase flavoprotein C-terminal domain	6	13	4	3
PF03451	HELP	HELP motif	5	7	23	1
PF03452	NA	Anp1	1	0	2	1
PF03453	MoeA_N	MoeA N-terminal region (domain I and II)	1	1	1	0
PF03454	MoeA_C	MoeA C-terminal region (domain IV)	1	1	1	1
PF03455	dDENN	dDENN domain	5	16	18	1
PF03456	uDENN	uDENN domain	6	19	25	1
PF03460	NIR_SIR_ferr	Nitrite/Sulfite reductase ferredoxin-like half domain	0	0	1	0
PF03462	PCRF	PCRF domain	1	3	1	0
PF03463	eRF1_1	eRF1 domain 1	2	2	4	0
PF03464	eRF1_2	eRF1 domain 2	2	2	4	0
PF03465	eRF1_3	eRF1 domain 3	3	2	5	0
PF03466	LysR_substrate	LysR substrate binding domain	0	0	1	0
PF03467	NA	Smg-4/UPF3 family	1	2	1	0
PF03473	MOSC	MOSC domain	6	5	4	1
PF03474	NA	DMRTA motif	0	4	2	2
PF03476	MOSC_N	MOSC N-terminal beta barrel domain	6	6	4	2
PF03477	ATP-cone	ATP cone domain	1	1	2	1
PF03478	NA	Protein of unknown function (DUF295)	0	0	1	0
PF03479	DUF296	Domain of unknown function (DUF296)	1	1	1	0
PF03483	B3_4	B3/4 domain	2	1	2	0
PF03484	B5	tRNA synthetase B5 domain	1	0	1	1
PF03485	Arg_tRNA_synt_N	Arginyl tRNA synthetase N terminal domain	1	1	2	1
PF03489	SapB_2	Saposin-like type B, region 2	3	7	4	3
PF03492	Methyltransf_7	SAM dependent carboxyl methyltransferase	1	0	8	1
PF03493	BK_channel_a	Calcium-activated BK potassium channel alpha subunit	5	16	27	0
PF03496	ADPrib_exo_Tox	ADP-ribosyltransferase exoenzyme	1	0	0	0
PF03501	S10_plectin	Plectin/S10 domain	1	1	1	0
PF03517	Voldacs	Regulator of volume decrease after cellular swelling	0	2	2	0
PF03520	KCNQ_channel	KCNQ voltage-gated potassium channel	3	4	9	0
PF03522	SLC12	Solute carrier family 12	5	8	6	3
PF03524	CagX	Conjugal transfer protein	0	1	0	0
PF03528	NA	Rabaptin	1	0	1	2
PF03530	SK_channel	Calcium-activated SK potassium channel	5	5	26	3
PF03531	SSrecog	Structure-specific recognition protein (SSRP1)	1	1	4	0
PF03537	Glyco_hydro_114	Glycoside-hydrolase family GH114	1	0	3	2
PF03540	TFIID_30kDa	Transcription initiation factor TFIID 23-30kDa subunit	1	1	2	0
PF03542	NA	Tuberin	1	1	1	0
PF03547	NA	Membrane transport protein	1	5	15	1
PF03556	Cullin_binding	Cullin binding	2	4	5	1
PF03561	Allantoicase	Allantoicase repeat	0	0	2	0
PF03564	NA	Protein of unknown function (DUF1759)	5	0	11	0
PF03567	Sulfotransfer_2	Sulfotransferase family	12	28	15	27
PF03568	Peptidase_C50	Peptidase family C50	1	1	2	1
PF03571	Peptidase_M49	Peptidase family M49	2	1	4	0
PF03575	Peptidase_S51	Peptidase family S51	1	0	2	0
PF03577	Peptidase_C69	Peptidase family C69	1	4	4	0
PF03587	EMG1	EMG1/NEP1 methyltransferase	1	1	1	0
PF03600	NA	Citrate transporter	3	1	1	0
PF03602	Cons_hypoth95	Conserved hypothetical protein 95	0	1	0	0
PF03604	DNA_RNApol_7kD	DNA directed RNA polymerase, 7 kDa subunit	1	0	0	0
PF03607	DCX	Doublecortin	4	4	54	4
PF03615	NA	GCM motif protein	1	1	2	0
PF03619	NA	Organic solute transporter Ostalpha	4	7	8	2
PF03623	Focal_AT	Focal adhesion targeting region	1	11	22	0
PF03629	SASA	Domain of unknown function (DUF303)	7	13	2	0

PF03630	Fumble	Fumble	2	3	2	2
PF03632	Glyco_hydro_65m	Glycosyl hydrolase family 65 central catalytic domain	2	3	2	3
PF03635	Vps35	Vacuolar protein sorting-associated protein 35	1	1	2	1
PF03637	Mob1_phocein	Mob1/phocein family	4	10	9	2
PF03638	TCR	Tesmin/TSO1-like CXC domain, cysteine-rich domain	1	1	1	1
PF03639	Glyco_hydro_81	Glycosyl hydrolase family 81	1	2	2	0
PF03643	Vps26	Vacuolar protein sorting-associated protein 26	2	2	3	0
PF03644	Glyco_hydro_85	Glycosyl hydrolase family 85	1	2	1	0
PF03645	Tctex-1	Tctex-1 family	13	9	32	6
PF03647	NA	Transmembrane proteins 14C	1	1	1	0
PF03650	NA	Uncharacterised protein family (UPF0041)	3	3	3	0
PF03656	Pam16	Pam16	1	1	1	0
PF03657	UPF0113	Uncharacterised protein family (UPF0113)	1	1	1	0
PF03660	PHF5	PHF5-like protein	1	1	1	0
PF03661	NA	Uncharacterised protein family (UPF0121)	1	1	2	0
PF03662	Glyco_hydro_79n	Glycosyl hydrolase family 79, N-terminal domain	3	11	2	2
PF03663	Glyco_hydro_76	Glycosyl hydrolase family 76	0	0	1	0
PF03665	NA	Uncharacterised protein family (UPF0172)	1	1	1	0
PF03666	NPR3	Nitrogen Permease regulator of amino acid transport activity 3	1	1	1	1
PF03669	NA	Uncharacterised protein family (UPF0139)	1	1	1	0
PF03670	NA	Uncharacterised protein family (UPF0184)	1	1	1	1
PF03671	Ufm1	Ubiquitin fold modifier 1 protein	1	1	1	0
PF03676	NA	Uncharacterised protein family (UPF0183)	2	1	1	0
PF03690	NA	Uncharacterised protein family (UPF0160)	2	2	5	1
PF03694	NA	Erg28 like protein	1	1	0	0
PF03702	AnmK	Uncharacterised protein family (UPF0075)	1	4	1	2
PF03712	Cu2_monoox_C	Copper type II ascorbate-dependent monooxygenase, C-terminal domain	15	24	20	18
PF03715	NA	Noc2p family	2	1	1	0
PF03718	Glyco_hydro_49	Glycosyl hydrolase family 49	2	0	0	0
PF03719	Ribosomal_S5_C	Ribosomal protein S5, C-terminal domain	2	3	3	0
PF03720	UDPG_MGDP_dh_C	UDP-glucose/GDP-mannose dehydrogenase family, UDP binding domain	1	2	3	0
PF03721	UDPG_MGDP_dh_N	UDP-glucose/GDP-mannose dehydrogenase family, NAD binding domain	1	5	3	1
PF03725	RNase_PH_C	3' exoribonuclease family, domain 2	4	7	5	1
PF03726	PNPase	Polyribonucleotide nucleotidyltransferase, RNA binding domain	0	1	1	0
PF03727	Hexokinase_2	Hexokinase	4	5	7	1
PF03730	Ku_C	Ku70/Ku80 C-terminal arm	2	3	4	1
PF03731	Ku_N	Ku70/Ku80 N-terminal alpha/beta domain	2	2	4	0
PF03732	Retrotrans_gag	Retrotransposon gag protein	5	0	7	2
PF03733	NA	Inner membrane component domain	1	3	1	0
PF03735	ENT	ENT domain	1	0	3	0
PF03736	NA	EPTP domain	1	1	3	2
PF03747	ADP_ribosyl_GH	ADP-ribosylglycohydrolase	6	9	14	1
PF03762	NA	Vitelline membrane outer layer protein I (VOMI)	0	0	1	0
PF03764	EFG_IV	Elongation factor G, domain IV	4	7	5	4
PF03765	CRAL_TRIO_N	CRAL/TRIO, N-terminal domain	3	6	6	0
PF03770	IPK	Inositol polyphosphate kinase	4	20	22	9
PF03781	FGE-sulfatase	Sulfatase-modifying factor enzyme 1	3	6	4	0
PF03782	NA	AMOP domain	3	1	13	3
PF03792	NA	PBC domain	1	2	19	1
PF03793	PASTA	PASTA domain	0	0	1	0
PF03796	DnaB_C	DnaB-like helicase C terminal domain	0	2	0	0
PF03798	NA	TLC domain	9	15	13	2
PF03800	NA	Nuf2 family	0	1	1	0
PF03801	NA	HEC/Ndc80p family	1	1	1	2
PF03803	NA	Scramblase	5	12	19	1
PF03807	F420_oxidored	NADP oxidoreductase coenzyme F420-dependent	3	6	6	1
PF03810	IBN_N	Importin-beta N-terminal domain	13	19	18	1
PF03813	Nrap	Nrap protein	1	2	4	0
PF03815	LCCL	LCCL domain	1	0	1	0
PF03819	MazG	MazG nucleotide pyrophosphohydrolase domain	1	0	2	0
PF03820	NA	Tricarboxylate carrier	3	7	4	1
PF03821	NA	Golgi 4-transmembrane spanning transporter	1	1	1	0
PF03825	NA	Nucleoside H+ symporter	0	1	1	0
PF03826	NA	OAR domain	7	8	15	9
PF03828	PAP_assoc	Cid1 family poly A polymerase	4	4	6	0
PF03834	Rad10	Binding domain of DNA repair protein Ercc1 (rad10/Swi10)	2	1	1	0
PF03835	Rad4	Rad4 transglutaminase-like domain	1	0	1	0
PF03836	NA	RasGAP C-terminus	1	5	6	0
PF03839	NA	Translocation protein Sec62	1	1	1	1
PF03847	TFIID_20kDa	Transcription initiation factor TFIID subunit A	1	9	4	1
PF03848	NA	Tellurite resistance protein TehB	0	0	0	1
PF03849	Tfb2	Transcription factor Tfb2	1	2	1	0
PF03850	Tfb4	Transcription factor Tfb4	1	1	1	0
PF03853	YjeF_N	YjeF-related protein N-terminus	2	4	4	3
PF03859	NA	CG-1 domain	0	1	5	1
PF03861	ANTAR	ANTAR domain	0	0	1	0
PF03870	RNA_pol_Rpb8	RNA polymerase Rpb8	1	1	1	0

PF03871	RNA_pol_Rpb5_N	RNA polymerase Rpb5, N-terminal domain	1	4	1	0
PF03874	RNA_pol_Rpb4	RNA polymerase Rpb4	2	3	2	1
PF03876	SHS2_Rpb7-N	SHS2 domain found in N terminus of Rpb7p/Rpc25p/MJ0397	2	5	3	0
PF03878	NA	YIF1	1	2	5	0
PF03879	NA	Cgr1 family	0	0	1	0
PF03881	Fructosamin_kin	Fructosamine kinase	3	7	1	0
PF03896	NA	Translocon-associated protein (TRAP), alpha subunit	1	1	1	0
PF03900	Porphobil_deamC	Porphobilinogen deaminase, C-terminal domain	1	2	3	0
PF03901	NA	Alg9-like mannosyltransferase family	6	9	3	4
PF03908	NA	Sec20	1	4	1	0
PF03909	BSD	BSD domain	3	9	5	0
PF03911	Sec61_beta	Sec61beta family	1	1	1	0
PF03914	CBF	CBF/Mak21 family	3	3	5	0
PF03915	NA	Actin interacting protein 3	3	3	9	0
PF03917	GSH_synth_ATP	Eukaryotic glutathione synthase, ATP binding domain	4	1	1	0
PF03919	mRNA_cap_C	mRNA capping enzyme, C-terminal domain	3	1	1	0
PF03920	NA	Groucho/TLE N-terminal Q-rich domain	2	1	4	0
PF03931	Skp1_POZ	Skp1 family, tetramerisation domain	2	2	2	2
PF03932	CutC	CutC family	1	1	3	0
PF03937	Sdh5	Flavinator of succinate dehydrogenase	1	3	1	0
PF03939	Ribosomal_L23eN	Ribosomal protein L23, N-terminal domain	1	2	2	0
PF03941	INCENP_ARK-bind	Inner centromere protein, ARK binding region	1	1	1	0
PF03942	NA	DTW domain	2	4	3	1
PF03943	TAP_C	TAP C-terminal domain	1	1	1	0
PF03946	Ribosomal_L11_N	Ribosomal protein L11, N-terminal domain	2	5	4	0
PF03947	Ribosomal_L2_C	Ribosomal Proteins L2, C-terminal domain	2	6	3	1
PF03949	Malic_M	Malic enzyme, NAD binding domain	3	3	2	1
PF03950	tRNA-synt_1c_C	tRNA synthetases class I (E and Q), anti-codon binding domain	2	2	2	0
PF03951	Gln-synt_N	Glutamine synthetase, beta-Grasp domain	2	7	4	0
PF03952	Enolase_N	Enolase, N-terminal domain	2	4	3	0
PF03953	Tubulin_C	Tubulin C-terminal domain	87	29	16	38
PF03957	NA	Jun-like transcription factor	1	1	1	1
PF03959	NA	Serine hydrolase (FSH1)	1	2	1	0
PF03962	NA	Mnd1 family	1	1	1	2
PF03966	Trm112p	Trm112p-like protein	1	1	1	0
PF03969	NA	AFG1-like ATPase	1	1	2	1
PF03972	NA	MmgE/PrpD family	1	1	1	2
PF03979	Sigma70_r1_1	Sigma-70 factor, region 1.1	0	1	0	0
PF03980	Nnf1	Nnf1	0	2	1	0
PF03981	NA	Ubiquinol-cytochrome C chaperone	1	1	3	0
PF03982	NA	Diacylglycerol acyltransferase	1	4	1	1
PF03985	Paf1	Paf1	1	1	1	0
PF03986	Autophagy_N	Autophagocytosis associated protein (Atg3), N-terminal domain	1	1	1	0
PF03987	Autophagy_act_C	Autophagocytosis associated protein, active-site domain	2	3	3	0
PF03989	DNA_gyraseA_C	DNA gyrase C-terminal domain, beta-propeller	1	2	0	0
PF03992	ABM	Antibiotic biosynthesis monooxygenase	0	0	2	0
PF03997	VPS28	VPS28 protein	1	11	2	0
PF03998	Utp11	Utp11 protein	1	1	1	0
PF03999	MAP65_ASE1	Microtubule associated protein (MAP65/ASE1 family)	1	4	13	1
PF04000	Sas10_Utp3	Sas10/Utp3/C1D family	3	1	3	0
PF04003	Utp12	Dip2/Utp12 Family	3	3	7	1
PF04004	NA	Leo1-like protein	1	2	1	0
PF04005	Hus1	Hus1-like protein	1	3	1	0
PF04006	Mpp10	Mpp10 protein	1	1	1	2
PF04030	ALO	D-arabinono-1,4-lactone oxidase	2	2	2	2
PF04031	NA	Las1-like	1	1	2	0
PF04032	Rpr2	RNAse P Rpr2/Rpp21/SNM1 subunit domain	1	1	1	0
PF04034	NA	Domain of unknown function (DUF367)	1	1	3	0
PF04037	DUF382	Domain of unknown function (DUF382)	1	3	1	0
PF04042	DNA_pol_E_B	DNA polymerase alpha/epsilon subunit B	3	4	3	0
PF04045	P34-Arc	Arp2/3 complex, 34 kD subunit p34-Arc	1	1	2	1
PF04046	PSP	PSP	2	3	2	0
PF04048	Sec8_exocyst	Sec8 exocyst complex component specific domain	1	4	5	0
PF04049	ANAPC8	Anaphase promoting complex subunit 8 / Cdc23	1	2	1	0
PF04050	NA	Up-frameshift suppressor 2	1	3	1	0
PF04051	TRAPP	Transport protein particle (TRAPP) component	3	2	4	1
PF04053	Coatomer_WDAD	Coatomer WD associated region	2	3	4	2
PF04054	Not1	CCR4-Not complex component, Not1	1	3	4	3
PF04055	Radical_SAM	Radical SAM superfamily	10	19	11	1
PF04056	Ssl1	Ssl1-like	1	2	1	0
PF04057	Rep-A_N	Replication factor-A protein 1, N-terminal domain	1	1	2	0
PF04061	NA	ORMDL family	1	3	1	0
PF04062	P21-Arc	ARP2/3 complex ARPC3 (21 kDa) subunit	1	1	1	0
PF04063	DUF383	Domain of unknown function (DUF383)	1	1	2	0
PF04064	DUF384	Domain of unknown function (DUF384)	1	1	2	0
PF04065	NA	Not1 N-terminal domain, CCR4-Not complex component	1	2	3	0
PF04068	RLI	Possible Fer4-like domain in RNase L inhibitor, RLI	1	4	5	0

PF04072	LCM	Leucine carboxyl methyltransferase	2	2	2	0
PF04073	tRNA_edit	Aminoacyl-tRNA editing domain	2	0	2	1
PF04078	Rcd1	Cell differentiation family, Rcd1-like	1	2	1	0
PF04080	NA	Per1-like	1	2	1	0
PF04081	DNA_pol_delta_4	DNA polymerase delta, subunit 4	1	1	1	0
PF04083	NA	Partial alpha/beta-hydrolase lipase region	1	2	0	0
PF04084	ORC2	Origin recognition complex subunit 2	1	1	1	0
PF04086	SRP-alpha_N	Signal recognition particle, alpha subunit, N-terminal	1	1	1	0
PF04087	NA	Domain of unknown function (DUF389)	1	1	3	0
PF04088	NA	Peroxin 13, N-terminal region	1	2	2	1
PF04089	NA	BRICHOS domain	6	2	2	0
PF04091	Sec15	Exocyst complex subunit Sec15-like	1	3	2	0
PF04095	NAPRTase	Nicotinate phosphoribosyltransferase (NAPRTase) family	2	2	4	2
PF04096	Nucleoporin2	Nucleoporin autopeptidase	0	1	2	1
PF04097	Nic96	Nup93/Nic96	1	3	2	0
PF04098	NA	Rad52/22 family double-strand break repair protein	1	5	4	0
PF04099	NA	Sybindin-like family	2	2	3	0
PF04100	NA	Vps53-like, N-terminal	1	1	1	1
PF04101	Glyco_tran_28_C	Glycosyltransferase family 28 C-terminal domain	1	1	1	0
PF04103	NA	CD20-like family	1	2	5	0
PF04104	DNA_primase_lrg	Eukaryotic and archaeal DNA primase, large subunit	2	2	2	1
PF04106	APG5	Autophagy protein Apg5	1	2	2	0
PF04109	NA	Autophagy protein Apg9	1	1	3	0
PF04110	APG12	Ubiquitin-like autophagy protein Apg12	1	1	1	0
PF04111	NA	Autophagy protein Apg6	1	2	1	0
PF04112	NA	Mak10 subunit, NatC N(alpha)-terminal acetyltransferase	1	1	2	0
PF04113	NA	Gpi16 subunit, GPI transamidase component	1	1	1	2
PF04114	NA	Gaa1-like, GPI transamidase component	1	1	1	2
PF04116	NA	Fatty acid hydroxylase superfamily	8	10	17	6
PF04117	NA	Mpv17 / PMP22 family	3	11	15	1
PF04118	NA	Dopey, N-terminal	1	6	8	2
PF04121	Nup84_Nup100	Nuclear pore protein 84 / 107	1	1	1	0
PF04124	NA	Dor1-like family	2	1	1	0
PF04127	DFP	DNA / pantothenate metabolism flavoprotein	1	1	1	0
PF04129	NA	Vps52 / Sac2 family	3	5	1	0
PF04130	GCP_C_terminal	Spc97 / Spc98 family	6	6	13	3
PF04133	NA	Vacuolar protein sorting 55	1	1	1	0
PF04134	NA	Protein of unknown function, DUF393	1	0	1	0
PF04135	Nop10p	Nucleolar RNA-binding protein, Nop10p family	1	0	0	0
PF04136	NA	Sec34-like family	1	2	1	0
PF04137	ERO1	Endoplasmic Reticulum Oxidoreductin 1 (ERO1)	1	7	3	0
PF04139	Rad9	Rad9	1	1	2	0
PF04140	ICMT	Isoprenylcysteine carboxyl methyltransferase (ICMT) family	1	2	1	0
PF04142	Nuc_sug_transp	Nucleotide-sugar transporter	6	12	6	6
PF04143	NA	Sulphur transport	1	4	9	0
PF04144	NA	SCAMP family	1	5	7	0
PF04145	Ctr	Ctr copper transporter family	3	20	10	5
PF04146	YTH	YT521-B-like domain	3	5	6	2
PF04147	Nop14	Nop14-like family	1	3	1	0
PF04148	NA	Transmembrane adaptor Erv26	1	1	1	0
PF04152	Mre11_DNA_bind	Mre11 DNA-binding presumed domain	1	4	1	0
PF04153	NOT2_3_5	NOT2 / NOT3 / NOT5 family	3	3	6	0
PF04157	EAP30	EAP30/Vps36 family	2	10	3	0
PF04158	Sof1	Sof1-like domain	1	1	1	0
PF04161	NA	Arv1-like family	1	1	1	0
PF04176	TIP41	TIP41-like family	1	2	4	0
PF04177	NA	TAP42-like family	1	1	2	0
PF04178	NA	Got1/Sft2-like family	3	3	4	0
PF04180	LTV	Low temperature viability protein	1	3	1	1
PF04181	RPAP2_Rtr1	Rtr1/RPAP2 family	1	3	1	0
PF04182	NA	B-block binding subunit of TFIIIC	0	0	2	0
PF04184	NA	ST7 protein	2	7	3	1
PF04188	NA	Mannosyltransferase (PIG-V)	1	1	2	1
PF04189	Gcd10p	Gcd10p family	1	1	1	0
PF04190	DUF410	Protein of unknown function (DUF410)	1	1	1	0
PF04191	NA	Phospholipid methyltransferase	1	2	2	1
PF04192	Utp21	Utp21 specific WD40 associated putative domain	1	2	1	0
PF04193	PQ-loop	PQ loop repeat	5	19	14	2
PF04194	NA	Programmed cell death protein 2, C-terminal putative domain	1	2	3	1
PF04199	Cyclase	Putative cyclase	2	19	7	8
PF04201	NA	Tumour protein D52 family	1	1	12	1
PF04209	HgmA	homogentisate 1,2-dioxygenase	1	1	2	0
PF04212	MIT	MIT (microtubule interacting and transport) domain	5	14	11	2
PF04218	CENP-B_N	CENP-B N-terminal DNA-binding domain	3	7	3	2
PF04227	Indigoidine_A	Indigoidine synthase A like protein	2	1	1	0
PF04230	PS_pyruv_trans	Polysaccharide pyruvyl transferase	5	1	0	1
PF04241	NA	Protein of unknown function (DUF423)	1	2	2	0

PF04253	TFR_dimer	Transferrin receptor-like dimerisation domain	2	6	16	2
PF04258	NA	Signal peptide peptidase	4	9	4	0
PF04261	Dyp_perox	Dyp-type peroxidase family	1	5	2	2
PF04263	TPK_catalytic	Thiamin pyrophosphokinase, catalytic domain	1	2	1	0
PF04264	YceI	YceI-like domain	0	1	0	0
PF04265	TPK_B1_binding	Thiamin pyrophosphokinase, vitamin B1 binding domain	1	2	1	0
PF04266	ASCH	ASCH domain	1	2	1	0
PF04275	NA	Phosphomevalonate kinase	1	1	1	1
PF04280	Tim44	Tim44-like domain	2	3	2	0
PF04281	NA	Mitochondrial import receptor subunit Tom22	1	0	1	1
PF04286	NA	Protein of unknown function (DUF445)	0	2	0	0
PF04300	FBA	F-box associated region	1	0	6	0
PF04305	NA	Protein of unknown function (DUF455)	0	0	2	0
PF04321	RmlD_sub_bind	RmlD substrate binding domain	1	2	2	0
PF04324	Fer2_BFD	BFD-like [2Fe-2S] binding domain	0	0	2	0
PF04326	AlbA_2	Putative DNA-binding domain	3	6	3	0
PF04366	NA	Las17-binding protein actin regulator	1	1	0	1
PF04376	NA	Arginine-tRNA-protein transferase, N terminus	1	2	3	1
PF04377	NA	Arginine-tRNA-protein transferase, C terminus	1	2	3	0
PF04379	DUF525	Protein of unknown function (DUF525)	2	1	1	0
PF04387	NA	Protein tyrosine phosphatase-like protein, PTPLA	2	8	2	0
PF04388	Hamartin	Hamartin protein	1	1	8	0
PF04389	Peptidase_M28	Peptidase family M28	6	13	26	2
PF04397	LytTR	LytTr DNA-binding domain	0	0	1	0
PF04402	NA	Protein of unknown function (DUF541)	1	1	1	1
PF04406	TP6A_N	Type IIB DNA topoisomerase	1	5	1	0
PF04408	HA2	Helicase associated domain (HA2)	13	29	22	6
PF04410	Gar1	Gar1/Naf1 RNA binding region	2	2	4	0
PF04418	NA	Domain of unknown function (DUF543)	1	2	1	0
PF04419	NA	4F5 protein family	1	1	0	1
PF04420	CHD5	CHD5-like protein	1	1	1	0
PF04421	Mss4	Mss4 protein	1	1	1	0
PF04423	Rad50_zn_hook	Rad50 zinc hook motif	1	2	2	0
PF04424	MINDY_DUB	Protein of unknown function (DUF544)	1	6	2	0
PF04427	Brix	Brix domain	5	3	6	0
PF04430	DUF498	Protein of unknown function (DUF498/DUF598)	1	1	1	0
PF04433	SWIRM	SWIRM domain	5	8	14	0
PF04434	NA	SWIM zinc finger	2	4	14	0
PF04437	RINT1_TIP1	RINT-1 / TIP-1 family	1	1	1	0
PF04438	zf-HIT	HIT zinc finger	4	7	7	0
PF04442	CtaG_Cox11	Cytochrome c oxidase assembly protein CtaG/Cox11	1	3	1	0
PF04446	Thg1	tRNAHis guanylyltransferase	1	2	4	0
PF04457	NA	Protein of unknown function (DUF504)	2	3	1	0
PF04478	NA	Mid2 like cell wall stress sensor	0	1	1	0
PF04484	NA	Family of unknown function (DUF566)	1	0	1	0
PF04488	Gly_transf_sug	Glycosyltransferase sugar-binding region containing DXD motif	7	18	10	1
PF04493	Endonuclease_5	Endonuclease V	1	0	1	1
PF04494	TFIID_NTD2	WD40 associated region in TFIID subunit, NTD2 domain	2	2	2	0
PF04495	GRASP55_65	GRASP55/65 PDZ-like domain	1	0	2	0
PF04499	NA	SIT4 phosphatase-associated protein	1	5	5	0
PF04500	FLYWCH	FLYWCH zinc finger domain	4	4	14	1
PF04502	DUF572	Family of unknown function (DUF572)	2	6	1	0
PF04503	NA	Single-stranded DNA binding protein, SSDP	0	5	13	0
PF04505	NA	Interferon-induced transmembrane protein	20	21	43	4
PF04506	NA	Rft protein	1	1	2	0
PF04511	DER1	Der1-like family	3	2	4	0
PF04515	NA	Plasma-membrane choline transporter	6	6	16	6
PF04516	CP2	CP2 transcription factor	2	12	7	0
PF04519	Bactofilin	Polymer-forming cytoskeletal	0	1	1	0
PF04536	TPM_phosphatase	TLP18.3, Psb32 and MOLO-1 founding proteins of phosphatase	2	0	0	0
PF04539	Sigma70_r3	Sigma-70 region 3	0	1	1	0
PF04542	Sigma70_r2	Sigma-70 region 2	0	1	1	0
PF04545	Sigma70_r4	Sigma-70, region 4	0	1	1	0
PF04547	Anoctamin	Calcium-activated chloride channel	6	23	40	6
PF04548	AIG1	AIG1 family	23	147	46	80
PF04554	NA	Extensin-like region	1	0	0	0
PF04557	NA	Glutaminyl-tRNA synthetase, non-specific RNA binding region part 2	1	1	1	0
PF04558	NA	Glutaminyl-tRNA synthetase, non-specific RNA binding region part 1	1	1	2	0
PF04560	RNA_pol_Rpb2_7	RNA polymerase Rpb2, domain 7	3	6	3	0
PF04561	RNA_pol_Rpb2_2	RNA polymerase Rpb2, domain 2	2	6	5	1
PF04563	RNA_pol_Rpb2_1	RNA polymerase beta subunit	3	6	5	2
PF04564	U-box	U-box domain	5	16	8	1
PF04565	RNA_pol_Rpb2_3	RNA polymerase Rpb2, domain 3	3	5	5	1
PF04566	RNA_pol_Rpb2_4	RNA polymerase Rpb2, domain 4	2	3	4	0
PF04567	RNA_pol_Rpb2_5	RNA polymerase Rpb2, domain 5	2	3	4	0
PF04568	IATP	Mitochondrial ATPase inhibitor, IATP	1	2	0	1
PF04571	Lipin_N	lipin, N-terminal conserved region	1	1	3	0

PF04573	NA	Signal peptidase subunit	0	0	1	0
PF04577	NA	Protein of unknown function (DUF563)	7	6	8	3
PF04582	Reo_sigmaC	Reovirus sigma C capsid protein	0	0	0	1
PF04587	ADP_PFK_GK	ADP-specific Phosphofructokinase/Glucokinase conserved region	2	1	2	2
PF04588	HIG_1_N	Hypoxia induced protein conserved region	1	1	1	0
PF04592	NA	Selenoprotein P, N terminal region	1	4	1	2
PF04597	Ribophorin_I	Ribophorin I	1	1	2	2
PF04598	Gasdermin	Gasdermin family	1	0	0	1
PF04603	NA	Ran-interacting Mog1 protein	1	0	1	0
PF04614	NA	Pex19 protein family	1	3	1	0
PF04615	Utp14	Utp14 protein	1	2	1	3
PF04621	NA	PEA3 subfamily ETS-domain transcription factor N terminal domain	0	0	2	0
PF04622	ERG2_Sigma1R	ERG2 and Sigma1 receptor like protein	1	1	2	0
PF04628	Sedlin_N	Sedlin, N-terminal conserved region	1	3	12	0
PF04629	NA	Islet cell autoantigen ICA69, C-terminal domain	1	4	6	1
PF04636	NA	PA26 p53-induced protein (sestrin)	1	4	5	1
PF04641	NA	Rtf2 RING-finger	1	5	2	0
PF04652	Vta1	Vta1 like	1	1	1	0
PF04658	TAFII55_N	TAFII55 protein conserved region	1	1	2	0
PF04664	NA	Opioid growth factor receptor (OGFr) conserved region	1	8	1	3
PF04665	NA	Poxvirus A32 protein	20	0	0	0
PF04666	NA	N-Acetylglucosaminyltransferase-IV (GnT-IV) conserved region	3	5	16	3
PF04667	NA	cAMP-regulated phosphoprotein/endosulfine conserved region	1	1	1	0
PF04668	NA	Twisted gastrulation (Tsg) protein conserved region	1	1	1	2
PF04669	NA	Polysaccharide biosynthesis	1	1	1	0
PF04670	Gtr1_RagA	Gtr1/RagA G protein conserved region	2	2	2	1
PF04675	DNA_ligase_A_N	DNA ligase N terminus	3	5	10	0
PF04676	CwfJ_C_2	Protein similar to CwfJ C-terminus 2	2	2	2	0
PF04677	CwfJ_C_1	Protein similar to CwfJ C-terminus 1	2	2	2	0
PF04678	MCU	Mitochondrial calcium uniporter	2	1	1	2
PF04679	DNA_ligase_A_C	ATP dependent DNA ligase C terminal region	3	3	9	2
PF04683	Proteasom_Rpn13	Proteasome complex subunit Rpn13 ubiquitin receptor	1	1	1	0
PF04685	DUF608	Protein of unknown function, DUF608	1	1	3	2
PF04695	Pex14_N	Peroxisomal membrane anchor protein (Pex14p) conserved region	1	2	0	0
PF04696	NA	pinin/SDK/memA/ protein conserved region	1	1	1	0
PF04697	NA	pinin/SDK conserved region	1	1	1	0
PF04699	P16-Arc	ARP2/3 complex 16 kDa subunit (p16-Arc)	1	2	1	0
PF04707	PRELI	PRELI-like family	3	7	4	0
PF04710	Pellino	Pellino	1	1	2	0
PF04712	NA	Radial spokehead-like protein	2	2	3	6
PF04714	NA	BCL7, N-terminal conserver region	1	1	1	0
PF04716	ETC_C1_NDUFA5	ETC complex I subunit conserved region	1	3	1	0
PF04718	NA	Mitochondrial ATP synthase g subunit	1	1	1	0
PF04719	TAFII28	hTAFII28-like protein conserved region	1	4	2	1
PF04721	PAW	PNGase C-terminal domain, mannose-binding module PAW	1	1	2	1
PF04722	Ssu72	Ssu72-like protein	1	1	1	0
PF04727	ELMO_CED12	ELMO/CED-12 family	4	8	12	0
PF04729	ASF1_hist_chap	ASF1 like histone chaperone	1	1	1	0
PF04733	Coatomer_E	Coatomer epsilon subunit	1	1	1	0
PF04734	Ceramidase_alk	Neutral/alkaline non-lysosomal ceramidase, N-terminal	1	2	1	0
PF04739	AMPKBI	5'-AMP-activated protein kinase beta subunit, interaction domain	1	7	2	0
PF04749	NA	PLAC8 family	8	10	16	3
PF04750	NA	FAR-17a/AIG1-like protein	1	1	4	1
PF04752	ChaC	ChaC-like protein	2	7	4	1
PF04756	OST3_OST6	OST3 / OST6 family	2	2	2	0
PF04757	NA	Pex2 / Pex12 amino terminal region	3	4	4	0
PF04758	Ribosomal_S30	Ribosomal protein S30	1	2	2	0
PF04762	IKI3	IKI3 family	2	1	1	0
PF04768	NAT	NAT, N-acetyltransferase, of N-acetylglutamate synthase	1	2	0	0
PF04774	HABP4_PAI-RBP1	Hyaluronan / mRNA binding family	1	1	2	1
PF04775	Bile_Hydr_Trans	Acyl-CoA thioester hydrolase/BAAT N-terminal region	1	3	6	2
PF04777	Evr1_Alr	Erv1 / Alr family	1	6	2	1
PF04784	NA	Protein of unknown function, DUF547	3	6	4	2
PF04790	NA	Sarcoglycan complex subunit protein	2	2	4	1
PF04791	NA	LMBR1-like membrane protein	3	3	4	4
PF04794	YdjC	YdjC-like protein	1	5	1	0
PF04795	NA	PAPA-1-like conserved region	1	1	1	0
PF04799	Fzo_mitofusin	fzo-like conserved region	1	1	2	1
PF04800	ETC_C1_NDUFA4	ETC complex I subunit conserved region	1	1	2	0
PF04801	Sin_N	Sin-like protein conserved region	1	1	2	1
PF04802	NA	Component of IIS longevity pathway SMK-1	1	1	1	0
PF04803	Cor1	Cor1/Xlr/Xmr conserved region	1	5	3	0
PF04810	zf-Sec23_Sec24	Sec23/Sec24 zinc finger	4	7	15	0
PF04811	Sec23_trunk	Sec23/Sec24 trunk domain	3	5	11	1
PF04814	NA	Hepatocyte nuclear factor 1 (HNF-1), N terminus	0	1	0	1
PF04815	Sec23_helical	Sec23/Sec24 helical domain	3	5	11	2
PF04818	CTD_bind	RNA polymerase II-binding domain.	6	8	8	0

PF04819	NA	Family of unknown function (DUF716)	2	2	5	2
PF04821	TIMELESS	Timeless protein	1	3	3	0
PF04824	Rad21_Rec8	Conserved region of Rad21 / Rec8 like protein	2	3	2	1
PF04825	Rad21_Rec8_N	N terminus of Rad21 / Rec8 like protein	2	2	1	1
PF04826	NA	Armadillo-like	1	2	1	0
PF04828	GFA	Glutathione-dependent formaldehyde-activating enzyme	1	2	4	0
PF04831	NA	Popeye protein conserved region	0	4	1	2
PF04832	SOUL	SOUL heme-binding protein	9	0	12	4
PF04836	NA	Interferon-related protein conserved region	1	1	2	0
PF04840	Vps16_C	Vps16, C-terminal region	1	1	2	0
PF04841	NA	Vps16, N-terminal region	1	2	2	0
PF04843	NA	Herpesvirus tegument protein, N-terminal conserved region	0	0	8	0
PF04845	NA	PurA ssDNA and RNA-binding protein	1	7	6	0
PF04847	Calcipressin	Calcipressin	1	2	1	0
PF04849	HAP1_N	HAP1 N-terminal conserved region	2	1	8	1
PF04851	ResIII	Type III restriction enzyme, res subunit	4	5	13	0
PF04852	NA	Protein of unknown function (DUF640)	3	0	0	0
PF04854	NA	Protein of unknown function, DUF624	0	0	1	0
PF04855	SNF5	SNF5 / SMARCB1 / INI1	0	1	1	0
PF04857	CAF1	CAF1 family ribonuclease	4	3	8	2
PF04858	TH1	TH1 protein	1	3	1	1
PF04863	EGF_alliinase	Alliinase EGF-like domain	0	0	1	0
PF04869	Uso1_p115_head	Uso1 / p115 like vesicle tethering protein, head region	1	2	1	0
PF04871	NA	Uso1 / p115 like vesicle tethering protein, C terminal region	0	2	1	1
PF04874	Mak16	Mak16 protein C-terminal region	1	1	1	0
PF04879	Molybdop_Fe4S4	Molybdopterin oxidoreductase Fe4S4 domain	0	0	1	0
PF04880	NA	NUDE protein, C-terminal conserved region	1	8	1	0
PF04882	Peroxin-3	Peroxin-3	1	1	2	0
PF04884	NA	Vitamin B6 photo-protection and homoeostasis	1	1	1	0
PF04886	NA	PT repeat	0	1	0	0
PF04889	Cwf_Cwc_15	Cwf15/Cwc15 cell cycle control protein	1	1	4	0
PF04893	NA	Yip1 domain	5	4	4	0
PF04898	Glu_syn_central	Glutamate synthase central domain	1	2	4	0
PF04900	Fcf1	Fcf1	2	2	3	0
PF04904	NA	NAB conserved region 1 (NCD1)	1	4	7	1
PF04905	NA	NAB conserved region 2 (NCD2)	1	4	7	0
PF04906	NA	Tweety	0	2	1	0
PF04908	SH3BGR	SH3-binding, glutamic acid-rich protein	1	2	30	1
PF04909	Amidohydro_2	Amidohydrolase	1	2	2	0
PF04910	NA	Transcriptional repressor TCF25	1	1	2	0
PF04912	Dynamitin	Dynamitin	2	1	3	0
PF04916	Phospholip_B	Phospholipase B	5	2	7	6
PF04921	NA	XAP5, circadian clock regulator	1	3	1	1
PF04922	NA	DIE2/ALG10 family	1	3	3	0
PF04923	NA	Ninjurin	1	1	4	4
PF04925	SHQ1	SHQ1 protein	1	1	4	1
PF04926	PAP_RNA-bind	Poly(A) polymerase predicted RNA binding domain	1	1	7	0
PF04928	PAP_central	Poly(A) polymerase central domain	1	1	7	0
PF04930	NA	FUN14 family	1	1	5	0
PF04931	NA	DNA polymerase phi	1	3	1	0
PF04934	Med6	MED6 mediator sub complex component	1	1	2	0
PF04935	NA	Surfeit locus protein 6	1	8	1	1
PF04937	NA	Protein of unknown function (DUF 659)	0	6	3	0
PF04938	SIP1	Survival motor neuron (SMN) interacting protein 1 (SIP1)	1	1	1	1
PF04939	RRS1	Ribosome biogenesis regulatory protein (RRS1)	1	1	1	0
PF04940	BLUF	Sensors of blue-light using FAD	0	0	2	0
PF04950	RIBIOP_C	40S ribosome biogenesis protein Tsr1 and BMS1 C-terminal	2	3	2	2
PF04959	ARS2	Arsenite-resistance protein 2	1	5	5	0
PF04960	Glutaminase	Glutaminase	1	1	2	1
PF04961	FTCD_C	Formiminotransferase-cyclodeaminase	1	1	3	1
PF04968	CHORD	CHORD	1	1	1	0
PF04969	CS	CS domain	14	15	24	1
PF04970	LRAT	Lecithin retinol acyltransferase	2	18	16	14
PF04979	IPP-2	Protein phosphatase inhibitor 2 (IPP-2)	1	2	1	2
PF04981	NMD3	NMD3 family	1	1	1	0
PF04983	RNA_pol_Rpb1_3	RNA polymerase Rpb1, domain 3	3	5	3	4
PF04987	NA	Phosphatidylinositolglycan class N (PIG-N)	1	3	1	0
PF04990	RNA_pol_Rpb1_7	RNA polymerase Rpb1, domain 7	1	1	1	1
PF04991	LicD	LicD family	8	24	2	18
PF04992	RNA_pol_Rpb1_6	RNA polymerase Rpb1, domain 6	1	1	1	3
PF04997	RNA_pol_Rpb1_1	RNA polymerase Rpb1, domain 1	3	5	3	3
PF04998	RNA_pol_Rpb1_5	RNA polymerase Rpb1, domain 5	3	5	3	5
PF05000	RNA_pol_Rpb1_4	RNA polymerase Rpb1, domain 4	3	4	3	1
PF05001	RNA_pol_Rpb1_R	RNA polymerase Rpb1 C-terminal repeat	0	2	1	1
PF05002	NA	SGS domain	2	2	1	0
PF05004	NA	Interferon-related developmental regulator (IFRD)	1	1	3	1
PF05005	Ocnus	Janus/Ocnus family (Ocnus)	1	1	1	1

PF05007	NA	Mannosyltransferase (PIG-M)	1	1	1	1
PF05008	V-SNARE	Vesicle transport v-SNARE protein N-terminus	1	1	2	0
PF05010	NA	Transforming acidic coiled-coil-containing protein (TACC)	1	3	7	0
PF05011	NA	Lariat debranching enzyme, C-terminal domain	2	1	2	0
PF05014	Nuc_deoxyrib_tr	Nucleoside 2-deoxyribosyltransferase	0	1	5	0
PF05018	DUF667	Protein of unknown function (DUF667)	3	2	3	1
PF05019	NA	Coenzyme Q (ubiquinone) biosynthesis protein Coq4	1	7	1	0
PF05020	zf-NPL4	NPL4 family, putative zinc binding region	1	1	1	0
PF05021	NPL4	NPL4 family	1	1	1	1
PF05022	NA	SRP40, C-terminal domain	1	1	2	0
PF05023	Phytochelatin	Phytochelatin synthase	3	1	2	0
PF05024	NA	N-acetylglucosaminyl transferase component (Gpi1)	1	1	1	0
PF05025	RbsD_FucU	RbsD / FucU transport protein family	1	5	2	0
PF05026	DCP2	Dcp2, box A domain	1	2	1	0
PF05028	PARG_cat	Poly (ADP-ribose) glycohydrolase (PARG)	2	2	4	0
PF05029	TIMELESS_C	Timeless protein C terminal region	1	1	1	0
PF05030	NA	SSXT protein (N-terminal region)	1	0	2	0
PF05033	Pre-SET	Pre-SET motif	3	3	10	1
PF05038	NA	Cytochrome Cytochrome b558 alpha-subunit	1	1	2	0
PF05041	NA	Pecanex protein (C-terminus)	2	9	3	3
PF05044	HPD	Homeo-prospero domain	1	1	3	1
PF05046	Img2	Mitochondrial large subunit ribosomal protein (Img2)	1	1	0	0
PF05047	L51_S25_CI-B8	Mitochondrial ribosomal protein L51 / S25 / CI-B8 domain	2	3	2	0
PF05048	NosD	Periplasmic copper-binding protein (NosD)	1	0	2	0
PF05049	IIGP	Interferon-inducible GTPase (IIGP)	0	0	4	0
PF05050	NA	Methyltransferase FkbM domain	12	6	25	15
PF05051	COX17	Cytochrome C oxidase copper chaperone (COX17)	1	0	0	0
PF05053	Menin	Menin	1	1	1	2
PF05057	NA	Putative serine esterase (DUF676)	1	1	7	0
PF05060	MGAT2	N-acetylglucosaminyltransferase II (MGAT2)	2	1	2	3
PF05063	MT-A70	MT-A70	2	4	3	0
PF05064	Nsp1_C	Nsp1-like C-terminal region	1	1	2	0
PF05071	NDUFA12	NADH ubiquinone oxidoreductase subunit NDUFA12	2	4	3	0
PF05076	SUFU	Suppressor of fused protein (SUFU)	2	4	2	0
PF05088	NA	Bacterial NAD-glutamate dehydrogenase	0	1	0	0
PF05089	NAGLU	Alpha-N-acetylglucosaminidase (NAGLU) tim-barrel domain	2	4	4	0
PF05090	NA	Vitamin K-dependent gamma-carboxylase	1	3	4	1
PF05091	eIF-3_zeta	Eukaryotic translation initiation factor 3 subunit 7 (eIF-3)	1	2	2	2
PF05093	NA	Cytokine-induced anti-apoptosis inhibitor 1, Fe-S biogenesis	1	2	1	0
PF05110	AF-4	AF-4 proto-oncoprotein	1	1	3	0
PF05118	Asp_Arg_Hydrox	Aspartyl/Asparaginyl beta-hydroxylase	2	2	2	2
PF05127	Helicase_RecD	Helicase	1	4	1	0
PF05129	Elf1	Transcription elongation factor Elf1 like	0	1	0	0
PF05131	Pep3_Vps18	Pep3/Vps18/deep orange family	1	1	1	0
PF05132	RNA_pol_Rpc4	RNA polymerase III RPC4	1	1	1	0
PF05147	LANC_like	Lanthionine synthetase C-like protein	3	4	3	3
PF05148	NA	Hypothetical methyltransferase	1	1	1	0
PF05153	MIOX	Myo-inositol oxygenase	1	2	1	2
PF05154	NA	TM2 domain	5	12	14	4
PF05158	RNA_pol_Rpc34	RNA polymerase Rpc34 subunit	1	1	2	0
PF05160	DSS1_SEM1	DSS1/SEM1 family	0	0	1	0
PF05161	NA	MOFRL family	1	9	2	1
PF05168	HEPN	HEPN domain	1	0	5	3
PF05172	Nup35_RRM	Nup53/35/40-type RNA recognition motif	1	1	1	0
PF05175	MTS	Methyltransferase small domain	0	1	0	0
PF05178	NA	KRI1-like family	1	1	2	1
PF05179	NA	RNA pol II accessory factor, Cdc73 family, C-terminal	1	1	1	0
PF05180	NA	DNL zinc finger	1	1	1	0
PF05181	XPA_C	XPA protein C-terminus	1	1	2	0
PF05182	NA	Fip1 motif	1	3	2	0
PF05183	RdRP	RNA dependent RNA polymerase	0	0	3	0
PF05184	SapB_1	Saposin-like type B, region 1	8	9	5	3
PF05185	PRMT5	PRMT5 arginine-N-methyltransferase	4	4	7	0
PF05186	Dpy-30	Dpy-30 motif	4	7	6	1
PF05187	ETF_QO	Electron transfer flavoprotein-ubiquinone oxidoreductase, 4Fe-4S	1	1	1	0
PF05188	MutS_II	MutS domain II	5	5	5	0
PF05189	RTC_insert	RNA 3'-terminal phosphate cyclase (RTC), insert domain	2	4	2	0
PF05190	MutS_IV	MutS family domain IV	5	5	10	2
PF05191	ADK_lid	Adenylate kinase, active site lid	3	2	2	0
PF05192	MutS_III	MutS domain III	5	6	10	1
PF05193	Peptidase_M16_C	Peptidase M16 inactive domain	6	15	8	1
PF05195	AMP_N	Aminopeptidase P, N-terminal domain	2	5	1	1
PF05197	TRIC	TRIC channel	1	5	1	2
PF05199	GMC_oxred_C	GMC oxidoreductase	2	22	25	5
PF05201	GlutR_N	Glutamyl-tRNAGlu reductase, N-terminal domain	0	0	1	0
PF05205	NA	COMPASS (Complex proteins associated with Set1p) component shg1	1	1	6	0
PF05206	NA	Methyltransferase TRM13	1	5	1	1

PF05207	zf-CSL	CSL zinc finger	1	1	1	1
PF05208	NA	ALG3 protein	1	1	1	0
PF05210	NA	Sprouty protein (Spry)	2	6	3	2
PF05216	NA	UNC-50 family	1	1	1	0
PF05217	NA	STOP protein	5	2	5	0
PF05219	NA	DREV methyltransferase	1	1	1	0
PF05221	AdoHcyase	S-adenosyl-L-homocysteine hydrolase	2	7	9	3
PF05222	AlaDh_PNT_N	Alanine dehydrogenase/PNT, N-terminal domain	2	3	1	0
PF05224	NDT80_PhoG	NDT80 / PhoG like DNA-binding family	1	2	8	1
PF05225	HTH_psq	helix-turn-helix, Psq domain	4	22	13	7
PF05236	TAF4	Transcription initiation factor TFIID component TAF4 family	1	1	2	0
PF05238	CENP-N	Kinetochore protein CHL4 like	0	1	1	0
PF05241	EBP	Emopamil binding protein	2	4	2	3
PF05250	NA	Uncharacterised protein family (UPF0193)	1	1	1	0
PF05251	Ost5	Uncharacterised protein family (UPF0197)	1	0	0	0
PF05253	zf-U11-48K	U11-48K-like CHHC zinc finger	5	9	8	0
PF05254	UPF0203	Uncharacterised protein family (UPF0203)	1	0	0	0
PF05255	NA	Uncharacterised protein family (UPF0220)	1	1	1	0
PF05257	CHAP	CHAP domain	0	0	2	0
PF05276	SH3BP5	SH3 domain-binding protein 5 (SH3BP5)	1	5	3	3
PF05277	NA	Protein of unknown function (DUF726)	1	5	2	2
PF05281	NA	Neuroendocrine protein 7B2 precursor (Secretogranin V)	1	1	2	1
PF05282	AAR2	AAR2 protein	1	1	1	1
PF05283	NA	Multi-glycosylated core protein 24 (MGC-24), sialomucin	2	2	2	1
PF05285	NA	SDA1	1	1	1	0
PF05291	Bystin	Bystin	1	1	1	1
PF05292	MCD	Malonyl-CoA decarboxylase (MCD)	1	3	4	2
PF05296	NA	Taste receptor protein (TAS2R)	0	0	0	1
PF05300	NA	Protein of unknown function (DUF737)	1	0	0	0
PF05301	Acetyltransf_16	Touch receptor neuron protein Mec-17	2	1	2	0
PF05303	DUF727	Protein of unknown function (DUF727)	1	0	2	0
PF05308	NA	Mitochondrial fission regulator	0	1	1	1
PF05327	RRN3	RNA polymerase I specific transcription initiation factor RRN3	1	1	2	0
PF05328	CybS	CybS, succinate dehydrogenase cytochrome B small subunit	1	1	1	0
PF05334	NA	Protein of unknown function (DUF719)	1	3	2	1
PF05345	NA	Putative Ig domain	0	0	2	0
PF05346	NA	Eukaryotic membrane protein family	1	3	1	1
PF05347	Complex1_LYR	Complex 1 protein (LYR family)	7	7	2	0
PF05348	NA	Proteasome maturation factor UMP1	1	1	1	0
PF05349	NA	GATA-type transcription activator, N-terminal	0	0	4	1
PF05350	GSK-3_bind	Glycogen synthase kinase-3 binding	1	0	0	0
PF05351	GMP_PDE_delta	GMP-PDE, delta subunit	2	2	3	0
PF05361	PP1_inhibitor	PKC-activated protein phosphatase-1 inhibitor	0	2	1	0
PF05362	Lon_C	Lon protease (S16) C-terminal proteolytic domain	2	4	4	4
PF05365	UCR_UQCRX_QCR9	Ubiquinol-cytochrome C reductase, UQCRX/QCR9 like	1	0	0	0
PF05368	NmrA	NmrA-like family	2	3	12	1
PF05375	Pacifastin_I	Pacifastin inhibitor (LCMII)	7	0	8	2
PF05378	Hydant_A_N	Hydantoinase/oxoprolinase N-terminal region	2	6	3	2
PF05380	NA	Pao retrotransposon peptidase	3	0	69	0
PF05383	La	La domain	5	13	10	1
PF05404	NA	Translocon-associated protein, delta subunit precursor (TRAP-delta)	1	1	2	0
PF05405	Mt_ATP-synt_B	Mitochondrial ATP synthase B chain precursor (ATP-synt_B)	1	1	1	0
PF05406	WGR	WGR domain	3	8	7	2
PF05422	NA	Stress-activated map kinase interacting protein 1 (SIN1)	1	2	1	0
PF05427	NA	Acidic fibroblast growth factor binding (FIBP)	0	5	1	0
PF05428	NA	Corticotropin-releasing factor binding protein (CRF-BP)	1	0	1	1
PF05434	NA	TMEM9	1	4	2	0
PF05439	NA	Jumping translocation breakpoint protein (JTB)	1	0	2	0
PF05450	Nicastrin	Nicastrin	1	1	2	1
PF05454	NA	Dystroglycan (Dystrophin-associated glycoprotein 1)	1	2	6	1
PF05456	eIF_4EBP	Eukaryotic translation initiation factor 4E binding protein (EIF4EBP)	1	1	1	0
PF05458	NA	Cd27 binding protein (Siva)	1	2	1	0
PF05460	ORC6	Origin recognition complex subunit 6 (ORC6)	0	0	1	0
PF05461	NA	Apolipoprotein L	4	0	13	0
PF05462	NA	Slime mold cyclic AMP receptor	1	3	3	1
PF05463	Sclerostin	Sclerostin (SOST)	1	2	1	1
PF05470	eIF-3c_N	Eukaryotic translation initiation factor 3 subunit 8 N-terminus	1	1	2	3
PF05473	NA	UL45 protein	0	0	1	0
PF05477	NA	Surfeit locus protein 2 (SURF2)	1	1	1	0
PF05478	NA	Prominin	3	8	8	1
PF05482	NA	Serendipity locus alpha protein (SRY-A)	0	0	1	0
PF05483	SCP-1	Synaptonemal complex protein 1 (SCP-1)	0	2	3	0
PF05485	THAP	THAP domain	16	28	72	4
PF05486	SRP9-21	Signal recognition particle 9 kDa protein (SRP9)	1	1	1	0
PF05493	ATP_synt_H	ATP synthase subunit H	1	1	1	0
PF05495	NA	CHY zinc finger	2	2	3	0
PF05496	RuvB_N	Holliday junction DNA helicase ruvB N-terminus	0	1	0	0

PF05497	Destabilase	Destabilase	0	1	5	0
PF05499	NA	DNA methyltransferase 1-associated protein 1 (DMAP1)	1	1	1	1
PF05502	Dynactin_p62	Dynactin p62 family	1	1	1	0
PF05510	NA	Sarcoglycan alpha/epsilon	0	1	0	0
PF05511	ATP-synt_F6	Mitochondrial ATP synthase coupling factor 6	1	1	0	0
PF05517	NA	p25-alpha	1	1	3	2
PF05524	PEP-utilisers_N	PEP-utilising enzyme, N-terminal	0	1	0	0
PF05527	DUF758	Domain of unknown function (DUF758)	1	6	2	1
PF05529	NA	B-cell receptor-associated protein 31-like	1	1	3	0
PF05536	NA	Neurochondrin	1	1	2	1
PF05544	Pro_racemase	Proline racemase	2	2	5	1
PF05551	zf-His_Me_endon	Zinc-binding loop region of homing endonuclease	0	0	0	2
PF05556	NA	Calcineurin-binding protein (Calsarcin)	1	0	0	0
PF05557	MAD	Mitotic checkpoint protein	1	3	1	2
PF05571	NA	Protein of unknown function (DUF766)	1	2	1	1
PF05577	NA	Serine carboxypeptidase S28	3	9	6	2
PF05585	NA	Putative peptidase (DUF1758)	1	0	5	0
PF05600	NA	Protein of unknown function (DUF773)	1	1	2	2
PF05602	NA	Cleft lip and palate transmembrane protein 1 (CLPTM1)	2	4	4	0
PF05603	NA	Protein of unknown function (DUF775)	1	1	1	0
PF05605	NA	Drought induced 19 protein (Di19), zinc-binding	7	1	12	2
PF05608	NA	Protein of unknown function (DUF778)	1	1	1	0
PF05609	LAP1C	Lamina-associated polypeptide 1C (LAP1C)	1	0	2	1
PF05615	NA	Tho complex subunit 7	1	2	1	0
PF05620	NA	Protein of unknown function (DUF788)	1	2	1	0
PF05622	HOOK	HOOK protein	2	4	8	1
PF05625	PAXNEB	PAXNEB protein	1	2	1	0
PF05631	NA	Sugar-tranasporters, 12 TM	2	4	9	1
PF05640	NA	Na,K-Atpase Interacting protein	1	1	5	0
PF05641	Agenet	Agenet domain	0	0	3	0
PF05644	NA	Mitochondrial and peroxisomal fission factor Mff	1	2	1	1
PF05645	RNA_pol_Rpc82	RNA polymerase III subunit RPC82	1	2	1	0
PF05648	NA	Peroxisomal biogenesis factor 11 (PEX11)	3	5	2	2
PF05649	Peptidase_M13_N	Peptidase family M13	17	44	26	11
PF05652	DcpS	Scavenger mRNA decapping enzyme (DcpS) N-terminal	1	0	1	1
PF05653	NA	Magnesium transporter NIPA	1	7	7	3
PF05667	NA	Protein of unknown function (DUF812)	0	2	1	0
PF05669	Med31	SOH1	1	2	1	0
PF05670	NA	Domain of unknown function (DUF814)	2	2	4	0
PF05672	NA	MAP7 (E-MAP-115) family	1	17	23	0
PF05676	NDUF_B7	NADH-ubiquinone oxidoreductase B18 subunit (NDUFB7)	1	2	1	0
PF05679	NA	Chondroitin N-acetylgalactosaminyltransferase	7	7	13	13
PF05680	NA	ATP synthase E chain	0	1	1	0
PF05681	Fumerase	Fumarate hydratase (Fumerase)	1	2	1	0
PF05683	Fumerase_C	Fumarase C-terminus	1	2	1	1
PF05686	Glyco_transf_90	Glycosyl transferase family 90	2	2	2	0
PF05693	Glycogen_syn	Glycogen synthase	1	2	2	2
PF05694	NA	56kDa selenium binding protein (SBP56)	2	3	2	5
PF05697	Trigger_N	Bacterial trigger factor protein (TF)	0	1	0	0
PF05699	Dimer_Tnp_hAT	hAT family C-terminal dimerisation region	3	3	23	25
PF05700	BCAS2	Breast carcinoma amplified sequence 2 (BCAS2)	1	0	1	2
PF05701	NA	Weak chloroplast movement under blue light	0	5	0	0
PF05704	NA	Capsular polysaccharide synthesis protein	0	0	0	1
PF05705	NA	Eukaryotic protein of unknown function (DUF829)	4	5	3	8
PF05706	CDKN3	Cyclin-dependent kinase inhibitor 3 (CDKN3)	1	1	1	0
PF05712	MRG	MRG	2	2	5	0
PF05719	GPP34	Golgi phosphoprotein 3 (GPP34)	1	1	1	1
PF05721	PhyH	Phytanoyl-CoA dioxygenase (PhyH)	13	12	42	1
PF05724	TPMT	Thiopurine S-methyltransferase (TPMT)	4	5	6	0
PF05726	Pirin_C	Pirin C-terminal cupin domain	2	3	4	0
PF05729	NACHT	NACHT domain	11	12	23	2
PF05731	NA	TROVE domain	2	2	8	1
PF05735	TSP_C	Thrombospondin C-terminal region	1	1	4	0
PF05739	SNARE	SNARE domain	5	8	18	1
PF05741	zf-nanos	Nanos RNA binding domain	1	7	5	2
PF05742	NA	Transport and Golgi organisation 2	1	1	2	0
PF05743	UEV	UEV domain	1	1	3	0
PF05746	DALR_1	DALR anticodon binding domain	3	3	5	1
PF05753	NA	Translocon-associated protein beta (TRAPB)	1	1	1	0
PF05760	NA	Immediate early response protein (IER)	1	1	1	1
PF05761	5_nucleotid	5' nucleotidase family	5	8	10	3
PF05764	YL1	YL1 nuclear protein	1	3	1	1
PF05768	NA	Glutaredoxin-like domain (DUF836)	1	3	1	0
PF05769	SIKE	Protein of unknown function (DUF837)	1	2	2	1
PF05770	Ins134_P3_kin	Inositol 1, 3, 4-trisphosphate 5/6-kinase	1	1	1	0
PF05773	RWD	RWD domain	10	14	19	1
PF05781	NA	MRVI1 protein	1	8	1	0

PF05783	DLIC	Dynein light intermediate chain (DLIC)	2	3	6	2
PF05786	Cnd2	Condensin complex subunit 2	1	1	1	0
PF05791	Bacillus_HBL	Bacillus haemolytic enterotoxin (HBL)	0	1	0	0
PF05793	TFIIF_alpha	Transcription initiation factor IIF, alpha subunit (TFIIF-alpha)	0	1	1	0
PF05794	NA	T-complex protein 11	1	2	2	1
PF05804	NA	Kinesin-associated protein (KAP)	1	1	2	1
PF05806	NA	Noggin	2	5	2	2
PF05811	NA	Eukaryotic protein of unknown function (DUF842)	1	2	2	0
PF05817	Ribophorin_II	Oligosaccharyltransferase subunit Ribophorin II	1	1	2	0
PF05821	NDUF_B8	NADH-ubiquinone oxidoreductase ASHI subunit (CI-ASHI or NDUFB8)	1	2	1	0
PF05822	UMPH-1	Pyrimidine 5'-nucleotidase (UMPH-1)	1	2	2	1
PF05825	PSP94	Beta-microseminoprotein (PSP-94)	2	0	0	0
PF05826	Phospholip_A2_2	Phospholipase A2	10	3	6	0
PF05827	NA	Vacuolar ATP synthase subunit S1 (ATP6S1)	0	0	0	1
PF05832	NA	Eukaryotic protein of unknown function (DUF846)	1	1	1	0
PF05833	FbpA	Fibronectin-binding protein A N-terminus (FbpA)	1	1	2	0
PF05835	Synaphin	Synaphin protein	1	1	10	0
PF05837	CENP-H	Centromere protein H (CENP-H)	0	1	1	0
PF05839	Apc13p	Apc13p protein	1	0	1	1
PF05843	Suf	Suppressor of forked protein (Suf)	2	4	2	0
PF05856	ARPC4	ARP2/3 complex 20 kDa subunit (ARPC4)	1	2	1	0
PF05859	Mis12	Mis12 protein	1	1	1	0
PF05871	ESCRT-II	ESCRT-II complex subunit	1	1	1	0
PF05873	Mt_ATP-synt_D	ATP synthase D chain, mitochondrial (ATP5H)	1	2	0	0
PF05875	Ceramidase	Ceramidase	0	3	1	0
PF05881	CNPase	2',3'-cyclic nucleotide 3'-phosphodiesterase (CNP or CNPase)	1	1	3	1
PF05889	SepSecS	O-phosphoseryl-tRNA(Sec) selenium transferase, SepSecS	1	1	3	0
PF05890	Ebp2	Eukaryotic rRNA processing protein EBP2	1	1	1	0
PF05891	Methyltransf_PK	AdoMet dependent proline di-methyltransferase	1	1	1	2
PF05902	NA	4.1 protein C-terminal domain (CTD)	0	4	5	0
PF05903	NA	PPPDE putative peptidase domain	2	5	2	0
PF05907	DUF866	Eukaryotic protein of unknown function (DUF866)	1	1	1	0
PF05914	RIB43A	RIB43A	1	1	1	2
PF05915	NA	Eukaryotic protein of unknown function (DUF872)	2	2	3	0
PF05916	Sld5	GINS complex protein	3	5	4	1
PF05918	NA	Apoptosis inhibitory protein 5 (API5)	1	1	1	1
PF05920	Homeobox_KN	Homeobox KN domain	9	11	20	3
PF05923	APC_r	APC cysteine-rich region	0	2	3	2
PF05924	NA	SAMP Motif	0	0	3	1
PF05934	NA	Mid-1-related chloride channel (MCLC)	2	3	4	0
PF05958	tRNA_U5-meth_tr	tRNA (Uracil-5-)-methyltransferase	1	0	1	0
PF05960	DUF885	Bacterial protein of unknown function (DUF885)	5	10	18	5
PF05964	FYRN	F/Y-rich N-terminus	1	3	5	1
PF05965	FYRC	F/Y rich C-terminus	3	3	6	0
PF05966	Chordopox_A33R	Chordopoxvirus A33R protein	0	0	1	0
PF05970	PIF1	PIF1-like helicase	0	9	16	2
PF05971	Methyltransf_10	Protein of unknown function (DUF890)	1	1	2	0
PF05972	NA	APC 15 residue motif	0	0	0	1
PF05978	NA	Ion channel regulatory protein UNC-93	7	11	12	7
PF05983	Med7	MED7 protein	1	4	2	1
PF05986	NA	ADAM-TS Spacer 1	9	18	27	3
PF05994	NA	Cytoplasmic Fragile-X interacting family	1	7	4	4
PF05995	CDO_I	Cysteine dioxygenase type I	1	17	3	1
PF05997	Nop52	Nucleolar protein,Nop52	1	0	1	0
PF06001	DUF902	Domain of Unknown Function (DUF902)	1	1	4	0
PF06003	SMN	Survival motor neuron protein (SMN)	2	3	4	1
PF06008	NA	Laminin Domain I	1	2	4	0
PF06009	NA	Laminin Domain II	0	0	1	0
PF06012	NA	Domain of Unknown Function (DUF908)	1	2	4	0
PF06017	Myosin_TH1	Unconventional myosin tail, actin- and lipid-binding	4	4	26	0
PF06021	NA	Aralkyl acyl-CoA:amino acid N-acyltransferase	5	4	4	0
PF06025	NA	Domain of Unknown Function (DUF913)	1	1	4	0
PF06026	Rib_5-P_isom_A	Ribose 5-phosphate isomerase A (phosphoriboisomerase A)	1	1	1	1
PF06027	NA	Solute carrier family 35	1	3	1	0
PF06031	NA	SERTA motif	0	0	7	2
PF06032	NA	Protein of unknown function (DUF917)	1	5	1	2
PF06046	Sec6	Exocyst complex component Sec6	1	1	3	0
PF06047	NA	Ras-induced vulval development antagonist	1	1	1	0
PF06052	3-HAO	3-hydroxyanthranilic acid dioxygenase	1	2	1	0
PF06058	DCP1	Dcp1-like decapping family	1	0	1	0
PF06068	TIP49	TIP49 C-terminus	2	2	2	0
PF06071	YchF-GTPase_C	Protein of unknown function (DUF933)	1	4	3	0
PF06079	Apyrase	Apyrase	0	1	1	2
PF06080	NA	Protein of unknown function (DUF938)	1	3	2	0
PF06083	IL17	Interleukin-17	10	8	13	14
PF06087	Tyr-DNA_phospho	Tyrosyl-DNA phosphodiesterase	1	1	1	0
PF06090	Ins_P5_2-kin	Inositol-pentakisphosphate 2-kinase	1	2	1	0

PF06093	Spt4	Spt4/RpoE2 zinc finger	1	1	1	0
PF06094	GGACT	Gamma-glutamyl cyclotransferase, AIG2-like	1	4	4	0
PF06098	NA	Radial spoke protein 3	2	2	2	2
PF06102	NA	Domain of unknown function (DUF947)	1	3	2	0
PF06105	Aph-1	Aph-1 protein	1	2	1	0
PF06110	DUF953	Eukaryotic protein of unknown function (DUF953)	1	2	1	0
PF06113	BRE	Brain and reproductive organ-expressed protein (BRE)	1	1	1	0
PF06119	NA	Nidogen-like	4	31	26	3
PF06140	NA	Interferon-induced 6-16 family	6	7	14	4
PF06148	NA	COG (conserved oligomeric Golgi) complex component, COG2	1	1	1	0
PF06151	NA	Trehalose receptor	1	0	1	1
PF06155	DUF971	Protein of unknown function (DUF971)	2	10	7	0
PF06159	NA	Protein of unknown function (DUF974)	1	3	2	2
PF06160	EzrA	Septation ring formation regulator, EzrA	0	1	0	0
PF06172	Cupin_5	Cupin superfamily (DUF985)	1	0	3	0
PF06179	Med22	Surfeit locus protein 5 subunit 22 of Mediator complex	1	2	2	1
PF06201	NA	PITH domain	2	1	2	0
PF06202	NA	Amylo-alpha-1,6-glucosidase	1	2	3	1
PF06206	CpeT	CpeT/CpcT family (DUF1001)	1	10	0	1
PF06209	NA	Cofactor of BRCA1 (COBRA1)	1	2	1	1
PF06211	NA	BMP and activin membrane-bound inhibitor (BAMBI) N-terminal domain	1	1	1	1
PF06212	GRIM-19	GRIM-19 protein	1	1	1	1
PF06218	NPR2	Nitrogen permease regulator 2	1	3	2	0
PF06220	zf-U1	U1 zinc finger	3	1	4	0
PF06221	NA	Putative zinc finger motif, C2HC5-type	1	2	1	0
PF06229	NA	FRG1-like domain	1	2	2	0
PF06237	NA	Protein of unknown function (DUF1011)	2	4	1	0
PF06239	NA	Evolutionarily conserved signalling intermediate in Toll pathway	1	1	1	1
PF06244	Ccdc124	Protein of unknown function (DUF1014)	1	0	1	0
PF06246	Isy1	Isy1-like splicing family	1	3	4	0
PF06248	NA	Centromere/kinetochore Zw10	2	5	1	1
PF06268	Fascin	Fascin domain	1	1	3	1
PF06271	NA	RDD family	0	0	1	0
PF06278	NA	Condensin II complex subunit CAP-H2 or CNDH2, N-terminal	1	1	0	0
PF06292	DUF1041	Domain of Unknown Function (DUF1041)	3	10	31	2
PF06294	CH_2	CH-like domain in sperm protein	4	9	23	0
PF06297	NA	PET Domain	3	4	11	3
PF06309	Torsin	Torsin	3	6	4	1
PF06311	NA	NUMB domain	1	8	8	1
PF06312	NA	Neurexophilin	5	2	0	0
PF06320	NA	GCN5-like protein 1 (GCN5L1)	1	1	1	0
PF06325	PrmA	Ribosomal protein L11 methyltransferase (PrmA)	5	3	5	0
PF06327	NA	Domain of Unknown Function (DUF1053)	3	4	3	0
PF06331	Tfb5	Transcription factor TFIIH complex subunit Tfb5	1	0	0	1
PF06333	NA	Mediator complex subunit 13 C-terminal	1	1	1	2
PF06337	DUSP	DUSP domain	3	3	4	0
PF06342	NA	Alpha/beta hydrolase of unknown function (DUF1057)	0	3	1	0
PF06345	Drf_DAD	DRF Autoregulatory Domain	1	4	4	0
PF06350	NA	Hormone-sensitive lipase (HSL) N-terminus	1	2	1	1
PF06367	Drf_FH3	Diaphanous FH3 Domain	7	12	24	0
PF06369	Anemone_cytotox	Sea anemone cytotoxic protein	0	0	1	0
PF06371	Drf_GBD	Diaphanous GTPase-binding Domain	6	9	23	2
PF06372	Gemin6	Gemin6 protein	1	1	2	1
PF06374	NDUF_C2	NADH-ubiquinone oxidoreductase subunit b14.5b (NDUFC2)	1	1	0	0
PF06375	AP3D1	Bovine leukaemia virus receptor (BLVR)	1	3	2	0
PF06377	NA	Adipokinetic hormone	0	0	1	0
PF06384	ICAT	Beta-catenin-interacting protein ICAT	1	1	1	0
PF06388	NA	Protein of unknown function (DUF1075)	1	1	0	0
PF06391	MAT1	CDK-activating kinase assembly factor MAT1	1	1	1	0
PF06396	NA	Angiotensin II, type I receptor-associated protein (AGTRAP)	1	1	1	0
PF06398	NA	Integral peroxisomal membrane peroxin	1	7	0	0
PF06399	GFRP	GTP cyclohydrolase I feedback regulatory protein (GFRP)	1	1	1	0
PF06400	Alpha-2-MRAP_N	Alpha-2-macroglobulin RAP, N-terminal domain	1	1	0	0
PF06401	Alpha-2-MRAP_C	Alpha-2-macroglobulin RAP, C-terminal domain	1	1	0	0
PF06413	NA	Neugrin	1	4	0	0
PF06415	iPGM_N	BPG-independent PGAM N-terminus (iPGM_N)	0	1	0	0
PF06417	DUF1077	Protein of unknown function (DUF1077)	1	1	2	0
PF06418	CTP_synth_N	CTP synthase N-terminus	1	3	2	0
PF06419	NA	Conserved oligomeric complex COG6	1	3	1	1
PF06421	LepA_C	GTP-binding protein LepA C-terminus	1	2	1	1
PF06422	NA	CDR ABC transporter	0	1	0	0
PF06423	NA	GWT1	1	1	0	0
PF06424	PRP1_N	PRP1 splicing factor, N-terminal	1	1	1	0
PF06427	UDP-g_GGTase	UDP-glucose:Glycoprotein Glucosyltransferase	1	1	1	1
PF06428	Sec2p	GDP/GTP exchange factor Sec2p	1	0	8	0
PF06429	Flg_bbr_C	Flagellar basal body rod FlgEFG protein C-terminal	0	0	1	0
PF06432	NA	Phosphatidylinositol N-acetylglucosaminyltransferase	1	3	1	0

PF06448	NA	Domain of Unknown Function (DUF1081)	0	3	1	1
PF06452	NA	Carbohydrate family 9 binding domain-like	2	2	0	0
PF06456	Arfaptin	Arfaptin-like domain	4	10	9	2
PF06459	RR_TM4-6	Ryanodine Receptor TM 4-6	1	1	5	0
PF06461	NA	Domain of Unknown Function (DUF1086)	1	2	12	1
PF06462	Hyd_WA	Propeller	2	3	5	1
PF06463	NA	Molybdenum Cofactor Synthesis C	1	1	1	0
PF06464	NA	DMAP1-binding Domain	1	1	9	0
PF06465	NA	Domain of Unknown Function (DUF1087)	1	2	12	1
PF06466	NA	PCAF (P300/CBP-associated factor) N-terminal domain	2	1	1	0
PF06467	zf-FCS	MYM-type Zinc finger with FCS sequence motif	1	0	5	0
PF06468	NA	Spondin_N	2	5	10	1
PF06469	NA	Domain of Unknown Function (DUF1088)	0	0	5	1
PF06470	SMC_hinge	SMC proteins Flexible Hinge Domain	4	5	6	2
PF06472	ABC_membrane_2	ABC transporter transmembrane region 2	3	6	5	1
PF06473	NA	FGF binding protein 1 (FGF-BP1)	3	0	0	0
PF06479	Ribonuc_2-5A	Ribonuclease 2-5A	2	2	3	0
PF06480	FtsH_ext	FtsH Extracellular	1	0	1	0
PF06482	Endostatin	Collagenase NC10 and Endostatin	1	1	4	1
PF06487	SAP18	Sin3 associated polypeptide p18 (SAP18)	1	1	1	1
PF06512	Na_trans_assoc	Sodium ion transport-associated	1	1	1	0
PF06522	NA	NADH-ubiquinone reductase complex 1 MLRQ subunit	2	2	1	0
PF06524	NA	NOA36 protein	1	1	0	0
PF06534	RGM_C	Repulsive guidance molecule (RGM) C-terminus	1	3	3	1
PF06535	RGM_N	Repulsive guidance molecule (RGM) N-terminus	1	3	3	0
PF06541	NA	Putative ABC-transporter type IV	2	0	1	2
PF06544	DUF1115	Protein of unknown function (DUF1115)	2	4	6	0
PF06546	NA	Vertebrate heat shock transcription factor	0	0	1	0
PF06553	BNIP3	BNIP3	1	1	2	0
PF06565	DUF1126	Repeat of unknown function (DUF1126)	2	3	2	4
PF06573	NA	Churchill protein	1	2	0	0
PF06574	FAD_syn	FAD synthetase	0	1	0	0
PF06581	NA	Mad1 and Cdc20-bound-Mad2 binding	1	1	1	1
PF06583	NA	Neogenin C-terminus	0	1	1	1
PF06584	NA	DIRP	1	1	4	0
PF06588	Muskelin_N	Muskelin N-terminus	1	2	1	0
PF06602	Myotub-related	Myotubularin-like phosphatase domain	7	9	17	4
PF06607	Prokineticin	Prokineticin	1	0	0	0
PF06608	NA	Protein of unknown function (DUF1143)	1	1	1	0
PF06617	NA	M-phase inducer phosphatase	0	1	4	0
PF06624	NA	Ribosome associated membrane protein RAMP4	1	0	0	0
PF06625	NA	Protein of unknown function (DUF1151)	1	1	3	1
PF06628	Catalase-rel	Catalase-related immune-responsive	1	3	8	0
PF06631	NA	Protein of unknown function (DUF1154)	2	5	8	1
PF06632	XRCC4	DNA double-strand break repair and V(D)J recombination protein XRCC4	1	1	4	0
PF06637	NA	PV-1 protein (PLVAP)	0	0	1	0
PF06638	NA	Strabismus protein	1	1	1	2
PF06644	NA	ATP11 protein	0	1	3	0
PF06645	NA	Microsomal signal peptidase 12 kDa subunit (SPC12)	0	1	1	0
PF06650	NA	SHR-binding domain of vacuolar-sorting associated protein 13	5	11	15	0
PF06653	NA	Tight junction protein, Claudin-like	1	8	0	0
PF06657	NA	Centrosome microtubule-binding domain of Cep57	1	1	7	1
PF06658	NA	Protein of unknown function (DUF1168)	1	1	2	0
PF06662	C5-epim_C	D-glucuronyl C5-epimerase C-terminus	1	2	13	2
PF06664	NA	Wnt-binding factor required for Wnt secretion	3	2	2	1
PF06668	NA	Inter-alpha-trypsin inhibitor heavy chain C-terminus	2	2	3	1
PF06677	Auto_anti-p27	Sjogren's syndrome/scleroderma autoantigen 1 (Autoantigen p27)	1	1	2	0
PF06679	NA	Protein of unknown function (DUF1180)	1	2	2	2
PF06682	SARAF	SOCE-associated regulatory factor of calcium homoeostasis	1	2	3	1
PF06694	NA	Plant nuclear matrix protein 1 (NMP1)	1	0	1	0
PF06699	NA	GPI biosynthesis protein family Pig-F	1	0	1	0
PF06701	MIB_HERC2	Mib_herc2	4	22	98	27
PF06702	Fam20C	Golgi casein kinase, C-terminal, Fam20	2	11	2	6
PF06703	NA	Microsomal signal peptidase 25 kDa subunit (SPC25)	1	2	2	0
PF06726	NA	Bladder cancer-related protein BC10	1	0	0	0
PF06728	NA	GPI transamidase subunit PIG-U	1	1	2	1
PF06730	NA	FAM92 protein	2	3	12	0
PF06732	Pescadillo_N	Pescadillo N-terminus	1	1	1	0
PF06733	DEAD_2	DEAD_2	4	7	5	0
PF06739	NA	Beta-propeller repeat	0	0	2	0
PF06741	NA	LsmAD domain	1	5	3	0
PF06743	NA	FAST kinase-like protein, subdomain 1	1	1	0	2
PF06747	CHCH	CHCH domain	2	4	2	2
PF06752	E_Pc_C	Enhancer of Polycomb C-terminus	0	1	1	1
PF06762	NA	Lipase maturation factor	2	2	1	0
PF06775	NA	Putative adipose-regulatory protein (Seipin)	1	1	2	0
PF06777	HBB	Helical and beta-bridge domain	1	1	1	0

PF06784	NA	Uncharacterised protein family (UPF0240)	1	1	1	1
PF06803	NA	Protein of unknown function (DUF1232)	1	1	1	1
PF06807	Clp1	Pre-mRNA cleavage complex II protein Clp1	1	2	1	0
PF06809	NA	Neural proliferation differentiation control-1 protein (NPDC1)	1	2	1	1
PF06813	NA	Nodulin-like	1	0	0	0
PF06814	NA	Lung seven transmembrane receptor	2	4	2	2
PF06816	NOD	NOTCH protein	1	2	3	0
PF06818	NA	Fez1	1	1	10	1
PF06821	NA	Serine hydrolase	1	0	3	0
PF06825	NA	Heat shock factor binding protein 1	0	1	1	0
PF06831	H2TH	Formamidopyrimidine-DNA glycosylase H2TH domain	2	3	2	0
PF06839	zf-GRF	GRF zinc finger	3	12	12	1
PF06840	DUF1241	Protein of unknown function (DUF1241)	1	2	2	0
PF06842	NA	Protein of unknown function (DUF1242)	2	1	1	0
PF06858	NOG1	Nucleolar GTP-binding protein 1 (NOG1)	1	1	1	0
PF06859	NA	Bicoid-interacting protein 3 (Bin3)	1	1	1	0
PF06862	NA	Utp25, U3 small nucleolar RNA-associated SSU processome protein 25	1	3	1	0
PF06869	NA	Protein of unknown function (DUF1258)	0	0	1	0
PF06870	RNA_pol_I_A49	A49-like RNA polymerase I associated factor	2	1	1	0
PF06881	NA	RNA polymerase II transcription factor SIII (Elongin) subunit A	1	1	1	0
PF06883	RNA_pol_Rpa2_4	RNA polymerase I, Rpa2 specific domain	1	1	1	1
PF06886	NA	Targeting protein for Xklp2 (TPX2)	1	1	2	1
PF06888	NA	Putative Phosphatase	1	1	1	1
PF06903	VirK	VirK protein	0	0	1	0
PF06905	FAIM1	Fas apoptotic inhibitory molecule (FAIM1)	1	4	6	0
PF06910	NA	Male enhanced antigen 1 (MEA1)	1	2	2	1
PF06911	NA	Senescence-associated protein	1	1	2	1
PF06916	NA	Protein of unknown function (DUF1279)	2	2	2	3
PF06920	DHR-2	Dock homology region 2	5	9	37	1
PF06936	NA	Selenoprotein S (SelS)	1	1	1	0
PF06941	NT5C	5' nucleotidase, deoxy (Pyrimidine), cytosolic type C protein (NT5C)	1	1	2	0
PF06951	NA	Group XII secretory phospholipase A2 precursor (PLA2G12)	1	2	2	0
PF06957	COPI_C	Coatomer (COPI) alpha subunit C-terminus	1	1	1	0
PF06963	NA	Ferroportin1 (FPN1)	1	6	1	7
PF06966	NA	Protein of unknown function (DUF1295)	2	2	2	0
PF06974	NA	Protein of unknown function (DUF1298)	1	1	3	1
PF06978	POP1	Ribonucleases P/MRP protein subunit POP1	1	1	2	0
PF06979	NA	Assembly, mitochondrial proton-transport ATP synth complex	1	2	1	1
PF06984	MRP-L47	Mitochondrial 39-S ribosomal protein L47 (MRP-L47)	1	1	1	0
PF06990	NA	Galactose-3-O-sulfotransferase	13	7	8	12
PF06991	MFAP1	Microfibril-associated/Pre-mRNA processing	1	1	1	0
PF07000	NA	Protein of unknown function (DUF1308)	1	1	2	1
PF07001	NA	BAT2 N-terminus	1	1	1	0
PF07002	Copine	Copine	9	12	20	5
PF07004	NA	Sperm-tail PG-rich repeat	13	11	18	2
PF07019	Rab5ip	Rab5-interacting protein (Rab5ip)	2	1	2	0
PF07034	ORC3_N	Origin recognition complex (ORC) subunit 3 N-terminus	1	1	2	0
PF07035	NA	Colon cancer-associated protein Mic1-like	1	1	1	0
PF07039	DUF1325	SGF29 tudor-like domain	1	8	2	0
PF07045	DUF1330	Domain of unknown function (DUF1330)	0	0	6	0
PF07047	NA	Optic atrophy 3 protein (OPA3)	2	0	0	0
PF07051	NA	Ovarian carcinoma immunoreactive antigen (OCIA)	2	1	0	0
PF07052	NA	Hepatocellular carcinoma-associated antigen 59	1	1	1	0
PF07054	NA	Pericardin like repeat	0	0	1	0
PF07061	Swi5	Swi5	1	1	1	0
PF07062	NA	Clc-like	0	1	0	0
PF07064	NA	RIC1	1	1	2	0
PF07065	D123	D123	1	7	1	1
PF07074	NA	Translocon-associated protein, gamma subunit (TRAP-gamma)	1	1	1	0
PF07084	Spot_14	Thyroid hormone-inducible hepatic protein Spot 14	1	1	1	1
PF07086	NA	Jagunal, ER re-organisation during oogenesis	1	1	1	0
PF07092	NA	Protein of unknown function (DUF1356)	0	1	4	0
PF07093	NA	SGT1 protein	1	1	3	0
PF07096	NA	Protein of unknown function (DUF1358)	1	1	1	0
PF07106	NA	Tat binding protein 1(TBP-1)-interacting protein (TBPIP)	1	4	1	0
PF07111	NA	Alpha helical coiled-coil rod protein (HCR)	1	1	1	0
PF07114	NA	Protein of unknown function (DUF1370)	1	1	0	1
PF07139	NA	Protein of unknown function (DUF1387)	1	2	3	0
PF07145	PAM2	Ataxin-2 C-terminal region	1	6	2	1
PF07147	PDCD9	Mitochondrial 28S ribosomal protein S30 (PDCD9)	1	1	1	0
PF07156	NA	Prenylcysteine lyase	1	2	2	1
PF07159	NA	Protein of unknown function (DUF1394)	2	2	15	0
PF07160	SKA1	Protein of unknown function (DUF1395)	1	2	2	0
PF07162	NA	Ciliary basal body-associated, B9 protein	3	5	6	0
PF07163	NA	Pex26 protein	1	5	1	0
PF07173	NA	Protein of unknown function (DUF1399)	3	6	11	8
PF07177	Neuralized	Neuralized	5	10	12	16

PF07189	SF3b10	Splicing factor 3B subunit 10 (SF3b10)	1	0	1	0
PF07200	Mod_r	Modifier of rudimentary (Mod(r)) protein	1	3	3	0
PF07202	Tcp10_C	T-complex protein 10 C-terminus	1	1	1	0
PF07206	NA	Baculovirus late expression factor 10 (LEF-10)	0	1	0	0
PF07217	NA	Heterokaryon incompatibility protein Het-C	0	0	1	0
PF07221	GlcNAc_2-epim	N-acylglucosamine 2-epimerase (GlcNAc 2-epimerase)	1	1	1	0
PF07225	NDUF_B4	NADH-ubiquinone oxidoreductase B15 subunit (NDUFB4)	1	1	0	0
PF07228	SpoIIE	Stage II sporulation protein E (SpoIIE)	1	1	1	0
PF07247	NA	Alcohol acetyltransferase	0	0	7	0
PF07258	NA	HCaRG protein	10	11	8	0
PF07264	EI24	Etoposide-induced protein 2.4 (EI24)	1	0	1	0
PF07281	NA	Insulin-induced protein (INSIG)	1	3	2	0
PF07287	NA	Protein of unknown function (DUF1446)	1	1	1	2
PF07289	BBL5	Protein of unknown function (DUF1448)	1	1	1	0
PF07297	NA	Dolichol phosphate-mannose biosynthesis regulatory protein (DPM2)	0	1	0	0
PF07303	Occludin_ELL	Occludin homology domain	1	1	1	0
PF07304	SRA1	Steroid receptor RNA activator (SRA1)	1	1	3	0
PF07324	NA	DiGeorge syndrome critical region 6 (DGCR6) protein	1	7	1	0
PF07347	CI-B14_5a	NADH:ubiquinone oxidoreductase subunit B14.5a (Complex I-B14.5a)	1	1	2	0
PF07366	SnoaL	SnoaL-like polyketide cyclase	0	0	1	0
PF07382	NA	Histone H1-like nucleoprotein HC2	0	1	0	0
PF07386	NA	Protein of unknown function (DUF1499)	1	0	0	0
PF07393	Sec10	Exocyst complex component Sec10	1	2	1	0
PF07406	NA	NICE-3 protein	1	2	1	0
PF07412	Geminin	Geminin	1	0	1	0
PF07426	Dynactin_p22	Dynactin subunit p22	1	2	2	0
PF07443	NA	HepA-related protein (HARP)	0	0	1	0
PF07452	NA	CHRD domain	1	0	1	0
PF07474	G2F	G2F domain	1	2	3	1
PF07475	Hpr_kinase_C	HPr Serine kinase C-terminal domain	0	1	0	0
PF07478	Dala_Dala_lig_C	D-ala D-ala ligase C-terminus	0	1	0	0
PF07479	NAD_Gly3P_dh_C	NAD-dependent glycerol-3-phosphate dehydrogenase C-terminus	2	4	2	0
PF07486	NA	Cell Wall Hydrolase	0	0	7	0
PF07491	NA	Protein phosphatase inhibitor	1	1	1	0
PF07494	Reg_prop	Two component regulator propeller	0	0	1	0
PF07496	zf-CW	CW-type Zinc Finger	6	19	9	0
PF07497	Rho_RNA_bind	Rho termination factor, RNA-binding domain	0	1	0	0
PF07498	Rho_N	Rho termination factor, N-terminal domain	8	2	2	0
PF07500	TFIIS_M	Transcription factor S-II (TFIIS), central domain	2	4	3	0
PF07502	MANEC	MANEC domain	1	6	4	1
PF07504	FTP	Fungalysin/Thermolysin Propeptide Motif	1	1	0	0
PF07516	SecA_SW	SecA Wing and Scaffold domain	0	1	0	0
PF07517	SecA_DEAD	SecA DEAD-like domain	0	2	0	0
PF07521	RMMBL	RNA-metabolising metallo-beta-lactamase	3	3	4	0
PF07522	DRMBL	DNA repair metallo-beta-lactamase	2	8	8	0
PF07524	Bromo_TP	Bromodomain associated	3	3	5	1
PF07525	SOCS_box	SOCS box	25	47	93	78
PF07527	NA	Hairy Orange	16	10	13	5
PF07528	DZF	DZF domain	2	7	4	1
PF07529	HSA	HSA	2	3	5	0
PF07531	TAFH	NHR1 homology to TAF	2	7	9	1
PF07533	BRK	BRK domain	1	6	12	1
PF07534	TLD	TLD	9	9	56	1
PF07535	zf-DBF	DBF zinc finger	2	2	1	0
PF07539	DRIM	Down-regulated in metastasis	1	1	2	0
PF07540	NOC3p	Nucleolar complex-associated protein	1	1	3	0
PF07541	EIF_2_alpha	Eukaryotic translation initiation factor 2 alpha subunit	1	1	0	0
PF07542	ATP12	ATP12 chaperone protein	1	1	1	0
PF07545	NA	Vestigial/Tondu family	2	4	3	1
PF07546	NA	EMI domain	2	0	3	0
PF07555	NAGidase	beta-N-acetylglucosaminidase	1	1	1	0
PF07557	NA	Shugoshin C terminus	1	0	0	0
PF07562	NCD3G	Nine Cysteines Domain of family 3 GPCR	8	12	5	5
PF07565	Band_3_cyto	Band 3 cytoplasmic domain	4	15	22	7
PF07569	NA	TUP1-like enhancer of split	1	2	1	0
PF07571	TAF6_C	TAF6 C-terminal HEAT repeat domain	2	5	3	0
PF07572	NA	Bucentaur or craniofacial development	1	2	3	0
PF07574	SMC_Nse1	Nse1 non-SMC component of SMC5-6 complex	1	2	1	0
PF07575	Nucleopor_Nup85	Nup85 Nucleoporin	1	2	1	0
PF07576	NA	BRCA1-associated protein 2	1	2	1	0
PF07593	NA	ASPIC and UnbV	1	0	1	1
PF07645	EGF_CA	Calcium-binding EGF domain	47	152	133	55
PF07646	NA	Kelch motif	1	1	6	2
PF07647	SAM_2	SAM domain (Sterile alpha motif)	12	32	85	5
PF07648	Kazal_2	Kazal-type serine protease inhibitor domain	18	26	87	7
PF07650	KH_2	KH domain	1	2	1	0
PF07651	ANTH	ANTH domain	2	8	7	0

PF07653	SH3_2	Variant SH3 domain	19	41	129	5
PF07657	MNNL	N terminus of Notch ligand	4	4	12	3
PF07662	Nucleos_tra2_C	Na+ dependent nucleoside transporter C-terminus	7	15	18	13
PF07670	Gate	Nucleoside recognition	3	4	9	0
PF07671	NA	Protein of unknown function (DUF1601)	0	0	2	0
PF07676	PD40	WD40-like Beta Propeller Repeat	0	5	3	0
PF07677	A2M_recep	A-macroglobulin receptor	13	12	15	1
PF07678	TED_complement	A-macroglobulin complement component	13	18	14	6
PF07679	I-set	Immunoglobulin I-set domain	155	185	460	135
PF07683	NA	Cobalamin synthesis protein cobW C-terminal domain	1	4	0	0
PF07684	NODP	NOTCH protein	1	2	0	0
PF07686	V-set	Immunoglobulin V-set domain	7	22	42	7
PF07687	M20_dimer	Peptidase dimerisation domain	7	7	9	1
PF07690	MFS_1	Major Facilitator Superfamily	145	215	276	30
PF07691	PA14	PA14 domain	1	1	3	1
PF07693	NA	KAP family P-loop domain	0	1	2	0
PF07699	NA	GCC2 and GCC3	10	49	54	5
PF07700	HNOB	Haem-NO-binding	7	5	24	6
PF07701	HNOBA	Heme NO binding associated	23	24	49	9
PF07703	A2M_BRD	Alpha-2-macroglobulin family N-terminal region	13	21	13	5
PF07707	BACK	BTB And C-terminal Kelch	49	169	157	43
PF07710	P53_tetramer	P53 tetramerisation motif	1	4	5	0
PF07711	RabGGT_insert	Rab geranylgeranyl transferase alpha-subunit, insert domain	1	1	1	0
PF07713	NA	Protein of unknown function (DUF1604)	1	1	1	0
PF07714	Pkinase_Tyr	Protein tyrosine kinase	93	173	252	78
PF07715	Plug	TonB-dependent Receptor Plug Domain	0	0	1	0
PF07716	bZIP_2	Basic region leucine zipper	16	36	31	12
PF07717	OB_NTP_bind	Oligonucleotide/oligosaccharide-binding (OB)-fold	14	24	19	4
PF07718	Coatamer_beta_C	Coatomer beta C-terminal region	1	1	1	1
PF07719	TPR_2	Tetratricopeptide repeat	22	37	72	3
PF07721	NA	Tetratricopeptide repeat	0	1	0	0
PF07722	Peptidase_C26	Peptidase C26	1	5	2	0
PF07724	AAA_2	AAA domain (Cdc48 subfamily)	2	4	4	0
PF07728	AAA_5	AAA domain (dynein-related subfamily)	13	27	42	7
PF07731	Cu-oxidase_2	Multicopper oxidase	9	20	46	12
PF07732	Cu-oxidase_3	Multicopper oxidase	10	18	45	11
PF07733	DNA_pol3_alpha	Bacterial DNA polymerase III alpha subunit	0	1	0	0
PF07738	Sad1_UNC	Sad1 / UNC-like C-terminal	2	7	7	0
PF07741	BRF1	Brf1-like TBP-binding domain	1	1	1	1
PF07742	BTG	BTG family	3	11	5	3
PF07743	NA	HSCB C-terminal oligomerisation domain	1	1	1	1
PF07744	SPOC	SPOC domain	3	3	4	3
PF07748	Glyco_hydro_38C	Glycosyl hydrolases family 38 C-terminal domain	6	16	22	5
PF07749	ERp29	Endoplasmic reticulum protein ERp29, C-terminal domain	2	1	1	1
PF07757	NA	Predicted AdoMet-dependent methyltransferase	1	1	1	0
PF07763	NA	FEZ-like protein	1	2	1	2
PF07766	NA	LETM1-like protein	2	3	2	4
PF07767	Nop53	Nop53 (60S ribosomal biogenesis)	1	1	2	0
PF07773	NA	Protein of unknown function (DUF1619)	2	2	5	1
PF07774	EMC1_C	Protein of unknown function (DUF1620)	1	1	3	1
PF07776	zf-AD	Zinc-finger associated domain (zf-AD)	0	0	1	0
PF07778	CENP-I	Mis6	0	3	2	0
PF07779	NA	10 TM Acyl Transferase domain found in Cas1p	2	4	2	2
PF07780	Spb1_C	Spb1 C-terminal domain	0	1	1	0
PF07782	NA	DC-STAMP-like protein	4	3	5	2
PF07786	NA	Protein of unknown function (DUF1624)	0	1	0	0
PF07787	NA	Protein of unknown function (DUF1625)	1	0	3	0
PF07798	DUF1640	Protein of unknown function (DUF1640)	1	0	2	0
PF07801	NA	Protein of unknown function (DUF1647)	4	20	3	0
PF07807	NA	RED-like protein C-terminal region	1	2	1	0
PF07808	RED_N	RED-like protein N-terminal region	1	2	2	0
PF07809	NA	RTP801 C-terminal region	1	1	0	1
PF07810	NA	TMC domain	5	11	17	2
PF07814	NA	Wings apart-like protein regulation of heterochromatin	1	2	4	1
PF07815	NA	Abl-interactor HHR	1	5	4	0
PF07817	GLE1	GLE1-like protein	1	1	1	2
PF07818	NA	HCNGP-like protein	1	5	1	0
PF07819	NA	PGAP1-like protein	1	2	1	0
PF07830	PP2C_C	Protein serine/threonine phosphatase 2C, C-terminal domain	1	2	1	0
PF07831	PYNP_C	Pyrimidine nucleoside phosphorylase C-terminal domain	1	5	2	0
PF07834	RanGAP1_C	RanGAP1 C-terminal domain	1	2	1	0
PF07836	DmpG_comm	DmpG-like communication domain	0	0	1	0
PF07837	FTCD_N	Formiminotransferase domain, N-terminal subdomain	1	1	3	1
PF07842	NA	GC-rich sequence DNA-binding factor-like protein	2	2	2	1
PF07847	PCO_ADO	Protein of unknown function (DUF1637)	1	1	1	1
PF07850	Renin_r	Renin receptor-like protein	1	1	1	0
PF07851	NA	TMPIT-like protein	1	1	1	0

PF07855	ATG101	Protein of unknown function (DUF1649)	1	1	1	0
PF07856	Orai-1	Mediator of CRAC channel activity	2	1	1	1
PF07857	NA	Transmembrane family, TMEM144 of transporters	1	1	1	0
PF07859	Abhydrolase_3	alpha/beta hydrolase fold	12	20	16	5
PF07883	Cupin_2	Cupin domain	1	2	3	3
PF07884	VKOR	Vitamin K epoxide reductase family	2	2	1	0
PF07885	Ion_trans_2	Ion channel	20	29	59	38
PF07888	NA	Calcium binding and coiled-coil domain (CALCOCO1) like	3	0	4	0
PF07890	NA	Rrp15p	1	1	2	0
PF07896	DUF1674	Protein of unknown function (DUF1674)	0	1	1	0
PF07904	NA	Chromatin modification-related protein EAF7	1	1	2	0
PF07910	Peptidase_C78	Peptidase family C78	3	7	3	0
PF07912	ERp29_N	ERp29, N-terminal domain	1	1	1	0
PF07915	PRKCSH	Glucosidase II beta subunit-like protein	2	2	3	0
PF07919	NA	Gryzun, putative trafficking through Golgi	1	0	0	0
PF07923	NA	N1221-like protein	1	1	3	0
PF07926	NA	TPR/MLP1/MLP2-like protein	1	2	1	0
PF07928	Vps54	Vps54-like protein	1	1	1	0
PF07933	DUF1681	Protein of unknown function (DUF1681)	1	1	2	0
PF07934	OGG_N	8-oxoguanine DNA glycosylase, N-terminal domain	0	2	1	1
PF07942	N2227	N2227-like protein	1	1	0	1
PF07946	NA	Protein of unknown function (DUF1682)	1	1	1	0
PF07947	NA	YhhN-like protein	2	1	4	4
PF07956	NA	Protein of Unknown function (DUF1690)	1	0	0	0
PF07959	NA	L-fucokinase	2	3	2	1
PF07962	Swi3	Replication Fork Protection Component Swi3	1	1	4	0
PF07965	Integrin_B_tail	Integrin beta tail domain	4	1	6	0
PF07966	A1_Propeptide	A1 Propeptide	1	1	0	0
PF07967	NA	C3HC zinc finger-like	1	1	1	0
PF07969	Amidohydro_3	Amidohydrolase family	0	2	2	0
PF07970	NA	Endoplasmic reticulum vesicle transporter	4	7	5	0
PF07973	tRNA_SAD	Threonyl and Alanyl tRNA synthetase second additional domain	5	12	6	0
PF07974	EGF_2	EGF-like domain	6	14	42	13
PF07975	C1_4	TFIIH C1-like domain	1	1	1	0
PF07978	NIPSNAP	NIPSNAP	1	4	3	1
PF07984	NTP_transf_7	Nucleotidyltransferase	1	1	1	1
PF07985	NA	SRR1	1	0	2	0
PF07986	TBCC	Tubulin binding cofactor C	3	3	4	1
PF07989	NA	Centrosomin N-terminal motif 1	0	7	6	1
PF07992	Pyr_redox_2	Pyridine nucleotide-disulphide oxidoreductase	13	26	24	2
PF07993	NAD_binding_4	Male sterility protein	4	4	6	2
PF07994	NAD_binding_5	Myo-inositol-1-phosphate synthase	1	1	2	6
PF07995	GSDH	Glucose / Sorbosone dehydrogenase	1	3	4	1
PF07998	Peptidase_M54	Peptidase family M54	2	1	4	2
PF08005	PHR	PHR domain	3	7	36	5
PF08007	Cupin_4	Cupin superfamily protein	1	4	1	0
PF08014	NA	Domain of unknown function (DUF1704)	2	7	5	0
PF08016	PKD_channel	Polycystin cation channel	12	32	30	31
PF08017	NA	Fibrinogen binding protein	2	2	0	1
PF08022	FAD_binding_8	FAD-binding domain	7	9	15	2
PF08030	NAD_binding_6	Ferric reductase NAD binding domain	7	6	14	2
PF08031	BBE	Berberine and berberine like	3	1	4	2
PF08032	SpoU_sub_bind	RNA 2'-O ribose methyltransferase substrate binding	0	3	1	0
PF08033	Sec23_BS	Sec23/Sec24 beta-sandwich domain	3	5	11	0
PF08059	NA	SEP domain	2	3	5	1
PF08063	PADR1	PADR1 (NUC008) domain	1	1	1	0
PF08064	UME	UME (NUC010) domain	1	1	1	0
PF08066	PMC2NT	PMC2NT (NUC016) domain	0	1	1	0
PF08068	DKCLD	DKCLD (NUC011) domain	1	1	1	0
PF08069	Ribosomal_S13_N	Ribosomal S13/S15 N-terminal domain	1	1	1	0
PF08070	NA	DTHCT (NUC029) region	0	4	1	0
PF08071	RS4NT	RS4NT (NUC023) domain	1	1	1	0
PF08073	NA	CHDNT (NUC034) domain	1	2	12	1
PF08074	NA	CHDCT2 (NUC038) domain	1	1	12	1
PF08075	NOPS	NOPS (NUC059) domain	1	1	3	0
PF08079	Ribosomal_L30_N	Ribosomal L30 N-terminal domain	0	1	1	0
PF08082	PRO8NT	PRO8NT (NUC069), PrP8 N-terminal domain	1	1	1	3
PF08083	PROCN	PROCN (NUC071) domain	2	1	1	2
PF08084	PROCT	PROCT (NUC072) domain	1	1	1	0
PF08123	DOT1	Histone methylation protein DOT1	1	1	5	0
PF08127	Propeptide_C1	Peptidase family C1 propeptide	1	2	2	0
PF08142	AARP2CN	AARP2CN (NUC121) domain	2	3	2	0
PF08144	CPL	CPL (NUC119) domain	1	1	2	0
PF08145	BOP1NT	BOP1NT (NUC169) domain	1	1	1	0
PF08146	BP28CT	BP28CT (NUC211) domain	1	1	1	0
PF08147	NA	DBP10CT (NUC160) domain	1	1	1	0
PF08148	DSHCT	DSHCT (NUC185) domain	2	4	3	0

PF08149	BING4CT	BING4CT (NUC141) domain	1	1	1	0
PF08150	NA	FerB (NUC096) domain	4	2	19	2
PF08151	NA	FerI (NUC094) domain	2	2	19	0
PF08152	GUCT	GUCT (NUC152) domain	1	0	1	0
PF08153	NA	NGP1NT (NUC091) domain	1	1	1	0
PF08154	NLE	NLE (NUC135) domain	2	1	2	0
PF08155	NOGCT	NOGCT (NUC087) domain	1	1	1	0
PF08156	NOP5NT	NOP5NT (NUC127) domain	2	2	2	0
PF08158	NA	NUC130/3NT domain	0	2	0	0
PF08159	NA	NUC153 domain	1	2	4	1
PF08161	NA	NUC173 domain	1	1	1	0
PF08163	NUC194	NUC194 domain	2	1	1	1
PF08164	TRAUB	Apoptosis-antagonizing transcription factor, C-terminal	1	1	1	0
PF08165	NA	FerA (NUC095) domain	1	1	18	1
PF08167	RIX1	rRNA processing/ribosome biogenesis	1	0	0	0
PF08169	RBB1NT	RBB1NT (NUC162) domain	1	1	1	0
PF08170	POPLD	POPLD (NUC188) domain	1	1	2	1
PF08172	NA	CASP C terminal	1	1	1	0
PF08174	NA	Cell division protein anillin	2	1	9	1
PF08190	PIH1	pre-RNA processing PIH1/Nop17	4	3	4	1
PF08192	NA	Peptidase family S64	0	0	9	0
PF08202	MIS13	Mis12-Mtw1 protein family	0	1	1	0
PF08205	C2-set_2	CD80-like C2-set immunoglobulin domain	5	6	62	6
PF08208	RNA_polI_A34	DNA-directed RNA polymerase I subunit RPA34.5	1	1	1	0
PF08209	Sgf11	Sgf11 (transcriptional regulation protein)	1	0	2	0
PF08210	NA	APOBEC-like N-terminal domain	0	0	5	11
PF08211	dCMP_cyt_deam_2	Cytidine and deoxycytidylate deaminase zinc-binding region	0	1	0	0
PF08212	Lipocalin_2	Lipocalin-like domain	5	0	7	0
PF08213	DUF1713	Mitochondrial domain of unknown function (DUF1713)	0	5	0	0
PF08214	HAT_KAT11	Histone acetylation protein	1	1	4	2
PF08216	NA	Catenin-beta-like, Arm-motif containing nuclear	1	1	1	0
PF08217	NA	Fungal domain of unknown function (DUF1712)	1	2	1	0
PF08221	HTH_9	RNA polymerase III subunit RPC82 helix-turn-helix domain	1	1	1	0
PF08231	SYF2	SYF2 splicing factor	1	1	1	0
PF08232	NA	Striatin family	1	3	6	1
PF08234	NA	Chromosome segregation protein Spc25	1	4	1	0
PF08235	LNS2	LNS2 (Lipin/Ned1/Smp2)	1	1	3	1
PF08236	SRI	SRI (Set2 Rpb1 interacting) domain	1	0	6	0
PF08238	Sel1	Sel1 repeat	5	7	10	2
PF08239	SH3_3	Bacterial SH3 domain	1	1	3	3
PF08240	ADH_N	Alcohol dehydrogenase GroES-like domain	16	25	17	5
PF08241	Methyltransf_11	Methyltransferase domain	14	29	30	3
PF08242	Methyltransf_12	Methyltransferase domain	1	2	1	0
PF08243	NA	SPT2 chromatin protein	1	1	1	0
PF08245	Mur_ligase_M	Mur ligase middle domain	0	7	5	0
PF08246	Inhibitor_I29	Cathepsin propeptide inhibitor domain (I29)	11	15	25	0
PF08264	Anticodon_1	Anticodon-binding domain of tRNA	7	11	10	4
PF08265	NA	YL1 nuclear protein C-terminal domain	2	4	3	1
PF08266	Cadherin_2	Cadherin-like	8	17	50	16
PF08271	TF_Zn_Ribbon	TFIIB zinc-binding	2	6	3	1
PF08276	PAN_2	PAN-like domain	1	1	0	1
PF08277	NA	PAN-like domain	6	0	0	1
PF08282	Hydrolase_3	haloacid dehalogenase-like hydrolase	1	2	1	0
PF08284	NA	Retroviral aspartyl protease	2	0	0	0
PF08285	NA	Dolichol-phosphate mannosyltransferase subunit 3 (DPM3)	1	1	1	0
PF08286	Spc24	Spc24 subunit of Ndc80	0	0	1	0
PF08288	NA	PIGA (GPI anchor biosynthesis)	1	1	1	0
PF08292	RNA_pol_Rbc25	RNA polymerase III subunit Rpc25	1	3	1	0
PF08293	MRP-S33	Mitochondrial ribosomal subunit S27	1	1	3	0
PF08294	TIM21	TIM21	0	0	1	0
PF08295	NA	Sin3 family co-repressor	1	2	6	0
PF08299	Bac_DnaA_C	Bacterial dnaA protein helix-turn-helix	0	1	0	0
PF08311	Mad3_BUB1_I	Mad3/BUB1 homology region 1	1	1	1	0
PF08312	NA	cwf21 domain	2	2	1	0
PF08313	SCA7	SCA7, zinc-binding domain	1	1	1	0
PF08314	NA	Secretory pathway protein Sec39	1	1	0	0
PF08315	NA	cwf18 pre-mRNA splicing factor	1	1	1	0
PF08317	NA	Spc7 kinetochore protein	1	1	0	0
PF08318	NA	COG4 transport protein	1	1	1	0
PF08320	NA	PIG-X / PBN1	1	2	3	0
PF08321	PPP5	PPP5 TPR repeat region	1	2	3	0
PF08324	PUL	PUL domain	1	1	2	1
PF08326	ACC_central	Acetyl-CoA carboxylase, central region	1	1	1	2
PF08327	AHSA1	Activator of Hsp90 ATPase homolog 1-like protein	1	1	1	0
PF08332	CaMKII_AD	Calcium/calmodulin dependent protein kinase II association domain	1	1	10	0
PF08336	P4Ha_N	Prolyl 4-Hydroxylase alpha-subunit, N-terminal region	5	5	10	3
PF08337	Plexin_cytopl	Plexin cytoplasmic RasGAP domain	4	7	10	2

PF08338	DUF1731	Domain of unknown function (DUF1731)	1	1	1	0
PF08344	TRP_2	Transient receptor ion channel II	6	9	13	4
PF08346	NA	AntA/AntB antirepressor	0	0	0	1
PF08347	NA	N-terminal CTNNB1 binding	1	4	2	0
PF08351	DUF1726	Domain of unknown function (DUF1726)	1	4	1	0
PF08355	EF_assoc_1	EF hand associated	1	1	4	0
PF08356	EF_assoc_2	EF hand associated	1	1	4	0
PF08357	SEFIR	SEFIR domain	4	9	13	3
PF08366	LLGL	LLGL2	2	3	10	1
PF08367	M16C_assoc	Peptidase M16C associated	1	2	0	0
PF08368	NA	FAST kinase-like protein, subdomain 2	1	1	0	0
PF08372	NA	Plant phosphoribosyltransferase C-terminal	1	3	2	0
PF08373	NA	RAP domain	0	2	3	0
PF08375	Rpn3_C	Proteasome regulatory subunit C-terminal	2	1	2	1
PF08376	NIT	Nitrate and nitrite sensing	7	1	12	3
PF08378	NA	Nuclease-related domain	0	0	0	3
PF08385	DHC_N1	Dynein heavy chain, N-terminal region 1	10	20	28	28
PF08389	Xpo1	Exportin 1-like protein	8	15	13	2
PF08390	NA	TRAM1-like protein	1	1	1	0
PF08393	DHC_N2	Dynein heavy chain, N-terminal region 2	21	39	55	54
PF08395	NA	7tm Chemosensory receptor	9	4	4	0
PF08397	IMD	IRSp53/MIM homology domain	1	12	16	0
PF08399	VWA_N	VWA N-terminal	4	3	13	0
PF08403	NA	Amino acid permease N-terminal	1	1	2	1
PF08409	NA	Domain of unknown function (DUF1736)	5	3	11	2
PF08412	Ion_trans_N	Ion transport protein N-terminal	1	1	4	1
PF08416	PTB	Phosphotyrosine-binding domain	2	1	13	0
PF08418	Pol_alpha_B_N	DNA polymerase alpha subunit B N-terminal	1	1	1	0
PF08423	Rad51	Rad51	2	12	10	1
PF08424	NA	NRDE-2, necessary for RNA interference	1	1	1	2
PF08427	NA	Domain of unknown function (DUF1741)	1	3	2	0
PF08429	PLU-1	PLU-1-like protein	1	2	4	1
PF08430	NA	Forkhead N-terminal region	1	1	1	1
PF08432	NA	AAA-ATPase Vps4-associated protein 1	1	0	0	0
PF08433	KTI12	Chromatin associated protein KTI12	2	7	2	0
PF08434	NA	Calcium-activated chloride channel	3	3	4	0
PF08438	MMR_HSR1_C	GTPase of unknown function C-terminal	0	0	1	0
PF08441	Integrin_alpha2	Integrin alpha	3	9	18	0
PF08442	ATP-grasp_2	ATP-grasp domain	3	7	7	0
PF08443	RimK	RimK-like ATP-grasp domain	0	0	1	0
PF08445	NA	FR47-like protein	5	0	16	3
PF08447	PAS_3	PAS fold	6	5	10	1
PF08449	NA	UAA transporter family	4	8	4	2
PF08450	SGL	SMP-30/Gluconolaconase/LRE-like region	4	2	14	5
PF08451	A_deaminase_N	Adenosine/AMP deaminase N-terminal	2	12	3	1
PF08454	RIH_assoc	RyR and IP3R Homology associated	4	13	17	1
PF08457	NA	Sfi1 spindle body protein	1	1	6	1
PF08473	VGCC_alpha2	Neuronal voltage-dependent calcium channel alpha 2acd	1	0	3	0
PF08477	Roc	Ras of Complex, Roc, domain of DAPkinase	6	21	45	6
PF08487	VIT	Vault protein inter-alpha-trypsin domain	12	14	18	0
PF08490	DUF1744	Domain of unknown function (DUF1744)	1	1	1	0
PF08491	SE	Squalene epoxidase	1	1	0	1
PF08492	SRP72	SRP72 RNA-binding domain	1	1	1	0
PF08499	NA	3'5'-cyclic nucleotide phosphodiesterase N-terminal	1	1	10	1
PF08502	LeuA_dimer	LeuA allosteric (dimerisation) domain	0	0	1	0
PF08506	NA	Cse1	2	2	1	0
PF08510	NA	PIG-P	1	1	0	0
PF08511	NA	COQ9	1	1	8	1
PF08512	Rtt106	Histone chaperone Rttp106-like	2	3	4	0
PF08513	NA	LisH	4	4	25	0
PF08514	STAG	STAG domain	1	6	4	0
PF08515	TGF_beta_GS	Transforming growth factor beta type I GS-motif	3	3	6	0
PF08516	ADAM_CR	ADAM cysteine-rich	2	2	4	0
PF08517	AXH	Ataxin-1 and HBP1 module (AXH)	1	1	5	2
PF08518	NA	Spa2 homology domain (SHD) of GIT	1	1	4	0
PF08519	NA	Replication factor RFC1 C terminal domain	1	0	2	0
PF08523	NA	Multiprotein bridging factor 1	1	0	1	0
PF08524	NA	rRNA processing	1	0	1	0
PF08529	NusA_N	NusA N-terminal domain	0	1	0	0
PF08534	Redoxin	Redoxin	2	2	2	0
PF08539	NA	HbrB-like	1	1	2	0
PF08540	HMG_CoA_synt_C	Hydroxymethylglutaryl-coenzyme A synthase C terminal	1	1	1	0
PF08541	ACP_syn_III_C	3-Oxoacyl-[acyl-carrier-protein (ACP)] synthase III C terminal	0	1	0	0
PF08542	Rep_fac_C	Replication factor C C-terminal domain	4	10	5	0
PF08543	Phos_pyr_kin	Phosphomethylpyrimidine kinase	1	1	1	0
PF08544	GHMP_kinases_C	GHMP kinases C terminal	5	6	3	0
PF08545	ACP_syn_III	3-Oxoacyl-[acyl-carrier-protein (ACP)] synthase III	0	1	0	0

PF08547	NA	Complex I intermediate-associated protein 30 (CIA30)	1	6	1	1
PF08551	NA	Eukaryotic integral membrane protein (DUF1751)	1	1	1	0
PF08555	NA	Eukaryotic family of unknown function (DUF1754)	1	2	3	0
PF08557	NA	Sphingolipid Delta4-desaturase (DES)	2	5	1	1
PF08558	TRF	Telomere repeat binding factor (TRF)	0	0	1	0
PF08559	Cut8	Cut8, nuclear proteasome tether protein	1	0	1	0
PF08561	NA	Mitochondrial ribosomal protein L37	1	0	1	1
PF08563	P53_TAD	P53 transactivation motif	0	0	4	0
PF08564	CDC37_C	Cdc37 C terminal domain	1	1	1	1
PF08565	CDC37_M	Cdc37 Hsp90 binding domain	1	1	1	0
PF08567	PH_TFIIH	TFIIH p62 subunit, N-terminal domain	1	1	2	0
PF08568	NA	Uncharacterised protein family, YAP/Alf4/glomulin	1	1	1	1
PF08569	Mo25	Mo25-like	0	1	1	0
PF08570	NA	Protein of unknown function (DUF1761)	1	0	1	0
PF08571	NA	Yos1-like	0	0	1	0
PF08572	PRP3	pre-mRNA processing factor 3 (PRP3)	1	1	1	0
PF08573	NA	DNA repair protein endonuclease SAE2/CtIP C-terminus	1	1	0	0
PF08574	NA	Protein of unknown function (DUF1762)	1	1	1	1
PF08583	NA	Cytochrome c oxidase biogenesis protein Cmc1 like	1	3	1	0
PF08584	Ribonuc_P_40	Ribonuclease P 40kDa (Rpp40) subunit	1	1	2	0
PF08585	RMI1_N	RecQ mediated genome instability protein	2	2	4	0
PF08590	NA	Domain of unknown function (DUF1771)	0	1	1	0
PF08592	NA	Domain of unknown function (DUF1772)	0	0	6	0
PF08596	Lgl_C	Lethal giant larvae(Lgl) like, C-terminal	0	2	10	0
PF08597	eIF3_subunit	Translation initiation factor eIF3 subunit	1	2	1	0
PF08598	NA	Sds3-like	2	6	6	0
PF08599	NA	DNA damage repair protein Nbs1	0	1	2	1
PF08600	NA	Rsm1-like	1	1	1	0
PF08603	CAP_C	Adenylate cyclase associated (CAP) C terminal	1	1	3	0
PF08604	NA	Nucleoporin Nup153-like	0	3	0	0
PF08606	Prp19	Prp19/Pso4-like	1	1	1	0
PF08608	Wyosine_form	Wyosine base formation	1	1	1	1
PF08609	NA	Nucleotide exchange factor Fes1	1	0	1	0
PF08610	NA	Peroxisomal membrane protein (Pex16)	1	1	1	0
PF08612	Med20	TATA-binding related factor (TRF) of subunit 20 of Mediator complex	1	1	1	1
PF08613	Cyclin	Cyclin	1	0	1	0
PF08614	ATG16	Autophagy protein 16 (ATG16)	1	1	3	0
PF08615	RNase_H2_suC	Ribonuclease H2 non-catalytic subunit (Ylr154p-like)	1	1	2	0
PF08616	NA	Stabilization of polarity axis	2	1	3	0
PF08617	CGI-121	Kinase binding protein CGI-121	1	1	1	0
PF08618	NA	Transcription factor Opi1	1	0	2	0
PF08620	NA	RPAP1-like, C-terminal	1	0	1	0
PF08621	NA	RPAP1-like, N-terminal	1	1	1	0
PF08623	NA	TATA-binding protein interacting (TIP20)	1	1	2	0
PF08625	Utp13	Utp13 specific WD40 associated domain	1	1	1	0
PF08626	NA	Transport protein Trs120 or TRAPPC9, TRAPP II complex subunit	1	1	3	0
PF08627	NA	CRT-like, chloroquine-resistance transporter-like	2	2	3	0
PF08628	NA	Sorting nexin C terminal	4	4	7	1
PF08629	NA	PDE8 phosphodiesterase	0	3	1	1
PF08631	NA	Meiosis protein SPO22/ZIP4 like	1	5	2	0
PF08638	Med14	Mediator complex subunit MED14	1	0	2	0
PF08640	U3_assoc_6	U3 small nucleolar RNA-associated protein 6	1	1	1	0
PF08644	SPT16	FACT complex subunit (SPT16/CDC68)	1	2	1	0
PF08645	PNK3P	Polynucleotide kinase 3 phosphatase	2	2	3	0
PF08646	Rep_fac-A_C	Replication factor-A C terminal domain	2	1	5	0
PF08647	NA	BRE1 E3 ubiquitin ligase	0	1	1	1
PF08648	NA	Protein of unknown function (DUF1777)	1	2	2	0
PF08652	RAI1	RAI1 like PD-(D/E)XK nuclease	1	4	1	0
PF08658	NA	Rad54 N terminal	0	1	0	0
PF08659	KR	KR domain	3	6	2	7
PF08660	NA	Oligosaccharide biosynthesis protein Alg14 like	1	2	1	0
PF08661	Rep_fac-A_3	Replication factor A protein 3	1	1	1	0
PF08662	eIF2A	Eukaryotic translation initiation factor eIF2A	4	4	10	0
PF08666	SAF	SAF domain	0	0	1	0
PF08669	GCV_T_C	Glycine cleavage T-protein C-terminal barrel domain	5	7	3	1
PF08672	ANAPC2	Anaphase promoting complex (APC) subunit 2	1	1	1	1
PF08674	AChE_tetra	Acetylcholinesterase tetramerisation domain	0	1	1	0
PF08675	RNA_bind	RNA binding domain	1	2	2	0
PF08676	MutL_C	MutL C terminal dimerisation domain	2	2	2	0
PF08683	CAMSAP_CKK	Microtubule-binding calmodulin-regulated spectrin-associated	1	1	3	0
PF08685	NA	GON domain	39	1	14	0
PF08686	NA	PLAC (protease and lacunin) domain	5	4	18	0
PF08687	ASD2	Apx/Shroom domain ASD2	1	1	5	1
PF08694	UFC1	Ubiquitin-fold modifier-conjugating enzyme 1	1	1	1	0
PF08695	NA	Cytochrome oxidase complex assembly protein 1	1	2	1	0
PF08696	Dna2	DNA replication factor Dna2	0	1	1	0
PF08698	Fcf2	Fcf2 pre-rRNA processing	1	1	1	0

PF08699	ArgoL1	Argonaute linker 1 domain	1	5	8	0
PF08700	Vps51	Vps51/Vps67	2	3	4	0
PF08701	GN3L_Grn1	GNL3L/Grn1 putative GTPase	1	2	1	0
PF08703	NA	PLC-beta C terminal	0	2	0	1
PF08704	GCD14	tRNA methyltransferase complex GCD14 subunit	1	1	1	1
PF08707	NA	Primase C terminal 2 (PriCT-2)	0	0	1	0
PF08709	Ins145_P3_rec	Inositol 1,4,5-trisphosphate/ryanodine receptor	5	13	18	1
PF08711	NA	TFIIS helical bundle-like domain	5	10	7	2
PF08712	NA	Scaffold protein Nfu/NifU N terminal	1	1	2	0
PF08713	DNA_alkylation	DNA alkylation repair enzyme	2	4	0	0
PF08718	GLTP	Glycolipid transfer protein (GLTP)	4	7	5	4
PF08719	NA	Domain of unknown function (DUF1768)	2	4	31	0
PF08725	Integrin_b_cyt	Integrin beta cytoplasmic domain	3	1	10	1
PF08726	EFhand_Ca_insen	Ca2+ insensitive EF hand	2	10	11	0
PF08729	HUN	HPC2 and ubinuclein domain	0	2	2	0
PF08736	NA	FERM adjacent (FA)	6	27	22	0
PF08737	NA	Rgp1	0	3	1	0
PF08738	NA	Gon7 family	0	0	1	0
PF08740	BCS1_N	BCS1 N terminal	1	4	1	0
PF08742	C8	C8 domain	6	6	8	1
PF08743	NA	Nse4 C-terminal	1	1	1	0
PF08746	zf-RING-like	RING-like domain	1	2	1	0
PF08752	COP-gamma_platf	Coatomer gamma subunit appendage platform subdomain	1	1	1	1
PF08755	YccV-like	Hemimethylated DNA-binding protein YccV like	2	3	3	0
PF08763	Ca_chan_IQ	Voltage gated calcium channel IQ domain	2	3	35	1
PF08766	DEK_C	DEK C terminal domain	3	5	9	0
PF08767	CRM1_C	CRM1 C terminal	1	1	2	2
PF08768	DUF1794	Domain of unknown function (DUF1794)	1	2	1	0
PF08771	FRB_dom	Rapamycin binding domain	1	3	3	1
PF08772	NOB1_Zn_bind	Nin one binding (NOB1) Zn-ribbon like	1	0	3	0
PF08773	CathepsinC_exc	Cathepsin C exclusion domain	2	3	1	0
PF08774	VRR_NUC	VRR-NUC domain	1	2	1	0
PF08776	VASP_tetra	VASP tetramerisation domain	0	1	9	0
PF08777	RRM_3	RNA binding motif	1	2	3	0
PF08778	HIF-1a_CTAD	HIF-1 alpha C terminal transactivation domain	0	1	0	0
PF08781	DP	Transcription factor DP	1	4	3	0
PF08782	c-SKI_SMAD_bind	c-SKI Smad4 binding domain	4	3	9	1
PF08783	NA	DWNN domain	1	1	1	0
PF08784	RPA_C	Replication protein A C terminal	0	1	2	0
PF08785	NA	Ku C terminal domain like	1	1	1	0
PF08788	NHR2	NHR2 domain like	1	6	7	1
PF08790	zf-LYAR	LYAR-type C2HC zinc finger	1	1	1	0
PF08797	HIRAN	HIRAN domain	3	1	1	0
PF08799	PRP4	pre-mRNA processing factor 4 (PRP4) like	2	2	3	1
PF08801	NA	Nup133 N terminal like	2	2	2	0
PF08806	NA	Sep15/SelM redox domain	0	1	1	0
PF08824	Serine_rich	Serine rich protein interaction domain	1	1	6	1
PF08825	E2_bind	E2 binding domain	1	1	2	0
PF08833	NA	Axin beta-catenin binding domain	0	4	3	1
PF08839	NA	DNA replication factor CDT1 like	0	3	2	1
PF08840	BAAT_C	BAAT / Acyl-CoA thioester hydrolase C terminal	1	5	7	2
PF08880	NA	QLQ	1	2	1	0
PF08892	NA	YqcI/YcgG family	1	0	1	0
PF08894	NA	Protein of unknown function (DUF1838)	2	0	1	0
PF08910	NA	Aida N-terminus	0	0	2	0
PF08911	NUP50	NUP50 (Nucleoporin 50 kDa)	1	1	4	0
PF08912	NA	Rho Binding	1	3	2	0
PF08913	VBS	Vinculin Binding Site	1	1	2	0
PF08914	NA	Rap1 Myb domain	1	1	1	0
PF08916	NA	Phenylalanine zipper	0	1	1	1
PF08919	NA	F-actin binding	0	3	2	0
PF08920	SF3b1	Splicing factor 3B subunit 1	1	2	2	0
PF08923	MAPKK1_Int	Mitogen-activated protein kinase kinase 1 interacting	1	2	1	0
PF08925	NA	Domain of Unknown Function (DUF1907)	1	2	1	0
PF08926	DUF1908	Domain of unknown function (DUF1908)	1	7	5	1
PF08934	Rb_C	Rb C-terminal domain	0	1	1	0
PF08938	NA	HBS1 N-terminus	0	3	2	1
PF08939	NA	Domain of unknown function (DUF1917)	0	0	2	0
PF08941	NA	USP8 interacting	1	1	2	1
PF08945	Bclx_interact	Bcl-x interacting, BH3 domain	1	0	1	0
PF08952	DUF1866	Domain of unknown function (DUF1866)	1	1	1	1
PF08953	NA	Domain of unknown function (DUF1899)	3	4	16	1
PF08961	NA	Domain of unknown function (DUF1875)	1	0	0	0
PF08969	NA	USP8 dimerisation domain	2	2	3	1
PF08976	EF-hand_11	EF-hand domain	2	2	8	2
PF08991	NA	Mature-T-Cell Proliferation I type	1	2	0	0
PF08996	zf-DNA_Pol	DNA Polymerase alpha zinc finger	1	1	3	0

PF09005	NA	Domain of unknown function (DUF1897)	0	7	5	0
PF09011	HMG_box_2	HMG-box domain	4	3	14	0
PF09027	NA	GTPase binding	1	2	0	0
PF09029	NA	5-aminolevulinate synthase presequence	0	1	6	1
PF09030	Creb_binding	Creb binding	0	0	4	0
PF09032	Siah-Interact_N	Siah interacting protein, N terminal	1	1	2	0
PF09033	DFF-C	DNA Fragmentation factor 45kDa, C terminal domain	1	1	1	0
PF09038	53-BP1_Tudor	Tumour suppressor p53-binding protein-1 Tudor	1	1	3	1
PF09045	NA	L27_2	1	5	7	0
PF09056	Phospholip_A2_3	Prokaryotic phospholipase A2	4	0	2	0
PF09057	Smac_DIABLO	Second Mitochondria-derived Activator of Caspases	1	1	1	0
PF09058	L27_1	L27_1	1	4	1	0
PF09060	NA	L27_N	1	0	5	1
PF09066	B2-adapt-app_C	Beta2-adaptin appendage, C-terminal sub-domain	1	1	1	0
PF09068	EF-hand_2	EF hand	2	4	10	1
PF09069	EF-hand_3	EF-hand	2	3	10	0
PF09070	PFU	PFU (PLAA family ubiquitin binding)	1	1	1	0
PF09073	NA	BUD22	0	0	2	0
PF09079	Cdc6_C	CDC6, C terminal	2	3	3	0
PF09088	NA	MIF4G like	1	1	1	0
PF09090	NA	MIF4G like	1	1	1	1
PF09103	BRCA-2_OB1	BRCA2, oligonucleotide/oligosaccharide-binding, domain 1	0	1	1	0
PF09104	BRCA-2_OB3	BRCA2, oligonucleotide/oligosaccharide-binding, domain 3	0	1	1	0
PF09110	HAND	HAND	1	1	2	1
PF09111	SLIDE	SLIDE	1	2	2	0
PF09112	N-glycanase_N	Peptide-N-glycosidase F, N terminal	0	0	1	0
PF09113	N-glycanase_C	Peptide-N-glycosidase F, C terminal	0	0	1	0
PF09121	Tower	Tower	0	1	1	0
PF09127	Leuk-A4-hydro_C	Leukotriene A4 hydrolase, C-terminal	2	4	5	0
PF09128	RGS-like	Regulator of G protein signalling-like domain	0	15	29	1
PF09129	Chol_subst-bind	Cholesterol oxidase, substrate-binding	0	0	1	0
PF09133	NA	SANTA (SANT Associated)	1	2	1	0
PF09138	Urm1	Urm1 (Ubiquitin related modifier)	1	1	1	0
PF09139	Tam41_Mmp37	Mitochondrial matrix Mmp37	1	1	1	1
PF09141	Talin_middle	Talin, middle domain	1	1	11	0
PF09162	Tap-RNA_bind	Tap, RNA-binding	1	2	1	0
PF09169	BRCA-2_helical	BRCA2, helical	1	1	1	0
PF09170	STN1_2	CST, Suppressor of cdc thirteen homolog, complex subunit STN1	0	1	1	0
PF09172	NA	Domain of unknown function (DUF1943)	2	5	8	9
PF09173	eIF2_C	Initiation factor eIF2 gamma, C terminal	1	1	1	0
PF09174	Maf1	Maf1 regulator	1	1	3	0
PF09177	Syntaxin-6_N	Syntaxin 6, N-terminal	1	2	2	0
PF09180	ProRS-C_1	Prolyl-tRNA synthetase, C-terminal	1	2	2	0
PF09184	NA	PPP4R2	1	1	1	0
PF09190	DALR_2	DALR domain	0	2	1	0
PF09202	Rio2_N	Rio2, N-terminal	1	1	2	0
PF09229	NA	Activator of Hsp90 ATPase, N-terminal	1	1	1	0
PF09230	NA	DNA fragmentation factor 40 kDa	1	1	2	2
PF09237	GAGA	GAGA factor	0	1	0	0
PF09243	NA	Mitochondrial small ribosomal subunit Rsm22	2	2	1	0
PF09247	TBP-binding	TATA box-binding protein binding	1	1	1	0
PF09248	DUF1965	Domain of unknown function (DUF1965)	0	1	0	1
PF09258	Glyco_transf_64	Glycosyl transferase family 64 domain	1	4	5	1
PF09261	Alpha-mann_mid	Alpha mannosidase, middle domain	6	13	18	3
PF09262	NA	Peroxisome biogenesis factor 1, N-terminal	0	4	1	0
PF09263	NA	Peroxisome biogenesis factor 1, N-terminal	0	0	1	0
PF09268	Clathrin-link	Clathrin, heavy-chain linker	1	1	4	1
PF09270	BTD	Beta-trefoil DNA-binding domain	1	1	3	0
PF09271	LAG1-DNAbind	LAG1, DNA binding	1	1	3	0
PF09273	Rubis-subs-bind	Rubisco LSMT substrate-binding	2	1	2	1
PF09279	EF-hand_like	Phosphoinositide-specific phospholipase C, efhand-like	5	12	24	1
PF09280	XPC-binding	XPC-binding domain	1	1	1	0
PF09282	NA	Mago binding	1	1	1	0
PF09285	Elong-fact-P_C	Elongation factor P, C-terminal	0	1	0	0
PF09292	Neil1-DNA_bind	Endonuclease VIII-like 1, DNA bind	1	3	1	0
PF09296	NA	NADH pyrophosphatase-like rudimentary NUDIX domain	1	2	2	1
PF09297	zf-NADH-PPase	NADH pyrophosphatase zinc ribbon domain	1	2	2	1
PF09298	FAA_hydrolase_N	Fumarylacetoacetase N-terminal	1	1	1	0
PF09302	XLF	XLF-Cernunnos, XRcc4-like factor, NHEJ component	1	1	1	0
PF09309	FCP1_C	FCP1, C-terminal	0	1	0	1
PF09311	Rab5-bind	Rabaptin-like protein	1	2	2	2
PF09316	NA	C-myb, C-terminal	0	2	2	0
PF09320	NA	Domain of unknown function (DUF1977)	1	1	1	0
PF09324	NA	Domain of unknown function (DUF1981)	3	7	10	1
PF09325	NA	Vps5 C terminal like	3	4	5	1
PF09326	NADH_dhqG_C	NADH-ubiquinone oxidoreductase subunit G, C-terminal	1	3	1	0
PF09329	NA	Primase zinc finger	1	1	1	0

PF09332	NA	Mcm10 replication factor	1	1	1	0
PF09333	NA	Autophagy-related protein C terminal domain	2	2	3	1
PF09334	tRNA-synt_1g	tRNA synthetases class I (M)	3	9	4	0
PF09335	NA	SNARE associated Golgi protein	2	5	9	0
PF09336	Vps4_C	Vps4 C terminal oligomerisation domain	3	10	12	1
PF09337	NA	His(2)-Cys(2) zinc finger	0	1	13	0
PF09340	NuA4	Histone acetyltransferase subunit NuA4	1	1	2	0
PF09341	Pcc1	Transcription factor Pcc1	1	1	0	0
PF09346	SMI1_KNR4	SMI1 / KNR4 family (SUKH-1)	1	0	0	0
PF09349	OHCU_decarbox	OHCU decarboxylase	0	0	3	0
PF09350	NA	Domain of unknown function (DUF1992)	2	0	2	0
PF09354	NA	HNF3 C-terminal domain	1	1	0	1
PF09358	E1_UFD	Ubiquitin fold domain	2	3	9	0
PF09360	zf-CDGSH	Iron-binding zinc finger CDGSH type	2	3	4	0
PF09368	Sas10	Sas10 C-terminal domain	1	2	1	0
PF09371	NA	Tex-like protein N-terminal domain	1	1	0	0
PF09377	SBDS_C	SBDS protein C-terminal domain	1	1	1	0
PF09379	FERM_N	FERM N-terminal domain	14	41	61	1
PF09380	FERM_C	FERM C-terminal PH-like domain	13	41	51	1
PF09382	RQC	RQC domain	2	4	4	1
PF09384	UTP15_C	UTP15 C terminal	1	1	1	0
PF09398	FOP_dimer	FOP N terminal dimerisation domain	2	11	5	0
PF09402	NA	Man1-Src1p-C-terminal domain	1	2	0	0
PF09404	NA	Eukaryotic protein of unknown function (DUF2003)	2	3	3	1
PF09405	Btz	CASC3/Barentsz eIF4AIII binding	1	1	6	1
PF09409	PUB	PUB domain	3	1	5	1
PF09412	NA	Endoribonuclease XendoU	4	2	3	1
PF09415	CENP-X	CENP-S associating Centromere protein X	0	3	1	0
PF09416	UPF1_Zn_bind	RNA helicase (UPF2 interacting domain)	0	1	2	1
PF09420	Nop16	Ribosome biogenesis protein Nop16	1	3	1	0
PF09429	NA	WW domain binding protein 11	1	1	1	0
PF09430	NA	Protein of unknown function (DUF2012)	1	1	2	0
PF09431	NA	Protein of unknown function (DUF2013)	0	1	1	0
PF09439	SRPRB	Signal recognition particle receptor beta subunit	1	1	1	1
PF09440	eIF3_N	eIF3 subunit 6 N terminal domain	1	1	1	0
PF09443	NA	Cripto_Frl-1_Cryptic (CFC)	3	2	1	0
PF09445	Methyltransf_15	RNA cap guanine-N2 methyltransferase	1	2	1	0
PF09446	NA	VMA21-like domain	1	0	1	0
PF09451	NA	Autophagy-related protein 27	2	4	1	0
PF09453	HIRA_B	HIRA B motif	1	2	0	0
PF09454	Vps23_core	Vps23 core domain	1	1	1	0
PF09457	RBD-FIP	FIP domain	0	7	5	0
PF09458	H_lectin	H-type lectin domain	1	8	15	6
PF09468	RNase_H2-Ydr279	Ydr279p protein family (RNase H2 complex component)	1	1	1	0
PF09469	NA	Cordon-bleu ubiquitin-like domain	0	1	0	1
PF09494	NA	Slx4 endonuclease	1	0	1	0
PF09495	NA	Protein of unknown function (DUF2462)	0	0	1	0
PF09496	CENP-O	Cenp-O kinetochore centromere component	0	0	1	0
PF09497	NA	Transcription mediator complex subunit Med12	1	3	3	0
PF09507	CDC27	DNA polymerase subunit Cdc27	1	2	3	1
PF09531	NA	Nucleoporin protein Ndc1-Nup	1	1	1	0
PF09532	FDF	FDF domain	0	2	7	1
PF09588	YqaJ	YqaJ-like viral recombinase domain	12	1	55	13
PF09596	NA	MamL-1 domain	0	0	1	0
PF09606	NA	ARC105 or Med15 subunit of Mediator complex non-fungal	1	6	2	1
PF09607	BrkDBD	Brinker DNA-binding domain	1	1	2	0
PF09612	NA	Bacterial protein of unknown function (HtrL_YibB)	3	22	15	10
PF09631	NA	Sen15 protein	1	4	1	0
PF09637	Med18	Med18 protein	1	1	3	0
PF09646	NA	Gp37 protein	0	0	0	1
PF09668	Asp_protease	Aspartyl protease	0	3	2	0
PF09696	Ctf8	Ctf8	1	3	1	0
PF09724	Dcc1	Uncharacterized conserved protein (DUF2036)	1	1	1	0
PF09725	NA	Folate-sensitive fragile site protein Fra10Ac1	1	0	1	0
PF09726	NA	Transmembrane protein	1	1	1	2
PF09727	NA	Cortactin-binding protein-2	1	1	1	0
PF09728	NA	Myosin-like coiled-coil protein	1	2	3	1
PF09730	BicD	Microtubule-associated protein Bicaudal-D	1	1	2	3
PF09731	NA	Mitochondrial inner membrane protein	1	1	2	3
PF09732	NA	Cactus-binding C-terminus of cactin protein	1	1	1	0
PF09733	VEFS-Box	VEFS-Box of polycomb protein	1	2	2	0
PF09734	NA	RNA polymerase III transcription factor (TF)IIIC subunit	1	1	1	0
PF09735	NA	Membrane-associated apoptosis protein	2	5	28	0
PF09736	Bud13	Pre-mRNA-splicing factor of RES complex	0	1	1	0
PF09737	NA	De-etiolated protein 1 Det1	1	1	2	1
PF09738	NA	Double stranded RNA binding protein (DUF2051)	1	1	1	1
PF09739	NA	Mini-chromosome maintenance replisome factor	1	1	1	0

PF09740	NA	Uncharacterized conserved protein (DUF2043)	1	1	3	0
PF09741	NA	Uncharacterized conserved protein (DUF2045)	1	5	1	0
PF09742	NA	Dyggve-Melchior-Clausen syndrome protein	3	4	1	1
PF09743	NA	Uncharacterized conserved protein (DUF2042)	1	1	3	1
PF09744	NA	JNK_SAPK-associated protein-1	2	2	18	2
PF09745	NA	Coiled-coil domain-containing protein 55 (DUF2040)	1	1	2	0
PF09746	NA	Tumour-associated protein	1	3	1	0
PF09747	NA	Coiled-coil domain containing protein (DUF2052)	1	2	1	1
PF09748	Med10	Transcription factor subunit Med10 of Mediator complex	1	1	1	0
PF09749	HVSL	Uncharacterised conserved protein	1	1	1	0
PF09750	NA	Alternative splicing regulator	2	3	2	0
PF09751	NA	Nuclear protein Es2	1	1	1	1
PF09752	NA	Uncharacterized conserved protein (DUF2048)	1	4	2	1
PF09753	NA	Membrane fusion protein Use1	1	2	1	0
PF09754	PAC2	PAC2 family	1	1	1	0
PF09755	NA	Uncharacterized conserved protein H4 (DUF2046)	0	1	1	1
PF09756	DDRGK	DDRGK domain	1	2	1	1
PF09757	NA	Arb2 domain	1	0	2	0
PF09758	NA	Uncharacterised conserved protein	1	2	3	0
PF09759	NA	Spinocerebellar ataxia type 10 protein domain	1	0	1	1
PF09762	NA	Coiled-coil domain-containing protein (DUF2037)	1	1	1	0
PF09763	Sec3_C	Exocyst complex component Sec3	1	1	1	0
PF09764	Nt_Gln_amidase	N-terminal glutamine amidase	1	1	2	0
PF09765	WD-3	WD-repeat region	1	1	1	0
PF09766	NA	Fms-interacting protein	1	1	4	0
PF09767	NA	Predicted membrane protein (DUF2053)	1	1	2	0
PF09768	NA	Peptidase M76 family	0	1	1	0
PF09769	NA	Apolipoprotein O	1	1	0	0
PF09770	PAT1	Topoisomerase II-associated protein PAT1	1	2	3	0
PF09771	NA	Transmembrane protein 188	1	1	1	0
PF09772	NA	Transmembrane protein 26	5	5	14	17
PF09773	NA	Meckelin (Transmembrane protein 67)	1	2	2	2
PF09774	NA	Caffeine-induced death protein 2	1	1	1	0
PF09775	NA	Keratinocyte-associated protein 2	1	2	1	0
PF09776	Mitoc_L55	Mitochondrial ribosomal protein L55	1	2	2	0
PF09777	NA	Osteopetrosis-associated transmembrane protein 1 precursor	1	1	1	0
PF09778	NA	Guanylylate cyclase	1	1	1	0
PF09779	NA	Ima1 N-terminal domain	0	2	1	0
PF09781	NDUF_B5	NADH:ubiquinone oxidoreductase, NDUFB5/SGDH subunit	1	2	1	0
PF09782	NDUF_B6	NADH:ubiquinone oxidoreductase, NDUFB6/B17 subunit	1	1	1	1
PF09783	Vac_ImportDeg	Vacuolar import and degradation protein	1	1	1	0
PF09784	NA	Mitochondrial ribosomal protein L31	1	0	0	0
PF09785	Prp31_C	Prp31 C terminal domain	1	1	2	0
PF09786	NA	Cytochrome B561, N terminal	1	1	2	0
PF09787	NA	Golgin subfamily A member 5	2	4	4	0
PF09788	NA	Transmembrane protein 55A	1	1	2	0
PF09789	NA	Uncharacterized coiled-coil protein (DUF2353)	1	6	2	0
PF09790	Hyccin	Hyccin	3	2	1	0
PF09791	NA	Oxidoreductase-like protein, N-terminal	1	1	1	2
PF09793	NA	Anticodon-binding domain	1	2	1	0
PF09794	NA	Transport protein Avl9	2	4	3	0
PF09797	NA	N-acetyltransferase B complex (NatB) non catalytic subunit	1	1	1	0
PF09799	NA	Predicted membrane protein	1	4	2	0
PF09801	NA	Integral membrane protein S linking to the trans Golgi network	2	1	1	0
PF09803	NA	Uncharacterized conserved protein (DUF2346)	0	1	0	0
PF09804	NA	Uncharacterized conserved protein (DUF2347)	1	1	1	0
PF09805	NA	Nucleolar protein 12 (25kDa)	1	1	2	0
PF09806	CDK2AP	Cyclin-dependent kinase 2-associated protein	1	2	0	1
PF09807	NA	Elongation complex protein 6	1	1	1	1
PF09808	NA	Small nuclear RNA activating complex (SNAPc), subunit SNAP43	1	1	1	0
PF09809	MRP-L27	Mitochondrial ribosomal protein L27	1	1	1	0
PF09810	NA	Exonuclease V - a 5' deoxyribonuclease	1	0	1	0
PF09811	NA	Essential protein Yae1, N terminal	0	1	0	0
PF09812	MRP-L28	Mitochondrial ribosomal protein L28	1	2	1	0
PF09813	NA	Coiled-coil domain-containing protein 56	0	1	0	0
PF09814	NA	HECT-like Ubiquitin-conjugating enzyme (E2)-binding	1	1	1	0
PF09815	NA	XK-related protein	4	7	24	8
PF09816	NA	RNA polymerase II transcription elongation factor	1	4	1	0
PF09817	Zwilch	Uncharacterized conserved protein (DUF2352)	1	1	1	2
PF09822	NA	ABC-type uncharacterized transport system	1	0	1	0
PF09825	NA	Biotin-protein ligase, N terminal	2	2	1	1
PF09837	NA	Uncharacterized protein conserved in bacteria (DUF2064)	0	0	1	0
PF09848	NA	Uncharacterized conserved protein (DUF2075)	0	1	0	0
PF09992	NAGPA	Predicted periplasmic protein (DUF2233)	1	1	1	0
PF09995	DUF2236	Uncharacterized protein conserved in bacteria (DUF2236)	1	3	2	1
PF10017	Methyltransf_33	Histidine-specific methyltransferase, SAM-dependent	0	0	4	0
PF10018	Med4	Vitamin-D-receptor interacting Mediator subunit 4	1	1	2	0

PF10033	ATG13	Autophagy-related protein 13	1	0	1	0
PF10034	NA	Q-cell neuroblast polarisation	2	4	2	0
PF10036	NA	Putative carnitine deficiency-associated protein	1	1	2	0
PF10037	MRP-S27	Mitochondrial 28S ribosomal protein S27	1	1	1	2
PF10044	NA	Retinal tissue protein	1	1	1	0
PF10046	NA	Biogenesis of lysosome-related organelles complex-1 subunit 2	1	1	1	0
PF10058	NA	Predicted integral membrane zinc-ribbon metal-binding protein	1	0	2	0
PF10075	CSN8_PSD8_EIF3K	CSN8/PSMD8/EIF3K family	3	3	3	0
PF10127	NA	Predicted nucleotidyltransferase	2	5	2	0
PF10142	NA	PhoPQ-activated pathogenicity-related protein	2	10	1	2
PF10146	NA	Zinc finger-containing protein	1	1	1	0
PF10147	CR6_interact	Growth arrest and DNA-damage-inducible proteins-interacting protein 1	1	1	1	1
PF10148	NA	Schwannomin-interacting protein 1	1	4	1	1
PF10149	NA	Transmembrane protein 231	1	1	1	0
PF10151	NA	Uncharacterised conserved protein (DUF2359)	1	2	1	1
PF10152	NA	Predicted coiled-coil domain-containing protein (DUF2360)	1	1	1	0
PF10154	NA	Uncharacterized conserved protein (DUF2362)	1	2	7	0
PF10155	NA	Uncharacterized conserved protein (DUF2363)	1	1	0	0
PF10156	Med17	Subunit 17 of Mediator complex	1	0	2	0
PF10157	NA	Uncharacterized conserved protein (DUF2365)	1	3	2	0
PF10158	NA	Tumour suppressor protein	1	5	1	0
PF10160	NA	Predicted membrane protein	1	1	1	0
PF10161	DDDD	Putative mitochondrial precursor protein	2	0	0	1
PF10162	NA	G8 domain	5	2	13	3
PF10163	EnY2	Transcription factor e(y)2	1	1	1	0
PF10164	NA	Uncharacterized conserved protein (DUF2367)	1	2	2	1
PF10165	NA	Guanine nucleotide exchange factor synembryn	1	2	1	1
PF10166	NA	Uncharacterised conserved protein (DUF2368)	1	1	2	0
PF10167	NA	Uncharacterised conserved protein	1	3	4	0
PF10168	NA	Nuclear pore component	1	1	1	2
PF10169	NA	Learning-associated protein	1	1	1	0
PF10170	NA	Cysteine-rich domain	0	2	1	0
PF10171	NA	Uncharacterised conserved protein (DUF2366)	1	1	1	1
PF10172	DDA1	Det1 complexing ubiquitin ligase	1	1	1	0
PF10174	NA	RIM-binding protein of the cytomatrix active zone	3	1	1	2
PF10175	MPP6	M-phase phosphoprotein 6	1	0	1	0
PF10176	NA	Protein of unknown function (DUF2370)	1	0	1	0
PF10177	NA	Uncharacterised conserved protein (DUF2371)	1	0	4	0
PF10178	PAC3	Proteasome assembly chaperone 3	0	1	1	0
PF10179	NA	Uncharacterised conserved protein (DUF2369)	1	1	6	1
PF10180	NA	Uncharacterised conserved protein (DUF2373)	1	2	1	0
PF10181	NA	GPI-GlcNAc transferase complex, PIG-H component	1	1	2	0
PF10183	ESSS	ESSS subunit of NADH:ubiquinone oxidoreductase (complex I)	1	1	1	0
PF10184	NA	Uncharacterized conserved protein (DUF2358)	2	3	1	1
PF10185	Mesd	Chaperone for wingless signalling and trafficking of LDL receptor	1	1	2	0
PF10186	NA	Vacuolar sorting 38 and autophagy-related subunit 14	2	2	1	0
PF10187	NA	N-terminal domain of NEFA-interacting nuclear protein NIP30	1	1	2	0
PF10188	NA	Organic solute transport protein 1	1	2	3	0
PF10189	NA	Conserved protein (DUF2356)	1	2	2	0
PF10190	NA	Putative transmembrane protein 170	1	0	1	0
PF10191	NA	Golgi complex component 7 (COG7)	1	2	1	0
PF10192	NA	Rhodopsin-like GPCR transmembrane domain	3	8	6	2
PF10193	NA	Telomere length regulation protein	1	1	0	0
PF10195	NA	DNA-binding nuclear phosphoprotein p8	0	0	1	1
PF10197	NA	N-terminal domain of CBF1 interacting co-repressor CIR	2	2	3	0
PF10198	NA	Histone acetyltransferases subunit 3	1	1	1	1
PF10199	NA	Alpha and gamma adaptin binding protein p34	1	2	1	0
PF10200	Ndufs5	NADH:ubiquinone oxidoreductase, NDUFS5-15kDa	1	2	0	1
PF10203	NA	Cytochrome c oxidase assembly protein PET191	1	1	1	0
PF10204	NA	Dual oxidase maturation factor	2	3	8	1
PF10205	NA	Predicted coiled-coil domain-containing protein	1	1	1	0
PF10206	WRW	Mitochondrial F1F0-ATP synthase, subunit f	1	1	1	0
PF10208	Armet	Degradation arginine-rich protein for mis-folding	1	3	1	0
PF10209	NA	Uncharacterized conserved protein (DUF2340)	0	0	1	0
PF10210	MRP-S32	Mitochondrial 28S ribosomal protein S32	1	1	0	0
PF10211	NA	Axonemal dynein light chain	2	2	4	3
PF10212	NA	Predicted coiled-coil domain-containing protein	1	1	1	1
PF10213	MRP-S28	Mitochondrial ribosomal subunit protein	1	1	1	0
PF10217	NA	Uncharacterized conserved protein (DUF2039)	1	3	1	0
PF10218	NA	Uncharacterized conserved protein (DUF2054)	1	1	1	0
PF10220	Smg8_Smg9	Uncharacterized conserved protein (DUF2146)	1	1	2	1
PF10221	NA	Cell cycle and development regulator	2	2	6	0
PF10222	NA	Uncharacterized conserved protein (DUF2152)	1	2	3	2
PF10223	NA	Uncharacterized conserved protein (DUF2181)	1	4	1	3
PF10224	DUF2205	Predicted coiled-coil protein (DUF2205)	1	4	2	0
PF10225	NA	Uncharacterized conserved protein (DUF2215)	1	1	2	1
PF10226	NA	Uncharacterized conserved proteins (DUF2216)	1	3	2	1

PF10228	DUF2228	Uncharacterised conserved protein (DUF2228)	1	1	1	1
PF10229	MMADHC	Uncharacterized conserved protein (DUF2246)	1	1	2	0
PF10230	NA	Uncharacterised conserved protein (DUF2305)	1	2	3	1
PF10231	NA	Uncharacterised conserved protein (DUF2315)	1	1	1	0
PF10232	Med8	Mediator of RNA polymerase II transcription complex subunit 8	1	3	2	0
PF10233	NA	Uncharacterized conserved protein CG6151-P	1	1	1	0
PF10234	NA	Clusterin-associated protein-1	1	2	2	1
PF10235	NA	Microtubule-associated protein CRIPT	1	1	1	0
PF10236	DAP3	Mitochondrial ribosomal death-associated protein 3	1	1	3	0
PF10237	NA	Probable N6-adenine methyltransferase	1	6	1	0
PF10238	NA	E2F-associated phosphoprotein	1	2	1	1
PF10239	NA	Protein of unknown function (DUF2465)	1	1	1	0
PF10240	DUF2464	Protein of unknown function (DUF2464)	2	1	3	0
PF10241	NA	Uncharacterized conserved protein	1	2	1	0
PF10242	L_HMGIC_fpl	Lipoma HMGIC fusion partner-like protein	6	6	11	11
PF10243	MIP-T3	Microtubule-binding protein MIP-T3	1	1	2	2
PF10244	MRP-L51	Mitochondrial ribosomal subunit	1	1	1	0
PF10245	MRP-S22	Mitochondrial 28S ribosomal protein S22	1	1	2	1
PF10246	MRP-S35	Mitochondrial ribosomal protein MRP-S35	1	1	0	1
PF10247	NA	Reactive mitochondrial oxygen species modulator 1	1	1	0	0
PF10248	NA	Myelodysplasia-myeloid leukemia factor 1-interacting protein	2	4	7	0
PF10249	NDUFB10	NADH-ubiquinone oxidoreductase subunit 10	1	1	1	0
PF10250	O-FucT	GDP-fucose protein O-fucosyltransferase	3	2	7	1
PF10251	PEN-2	Presenilin enhancer-2 subunit of gamma secretase	1	1	1	0
PF10252	NA	Casein kinase substrate phosphoprotein PP28	1	1	2	0
PF10253	NA	Mitotic checkpoint regulator, MAD2B-interacting	1	1	2	0
PF10254	NA	PACS-1 cytosolic sorting protein	1	4	5	1
PF10255	Paf67	RNA polymerase I-associated factor PAF67	1	1	1	1
PF10256	NA	Golgin subfamily A member 7/ERF4 family	2	5	2	0
PF10257	NA	Retinoic acid induced 16-like protein	3	3	11	2
PF10258	RNA_GG_bind	PHAX RNA-binding domain	1	1	1	0
PF10259	NA	Rogdi leucine zipper containing protein	1	1	1	0
PF10260	NA	Uncharacterized conserved domain (SAYSvFN)	1	1	1	0
PF10261	NA	Inositol phospholipid synthesis and fat-storage-inducing TM	1	1	1	1
PF10262	Rdx	Rdx family	1	0	1	0
PF10263	NA	SprT-like family	2	2	3	1
PF10264	NA	Winged helix Storkhead-box1 domain	1	1	3	1
PF10265	NA	Uncharacterized conserved protein (DUF2217)	1	2	1	0
PF10266	NA	Hereditary spastic paraplegia protein strumpellin	2	1	0	0
PF10267	NA	Predicted transmembrane and coiled-coil 2 protein	1	1	16	2
PF10268	NA	Predicted transmembrane protein 161AB	1	1	2	1
PF10269	NA	Transmembrane Fragile-X-F protein	3	3	4	0
PF10270	MMgT	Membrane magnesium transporter	1	1	1	0
PF10271	NA	Putative transmembrane protein	1	1	5	2
PF10272	NA	Putative transmembrane protein precursor	1	1	1	0
PF10273	WGG	Pre-rRNA-processing protein TSR2	1	1	1	0
PF10274	ParcG	Parkin co-regulated protein	2	3	2	0
PF10275	Peptidase_C65	Peptidase C65 Otubain	1	1	1	1
PF10276	zf-CHCC	Zinc-finger domain	1	2	1	0
PF10277	NA	Frag1/DRAM/Sfk1 family	9	5	30	0
PF10278	NA	Mediator of RNA pol II transcription subunit 19	1	2	2	0
PF10280	Med11	Mediator complex protein	1	1	1	0
PF10283	zf-CCHH	Zinc-finger (CX5CX6HX5H) motif	5	5	12	0
PF10288	NA	Cytoplasmic tRNA 2-thiolation protein 2	1	0	2	0
PF10291	muHD	Muniscin C-terminal mu homology domain	1	1	4	0
PF10293	NA	Domain of unknown function (DUF2405)	1	0	1	0
PF10294	Methyltransf_16	Lysine methyltransferase	6	11	10	0
PF10296	MMM1	Maintenance of mitochondrial morphology protein 1	1	0	0	0
PF10300	NA	Protein of unknown function (DUF3808)	3	3	2	1
PF10304	NA	Required for nuclear transport of RNA pol II C-terminus 2	1	0	0	0
PF10309	NA	Protein of unknown function (DUF2414)	1	1	1	0
PF10312	NA	Conserved mid region of cactin	1	1	1	1
PF10316	NA	Serpentine type 7TM GPCR chemoreceptor Srbc	0	0	0	1
PF10318	NA	Serpentine type 7TM GPCR chemoreceptor Srh	0	0	2	0
PF10320	NA	Serpentine type 7TM GPCR chemoreceptor Srsx	0	3	0	2
PF10321	NA	Serpentine type 7TM GPCR chemoreceptor Srt	0	2	0	0
PF10323	NA	Serpentine type 7TM GPCR chemoreceptor Srv	0	0	0	3
PF10324	NA	Serpentine type 7TM GPCR chemoreceptor Srw	26	196	87	151
PF10328	NA	Serpentine type 7TM GPCR chemoreceptor Srx	0	18	0	3
PF10341	TPP1	Shelterin complex subunit, TPP1/ACD	1	0	2	0
PF10343	NA	Potential Queuosine, Q, salvage protein family	1	9	2	0
PF10345	NA	Cohesin loading factor	1	1	1	0
PF10347	NA	RNA pol II promoter Fmp27 protein domain	1	1	1	1
PF10349	NA	WW-domain ligand protein	1	1	1	0
PF10350	NA	Putative death-receptor fusion protein (DUF2428)	2	2	4	1
PF10351	NA	Golgi-body localisation protein domain	1	1	1	0
PF10354	NA	Domain of unknown function (DUF2431)	1	0	1	1

PF10357	NA	Domain of Kin17 curved DNA-binding protein	2	1	2	0
PF10358	NA	N-terminal C2 in EEIG1 and EHBP1 proteins	2	7	6	1
PF10363	NA	Required for nuclear transport of RNA pol II C-terminus 1	1	1	1	1
PF10366	NA	Vacuolar sorting protein 39 domain 1	2	2	2	0
PF10367	NA	Vacuolar sorting protein 39 domain 2	4	3	5	0
PF10368	YkyA	Putative cell-wall binding lipoprotein	0	0	4	0
PF10373	EST1_DNA_bind	Est1 DNA/RNA binding domain	2	4	5	1
PF10374	EST1	Telomerase activating protein Est1	3	4	5	0
PF10376	Mei5	Double-strand recombination repair protein	1	2	1	0
PF10377	ATG11	Autophagy-related protein 11	1	2	1	0
PF10381	Autophagy_C	Autophagocytosis associated protein C-terminal	1	1	1	0
PF10382	NA	Protein of unknown function (DUF2439)	0	0	3	0
PF10384	Scm3	Centromere protein Scm3	0	1	0	0
PF10385	RNA_pol_Rpb2_45	RNA polymerase beta subunit external 1 domain	0	1	0	0
PF10390	NA	RNA polymerase II elongation factor ELL	1	1	1	0
PF10391	DNA_pol_lambd_f	Fingers domain of DNA polymerase lambda	2	6	3	0
PF10392	NA	Golgi transport complex subunit 5	1	1	1	0
PF10394	Hat1_N	Histone acetyl transferase HAT1 N-terminus	1	1	1	0
PF10395	Utp8	Utp8 family	0	0	1	0
PF10396	NA	GTP-binding protein TrmE N-terminus	1	3	1	0
PF10397	ADSL_C	Adenylosuccinate lyase C-terminus	1	4	1	0
PF10401	IRF-3	Interferon-regulatory factor 3	1	12	3	1
PF10403	BHD_1	Rad4 beta-hairpin domain 1	1	1	1	0
PF10404	NA	Rad4 beta-hairpin domain 2	1	1	1	0
PF10405	BHD_3	Rad4 beta-hairpin domain 3	1	1	1	0
PF10406	NA	Transcription factor TFIID complex subunit 8 C-term	1	1	1	0
PF10408	Ufd2P_core	Ubiquitin elongating factor core	3	6	2	5
PF10409	PTEN_C2	C2 domain of PTEN tumour-suppressor protein	4	4	13	1
PF10415	FumaraseC_C	Fumarase C C-terminus	1	4	2	1
PF10417	1-cysPrx_C	C-terminal domain of 1-Cys peroxiredoxin	3	3	3	0
PF10419	TFIIIC_sub6	TFIIIC subunit	1	4	2	0
PF10421	OAS1_C	2'-5'-oligoadenylate synthetase 1, domain 2, C-terminus	2	0	4	0
PF10431	ClpB_D2-small	C-terminal, D2-small domain, of ClpB protein	2	4	4	0
PF10433	MMS1_N	Mono-functional DNA-alkylating methyl methanesulfonate N-term	3	3	4	0
PF10436	BCDHK_Adom3	Mitochondrial branched-chain alpha-ketoacid dehydrogenase kinase	2	5	5	0
PF10441	NA	Urb2/Npa2 family	1	0	1	0
PF10442	NA	FIST C domain	1	1	1	1
PF10444	Nbl1_Borealin_N	Nbl1 / Borealin N terminal	1	0	1	0
PF10447	EXOSC1	Exosome component EXOSC1/CSL4	1	1	1	0
PF10451	Stn1	Telomere regulation protein Stn1	0	0	1	0
PF10453	NUFIP1	Nuclear fragile X mental retardation-interacting protein 1 (NUFIP1)	1	0	1	0
PF10456	BAR_3_WASP_bdg	WASP-binding domain of Sorting nexin protein	1	1	7	1
PF10457	NA	Cholesterol-capturing domain	1	1	3	1
PF10458	Val_tRNA-synt_C	Valyl tRNA synthetase tRNA binding arm	0	0	3	0
PF10469	AKAP7_NLS	AKAP7 2'5' RNA ligase-like domain	3	10	7	1
PF10471	ANAPC_CDC26	Anaphase-promoting complex APC subunit CDC26	1	0	1	0
PF10473	NA	Leucine-rich repeats of kinetochore protein Cenp-F/LEK1	1	0	0	0
PF10474	NA	Protein of unknown function C-terminus (DUF2451)	1	1	2	0
PF10475	NA	Protein of unknown function N-terminal domain (DUF2450)	2	2	3	0
PF10477	EIF4E-T	Nucleocytoplasmic shuttling protein for mRNA cap-binding EIF4E	1	4	3	0
PF10479	NA	Fragile site-associated protein C-terminus	1	1	5	2
PF10480	NA	Beta-1 integrin binding protein	0	1	4	0
PF10481	NA	Cenp-F N-terminal domain	0	0	1	0
PF10483	Elong_Iki1	Elongator subunit Iki1	1	2	2	0
PF10484	MRP-S23	Mitochondrial ribosomal protein S23	1	5	2	0
PF10487	NA	Nucleoporin subcomplex protein binding to Pom34	2	1	1	1
PF10488	PP1c_bdg	Phosphatase-1 catalytic subunit binding region	1	0	1	0
PF10491	NA	NLS-binding and DNA-binding and dimerisation domains of Nrf1	1	4	7	0
PF10492	NA	Nrf1 activator activation site binding domain	1	0	0	0
PF10493	NA	Rough deal protein C-terminal region	1	1	1	0
PF10494	NA	Serine-threonine protein kinase 19	1	0	1	0
PF10495	NA	Pericentrin-AKAP-450 domain of centrosomal targeting protein	0	1	8	1
PF10496	NA	SNARE-complex protein Syntaxin-18 N-terminus	1	3	2	0
PF10497	NA	Zinc-finger domain of monoamine-oxidase A repressor R1	0	3	2	0
PF10498	NA	Intra-flagellar transport protein 57	1	3	1	1
PF10500	NA	Nuclear RNA-splicing-associated protein	1	1	1	0
PF10501	Ribosomal_L50	Ribosomal subunit 39S	0	1	1	0
PF10503	Esterase_phd	Esterase PHB depolymerase	0	0	1	0
PF10504	NA	Protein of unknown function (DUF2452)	1	1	1	0
PF10505	NA	NMDA receptor-regulated gene protein 2 C-terminus	1	1	1	1
PF10506	NA	PDZ domain of MCC-2 bdg protein for Usher syndrome	0	1	3	2
PF10507	NA	Protein of unknown function (DUF2453)	1	1	1	0
PF10508	NA	Proteasome non-ATPase 26S subunit	1	2	2	2
PF10509	GalKase_gal_bdg	Galactokinase galactose-binding signature	2	5	2	0
PF10510	NA	Phosphatidylinositol-glycan biosynthesis class S protein	1	1	1	0
PF10512	NA	Cell division cycle-associated protein 8	1	0	1	0
PF10513	EPL1	Enhancer of polycomb-like	2	8	8	2

PF10515	APP_amyloid	beta-amyloid precursor protein C-terminus	1	1	1	1
PF10516	NA	SHNi-TPR	1	2	3	0
PF10520	NA	B domain of TMEM189, localisation domain	1	1	1	1
PF10521	NA	Protein of unknown function (DUF2454)	1	1	2	1
PF10523	BEN	BEN domain	1	5	12	0
PF10524	NA	Nuclear factor I protein pre-N-terminus	1	6	5	0
PF10525	NA	Engrailed homeobox C-terminal signature domain	1	2	2	1
PF10531	SLBB	SLBB domain	1	2	1	0
PF10534	NA	Connector enhancer of kinase suppressor of ras	1	1	2	1
PF10537	NA	ATP-utilising chromatin assembly and remodelling N-terminal	2	2	7	0
PF10540	Membr_traf_MHD	Munc13 (mammalian uncoordinated) homology domain	2	2	19	1
PF10541	NA	Nuclear envelope localisation domain	1	5	1	0
PF10545	NA	Alcohol dehydrogenase transcription factor Myb/SANT-like	15	5	3	3
PF10551	NA	MULE transposase domain	2	4	25	1
PF10557	Cullin_Nedd8	Cullin protein neddylation domain	5	4	8	0
PF10558	NA	Mitochondrial 18 KDa protein (MTP18)	2	2	1	0
PF10559	Plug_translocon	Plug domain of Sec61p	1	1	2	0
PF10561	NA	Uncharacterised protein family UPF0565	1	2	1	1
PF10562	CaM_bdg_C0	Calmodulin-binding domain C0 of NMDA receptor NR1 subunit	1	6	1	1
PF10568	Tom37	Outer mitochondrial membrane transport complex protein	1	1	0	0
PF10569	NA	Alpha-macro-globulin thiol-ester bond-forming region	9	10	12	0
PF10571	NA	Uncharacterised protein family UPF0547	1	0	1	0
PF10572	UPF0556	Uncharacterised protein family UPF0556	1	1	1	0
PF10573	NA	Uncharacterised protein family UPF0561	1	0	1	0
PF10574	NA	Uncharacterised protein family UPF0552	1	1	2	0
PF10576	EndIII_4Fe-2S	Iron-sulfur binding domain of endonuclease III	1	0	0	0
PF10579	NA	Rapsyn N-terminal myristoylation and linker region	1	2	2	2
PF10584	Proteasome_A_N	Proteasome subunit A N-terminal signature	7	4	7	0
PF10585	UBA_e1_thiolCys	Ubiquitin-activating enzyme active site	3	3	7	0
PF10587	EF-1_beta_acid	Eukaryotic elongation factor 1 beta central acidic region	0	4	6	0
PF10588	NADH-G_4Fe-4S_3	NADH-ubiquinone oxidoreductase-G iron-sulfur binding region	1	3	1	0
PF10589	NADH_4Fe-4S	NADH-ubiquinone oxidoreductase-F iron-sulfur binding region	1	2	1	0
PF10590	PNP_phzG_C	Pyridoxine 5'-phosphate oxidase C-terminal dimerisation region	1	2	1	0
PF10591	SPARC_Ca_bdg	Secreted protein acidic and rich in cysteine Ca binding region	2	7	11	0
PF10595	NA	Uncharacterised protein family UPF0564	1	1	1	1
PF10596	U6-snRNA_bdg	U6-snRNA interacting domain of PrP8	1	1	1	0
PF10597	U5_2-snRNA_bdg	U5-snRNA binding site 2 of PrP8	1	1	1	0
PF10598	RRM_4	RNA recognition motif of the spliceosomal PrP8	1	1	1	1
PF10601	NA	LITAF-like zinc ribbon domain	3	11	22	0
PF10602	RPN7	26S proteasome subunit RPN7	2	3	4	1
PF10607	NA	CTLH/CRA C-terminal to LisH motif domain	4	5	7	3
PF10609	ParA	NUBPL iron-transfer P-loop NTPase	4	6	6	2
PF10613	Lig_chan-Glu_bd	Ligated ion channel L-glutamate- and glycine-binding site	44	22	46	29
PF10629	NA	Protein of unknown function (DUF2475)	3	0	4	1
PF10637	Ofd1_CTDD	Oxoglutarate and iron-dependent oxygenase degradation C-term	1	2	1	0
PF10639	NA	Putative transmembrane family 234	1	1	1	0
PF10644	NA	Misato Segment II tubulin-like domain	1	1	1	0
PF10650	NA	Putative zinc-finger domain	1	2	0	0
PF10660	NA	Iron-containing outer mitochondrial membrane protein N-terminus	1	1	1	1
PF10712	NA	NAD-specific glutamate dehydrogenase	0	0	0	2
PF10717	NA	Occlusion-derived virus envelope protein ODV-E18	0	1	0	0
PF10744	NA	Mediator of RNA polymerase II transcription subunit 1	1	1	1	1
PF10780	MRP_L53	39S ribosomal protein L53/MRP-L53	0	1	1	0
PF10856	NA	Protein of unknown function (DUF2678)	1	2	0	0
PF10873	NA	Protein of unknown function (DUF2668)	0	0	1	0
PF10914	NA	Protein of unknown function (DUF2781)	1	9	3	0
PF10937	NA	Protein of unknown function (DUF2638)	1	1	1	0
PF10961	NA	Protein of unknown function (DUF2763)	0	4	1	1
PF10996	Beta-Casp	Beta-Casp domain	4	3	4	0
PF10998	NA	Protein of unknown function (DUF2838)	2	2	2	0
PF11020	NA	Domain of unknown function (DUF2610)	0	1	0	0
PF11027	NA	Protein of unknown function (DUF2615)	1	0	2	0
PF11028	NA	Protein of unknown function (DUF2723)	0	1	0	0
PF11029	NA	DAZ associated protein 2 (DAZAP2)	1	1	2	0
PF11035	NA	Small nuclear RNA activating complex subunit 2, SNAP190 Myb	1	0	3	0
PF11069	NA	Protein of unknown function (DUF2870)	1	1	3	0
PF11095	Gemin7	Gem-associated protein 7 (Gemin7)	1	0	1	0
PF11105	NA	Arthropod cardioacceleratory peptide 2a	0	0	0	1
PF11107	FANCF	Fanconi anemia group F protein (FANCF)	1	3	2	1
PF11111	CENP-M	Centromere protein M (CENP-M)	0	1	1	0
PF11176	NA	Protein of unknown function (DUF2962)	1	1	1	1
PF11179	NA	Protein of unknown function (DUF2967)	0	0	0	1
PF11214	NA	Mediator complex subunit 2	0	0	1	0
PF11218	NA	Protein of unknown function (DUF3011)	0	1	5	2
PF11221	Med21	Subunit 21 of Mediator complex	1	2	2	0
PF11229	NA	Protein of unknown function (DUF3028)	1	2	2	0
PF11232	Med25	Mediator complex subunit 25 PTOV activation and synapsin 2	1	6	9	0

PF11261	NA	Interferon regulatory factor 2-binding protein zinc finger	1	1	1	0
PF11262	NA	Transcription factor/nuclear export subunit protein 2	1	1	1	0
PF11265	NA	Mediator complex subunit 25 von Willebrand factor type A	1	6	2	0
PF11277	NA	Mediator complex subunit 24 N-terminal	1	5	2	3
PF11303	NA	Protein of unknown function (DUF3105)	1	1	1	0
PF11315	NA	Mediator complex subunit 30	1	1	1	1
PF11326	NA	Protein of unknown function (DUF3128)	1	1	1	0
PF11357	NA	Cell cycle regulatory protein	1	1	1	0
PF11362	NA	Protein of unknown function (DUF3161)	1	2	2	0
PF11365	NA	Protein of unknown function (DUF3166)	0	1	1	1
PF11380	NA	Stealth protein CR2, conserved region 2	1	1	3	0
PF11409	NA	Smad anchor for receptor activation (SARA)	1	1	0	0
PF11411	DNA_ligase_IV	DNA ligase IV	1	2	2	0
PF11413	HIF-1	Hypoxia-inducible factor-1	0	1	2	0
PF11414	Suppressor_APC	Adenomatous polyposis coli tumour suppressor protein	3	0	4	0
PF11416	NA	Syntaxin-5 N-terminal, Sly1p-binding domain	1	1	2	0
PF11461	NA	Rab interacting lysosomal protein	1	1	5	0
PF11464	Rbsn	Rabenosyn Rab binding domain	1	1	1	1
PF11467	LEDGF	Lens epithelium-derived growth factor (LEDGF)	0	1	4	0
PF11470	TUG-UBL1	TUG ubiquitin-like domain	1	1	1	0
PF11502	NA	B-cell lymphoma 9 protein	1	0	3	1
PF11510	NA	Fanconi Anaemia group E protein FANCE	1	2	1	0
PF11515	NA	Mouse development and cellular proliferation protein Cullin-7	1	1	1	0
PF11521	NA	C-terminal general transcription factor TFIIE alpha	1	5	0	0
PF11527	ARL2_Bind_BART	The ARF-like 2 binding protein BART	2	2	3	0
PF11531	NA	Coactivator-associated arginine methyltransferase 1 N terminal	0	1	0	0
PF11538	Snurportin1	Snurportin1	1	1	5	0
PF11540	Dynein_IC2	Cytoplasmic dynein 1 intermediate chain 2	1	1	19	0
PF11543	UN_NPL4	Nuclear pore localisation protein NPL4	1	1	1	0
PF11547	E3_UbLigase_EDD	E3 ubiquitin ligase EDD	1	2	1	0
PF11548	Receptor_IA-2	Protein-tyrosine phosphatase receptor IA-2	1	0	0	0
PF11559	NA	Afadin- and alpha -actinin-Binding	1	1	3	0
PF11566	PI31_Prot_N	PI31 proteasome regulator N-terminal	1	4	2	0
PF11568	NA	Mediator complex subunit 29	1	1	1	1
PF11571	NA	Mediator complex subunit 27	1	1	1	0
PF11573	NA	Mediator complex subunit 23	1	7	2	5
PF11577	NEMO	NF-kappa-B essential modulator NEMO	0	0	2	0
PF11594	NA	Mediator complex subunit 28	1	1	1	0
PF11597	NA	Mediator complex subunit 13 N-terminal	1	2	1	1
PF11598	COMP	Cartilage oligomeric matrix protein	0	1	0	0
PF11600	NA	Chromatin assembly factor 1 complex p150 subunit, N-terminal	0	1	1	0
PF11601	Shal-type	Shal-type voltage-gated potassium channels	1	3	2	1
PF11605	NA	Vacuolar protein sorting protein 36 Vps36	1	3	2	0
PF11608	NA	Limkain b1	1	1	1	0
PF11618	NA	First C2 domain of RPGR-interacting protein 1	1	1	3	0
PF11620	NA	GA-binding protein alpha chain	1	4	1	0
PF11626	Rap1_C	TRF2-interacting telomeric protein/Rap1 - C terminal domain	1	0	0	0
PF11629	Mst1_SARAH	C terminal SARAH domain of Mst1	1	1	3	0
PF11635	NA	Mediator complex subunit 16	1	2	1	0
PF11640	NA	Telomere-length maintenance and DNA damage repair	1	0	1	0
PF11648	RIG-I_C-RD	C-terminal domain of RIG-I	3	8	12	8
PF11652	NA	Protein of unknown function (DUF3259)	1	3	11	1
PF11669	NA	WW domain-binding protein 1	2	5	6	0
PF11698	V-ATPase_H_C	V-ATPase subunit H	1	1	4	0
PF11699	NA	Mif2/CENP-C like	1	1	2	0
PF11701	UNC45-central	Myosin-binding striated muscle assembly central	1	1	2	0
PF11704	Folliculin	Vesicle coat protein involved in Golgi to plasma membrane transport	2	8	2	0
PF11705	RNA_pol_3_Rpc31	DNA-directed RNA polymerase III subunit Rpc31	1	1	1	0
PF11707	Npa1	Ribosome 60S biogenesis N-terminal	1	1	1	0
PF11708	Slu7	Pre-mRNA splicing Prp18-interacting factor	1	2	2	0
PF11710	NA	G protein-coupled glucose receptor regulating Gpa2	0	0	4	0
PF11711	NA	Inner membrane protein import complex subunit Tim54	1	0	0	0
PF11712	NA	Endoplasmic reticulum-based factor for assembly of V-ATPase	1	0	1	0
PF11715	Nup160	Nucleoporin Nup120/160	2	2	4	1
PF11717	Tudor-knot	RNA binding activity-knot of a chromodomain	3	5	12	0
PF11718	NA	Pre-mRNA 3'-end-processing endonuclease polyadenylation factor C-term	1	1	1	0
PF11719	NA	DNA replication and checkpoint protein	0	1	1	0
PF11721	Malectin	Di-glucose binding within endoplasmic reticulum	1	1	1	1
PF11722	NA	CCCH zinc finger in TRM13 protein	1	4	1	0
PF11732	NA	Transcription- and export-related complex subunit	1	1	1	0
PF11735	NA	Cryptococcal mannosyltransferase 1	0	0	0	1
PF11764	N-SET	COMPASS (Complex proteins associated with Set1p) component N	1	1	1	0
PF11768	NA	Protein of unknown function (DUF3312)	1	1	1	0
PF11779	NA	Protein of unknown function (DUF3317)	1	0	0	1
PF11781	zf-RRN7	RNA polymerase I-specific transcription initiation factor Rrn7	0	0	1	0
PF11788	MRP-L46	39S mitochondrial ribosomal protein L46	1	2	1	0
PF11789	NA	Zinc-finger of the MIZ type in Nse subunit	1	1	2	1

PF11790	Glyco_hydro_cc	Glycosyl hydrolase catalytic core	8	0	0	5
PF11793	NA	FANCL C-terminal domain	1	1	2	0
PF11798	IMS_HHH	IMS family HHH motif	0	2	0	0
PF11799	IMS_C	impB/mucB/samB family C-terminal domain	4	6	4	0
PF11801	NA	Tom37 C-terminal domain	1	1	0	0
PF11802	CENP-K	Centromere-associated protein K	0	1	1	0
PF11816	DUF3337	Domain of unknown function (DUF3337)	1	1	3	1
PF11817	NA	Foie gras liver health family 1	1	6	1	0
PF11819	NA	Domain of unknown function (DUF3338)	0	1	6	0
PF11822	NA	Domain of unknown function (DUF3342)	1	1	4	0
PF11830	NA	Domain of unknown function (DUF3350)	0	2	5	1
PF11831	Myb_Cef	pre-mRNA splicing factor component	1	1	0	1
PF11834	NA	KHA, dimerisation domain of potassium ion channel	0	1	1	1
PF11838	ERAP1_C	ERAP1-like C-terminal domain	9	19	15	5
PF11841	DUF3361	Domain of unknown function (DUF3361)	1	4	6	0
PF11861	DUF3381	Domain of unknown function (DUF3381)	1	1	1	0
PF11864	NA	Domain of unknown function (DUF3384)	1	1	1	0
PF11865	DUF3385	Domain of unknown function (DUF3385)	1	3	3	1
PF11875	NA	Domain of unknown function (DUF3395)	1	1	1	0
PF11878	NA	Domain of unknown function (DUF3398)	2	6	28	0
PF11879	NA	Domain of unknown function (DUF3399)	1	1	2	1
PF11881	NA	C-terminal domain of SPAR protein	0	0	4	0
PF11882	NA	Domain of unknown function (DUF3402)	1	1	3	2
PF11894	Nup192	Nuclear pore complex scaffold, nucleoporins 186/192/205	1	3	1	2
PF11901	DUF3421	Protein of unknown function (DUF3421)	0	0	9	0
PF11904	NA	GPCR-chaperone	2	2	3	1
PF11911	NA	Protein of unknown function (DUF3429)	1	1	1	0
PF11914	NA	Domain of unknown function (DUF3432)	0	1	0	0
PF11916	NA	Vacuolar protein 14 C-terminal Fig4p binding	1	1	1	1
PF11919	DUF3437	Domain of unknown function (DUF3437)	1	2	2	0
PF11923	NA	Domain of unknown function (DUF3441)	1	1	2	0
PF11931	DUF3449	Domain of unknown function (DUF3449)	1	1	1	1
PF11933	Na_trans_cytopl	Cytoplasmic domain of voltage-gated Na+ ion channel	1	1	0	1
PF11934	DUF3452	Domain of unknown function (DUF3452)	1	2	2	0
PF11935	DUF3453	Domain of unknown function (DUF3453)	1	1	1	0
PF11936	NA	Domain of unknown function (DUF3454)	0	1	3	1
PF11938	NA	TLR4 regulator and MIR-interacting MSAP	3	6	2	1
PF11940	DUF3458	Domain of unknown function (DUF3458)	0	0	1	0
PF11942	Spt5_N	Spt5 transcription elongation factor, acidic N-terminal	1	1	0	0
PF11945	NA	WAHD domain of WASH complex	1	1	1	2
PF11952	XTBD	XRN-Two Binding Domain, XTBD	1	0	2	2
PF11954	NA	Domain of unknown function (DUF3471)	0	0	1	0
PF11957	NA	THO complex subunit 1 transcription elongation factor	1	1	1	0
PF11958	NA	Domain of unknown function (DUF3472)	0	0	1	0
PF11968	NA	Putative methyltransferase (DUF3321)	0	1	0	0
PF11969	DcpS_C	Scavenger mRNA decapping enzyme C-term binding	3	3	3	2
PF11971	CAMSAP_CH	CAMSAP CH domain	1	5	3	2
PF11976	Rad60-SLD	Ubiquitin-2 like Rad60 SUMO-like	2	1	2	0
PF11977	RNase_Zc3h12a	Zc3h12a-like Ribonuclease NYN domain	2	3	3	1
PF11978	MVP_shoulder	Shoulder domain	2	5	3	2
PF11979	NA	Domain of unknown function (DUF3480)	1	1	2	3
PF11987	IF-2	Translation-initiation factor 2	2	6	2	1
PF12002	MgsA_C	MgsA AAA+ ATPase C terminal	1	2	0	1
PF12004	NA	Domain of unknown function (DUF3498)	1	6	19	1
PF12009	Telomerase_RBD	Telomerase ribonucleoprotein complex - RNA binding domain	0	1	1	0
PF12012	NA	Domain of unknown function (DUF3504)	23	4	46	4
PF12017	Tnp_P_element	Transposase protein	0	1	2	1
PF12018	NA	Domain of unknown function (DUF3508)	2	1	1	1
PF12022	NA	Domain of unknown function (DUF3510)	1	1	1	0
PF12024	NA	Domain of unknown function (DUF3512)	1	2	1	0
PF12026	NA	Domain of unknown function (DUF3513)	1	1	6	1
PF12031	BAF250_C	Domain of unknown function (DUF3518)	1	6	5	1
PF12036	NA	Protein of unknown function (DUF3522)	1	6	2	7
PF12037	NA	Domain of unknown function (DUF3523)	1	1	1	1
PF12038	NA	Domain of unknown function (DUF3524)	0	1	2	1
PF12044	NA	Putative peptidase family	1	1	2	1
PF12047	DNMT1-RFD	Cytosine specific DNA methyltransferase replication foci domain	1	2	1	1
PF12052	VGCC_beta4Aa_N	Voltage gated calcium channel subunit beta domain 4Aa N terminal	1	1	0	1
PF12053	Par3_HAL_N_term	Domain of unknown function (DUF3534)	2	6	12	0
PF12054	NA	Domain of unknown function (DUF3535)	2	1	1	1
PF12057	NA	Domain of unknown function (DUF3538)	1	1	2	1
PF12062	NA	heparan sulfate-N-deacetylase	1	9	3	1
PF12063	NA	Domain of unknown function (DUF3543)	0	1	3	0
PF12066	DUF3546	Domain of unknown function (DUF3546)	1	5	5	0
PF12068	NA	Domain of unknown function (DUF3548)	1	2	2	0
PF12070	NA	Protein of unknown function (DUF3550/UPF0682)	1	1	2	1
PF12074	NA	Domain of unknown function (DUF3554)	1	1	1	0

PF12075	NA	KN motif	0	10	15	1
PF12090	NA	Spt20 family	1	2	1	1
PF12108	SF3a60_bindingd	Splicing factor SF3a60 binding domain	0	1	1	0
PF12110	Nup96	Nuclear protein 96	0	1	1	0
PF12114	Period_C	Period protein 2/3C-terminal region	0	1	4	1
PF12125	Beta-TrCP_D	D domain of beta-TrCP	1	1	2	0
PF12129	NA	Male germ-cell putative homeodomain transcription factor	0	10	6	0
PF12130	DUF3585	Protein of unknown function (DUF3585)	3	5	17	0
PF12134	PRP8_domainIV	PRP8 domain IV core	1	1	1	1
PF12140	NA	Protein of unknown function (DUF3588)	3	5	6	0
PF12145	Med12-LCEWAV	Eukaryotic Mediator 12 subunit domain	1	3	3	2
PF12146	Hydrolase_4	Serine aminopeptidase, S33	3	2	10	1
PF12148	TTD	Tandem tudor domain within UHRF1	1	1	1	1
PF12157	DUF3591	Protein of unknown function (DUF3591)	1	1	1	1
PF12166	Piezo_RRas_bdg	Piezo non-specific cation channel, R-Ras-binding domain	1	1	2	3
PF12171	zf-C2H2_jaz	Zinc-finger double-stranded RNA-binding	10	10	18	0
PF12202	OSR1_C	Oxidative-stress-responsive kinase 1 C terminal	1	8	6	1
PF12203	HDAC4_Gln	Glutamine rich N terminal domain of histone deacetylase 4	0	0	11	0
PF12205	GIT1_C	G protein-coupled receptor kinase-interacting protein 1 C term	1	1	4	1
PF12210	NA	Hepatocyte growth factor-regulated tyrosine kinase substrate	1	7	2	0
PF12213	NA	DNA polymerases epsilon N terminal	1	1	1	0
PF12214	NA	Cell cycle regulated microtubule associated protein	1	1	2	0
PF12215	Glyco_hydr_116N	beta-Glucocerebrosidase 2 N terminal	1	1	3	1
PF12220	U1snRNP70_N	U1 small nuclear ribonucleoprotein of 70kDa MW N terminal	1	1	4	0
PF12230	PRP21_like_P	Pre-mRNA splicing factor PRP21 like protein	1	1	1	0
PF12231	Rif1_N	Rap1-interacting factor 1 N terminal	1	1	2	0
PF12232	NA	Myogenic determination factor 5	0	0	1	0
PF12234	NA	RAVE protein 1 C terminal	1	3	3	3
PF12235	NA	Fragile X-related 1 protein core C terminal	1	2	1	0
PF12237	PCIF1_WW	Phosphorylated CTD interacting factor 1 WW domain	1	1	2	0
PF12240	NA	Angiomotin C terminal	1	1	5	0
PF12248	NA	Farnesoic acid 0-methyl transferase	11	1	19	6
PF12251	NA	snRNA-activating protein of 50kDa MW C terminal	1	1	1	0
PF12253	NA	Chromatin assembly factor 1 subunit A	0	1	1	0
PF12254	NA	DNA polymerase alpha subunit p180 N terminal	1	1	2	0
PF12257	IML1	Protein of unknown function (DUF3608)	1	1	5	2
PF12259	NA	Protein of unknown function (DUF3609)	3	0	0	0
PF12260	NA	Protein-kinase domain of FAM69	7	10	10	6
PF12265	CAF1C_H4-bd	Histone-binding protein RBBP4 or subunit C of CAF1 complex	2	0	2	1
PF12269	NA	CpG binding protein zinc finger C terminal domain	2	1	2	1
PF12280	NA	Brain specific membrane anchored protein	0	1	1	0
PF12287	NA	Cytoplasmic activation/proliferation-associated protein-1 C term	1	0	0	0
PF12295	NA	Symplekin tight junction protein C terminal	1	1	1	1
PF12297	NA	Ellis van Creveld protein 2 like protein	1	1	5	2
PF12309	NA	KIF-1 binding protein C terminal	1	1	1	1
PF12316	NA	Segment polarity protein dishevelled (Dsh) C terminal	1	3	0	1
PF12317	NA	Intraflagellar transport complex B protein 46 C terminal	1	5	6	0
PF12325	NA	TATA element modulatory factor 1 TATA binding	0	0	2	0
PF12327	FtsZ_C	FtsZ family, C-terminal domain	0	2	0	0
PF12328	Rpp20	Rpp20 subunit of nuclear RNase MRP and P	0	0	2	1
PF12329	NA	TATA element modulatory factor 1 DNA binding	0	9	2	0
PF12330	Haspin_kinase	Domain of unknown function (DUF3635)	1	1	1	0
PF12333	NA	Rix1 complex component involved in 60S ribosome maturation	1	1	2	1
PF12335	NA	Myotubularin protein	2	1	7	1
PF12336	NA	SOX transcription factor	1	4	2	2
PF12341	Mcl1_mid	Minichromosome loss protein, Mcl1, middle region	1	1	1	0
PF12344	UvrB	Ultra-violet resistance protein B	0	2	0	0
PF12345	NA	Protein of unknown function (DUF3641)	0	0	1	0
PF12347	NA	Holliday junction regulator protein family C-terminal repeat	1	0	0	0
PF12348	CLASP_N	CLASP N terminal	3	19	30	1
PF12349	Sterol-sensing	Sterol-sensing domain of SREBP cleavage-activation	3	12	8	6
PF12352	NA	Snare region anchored in the vesicle membrane C-terminus	4	5	4	1
PF12353	eIF3g	Eukaryotic translation initiation factor 3 subunit G	1	1	1	0
PF12356	NA	Protein of unknown function (DUF3643)	1	2	1	2
PF12366	NA	Cancer susceptibility candidate 1	0	1	4	0
PF12371	NA	Transmembrane protein 131-like	1	1	1	0
PF12372	DUF3652	Huntingtin protein region	1	1	1	0
PF12394	NA	Protein of unknown function (DUF3657)	1	1	6	0
PF12397	U3snoRNP10	U3 small nucleolar RNA-associated protein 10	0	1	1	0
PF12400	NA	Vaculolar membrane protein	1	5	1	0
PF12409	NA	P5-type ATPase cation transporter	2	5	8	0
PF12416	DUF3668	Cep120 protein	1	1	1	0
PF12422	NA	Condensin II non structural maintenance of chromosomes subunit	1	1	2	0
PF12423	KIF1B	Kinesin protein 1B	4	17	15	1
PF12424	NA	Plasma membrane calcium transporter ATPase C terminal	0	1	1	0
PF12430	NA	Abscisic acid G-protein coupled receptor	1	1	1	1
PF12432	NA	Protein of unknown function (DUF3677)	1	1	1	0

PF12436	USP7_ICP0_bdg	ICP0-binding domain of Ubiquitin-specific protease 7	1	1	4	2
PF12444	NA	Sox developmental protein N terminal	1	1	2	1
PF12448	NA	Kinesin associated protein	0	2	8	1
PF12451	NA	Vacuolar protein sorting protein 11 C terminal	1	1	1	0
PF12455	NA	Dynein associated protein	1	1	2	0
PF12456	NA	Inositol phosphatase	2	2	2	0
PF12457	NA	Tuftelin interacting protein N terminal	1	1	1	0
PF12460	MMS19_C	RNAPII transcription regulator C-terminal	1	1	1	1
PF12463	NA	Protein of unknown function (DUF3689)	1	2	3	2
PF12465	Pr_beta_C	Proteasome beta subunits C terminal	1	1	1	0
PF12470	SUFU_C	Suppressor of Fused Gli/Ci N terminal binding domain	1	3	2	1
PF12473	NA	Kinesin protein	3	15	16	2
PF12474	NA	Polo kinase kinase	1	1	1	1
PF12478	NA	Ubiquitin-associated protein 2	1	1	3	0
PF12483	NA	E3 Ubiquitin ligase	1	1	2	1
PF12489	NA	Nuclear coactivator	1	2	2	1
PF12490	NA	Breast carcinoma amplified sequence 3	1	2	1	0
PF12494	NA	Protein of unknown function (DUF3695)	1	2	2	1
PF12496	NA	Bcl2-/adenovirus E1B nineteen kDa-interacting protein 2	1	1	1	0
PF12509	NA	Protein of unknown function (DUF3715)	2	1	9	0
PF12510	NA	Smoothelin cytoskeleton protein	1	1	10	0
PF12513	NA	Mitochondrial degradasome RNA helicase subunit C terminal	1	1	1	0
PF12516	NA	Protein of unknown function (DUF3719)	1	4	8	0
PF12529	Xylo_C	Xylosyltransferase C terminal	1	1	1	2
PF12530	NA	Protein of unknown function (DUF3730)	1	1	1	0
PF12533	NA	Neuronal helix-loop-helix transcription factor	1	1	2	0
PF12537	NA	The Golgi pH Regulator (GPHR) Family N-terminal	1	1	0	0
PF12540	NA	Protein of unknown function (DUF3736)	1	4	2	1
PF12542	NA	Pre-mRNA splicing factor	0	1	1	0
PF12548	NA	Sulfatase protein	0	0	1	0
PF12554	MOZART1	Mitotic-spindle organizing gamma-tubulin ring associated	1	1	0	1
PF12569	NARP1	NMDA receptor-regulated protein 1	2	1	1	1
PF12572	NA	Protein of unknown function (DUF3752)	0	1	1	0
PF12576	NA	Protein of unknown function (DUF3754)	1	2	1	1
PF12578	NA	Myotubularin-associated protein	1	1	1	1
PF12580	TPPII	Tripeptidyl peptidase II	1	2	1	1
PF12583	TPPII_N	Tripeptidyl peptidase II N terminal	1	0	1	0
PF12584	NA	Trafficking protein particle complex subunit 10, TRAPPC10	1	0	0	0
PF12589	NA	Methyltransferase involved in Williams-Beuren syndrome	1	0	2	0
PF12597	NA	Protein of unknown function (DUF3767)	0	1	0	0
PF12605	CK1gamma_C	Casein kinase 1 gamma C terminal	1	8	2	0
PF12612	NA	Tubulin folding cofactor D C terminal	1	1	2	0
PF12619	MCM2_N	Mini-chromosome maintenance protein 2	0	2	1	0
PF12624	Chorein_N	N-terminal region of Chorein or VPS13	5	13	18	2
PF12627	PolyA_pol_RNAbd	Probable RNA and SrmB- binding site of polymerase A	1	2	1	0
PF12631	NA	MnmE helical domain	1	3	1	1
PF12632	NA	Mysoin-binding motif of peroxisomes	1	0	1	0
PF12640	NA	UPF0489 domain	8	2	6	0
PF12656	NA	DExH-box splicing factor binding site	1	1	2	1
PF12657	NA	Transcription factor IIIC subunit delta N-term	0	4	0	0
PF12661	hEGF	Human growth factor-like EGF	33	37	78	12
PF12662	cEGF	Complement Clr-like EGF-like	12	38	39	5
PF12678	zf-rbx1	RING-H2 zinc finger	2	1	4	0
PF12681	NA	Glyoxalase-like domain	0	0	2	0
PF12689	Acid_PPase	Acid Phosphatase	1	2	2	0
PF12695	NA	Alpha/beta hydrolase family	13	21	16	0
PF12697	Abhydrolase_6	Alpha/beta hydrolase family	1	6	2	1
PF12698	NA	ABC-2 family transporter protein	5	38	24	11
PF12701	LSM14	Scd6-like Sm domain	2	2	7	0
PF12705	PDDEXK_1	PD-(D/E)XK nuclease superfamily	0	2	2	0
PF12706	Lactamase_B_2	Beta-lactamase superfamily domain	4	13	11	1
PF12710	HAD	haloacid dehalogenase-like hydrolase	9	31	43	0
PF12711	NA	Kinesin motor	1	1	1	0
PF12714	NA	TILa domain	0	0	1	0
PF12717	NA	non-SMC mitotic condensation complex subunit 1	3	3	5	1
PF12719	Cnd3	Nuclear condensing complex subunits, C-term domain	1	1	1	1
PF12721	NA	RIP homotypic interaction motif	0	0	1	0
PF12722	NA	High-temperature-induced dauer-formation protein	0	0	1	2
PF12733	NA	Cadherin-like beta sandwich domain	1	2	3	0
PF12736	NA	Cell-cycle sustaining, positive selection,	8	13	20	3
PF12738	PTCB-BRCT	twin BRCT domain	6	8	7	0
PF12739	NA	ER-Golgi trafficking TRAPP I complex 85 kDa subunit	1	2	2	0
PF12740	Chlorophyllase2	Chlorophyllase enzyme	1	4	1	1
PF12742	NA	Gryzun, putative Golgi trafficking	0	4	1	1
PF12745	HGTP_anticodon2	Anticodon binding domain of tRNAs	1	2	1	0
PF12752	NA	SUZ domain	1	1	1	0
PF12755	NA	Vacuolar 14 Fab1-binding region	1	1	1	0

PF12756	zf-C2H2_2	C2H2 type zinc-finger (2 copies)	4	4	5	1
PF12762	NA	ISXO2-like transposase domain	1	6	5	0
PF12763	EF-hand_4	Cytoskeletal-regulatory complex EF hand	5	12	11	0
PF12765	Cohesin_HEAT	HEAT repeat associated with sister chromatid cohesion	4	2	5	0
PF12767	NA	Transcriptional regulator of RNA polII, SAGA, subunit	1	1	1	0
PF12768	NA	Cortical protein marker for cell polarity	0	1	0	0
PF12769	PNTB_4TM	4TM region of pyridine nucleotide transhydrogenase, mitoch	1	1	1	1
PF12770	NA	CHAT domain	2	2	7	3
PF12773	NA	Double zinc ribbon	1	0	2	2
PF12774	AAA_6	Hydrolytic ATP binding site of dynein motor region D1	19	41	63	36
PF12775	AAA_7	P-loop containing dynein motor region D3	17	35	62	28
PF12777	MT	Microtubule-binding stalk of dynein motor	17	40	63	28
PF12780	AAA_8	P-loop containing dynein motor region D4	18	35	66	32
PF12781	AAA_9	ATP-binding dynein motor region D5	21	41	68	30
PF12783	NA	Guanine nucleotide exchange factor in Golgi transport N-terminal	3	5	12	2
PF12796	Ank_2	Ankyrin repeats (3 copies)	130	505	711	164
PF12799	LRR_4	Leucine Rich repeats (2 copies)	6	12	11	9
PF12807	NA	Translation initiation factor eIF3 subunit 135	1	1	1	0
PF12810	NA	Glycine rich protein	1	2	2	0
PF12813	NA	XPG domain containing	2	1	3	1
PF12815	NA	Spt5 C-terminal nonapeptide repeat binding Spt4	2	1	0	2
PF12816	NA	Golgi CORVET complex core vacuolar protein 8	1	1	1	1
PF12826	HHH_2	Helix-hairpin-helix motif	0	0	1	0
PF12830	Nipped-B_C	Sister chromatid cohesion C-terminus	1	1	4	1
PF12832	NA	MFS_1 like family	2	6	5	13
PF12836	HHH_3	Helix-hairpin-helix motif	2	10	6	1
PF12838	Fer4_7	4Fe-4S dicluster domain	1	1	1	0
PF12842	DUF3819	Domain of unknown function (DUF3819)	1	1	3	1
PF12847	NA	Methyltransferase domain	11	5	11	0
PF12848	ABC_tran_Xtn	ABC transporter	3	4	3	0
PF12849	PBP_like_2	PBP superfamily domain	0	0	2	1
PF12850	Metallophos_2	Calcineurin-like phosphoesterase superfamily domain	1	1	1	1
PF12851	Tet_JBP	Oxygenase domain of the 2OGFeDO superfamily	2	4	9	1
PF12859	ANAPC1	Anaphase-promoting complex subunit 1	1	0	1	0
PF12861	zf-ANAPC11	Anaphase-promoting complex subunit 11 RING-H2 finger	1	1	0	0
PF12862	ANAPC5	Anaphase-promoting complex subunit 5	1	1	1	1
PF12867	DinB_2	DinB superfamily	1	3	1	0
PF12872	OST-HTH	OST-HTH/LOTUS domain	3	7	5	1
PF12874	zf-met	Zinc-finger of C2H2 type	150	249	103	25
PF12877	NA	Domain of unknown function (DUF3827)	1	1	4	1
PF12884	TORC_N	Transducer of regulated CREB activity, N terminus	1	5	4	0
PF12885	NA	Transducer of regulated CREB activity middle domain	1	5	4	1
PF12886	NA	Transducer of regulated CREB activity, C terminus	1	5	4	1
PF12894	ANAPC4_WD40	Anaphase-promoting complex subunit 4 WD40 domain	1	2	1	3
PF12895	ANAPC3	Anaphase-promoting complex, cyclosome, subunit 3	3	1	4	0
PF12896	ANAPC4	Anaphase-promoting complex, cyclosome, subunit 4	2	1	1	1
PF12901	NA	SUZ-C motif	3	1	3	0
PF12906	RINGv	RING-variant domain	6	8	16	1
PF12907	NA	Zinc-binding	0	0	0	1
PF12922	NA	non-SMC mitotic condensation complex subunit 1, N-term	1	1	3	0
PF12923	RRP7	Ribosomal RNA-processing protein 7 (RRP7)	1	1	1	0
PF12924	APP_Cu_bd	Copper-binding of amyloid precursor, CuBD	1	1	0	0
PF12925	APP_E2	E2 domain of amyloid precursor protein	1	1	0	1
PF12926	NA	Mitotic-spindle organizing gamma-tubulin ring associated	1	1	1	0
PF12928	NA	tRNA-splicing endonuclease subunit sen54 N-term	1	3	0	0
PF12929	NA	Stretch-activated Ca2+-permeable channel component	0	5	2	0
PF12931	Sec16_C	Sec23-binding domain of Sec16	1	8	2	0
PF12932	NA	Vesicle coat trafficking protein Sec16 mid-region	1	8	2	0
PF12936	NA	KRI1-like family C-terminal	1	1	2	0
PF12937	F-box-like	F-box-like	48	86	103	16
PF12938	NA	M domain of GW182	0	4	4	1
PF12947	EGF_3	EGF domain	7	8	44	3
PF12971	NAGLU_N	Alpha-N-acetylglucosaminidase (NAGLU) N-terminal domain	2	4	4	0
PF12972	NAGLU_C	Alpha-N-acetylglucosaminidase (NAGLU) C-terminal domain	3	4	4	0
PF12974	Phosphonate-bd	ABC transporter, phosphonate, periplasmic substrate-binding protein	1	2	2	0
PF12998	ING	Inhibitor of growth proteins N-terminal histone-binding	3	3	3	0
PF12999	PRKCSH-like	Glucosidase II beta subunit-like	2	2	4	0
PF13000	NA	Acetyl-coenzyme A transporter 1	1	1	1	1
PF13001	NA	Proteasome stabiliser	1	9	1	0
PF13012	MitMem_reg	Maintenance of mitochondrial structure and function	4	6	5	2
PF13014	NA	KH domain	1	2	0	0
PF13015	NA	Glucosidase II beta subunit-like protein	3	3	5	0
PF13017	NA	piRNA pathway germ-plasm component	1	1	2	0
PF13019	NA	Telomere stability and silencing	1	2	1	0
PF13020	NA	Domain of unknown function (DUF3883)	0	2	2	1
PF13023	HD_3	HD domain	1	2	1	0
PF13041	PPR_2	PPR repeat family	1	0	0	0

PF13085	Fer2_3	2Fe-2S iron-sulfur cluster binding domain	1	2	2	1
PF13086	AAA_11	AAA domain	13	29	39	25
PF13087	AAA_12	AAA domain	12	26	41	18
PF13088	BNR_2	BNR repeat-like domain	0	0	3	0
PF13091	PLDc_2	PLD-like domain	3	1	7	1
PF13092	NA	Kinetochore complex Sim4 subunit Fta1	0	1	1	0
PF13096	NA	CENP-A-nucleosome distal (CAD) centromere subunit, CENP-P	0	1	1	0
PF13097	NA	CENP-A nucleosome associated complex (NAC) subunit	0	0	1	0
PF13148	NA	Protein of unknown function (DUF3987)	0	1	5	1
PF13151	NA	Protein of unknown function (DUF3990)	0	11	0	0
PF13167	GTP-bdg_N	GTP-binding GTPase N-terminal	1	1	3	1
PF13174	TPR_6	Tetratricopeptide repeat	3	5	4	2
PF13176	TPR_7	Tetratricopeptide repeat	4	10	7	2
PF13177	DNA_pol3_delta2	DNA polymerase III, delta subunit	1	1	2	0
PF13180	PDZ_2	PDZ domain	1	2	2	0
PF13181	TPR_8	Tetratricopeptide repeat	24	41	50	10
PF13184	KH_5	NusA-like KH domain	0	1	0	0
PF13185	GAF_2	GAF domain	1	5	12	0
PF13191	NA	AAA ATPase domain	6	6	15	0
PF13193	AMP-binding_C	AMP-binding enzyme C-terminal domain	23	34	31	18
PF13202	EF-hand_5	EF hand	40	52	59	5
PF13207	AAA_17	AAA domain	1	2	17	1
PF13229	Beta_helix	Right handed beta helix region	5	3	6	1
PF13231	PMT_2	Dolichyl-phosphate-mannose-protein mannosyltransferase	1	0	0	0
PF13232	NA	Complex1_LYR-like	1	1	1	0
PF13233	NA	Complex1_LYR-like	1	1	1	0
PF13234	rRNA_proc-arch	rRNA-processing arch domain	2	4	3	1
PF13236	NA	Clustered mitochondria	2	1	1	0
PF13238	AAA_18	AAA domain	2	7	2	0
PF13240	NA	zinc-ribbon domain	0	0	1	0
PF13242	Hydrolase_like	HAD-hyrolase-like	4	10	8	2
PF13243	SQHop_cyclase_C	Squalene-hopene cyclase C-terminal domain	1	1	0	0
PF13245	NA	Part of AAA domain	0	1	3	0
PF13246	Cation_ATPase	Cation transport ATPase (P-type)	3	7	8	2
PF13249	SQHop_cyclase_N	Squalene-hopene cyclase N-terminal domain	1	1	0	0
PF13251	NA	Domain of unknown function (DUF4042)	1	1	1	0
PF13270	NA	Domain of unknown function (DUF4061)	1	2	2	1
PF13271	NA	Domain of unknown function (DUF4062)	13	7	18	4
PF13279	4HBT_2	Thioesterase-like superfamily	1	0	2	1
PF13281	NA	Domain of unknown function (DUF4071)	1	10	10	1
PF13287	NA	Fn3 associated	1	0	1	0
PF13290	NA	Chitobiase/beta-hexosaminidase C-terminal domain	1	1	0	0
PF13292	NA	1-deoxy-D-xylulose-5-phosphate synthase	0	0	1	0
PF13297	Telomere_Sde2_2	Telomere stability C-terminal	2	2	2	1
PF13299	NA	Cleavage and polyadenylation factor 2 C-terminal	1	1	1	0
PF13300	NA	Domain of unknown function (DUF4078)	1	2	1	0
PF13302	Acetyltransf_3	Acetyltransferase (GNAT) domain	1	1	1	0
PF13304	AAA_21	AAA domain, putative AbiEii toxin, Type IV TA system	0	0	1	0
PF13306	LRR_5	Leucine rich repeats (6 copies)	3	35	41	25
PF13307	Helicase_C_2	Helicase C-terminal domain	4	6	6	0
PF13324	NA	Grap2 and cyclin-D-interacting	2	5	5	1
PF13325	MCRS_N	N-terminal region of micro-spherule protein	1	2	2	0
PF13328	HD_4	HD domain	1	4	1	0
PF13330	NA	Mucin-2 protein WxxW repeating region	12	4	3	21
PF13336	AcetylCoA_hyd_C	Acetyl-CoA hydrolase/transferase C-terminal domain	1	0	3	0
PF13339	NA	Apoptosis antagonizing transcription factor	1	1	1	0
PF13344	Hydrolase_6	Haloacid dehalogenase-like hydrolase	5	13	11	2
PF13346	NA	ABC-2 family transporter protein	0	0	1	0
PF13347	MFS_2	MFS/sugar transport protein	5	5	15	0
PF13348	NA	Tyrosine phosphatase family C-terminal region	1	0	0	0
PF13349	NA	Putative adhesin	1	1	1	0
PF13350	NA	Tyrosine phosphatase family	1	1	0	0
PF13353	Fer4_12	4Fe-4S single cluster domain	3	1	1	0
PF13358	NA	DDE superfamily endonuclease	2	0	11	2
PF13359	NA	DDE superfamily endonuclease	24	14	143	11
PF13360	PQQ_2	PQQ-like domain	3	2	3	0
PF13361	UvrD_C	UvrD-like helicase C-terminal domain	1	0	0	0
PF13365	Trypsin_2	Trypsin-like peptidase domain	4	24	17	0
PF13369	NA	Transglutaminase-like superfamily	1	0	1	0
PF13371	NA	Tetratricopeptide repeat	4	4	10	0
PF13374	TPR_10	Tetratricopeptide repeat	8	1	21	0
PF13378	MR_MLE_C	Enolase C-terminal domain-like	2	4	7	1
PF13383	NA	Methyltransferase domain	9	30	20	0
PF13385	Laminin_G_3	Concanavalin A-like lectin/glucanases superfamily	14	24	33	6
PF13392	HNH_3	HNH endonuclease	1	1	1	0
PF13393	tRNA-synt_His	Histidyl-tRNA synthetase	2	5	5	0
PF13401	AAA_22	AAA domain	0	2	0	0

PF13402	Peptidase_M60	Peptidase M60, enhancin and enhancin-like	3	0	0	0
PF13405	EF-hand_6	EF-hand domain	8	24	39	7
PF13409	GST_N_2	Glutathione S-transferase, N-terminal domain	1	0	2	1
PF13410	GST_C_2	Glutathione S-transferase, C-terminal domain	4	5	5	1
PF13414	TPR_11	TPR repeat	50	114	136	5
PF13415	NA	Galactose oxidase, central domain	11	13	19	2
PF13417	GST_N_3	Glutathione S-transferase, N-terminal domain	13	13	17	3
PF13418	Kelch_4	Galactose oxidase, central domain	16	16	12	5
PF13419	HAD_2	Haloacid dehalogenase-like hydrolase	5	14	7	2
PF13420	Acetyltransf_4	Acetyltransferase (GNAT) domain	0	1	0	0
PF13423	UCH_1	Ubiquitin carboxyl-terminal hydrolase	1	3	3	0
PF13424	TPR_12	Tetratricopeptide repeat	22	28	64	4
PF13426	PAS_9	PAS domain	4	5	17	1
PF13428	NA	Tetratricopeptide repeat	0	0	1	0
PF13431	NA	Tetratricopeptide repeat	0	3	0	0
PF13432	TPR_16	Tetratricopeptide repeat	1	0	2	2
PF13445	zf-RING_UBOX	RING-type zinc-finger	15	5	25	56
PF13450	NAD_binding_8	NAD(P)-binding Rossmann-like domain	5	12	14	3
PF13452	MaoC_dehydrat_N	N-terminal half of MaoC dehydratase	0	1	1	0
PF13456	RVT_3	Reverse transcriptase-like	0	0	1	0
PF13460	NAD_binding_10	NAD(P)H-binding	3	13	3	1
PF13462	Thioredoxin_4	Thioredoxin	3	2	1	1
PF13465	zf-H2C2_2	Zinc-finger double domain	28	1	6	5
PF13469	Sulfotransfer_3	Sulfotransferase family	4	3	7	3
PF13472	Lipase_GDSL_2	GDSL-like Lipase/Acylhydrolase family	1	1	1	2
PF13476	AAA_23	AAA domain	2	2	3	0
PF13481	AAA_25	AAA domain	1	1	3	1
PF13489	Methyltransf_23	Methyltransferase domain	4	8	8	1
PF13495	Phage_int_SAM_4	Phage integrase, N-terminal SAM-like domain	1	0	0	0
PF13499	EF-hand_7	EF-hand domain pair	86	123	318	23
PF13506	NA	Glycosyl transferase family 21	3	2	1	0
PF13507	GATase_5	CobB/CobQ-like glutamine amidotransferase domain	1	1	5	0
PF13508	Acetyltransf_7	Acetyltransferase (GNAT) domain	4	3	5	1
PF13510	Fer2_4	2Fe-2S iron-sulfur cluster binding domain	1	3	1	0
PF13513	HEAT_EZ	HEAT-like repeat	5	4	7	2
PF13516	LRR_6	Leucine Rich repeat	5	16	14	37
PF13517	VCBS	Repeat domain in Vibrio, Colwellia, Bradyrhizobium and Shewanella	1	0	2	1
PF13518	NA	Helix-turn-helix domain	0	0	2	0
PF13519	VWA_2	von Willebrand factor type A domain	14	14	61	5
PF13520	AA_permease_2	Amino acid permease	22	39	41	14
PF13521	AAA_28	AAA domain	1	0	2	0
PF13522	GATase_6	Glutamine amidotransferase domain	2	7	9	0
PF13532	2OG-FeII_Oxy_2	2OG-Fe(II) oxygenase superfamily	7	15	7	3
PF13534	Fer4_17	4Fe-4S dicluster domain	1	2	2	0
PF13535	ATP-grasp_4	ATP-grasp domain	3	0	6	3
PF13537	GATase_7	Glutamine amidotransferase domain	2	2	2	0
PF13538	UvrD_C_2	UvrD-like helicase C-terminal domain	1	1	2	0
PF13540	RCC1_2	Regulator of chromosome condensation (RCC1) repeat	3	4	4	3
PF13543	NA	SAM like domain present in kinase suppressor RAS 1	1	1	4	0
PF13561	adh_short_C2	Enoyl-(Acyl carrier protein) reductase	0	1	2	6
PF13563	NA	2'-5' RNA ligase superfamily	1	0	0	0
PF13564	NA	DoxX-like family	2	0	3	0
PF13570	PQQ_3	PQQ-like domain	1	1	0	0
PF13574	Reprolysin_2	Metallo-peptidase family M12B Reprolysin-like	14	37	12	5
PF13582	NA	Metallo-peptidase family M12B Reprolysin-like	1	7	0	1
PF13583	Reprolysin_4	Metallo-peptidase family M12B Reprolysin-like	2	7	1	0
PF13589	HATPase_c_3	Histidine kinase-, DNA gyrase B-, and HSP90-like ATPase	7	10	8	0
PF13593	NA	SBF-like CPA transporter family (DUF4137)	1	9	1	0
PF13598	NA	Domain of unknown function (DUF4139)	1	1	0	0
PF13599	Pentapeptide_4	Pentapeptide repeats (9 copies)	2	4	0	0
PF13600	NA	N-terminal domain of unknown function (DUF4140)	1	1	0	2
PF13602	ADH_zinc_N_2	Zinc-binding dehydrogenase	2	6	4	0
PF13603	tRNA-synt_1_2	Leucyl-tRNA synthetase, Domain 2	0	2	1	0
PF13604	AAA_30	AAA domain	1	0	2	3
PF13606	Ank_3	Ankyrin repeat	2	34	55	20
PF13613	NA	Helix-turn-helix of DDE superfamily endonuclease	9	11	49	2
PF13616	Rotamase_3	PPIC-type PPIASE domain	2	1	1	0
PF13620	CarboxypepD_reg	Carboxypeptidase regulatory-like domain	3	4	3	1
PF13621	Cupin_8	Cupin-like domain	18	13	32	4
PF13622	4HBT_3	Thioesterase-like superfamily	2	7	1	0
PF13625	NA	Helicase conserved C-terminal domain	1	1	1	0
PF13629	NA	Pilus formation protein N terminal region	0	1	0	0
PF13631	NA	Cytochrome b(N-terminal)/b6/petB	0	1	1	0
PF13632	NA	Glycosyl transferase family group 2	3	5	1	2
PF13634	NA	Nucleoporin FG repeat region	2	0	1	0
PF13637	Ank_4	Ankyrin repeats (many copies)	43	157	203	63
PF13638	PIN_4	PIN domain	4	8	5	0

PF13639	zf-RING_2	Ring finger domain	43	57	89	14
PF13640	2OG-FeII_Oxy_3	2OG-Fe(II) oxygenase superfamily	11	9	14	2
PF13646	HEAT_2	HEAT repeats	15	22	30	4
PF13649	Methyltransf_25	Methyltransferase domain	0	1	0	0
PF13650	NA	Aspartyl protease	1	3	9	3
PF13656	RNA_pol_L_2	RNA polymerase Rpb3/Rpb11 dimerisation domain	2	3	2	0
PF13659	NA	Methyltransferase domain	1	2	1	0
PF13660	NA	Domain of unknown function (DUF4147)	1	9	2	2
PF13661	2OG-FeII_Oxy_4	2OG-Fe(II) oxygenase superfamily	1	0	0	0
PF13664	NA	Domain of unknown function (DUF4149)	1	0	0	0
PF13667	ThiC-associated	ThiC-associated domain	0	0	1	0
PF13669	Glyoxalase_4	Glyoxalase/Bleomycin resistance protein/Dioxygenase superfamily	2	2	2	0
PF13671	AAA_33	AAA domain	7	13	10	2
PF13672	PP2C_2	Protein phosphatase 2C	1	1	1	0
PF13673	Acetyltransf_10	Acetyltransferase (GNAT) domain	0	0	0	1
PF13676	TIR_2	TIR domain	26	33	98	11
PF13679	NA	Methyltransferase domain	2	13	8	0
PF13684	NA	Dihydroxyacetone kinase family	0	1	0	0
PF13688	Reprolysin_5	Metallo-peptidase family M12	10	10	13	4
PF13691	NA	tRNase Z endonuclease	2	0	0	0
PF13692	Glyco_trans_1_4	Glycosyl transferases group 1	1	1	2	0
PF13695	NA	Zinc-binding domain	2	13	4	0
PF13696	NA	Zinc knuckle	1	1	2	0
PF13704	NA	Glycosyl transferase family 2	1	1	2	0
PF13705	NA	TRC8 N-terminal domain	2	7	3	2
PF13714	PEP_mutase	Phosphoenolpyruvate phosphomutase	1	1	1	1
PF13715	NA	CarboxypepD_reg-like domain	1	0	0	0
PF13716	CRAL_TRIO_2	Divergent CRAL/TRIO domain	5	9	31	0
PF13718	GNAT_acetyltr_2	GNAT acetyltransferase 2	1	4	1	1
PF13725	tRNA_bind_2	Possible tRNA binding domain	1	4	1	1
PF13733	Glyco_transf_7N	N-terminal region of glycosyl transferase group 7	8	45	26	9
PF13738	Pyr_redox_3	Pyridine nucleotide-disulphide oxidoreductase	1	3	7	1
PF13750	NA	Bacterial Ig-like domain (group 3)	0	0	1	0
PF13764	NA	E3 ubiquitin-protein ligase UBR4	2	1	2	3
PF13768	NA	von Willebrand factor type A domain	7	21	18	2
PF13771	NA	PHD-like zinc-binding domain	4	11	36	0
PF13772	AIG2_2	AIG2-like family	3	3	2	3
PF13774	Longin	Regulated-SNARE-like domain	5	7	9	0
PF13793	Pribosyltran_N	N-terminal domain of ribose phosphate pyrophosphokinase	2	5	4	0
PF13802	Gal_mutarotas_2	Galactose mutarotase-like	5	2	4	0
PF13805	Pil1	Eisosome component PIL1	0	0	3	0
PF13812	PPR_3	Pentatricopeptide repeat domain	1	2	0	2
PF13815	NA	Iguana/Dzip1-like DAZ-interacting protein N-terminal	1	3	1	1
PF13820	NA	Putative nucleic acid-binding region	1	3	3	1
PF13821	NA	Domain of unknown function (DUF4187)	1	0	1	0
PF13831	PHD_2	PHD-finger	4	9	10	1
PF13832	zf-HC5HC2H_2	PHD-zinc-finger like domain	6	12	11	2
PF13833	EF-hand_8	EF-hand domain pair	31	65	121	8
PF13837	Myb_DNA-bind_4	Myb/SANT-like DNA-binding domain	6	1	19	2
PF13838	Clathrin_H_link	Clathrin-H-link	2	2	6	3
PF13839	PC-Esterase	GDSL/SGNH-like Acyl-Esterase family found in Pmr5 and Cas1p	3	3	6	0
PF13842	NA	DDE_Tnp_1-like zinc-ribbon	2	0	7	5
PF13843	NA	Transposase IS4	3	27	24	68
PF13844	Glyco_transf_41	Glycosyl transferase family 41	1	1	3	4
PF13847	Methyltransf_31	Methyltransferase domain	12	14	16	0
PF13848	Thioredoxin_6	Thioredoxin-like domain	9	6	10	2
PF13850	NA	Endoplasmic Reticulum-Golgi Intermediate Compartment (ERGIC)	3	7	5	0
PF13851	NA	Growth-arrest specific micro-tubule binding	1	1	1	1
PF13854	NA	Kelch motif	5	14	8	4
PF13855	LRR_8	Leucine rich repeat	92	226	294	271
PF13857	Ank_5	Ankyrin repeats (many copies)	35	134	98	38
PF13862	NA	p21-C-terminal region-binding protein	1	1	1	0
PF13863	NA	Domain of unknown function (DUF4200)	3	3	5	0
PF13864	NA	Calmodulin-binding	2	2	3	1
PF13865	NA	C-terminal duplication domain of Friend of PRMT1	1	4	1	0
PF13866	NA	SAP30 zinc-finger	1	2	2	0
PF13867	NA	Sin3 binding region of histone deacetylase complex subunit SAP30	1	2	2	0
PF13868	TPH	Trichohyalin-plectin-homology domain	5	5	8	4
PF13869	NUDIX_2	Nucleotide hydrolase	1	1	1	0
PF13870	NA	Domain of unknown function (DUF4201)	2	4	3	1
PF13871	NA	C-terminal domain on Strawberry notch homologue	3	1	3	0
PF13872	NA	P-loop containing NTP hydrolase pore-1	2	1	4	1
PF13873	NA	Myb/SANT-like DNA-binding domain	14	6	39	9
PF13874	Nup54	Nucleoporin complex subunit 54	1	2	2	0
PF13877	RPAP3_C	Potential Monad-binding region of RPAP3	1	13	3	1
PF13878	zf-C2H2_3	zinc-finger of acetyl-transferase ESCO	1	2	3	0
PF13879	NA	KIAA1430 homologue	1	5	10	1

PF13880	Acetyltransf_13	ESCO1/2 acetyl-transferase	1	2	3	0
PF13881	Rad60-SLD_2	Ubiquitin-2 like Rad60 SUMO-like	1	1	1	0
PF13882	NA	Bravo-like intracellular region	1	7	16	1
PF13884	NA	Chaperone of endosialidase	1	2	8	0
PF13885	NA	Keratin, high sulfur B2 protein	3	12	0	1
PF13886	NA	Domain of unknown function (DUF4203)	2	2	9	1
PF13887	NA	Myelin gene regulatory factor -C-terminal domain 1	1	2	8	0
PF13888	NA	Myelin gene regulatory factor C-terminal domain 2	0	2	8	0
PF13889	NA	Chromosome segregation during meiosis	1	1	1	0
PF13890	NA	Rab3 GTPase-activating protein catalytic subunit	1	1	1	1
PF13891	NA	Potential DNA-binding domain	2	6	6	1
PF13892	NA	DNA-binding domain	2	4	6	1
PF13893	RRM_5	RNA recognition motif. (a.k.a. RRM, RBD, or RNP domain)	9	32	30	0
PF13894	zf-C2H2_4	C2H2-type zinc finger	57	72	79	43
PF13895	Ig_2	Immunoglobulin domain	34	35	178	29
PF13896	NA	Glycosyl-transferase for dystroglycan	3	3	2	4
PF13897	GOLD_2	Golgi-dynamics membrane-trafficking	1	5	2	1
PF13898	NA	Domain of unknown function (DUF4205)	3	3	4	0
PF13899	Thioredoxin_7	Thioredoxin-like	2	10	3	0
PF13901	zf-RING_9	Putative zinc-RING and/or ribbon	3	7	6	1
PF13902	NA	R3H-associated N-terminal domain	1	2	1	0
PF13903	Claudin_2	PMP-22/EMP/MP20/Claudin tight junction	9	17	25	1
PF13904	NA	Domain of unknown function (DUF4207)	2	3	2	1
PF13905	Thioredoxin_8	Thioredoxin-like	5	8	7	3
PF13906	NA	C-terminus of AA_permease	1	3	14	1
PF13907	NA	Domain of unknown function (DUF4208)	1	4	5	0
PF13908	NA	Wnt and FGF inhibitory regulator	0	0	1	0
PF13909	zf-H2C2_5	C2H2-type zinc-finger domain	32	52	58	16
PF13910	NA	Domain of unknown function (DUF4209)	1	2	2	0
PF13911	NA	AhpC/TSA antioxidant enzyme	1	8	4	0
PF13912	zf-C2H2_6	C2H2-type zinc finger	134	132	97	60
PF13913	NA	zinc-finger of a C2HC-type	4	6	11	4
PF13915	NA	Domain of unknown function (DUF4210)	1	1	1	0
PF13917	NA	Zinc knuckle	2	2	3	1
PF13918	NA	PLD-like domain	1	4	2	0
PF13919	ASXH	Asx homology domain	1	1	1	0
PF13920	zf-C3HC4_3	Zinc finger, C3HC4 type (RING finger)	45	154	160	50
PF13921	Myb_DNA-bind_6	Myb-like DNA-binding domain	3	6	3	0
PF13922	NA	PHD domain of transcriptional enhancer, Asx	1	1	1	1
PF13923	zf-C3HC4_2	Zinc finger, C3HC4 type (RING finger)	5	3	4	6
PF13925	NA	con80 domain of Katanin	1	1	3	0
PF13926	NA	Domain of unknown function (DUF4211)	1	1	2	0
PF13927	Ig_3	Immunoglobulin domain	37	60	150	51
PF13931	NA	Kinesin-associated microtubule-binding	0	1	1	0
PF13932	GIDA_assoc	GidA associated domain	1	2	1	0
PF13934	NA	Nuclear pore complex assembly	0	1	1	0
PF13948	NA	Domain of unknown function (DUF4215)	1	0	0	0
PF13949	ALIX_LYPXL_bnd	ALIX V-shaped domain binding to HIV	2	5	8	1
PF13959	DUF4217	Domain of unknown function (DUF4217)	4	8	4	0
PF13960	NA	Domain of unknown function (DUF4218)	0	0	3	0
PF13964	NA	Kelch motif	9	4	27	2
PF13965	NA	dsRNA-gated channel SID-1	2	2	1	0
PF13966	NA	zinc-binding in reverse transcriptase	2	0	0	0
PF13967	NA	Late exocytosis, associated with Golgi transport	1	8	8	0
PF13971	NA	Meiosis-specific protein Mei4	0	1	1	0
PF13975	gag-asp_proteas	gag-polyprotein putative aspartyl protease	0	0	0	7
PF14008	Metallophos_C	Iron/zinc purple acid phosphatase-like protein C	5	14	5	3
PF14023	NA	Protein of unknown function (DUF4239)	1	0	2	0
PF14031	D-ser_dehydrat	Putative serine dehydratase domain	1	5	0	0
PF14048	NA	C-terminal domain of methyl-CpG binding protein 2 and 3	1	1	3	1
PF14050	NA	N-terminal conserved domain of Nudc.	1	1	1	0
PF14051	Requiem_N	N-terminal domain of DPF2/REQ.	1	7	6	0
PF14073	NA	Centrosome localisation domain of Cep57	2	1	6	1
PF14075	NA	Ubinuclein conserved middle domain	0	2	2	1
PF14113	Tae4	Type VI secretion system (T6SS), amidase effector protein 4	1	0	3	1
PF14124	NA	Domain of unknown function (DUF4291)	1	1	2	0
PF14138	NA	Cytochrome c oxidase assembly protein COX16	1	1	1	0
PF14160	NA	Centrosome-associated C terminus	1	1	9	1
PF14161	NA	Centrosome-associated N terminus	1	0	9	0
PF14186	NA	Cytoskeletal adhesion	0	0	2	1
PF14204	Ribosomal_L18_c	Ribosomal L18 C-terminal region	1	2	2	0
PF14214	NA	Helitron helicase-like domain at N-terminus	0	4	17	0
PF14216	NA	Domain of unknown function (DUF4326)	0	0	1	0
PF14222	NA	Cell morphogenesis N-terminal	1	1	5	1
PF14225	NA	Cell morphogenesis C-terminal	1	1	5	1
PF14226	DIOX_N	non-haem dioxygenase in morphine synthesis N-terminal	6	0	6	1
PF14228	NA	Cell morphogenesis central region	1	1	5	0

PF14229	NA	Domain of unknown function (DUF4332)	0	0	0	1
PF14237	NA	Domain of unknown function (DUF4339)	1	4	7	0
PF14240	NA	YHYH protein	0	0	7	0
PF14249	NA	Tocopherol cyclase	0	0	0	1
PF14252	NA	Domain of unknown function (DUF4347)	1	1	3	0
PF14259	NA	RNA recognition motif (a.k.a. RRM, RBD, or RNP domain)	17	42	44	0
PF14260	zf-C4pol	C4-type zinc-finger of DNA polymerase delta	2	4	1	0
PF14283	NA	Domain of unknown function (DUF4366)	0	1	0	0
PF14291	NA	Domain of unknown function (DUF4371)	0	4	13	4
PF14295	PAN_4	PAN domain	2	1	1	2
PF14304	NA	Transcription termination and cleavage factor C-terminal	0	1	3	0
PF14306	PUA_2	PUA-like domain	1	1	5	1
PF14309	NA	Domain of unknown function (DUF4378)	0	0	2	0
PF14310	Fn3-like	Fibronectin type III-like domain	15	5	3	2
PF14324	PINIT	PINIT domain	1	5	4	0
PF14327	CSTF2_hinge	Hinge domain of cleavage stimulation factor subunit 2	0	1	3	0
PF14360	NA	PAP2 superfamily C-terminal	2	1	1	1
PF14369	zinc_ribbon_9	zinc-ribbon	0	1	1	0
PF14370	Topo_C_assoc	C-terminal topoisomerase domain	1	1	3	0
PF14374	Ribos_L4_asso_C	60S ribosomal protein L4 C-terminal domain	1	1	2	0
PF14377	UBM	Domain of unknown function (DUF4414)	1	3	3	1
PF14381	NA	Ethylene-responsive protein kinase Le-CTR1	1	8	4	1
PF14382	ECR1_N	Exosome complex exonuclease RRP4 N-terminal region	2	2	2	1
PF14413	Thg1C	Thg1 C terminal domain	1	2	4	0
PF14429	DOCK-C2	C2 domain in Dock180 and Zizimin proteins	4	9	34	0
PF14437	MafB19-deam	MafB19-like deaminase	0	0	0	1
PF14438	SM-ATX	Ataxin 2 SM domain	1	0	3	0
PF14443	NA	DBC1	1	1	1	1
PF14444	NA	S1-like	1	2	3	2
PF14465	NA	NFRKB Winged Helix-like	0	3	3	1
PF14469	NA	28 kDa A-kinase anchor	1	2	2	0
PF14473	NA	RD3 protein	1	2	1	0
PF14478	DUF4430	Domain of unknown function (DUF4430)	3	4	0	0
PF14492	EFG_II	Elongation Factor G, domain II	5	7	6	1
PF14493	NA	Helix-turn-helix domain	0	3	3	0
PF14497	GST_C_3	Glutathione S-transferase, C-terminal domain	14	15	24	7
PF14500	MMS19_N	Dos2-interacting transcription regulator of RNA-Pol-II	1	1	1	0
PF14513	DAG_kinase_N	Diacylglycerol kinase N-terminus	1	1	1	0
PF14519	Macro_2	Macro-like domain	1	1	1	0
PF14520	HHH_5	Helix-hairpin-helix domain	0	2	5	0
PF14523	Syntaxin_2	Syntaxin-like protein	1	2	8	0
PF14529	Exo_endo_phos_2	Endonuclease-reverse transcriptase	23	17	7	4
PF14533	USP7_C2	Ubiquitin-specific protease C-terminal	3	2	5	0
PF14538	Raptor_N	Raptor N-terminal CASPase like domain	1	4	1	0
PF14542	Acetyltransf_CG	GCN5-related N-acetyl-transferase	0	2	0	0
PF14545	NA	Dof, BCAP, and BANK (DBB) motif,	2	9	13	0
PF14551	MCM_N	MCM N-terminal domain	7	9	6	0
PF14555	UBA_4	UBA-like domain	6	9	10	1
PF14559	TPR_19	Tetratricopeptide repeat	4	12	5	3
PF14560	Ubiquitin_2	Ubiquitin-like domain	4	1	1	1
PF14566	PTPlike_phytase	Inositol hexakisphosphate	1	6	2	0
PF14570	zf-RING_4	RING/Ubox like zinc-binding domain	1	4	4	0
PF14572	Pribosyl_synth	Phosphoribosyl synthetase-associated domain	2	2	3	0
PF14575	EphA2_TM	Ephrin type-A receptor 2 transmembrane domain	1	1	6	0
PF14580	LRR_9	Leucine-rich repeat	13	21	36	2
PF14593	PH_3	PH domain	1	6	4	0
PF14598	PAS_11	PAS domain	6	17	23	1
PF14599	NA	Zinc-ribbon	1	1	2	0
PF14604	SH3_9	Variant SH3 domain	25	78	191	12
PF14608	NA	RNA-binding, Nab2-type zinc finger	2	2	1	1
PF14617	NA	U3-containing 90S pre-ribosomal complex subunit	1	1	1	0
PF14619	SnAC	Snf2-ATP coupling, chromatin remodelling complex	1	2	1	0
PF14622	Ribonucleas_3_3	Ribonuclease-III-like	1	1	4	0
PF14625	NA	Lustrin, cysteine-rich repeated domain	4	0	4	1
PF14629	ORC4_C	Origin recognition complex (ORC) subunit 4 C-terminus	1	1	1	0
PF14630	ORC5_C	Origin recognition complex (ORC) subunit 5 C-terminus	1	2	1	0
PF14631	FancD2	Fanconi anaemia protein FancD2 nuclease	1	3	1	0
PF14632	NA	Acidic N-terminal SPT6	1	1	1	0
PF14633	SH2_2	SH2 domain	1	2	1	3
PF14634	zf-RING_5	zinc-RING finger domain	3	6	6	6
PF14635	HHH_7	Helix-hairpin-helix motif	1	1	1	1
PF14636	NA	Folliculin-interacting protein N-terminus	1	5	4	0
PF14637	NA	Folliculin-interacting protein middle domain	1	5	4	1
PF14638	NA	Folliculin-interacting protein C-terminus	1	5	4	1
PF14639	YqgF	Holliday-junction resolvase-like of SPT6	1	1	1	1
PF14640	NA	Transmembrane protein 223	1	1	1	0
PF14641	HTH_44	Helix-turn-helix DNA-binding domain of SPT6	1	1	1	0

PF14642	NA	FAM47 family	1	2	1	0
PF14643	NA	Domain of unknown function (DUF4455)	1	2	2	0
PF14644	NA	Domain of unknown function (DUF4456)	1	1	1	1
PF14645	NA	Chibby family	1	2	1	2
PF14646	NA	MYCBP-associated protein family	5	7	5	4
PF14647	NA	FAM91 N-terminus	2	1	1	0
PF14648	NA	FAM91 C-terminus	1	1	1	1
PF14649	NA	Spatacsin C-terminus	1	1	1	1
PF14651	Lipocalin_7	Lipocalin / cytosolic fatty-acid binding protein family	0	2	4	0
PF14652	NA	Domain of unknown function (DUF4457)	1	4	2	1
PF14655	NA	Rab3 GTPase-activating protein regulatory subunit N-terminus	1	1	1	0
PF14656	NA	Rab3 GTPase-activating protein regulatory subunit C-terminus	1	1	3	1
PF14658	NA	EF-hand domain	0	2	2	0
PF14661	NA	HAUS augmin-like complex subunit 6 N-terminus	1	2	0	0
PF14662	NA	Coiled-coil region of CCDC155 or KASH	1	0	2	0
PF14663	RasGEF_N_2	Rapamycin-insensitive companion of mTOR RasGEF_N domain	1	2	1	0
PF14664	RICTOR_N	Rapamycin-insensitive companion of mTOR, N-term	1	2	1	0
PF14666	RICTOR_M	Rapamycin-insensitive companion of mTOR, middle domain	1	2	1	0
PF14668	RICTOR_V	Rapamycin-insensitive companion of mTOR, domain 5	1	2	1	0
PF14670	FXa_inhibition	Coagulation Factor Xa inhibitory site	25	44	53	6
PF14671	DSPn	Dual specificity protein phosphatase, N-terminal half	1	1	6	0
PF14675	FANCI_S1	FANCI solenoid 1	1	1	1	0
PF14676	FANCI_S2	FANCI solenoid 2	1	1	1	1
PF14677	FANCI_S3	FANCI solenoid 3	1	1	1	1
PF14678	FANCI_S4	FANCI solenoid 4	1	2	1	0
PF14679	FANCI_HD1	FANCI helical domain 1	1	1	1	0
PF14680	FANCI_HD2	FANCI helical domain 2	1	1	1	0
PF14681	UPRTase	Uracil phosphoribosyltransferase	2	3	4	1
PF14687	DUF4460	Domain of unknown function (DUF4460)	1	1	2	0
PF14688	NA	Domain of unknown function (DUF4461)	1	1	2	0
PF14691	Fer4_20	Dihydroprymidine dehydrogenase domain II, 4Fe-4S cluster	2	3	5	0
PF14694	NA	Lines N-terminus	1	1	0	0
PF14695	NA	Lines C-terminus	0	1	0	0
PF14697	Fer4_21	4Fe-4S dicluster domain	1	1	1	0
PF14698	ASL_C2	Argininosuccinate lyase C-terminal	1	2	1	0
PF14699	NA	N-terminal domain from the human glycogen debranching enzyme	1	0	2	0
PF14700	RPOL_N	DNA-directed RNA polymerase N-terminal	1	1	1	0
PF14701	NA	glucanotransferase domain of human glycogen debranching enzyme	1	1	2	3
PF14702	NA	central domain of human glycogen debranching enzyme	1	1	2	1
PF14703	NA	Cytosolic domain of 10TM putative phosphate transporter	1	8	8	0
PF14704	NA	Dermatopontin	2	14	17	6
PF14705	NA	Costars	4	2	6	1
PF14707	Sulfatase_C	C-terminal region of aryl-sulfatase	2	1	4	1
PF14709	DND1_DSRM	double strand RNA binding domain from DEAD END PROTEIN 1	2	3	7	0
PF14712	NA	Snapin/Pallidin	2	7	5	0
PF14713	NA	Domain of unknown function (DUF4464)	2	3	1	0
PF14715	FixP_N	N-terminal domain of cytochrome oxidase-cbb3, FixP	0	1	0	0
PF14716	HHH_8	Helix-hairpin-helix domain	2	5	3	0
PF14719	NA	Phosphotyrosine interaction domain (PTB/PID)	2	2	2	4
PF14721	AIF_C	Apoptosis-inducing factor, mitochondrion-associated, C-term	1	2	1	1
PF14722	NA	Ki-ras-induced actin-interacting protein-IP3R-interacting domain	1	0	1	0
PF14723	NA	Sperm-specific antigen 2 C-terminus	1	0	0	0
PF14724	NA	Mitochondrial-associated sphingomyelin phosphodiesterase	1	1	2	2
PF14726	NA	Rotatin, an armadillo repeat protein, centriole functioning	1	1	1	0
PF14727	PHTB1_N	PTHB1 N-terminus	2	2	1	1
PF14728	PHTB1_C	PTHB1 C-terminus	1	2	1	1
PF14732	UAE_UbL	Ubiquitin/SUMO-activating enzyme ubiquitin-like domain	1	1	1	0
PF14735	NA	HAUS augmin-like complex subunit 4	1	2	1	0
PF14736	NA	Protein N-terminal asparagine amidohydrolase	1	1	2	0
PF14737	NA	Domain of unknown function (DUF4470)	1	1	1	0
PF14738	NA	Solute carrier (proton/amino acid symporter), TRAMD3 or PAT1	1	4	2	0
PF14739	NA	Domain of unknown function (DUF4472)	1	14	5	0
PF14740	NA	Domain of unknown function (DUF4471)	1	3	1	0
PF14743	DNA_ligase_OB_2	DNA ligase OB-like domain	1	2	1	0
PF14744	NA	WASH complex subunit 7	1	3	3	2
PF14745	NA	WASH complex subunit 7, N-terminal	1	3	1	1
PF14746	NA	WASH complex subunit 7, C-terminal	1	3	1	0
PF14748	P5CR_dimer	Pyrroline-5-carboxylate reductase dimerisation	1	2	2	0
PF14749	Acyl-CoA_ox_N	Acyl-coenzyme A oxidase N-terminal	2	2	6	0
PF14750	NA	Integrator complex subunit 2	2	4	1	2
PF14752	NA	Retinol binding protein receptor	4	32	14	5
PF14753	NA	Domain of unknown function (DUF4475)	2	2	5	1
PF14759	Reductase_C	Reductase C-terminal	1	5	5	0
PF14761	NA	Hermansky-Pudlak syndrome 3	0	1	1	1
PF14762	NA	Hermansky-Pudlak syndrome 3, middle region	0	1	2	0
PF14764	NA	AP-5 complex subunit, vesicle trafficking	2	1	1	1
PF14765	PS-DH	Polyketide synthase dehydratase	2	5	2	6

PF14766	NA	Replication protein A interacting N-terminal	1	2	3	0
PF14767	NA	Replication protein A interacting middle	1	0	3	0
PF14768	NA	Replication protein A interacting C-terminal	1	0	3	0
PF14769	NA	Flagellar C1a complex subunit C1a-32	3	5	5	0
PF14770	NA	Transmembrane protein 18	1	0	1	0
PF14771	NA	Domain of unknown function (DUF4476)	1	0	0	0
PF14772	NA	Sperm tail	1	2	4	0
PF14773	NA	Helicase-associated putative binding domain, C-terminal	0	1	0	0
PF14774	NA	FAM177 family	1	1	1	0
PF14775	NA	Sperm tail C-terminal domain	1	2	1	1
PF14776	NA	Cation-channel complex subunit UNC-79	1	3	6	1
PF14777	BBIP10	Cilia BBSome complex subunit 10	0	0	1	1
PF14778	NA	Olfactory receptor 4-like	1	2	1	1
PF14779	BBS1	Ciliary BBSome complex subunit 1	1	1	3	0
PF14780	NA	Domain of unknown function (DUF4477)	1	2	1	0
PF14781	BBS2_N	Ciliary BBSome complex subunit 2, N-terminal	1	0	1	0
PF14782	BBS2_C	Ciliary BBSome complex subunit 2, C-terminal	1	1	1	0
PF14783	BBS2_Mid	Ciliary BBSome complex subunit 2, middle region	1	1	1	0
PF14784	NA	C-terminal domain of the ECSIT protein	1	1	1	1
PF14786	NA	Tube Death domain	1	2	2	0
PF14788	NA	EF hand	1	1	5	0
PF14791	DNA_pol_B_thumb	DNA polymerase beta thumb	2	6	3	1
PF14792	DNA_pol_B_palm	DNA polymerase beta palm	2	6	3	0
PF14796	NA	Clathrin-adaptor complex-3 beta-1 subunit C-terminal	1	1	2	1
PF14798	Ca_hom_mod	Calcium homeostasis modulator	0	0	0	1
PF14799	NA	FAM195 family	1	1	1	0
PF14802	NA	TMEM192 family	1	1	3	0
PF14806	Coatomer_b_Cpla	Coatomer beta subunit appendage platform	1	1	1	1
PF14808	NA	TMEM164 family	2	1	7	1
PF14811	NA	Protein of unknown function TPD sequence-motif	1	3	1	0
PF14813	NADH_B2	NADH dehydrogenase 1 beta subcomplex subunit 2	1	0	0	0
PF14815	NUDIX_4	NUDIX domain	0	1	1	0
PF14816	NA	Family of unknown function, FAM178	1	1	5	0
PF14817	NA	HAUS augmin-like complex subunit 5	1	6	1	0
PF14821	Thr_synth_N	Threonine synthase N terminus	3	9	5	2
PF14822	Vasohibin	Vasohibin	1	1	2	0
PF14825	NA	Domain of unknown function (DUF4483)	1	4	2	0
PF14826	FACT-Spt16_Nlob	FACT complex subunit SPT16 N-terminal lobe domain	1	2	1	0
PF14828	NA	Amnionless	1	1	2	1
PF14830	Haemocyan_bet_s	Haemocyanin beta-sandwich	0	6	0	25
PF14833	NAD_binding_11	NAD-binding of NADP-dependent 3-hydroxyisobutyrate dehydrogenase	2	2	3	0
PF14835	NA	zf-RING of BARD1-type protein	2	4	0	0
PF14836	Ubiquitin_3	Ubiquitin-like domain	2	1	4	1
PF14837	NA	Integrator complex subunit 5 N-terminus	1	1	1	1
PF14838	NA	Integrator complex subunit 5 C-terminus	1	1	1	1
PF14839	NA	DOR family	1	1	4	1
PF14843	GF_recep_IV	Growth factor receptor domain IV	2	2	1	1
PF14844	PH_BEACH	PH domain associated with Beige/BEACH	5	9	20	0
PF14845	Glycohydro_20b2	beta-acetyl hexosaminidase like	4	9	9	0
PF14846	NA	Domain of unknown function (DUF4485)	1	1	6	0
PF14851	NA	FAM176 family	1	1	2	0
PF14852	Fis1_TPR_N	Fis1 N-terminal tetratricopeptide repeat	1	1	2	0
PF14853	Fis1_TPR_C	Fis1 C-terminal tetratricopeptide repeat	1	3	2	0
PF14854	NA	Leucine rich adaptor protein	1	6	11	1
PF14857	NA	TMEM151 family	1	1	1	2
PF14858	NA	Domain of unknown function (DUF4486)	1	1	1	2
PF14862	Defensin_big	Big defensin	0	0	3	0
PF14863	Alkyl_sulf_dimr	Alkyl sulfatase dimerisation	1	0	2	0
PF14864	Alkyl_sulf_C	Alkyl sulfatase C-terminal	1	0	1	0
PF14868	NA	Domain of unknown function (DUF4487)	0	2	2	0
PF14874	NA	Flagellar-associated PapD-like	2	3	14	0
PF14875	NA	N-term cysteine-rich ER, FAM69	4	5	4	0
PF14878	NA	Death-like domain of SPT6	1	2	1	1
PF14881	NA	Tubulin domain	1	1	1	0
PF14886	NA	FAM183A and FAM183B related	1	0	1	0
PF14892	DUF4490	Domain of unknown function (DUF4490)	1	6	2	1
PF14893	NA	PNMA	1	0	5	3
PF14895	NA	Protein phosphatase 1 inhibitor	1	1	3	0
PF14901	NA	Cleavage inducing molecular chaperone	0	1	2	0
PF14904	NA	Family of unknown function	1	0	1	0
PF14906	NA	Domain of unknown function (DUF4495)	1	1	1	0
PF14908	NA	Domain of unknown function (DUF4496)	1	1	4	0
PF14909	NA	Spermatogenesis-assoc protein 6	1	3	4	1
PF14910	NA	S-phase genomic integrity recombination mediator, N-terminal	1	0	1	0
PF14911	NA	S-phase genomic integrity recombination mediator, C-terminal	1	0	1	0
PF14912	NA	Testicular haploid expressed repeat	2	2	10	4
PF14913	NA	DPCD protein family	1	1	1	0

PF14915	NA	CCDC144C protein coiled-coil region	0	1	4	0
PF14916	NA	Coiled-coil domain of unknown function	1	2	4	0
PF14917	NA	Coiled coil protein 74, C terminal	0	1	3	0
PF14918	NA	MDM2-binding	1	1	1	0
PF14919	NA	MDM2-binding	1	0	1	0
PF14920	NA	MDM2-binding	0	1	1	0
PF14921	NA	Adenomatosis polyposis coli down-regulated 1	0	2	1	1
PF14922	NA	Protein of unknown function	2	2	6	0
PF14923	NA	Coiled-coil protein 142	1	2	2	0
PF14924	NA	Protein of unknown function (DUF4497)	1	1	1	1
PF14925	NA	Domain of unknown function	1	1	0	0
PF14926	NA	Domain of unknown function (DUF4498)	1	2	1	0
PF14927	NA	Neurensin	1	1	1	0
PF14929	NA	TAF RNA Polymerase I subunit A	0	1	2	0
PF14931	NA	Intraflagellar transport complex B, subunit 20	1	1	1	0
PF14932	NA	HAUS augmin-like complex subunit 3	1	1	2	0
PF14933	NA	CEP19-like protein	2	1	1	1
PF14934	NA	Domain of unknown function (DUF4499)	1	0	1	0
PF14935	NA	Transmembrane protein 138	1	1	2	0
PF14936	NA	Tumour protein p53-inducible protein 11	1	4	7	0
PF14937	NA	Domain of unknown function (DUF4500)	1	1	1	0
PF14938	SNAP	Soluble NSF attachment protein, SNAP	2	3	4	0
PF14939	DCAF15_WD40	DDB1-and CUL4-substrate receptor 15, WD repeat	1	1	1	1
PF14940	NA	Transmembrane 219	1	2	1	0
PF14941	NA	Transcriptional regulator, Out at first	1	2	1	2
PF14942	NA	Organelle biogenesis, Muted-like protein	1	1	1	0
PF14943	MRP-S26	Mitochondrial ribosome subunit S26	1	1	2	0
PF14945	NA	Normal lung function maintenance, Low in Lung Cancer 1 protein	2	0	8	0
PF14949	NA	ARF7 effector protein C-terminus	1	3	1	1
PF14950	NA	Domain of unknown function (DUF4502)	1	1	3	0
PF14951	NA	Domain of unknown function (DUF4503)	1	1	3	0
PF14952	NA	Putative treble-clef, zinc-finger, Zn-binding	1	1	1	1
PF14953	NA	Domain of unknown function (DUF4504)	1	1	1	0
PF14954	NA	Limb expression 1	1	3	1	1
PF14955	MRP-S24	Mitochondrial ribosome subunit S24	1	1	2	0
PF14956	NA	Domain of unknown function (DUF4505)	1	1	1	0
PF14958	NA	Domain of unknown function (DUF4506)	1	1	2	0
PF14959	NA	gamma-Secretase-activating protein C-term	1	1	1	0
PF14961	NA	Broad-minded protein	1	2	4	1
PF14963	NA	Calcium signal-modulating cyclophilin ligand	1	0	1	0
PF14964	NA	Domain of unknown function (DUF4507)	1	7	1	3
PF14966	NA	DNA repair REX1-B	1	1	2	1
PF14968	NA	Coiled coil protein 84	1	4	3	0
PF14969	NA	Domain of unknown function (DUF4508)	1	1	0	0
PF14970	NA	Domain of unknown function (DUF4509)	1	1	1	0
PF14972	NA	Mitochondrial morphogenesis regulator	0	3	2	1
PF14973	NA	TERF1-interacting nuclear factor 2 N-terminus	1	0	1	0
PF14974	NA	Domain of unknown function (DUF4511)	1	1	2	0
PF14975	NA	Domain of unknown function (DUF4512)	1	0	0	0
PF14976	NA	FAM72 protein	1	1	1	0
PF14977	NA	FAM194 protein	2	8	18	1
PF14978	MRP-63	Mitochondrial ribosome protein 63	1	1	1	0
PF14979	NA	Transmembrane 52	0	3	0	0
PF14983	NA	Domain of unknown function (DUF4513)	1	2	1	0
PF14988	NA	Domain of unknown function (DUF4515)	3	4	7	1
PF14989	NA	Coiled-coil domain containing 32	1	1	1	1
PF14994	NA	Testis-specific gene 13 protein	0	1	10	0
PF14995	NA	Transmembrane protein	1	2	2	0
PF14996	NA	Retinal Maintenance	1	1	2	0
PF14997	NA	CECR6/TMEM121 family	1	5	6	2
PF15000	NA	Tumour suppressor candidate 2	1	0	1	0
PF15001	NA	AP-5 complex subunit sigma-1	1	1	1	1
PF15002	NA	ERK and JNK pathways, inhibitor	1	1	1	0
PF15003	NA	HAUS augmin-like complex subunit 2	1	1	3	0
PF15004	NA	Myeloma-overexpressed-like	1	0	0	0
PF15006	NA	Domain of unknown function (DUF4517)	1	2	2	0
PF15007	NA	Centrosomal spindle body, CEP44	1	0	1	0
PF15008	NA	Domain of unknown function (DUF4518)	1	2	1	1
PF15009	TMEM173	Transmembrane protein 173	0	0	21	1
PF15011	NA	Casein Kinase 2 substrate	1	2	1	0
PF15014	NA	Ceroid-lipofuscinosis neuronal protein 5	1	2	1	0
PF15016	NA	Domain of unknown function (DUF4520)	1	1	1	0
PF15017	WRNPLPNID	Drug resistance and apoptosis regulator	0	0	4	0
PF15018	NA	TRP-interacting helix	0	0	0	1
PF15019	C9orf72-like	C9orf72-like protein family	1	2	1	0
PF15023	NA	Protein of unknown function (DUF4523)	1	2	0	0
PF15024	Glyco_transf_18	Glycosyltransferase family 18	1	6	3	0

PF15025	NA	Domain of unknown function (DUF4524)	1	1	1	1
PF15027	NA	Domain of unknown function (DUF4525)	0	6	3	0
PF15030	NA	Protein of unknown function (DUF4527)	0	0	2	0
PF15031	NA	Domain of unknown function (DUF4528)	1	0	0	0
PF15035	NA	Ciliary rootlet component, centrosome cohesion	0	1	8	1
PF15038	NA	Jiraiya	1	0	2	1
PF15043	NA	CB1 cannabinoid receptor-interacting protein 1	1	0	1	0
PF15044	NA	Mitochondrial function, CLU-N-term	1	1	1	0
PF15045	NA	Clathrin-binding box of Aftiphilin, vesicle trafficking	1	4	2	0
PF15046	NA	Protein of unknown function (DUF4532)	1	0	4	1
PF15050	NA	SCIMP protein	0	0	1	0
PF15052	NA	TMEM169 protein family	1	2	1	0
PF15053	NA	Mjmu-R1-like protein family	1	2	1	0
PF15057	NA	Domain of unknown function (DUF4537)	1	4	5	3
PF15059	NA	Speriolin C-terminus	1	0	2	2
PF15065	NCU-G1	Lysosomal transcription factor, NCU-G1	1	2	1	2
PF15067	NA	FAM124 family	2	4	4	2
PF15068	NA	FAM101 family	1	1	1	1
PF15070	NA	Putative golgin subfamily A member 2-like protein 5	1	1	3	1
PF15071	NA	Transmembrane family 220, helix	1	0	2	0
PF15072	NA	Domain of unknown function (DUF4539)	1	5	1	0
PF15073	NA	Domain of unknown function (DUF4540)	1	1	2	0
PF15074	NA	Domain of unknown function (DUF4541)	2	4	3	0
PF15075	NA	Domain of unknown function (DUF4542)	1	1	3	2
PF15082	NA	Domain of unknown function (DUF4549)	1	1	4	1
PF15084	NA	Domain of unknown function (DUF4550)	0	1	1	0
PF15087	NA	Protein of unknown function (DUF4551)	1	3	6	0
PF15092	NA	Uncharacterised protein family UPF0728	1	1	0	0
PF15093	NA	Domain of unknown function (DUF4555)	1	1	1	1
PF15100	NA	TMEM187 protein family	1	5	2	1
PF15101	TERB2	Domain of unknown function (DUF4557)	1	0	2	0
PF15102	NA	TMEM154 protein family	0	1	3	0
PF15104	DUF4558	Domain of unknown function (DUF4558)	1	0	2	0
PF15107	NA	FAM216B protein family	0	0	2	1
PF15110	NA	TMEM141 protein family	0	2	0	0
PF15111	NA	TMEM101 protein family	1	1	1	1
PF15112	NA	Domain of unknown function (DUF4559)	1	2	1	0
PF15113	NA	TMEM117 protein family	1	3	1	4
PF15114	NA	Uncharacterised protein family UPF0640	1	0	0	0
PF15115	NA	Domain of unknown function with conserved HDNR motif	2	2	3	0
PF15123	NA	Domain of unknown function (DUF4562)	1	5	4	1
PF15130	NA	Domain of unknown function (DUF4566)	0	1	1	0
PF15134	NA	Domain of unknown function (DUF4570)	1	2	2	0
PF15135	NA	Uncharacterised protein UPF0515	0	9	4	1
PF15136	NA	Uncharacterised protein family UPF0449	0	1	2	0
PF15139	NA	Domain of unknown function (DUF4572)	1	1	1	0
PF15140	NA	Domain of unknown function (DUF4573)	3	2	0	1
PF15146	NA	Fanconi anemia-associated	1	0	0	0
PF15156	NA	Ceroid-lipofuscinosis neuronal protein 6	1	2	2	0
PF15160	NA	Spermatogenesis-associated serine-rich protein 1	1	1	6	0
PF15162	NA	Domain of unknown function (DUF4580)	1	1	0	0
PF15163	Meiosis_expr	Meiosis-expressed	1	4	1	0
PF15165	NA	Meiotic recombination protein REC114-like	1	3	2	0
PF15167	NA	Domain of unknown function (DUF4581)	1	0	1	0
PF15168	NA	Triple QxxK/R motif-containing protein family	0	0	1	0
PF15169	NA	Domain of unknown function (DUF4564)	1	5	3	0
PF15175	NA	Spermatogenesis-associated protein 24	1	1	1	1
PF15189	NA	Domain of unknown function (DUF4582)	1	4	2	0
PF15190	NA	Domain of unknown function (DUF4583)	0	2	0	0
PF15227	zf-C3HC4_4	zinc finger of C3HC4-type, RING	10	3	5	0
PF15228	NA	Death-associated protein	1	1	1	0
PF15230	NA	Serine/arginine repetitive matrix protein C-terminus	0	0	1	0
PF15233	NA	Synaptonemal complex central element protein 1	1	0	2	0
PF15236	NA	Coiled-coil domain-containing protein 66	1	1	12	0
PF15238	NA	FAM181	1	0	2	2
PF15239	NA	Domain of unknown function (DUF4586)	1	2	3	0
PF15242	NA	Family of FAM53	2	6	9	0
PF15243	ANAPC15	Anaphase-promoting complex subunit 15	1	1	1	0
PF15244	NA	Spermatogenesis-associated protein 7, or HSD3	1	3	2	0
PF15245	NA	Transcription cofactor vestigial-like protein 4	1	0	3	0
PF15247	SLBP_RNA_bind	Histone RNA hairpin-binding protein RNA-binding domain	1	2	1	0
PF15248	NA	Domain of unknown function (DUF4587)	0	0	5	0
PF15249	NA	Conserved region of unknown function on GLTSCR protein	1	2	1	0
PF15250	NA	Raftlin	2	0	1	0
PF15251	NA	Domain of unknown function (DUF4588)	1	1	1	1
PF15253	STIL_N	SCL-interrupting locus protein N-terminus	1	1	2	1
PF15254	NA	Coiled-coil domain-containing protein 14	1	0	2	0

PF15255	NA	WASH complex subunit CAP-Z interacting, central region	1	1	3	0
PF15256	NA	SPATIAL	1	3	7	0
PF15257	NA	Domain of unknown function (DUF4590)	2	2	8	1
PF15259	NA	G-2 and S-phase expressed 1	1	0	2	0
PF15260	NA	Protein family FAM219A	1	1	1	0
PF15261	NA	Domain of unknown function (DUF4591)	0	1	5	1
PF15262	NA	Domain of unknown function (DUF4592)	0	0	3	0
PF15264	NA	Tumour suppressing sub-chromosomal transferable candidate 4	1	1	1	1
PF15266	NA	Domain of unknown function (DUF4594)	1	1	6	1
PF15275	NA	PEHE domain	2	3	3	0
PF15276	PP1_bind	Protein phosphatase 1 binding	0	1	6	0
PF15277	Sec3-PIP2_bind	Exocyst complex component SEC3 N-terminal PIP2 binding PH	1	1	0	1
PF15279	NA	Sine oculis-binding protein	1	2	4	1
PF15280	NA	Protein aurora borealis N-terminus	1	1	1	0
PF15285	BH3	Beclin-1 BH3 domain, Bcl-2-interacting	1	0	1	0
PF15287	NA	KRBA1 family repeat	0	0	0	1
PF15288	NA	Zinc knuckle	1	1	1	1
PF15289	RFXA_RFXANK_bdg	Regulatory factor X-associated C-terminal binding domain	1	0	0	0
PF15290	NA	Golgi-localised syntaxin-1-binding clamp	0	0	0	1
PF15292	NA	Treslin N-terminus	1	4	1	0
PF15294	NA	Leucine zipper	1	2	2	1
PF15295	NA	Coiled-coil domain-containing protein 50 N-terminus	1	5	3	0
PF15296	NA	Codanin-1 C-terminus	1	0	1	0
PF15297	NA	Cytoskeleton-associated protein 2 C-terminus	0	1	2	0
PF15299	NA	Amyotrophic lateral sclerosis 2 chromosomal region candidate gene 8	3	1	8	0
PF15300	NA	INTS6/SAGE1/DDX26B/CT45 C-terminus	1	1	4	0
PF15301	NA	SLAIN motif-containing family	1	2	2	0
PF15305	NA	Intraflagellar transport protein 43	1	1	3	0
PF15306	NA	LIN37	1	1	1	0
PF15309	NA	ALMS motif	1	2	4	0
PF15311	NA	Hydrolethalus syndrome protein 1 C-terminus	1	9	1	0
PF15313	HEXIM	Hexamethylene bis-acetamide-inducible protein	1	1	2	1
PF15319	NA	RAD9, RAD1, HUS1-interacting nuclear orphan protein	1	1	2	0
PF15320	NA	mRNA cap methylation, RNMT-activating mini protein	1	1	2	0
PF15323	NA	Developmental protein	1	1	1	0
PF15324	NA	Hedgehog signalling target	1	2	1	0
PF15328	NA	Putative GRINL1B complex locus protein 2	1	6	2	0
PF15335	NA	Caspase activity and apoptosis inhibitor 1	1	1	2	0
PF15336	NA	Autism susceptibility gene 2 protein	1	2	5	0
PF15337	NA	Vascular protein family Vasculin-like 1	0	1	1	0
PF15341	NA	Ribosome biogenesis protein SLX9	1	0	3	0
PF15344	NA	FAM217 family	1	0	6	0
PF15345	NA	Transmembrane protein 51	0	0	1	0
PF15346	NA	Arginine and glutamate-rich 1	2	1	1	1
PF15348	NA	Gemini of Cajal bodies-associated protein 8	1	1	1	1
PF15352	NA	Susceptibility to monomelic amyotrophy	0	2	4	0
PF15353	NA	Headcase protein family homologue	1	1	2	1
PF15359	NA	Carnitine deficiency-associated protein 3	1	1	3	0
PF15361	NA	Resistance to inhibitors of cholinesterase homologue 3	1	1	1	0
PF15364	NA	PAXIP1-associated-protein-1 C term PTIP binding protein	0	3	2	0
PF15365	PNRC	Proline-rich nuclear receptor coactivator	1	0	0	0
PF15369	NA	Uncharacterised protein KIAA1328	0	0	1	0
PF15370	NA	Domain of unknown function (DUF4598)	0	1	1	0
PF15372	NA	Domain of unknown function (DUF4600)	2	1	12	0
PF15373	NA	Domain of unknown function (DUF4601)	1	0	1	0
PF15375	DUF4602	Domain of unknown function (DUF4602)	1	2	1	0
PF15376	NA	Domain of unknown function (DUF4603)	1	0	1	0
PF15377	NA	Domain of unknown function (DUF4604)	1	1	1	0
PF15378	NA	Domain of unknown function (DUF4605)	1	1	2	1
PF15379	NA	Domain of unknown function (DUF4606)	1	0	1	0
PF15383	NA	Transmembrane protein 237	1	1	1	1
PF15384	PAXX	PAXX, PAralog of XRCC4 and XLF, also called C9orf142	1	1	1	1
PF15386	NA	Drosophila Tantalus-like	0	0	2	0
PF15387	NA	Domain of unknown function (DUF4611)	0	1	1	0
PF15388	NA	Protein Family FAM117	1	2	1	1
PF15389	NA	Domain of unknown function (DUF4612)	1	1	9	0
PF15390	NA	Domain of unknown function (DUF4613)	1	4	1	0
PF15391	NA	Domain of unknown function (DUF4614)	1	1	2	1
PF15392	NA	Joubert syndrome-associated	0	0	0	2
PF15393	NA	Domain of unknown function (DUF4615)	0	2	0	0
PF15396	NA	Protein Family FAM60A	1	0	2	1
PF15397	NA	Domain of unknown function (DUF4618)	1	1	2	0
PF15400	NA	Testis-expressed sequence 33 protein family	1	8	3	1
PF15405	NA	Pleckstrin homology domain	1	1	2	0
PF15409	NA	Pleckstrin homology domain	1	6	3	0
PF15410	PH_9	Pleckstrin homology domain	4	16	28	3
PF15412	NA	Binding domain of Nse4/EID3 to Nse3-MAGE	1	1	2	0

PF15430	NA	Single domain von Willebrand factor type C	0	1	3	0
PF15433	MRP-S31	Mitochondrial 28S ribosomal protein S31	1	1	1	0
PF15450	NA	Domain of unknown function (DUF4631)	0	2	21	0
PF15454	LAMTOR	Late endosomal/lysosomal adaptor and MAPK and MTOR activator	1	1	1	0
PF15458	NA	Nineteen complex-related protein 2	0	1	0	0
PF15459	NA	60S ribosome biogenesis protein Rrp14	0	8	0	0
PF15469	Sec5	Exocyst complex component Sec5	1	1	5	0
PF15471	NA	Transmembrane protein family 171	1	0	0	0
PF15472	NA	Domain of unknown function (DUF4638)	0	1	0	0
PF15473	NA	PEST, proteolytic signal-containing nuclear protein family	1	1	1	0
PF15475	NA	Transmembrane protein C12orf23, UPF0444	1	0	1	0
PF15477	NA	Small acidic protein family	3	4	6	2
PF15478	NA	Family of unknown function with LKAAEAR motif	1	1	1	0
PF15479	NA	Domain of unknown function (DUF4639)	1	3	1	0
PF15487	NA	FAM220 family	1	1	1	1
PF15489	NA	CST, telomere maintenance, complex subunit CTC1	1	1	2	0
PF15490	Ten1_2	Telomere-capping, CST complex subunit	1	1	2	0
PF15492	NA	Neuroblastoma-amplified sequence, N terminal	1	1	0	0
PF15494	NA	Scavenger receptor cysteine-rich domain	1	0	5	0
PF15497	NA	snRNA-activating protein complex subunit 19, SNAPc subunit 19	0	1	2	0
PF15499	NA	Ubiquitin-specific peptidase-like, SUMO isopeptidase	1	8	1	3
PF15501	NA	Nuclear protein MDM1	1	1	9	0
PF15502	NA	M-phase-specific PLK1-interacting protein	0	3	0	0
PF15503	NA	Protein phosphatase 1 regulatory subunit 35 C-terminus	1	2	1	0
PF15508	NAAA-beta	beta subunit of N-acylethanolamine-hydrolyzing acid amidase	3	2	2	0
PF15510	CENP-W	Centromere kinetochore component W	0	1	0	0
PF15511	CENP-T_C	Centromere kinetochore component CENP-T histone fold	22	3	4	0
PF15519	RBM39linker	linker between RRM2 and RRM3 domains in RBM39 protein	1	1	3	1
PF15539	NA	CAF1 complex subunit p150, region binding to CAF1-p60 at C-term	0	1	1	0
PF15554	NA	FSIP1 family	1	1	2	0
PF15558	NA	Domain of unknown function (DUF4659)	1	1	1	0
PF15559	NA	Domain of unknown function (DUF4660)	0	1	0	0
PF15612	WHIM1	WSTF, HB1, Itc1p, MBD9 motif 1	3	3	6	1
PF15613	NA	WSTF, HB1, Itc1p, MBD9 motif 2	0	0	4	2
PF15614	NA	WSTF, HB1, Itc1p, MBD9 motif 3	3	2	19	0
PF15619	NA	Ciliary protein causing Leber congenital amaurosis disease	1	1	0	0
PF15625	NA	CC2D2A N-terminal C2 domain	1	6	3	0
PF15627	NA	CEP76 C2 domain	1	1	2	1
PF15630	CENP-S	Kinetochore component CENP-S	1	1	1	0
PF15633	NA	HYD1 signature containing ADP-ribosyltransferase	1	0	0	0
PF15636	Tox-GHH	GHH signature containing HNH/Endo VII superfamily nuclease toxin	1	1	17	1
PF15663	zf-CCCH_3	Zinc-finger containing family	2	2	4	0
PF15665	NA	Family with sequence similarity 184, A and B	1	1	1	0
PF15667	NA	Protein of unknown function with motif GDWWSH	1	2	4	0
PF15668	NA	Domain of unknown function (DUF4663)	1	0	1	0
PF15669	NA	Coiled-coil domain-containing protein 24 family	1	3	2	0
PF15674	CCDC23	Coiled-coil domain-containing protein 23	1	2	1	0
PF15676	NA	Six6 opposite strand transcript 1 family	1	1	4	0
PF15678	NA	Centriole duplication and mitotic chromosome congression	1	4	1	1
PF15679	NA	Domain of unknown function (DUF4665)	1	0	3	0
PF15684	NA	Active regulator of SIRT1, or 40S ribosomal protein S19-binding 1	1	1	1	0
PF15691	NA	Protein phosphatase 1 regulatory subunit 32	1	3	3	2
PF15692	NA	NF-kappa-B-activating protein	1	1	0	0
PF15693	NA	Mediator complex subunit 26 C-terminal	1	1	1	1
PF15696	NA	RAD51 interacting motif	1	1	1	0
PF15702	NA	Hermansky-Pudlak syndrome 6 protein	1	0	3	0
PF15705	NA	Mature oligodendrocyte transmembrane protein, TMEM132D, N-term	1	1	0	0
PF15706	NA	Mature oligodendrocyte transmembrane protein, TMEM132D, C-term	0	1	1	1
PF15709	NA	Domain of unknown function (DUF4670)	0	1	1	0
PF15711	ILEI	Interleukin-like EMT inducer	5	2	3	1
PF15715	PAF	PCNA-associated factor	1	1	1	0
PF15717	NA	Pericentriolar material 1 C terminus	1	1	2	3
PF15718	NA	Domain of unknown function (DUF4673)	1	4	3	2
PF15719	NA	Domain of unknown function (DUF4674)	1	2	2	0
PF15733	NA	Domain of unknown function (DUF4682)	0	0	2	1
PF15734	NA	Migration and invasion-inhibitory	0	1	2	0
PF15739	NA	Translin-associated factor X-interacting N-terminus	3	4	4	0
PF15742	NA	Domain of unknown function (DUF4686)	1	2	18	0
PF15743	NA	Spermatogenesis-associated C-terminus	1	1	11	1
PF15745	NA	AP-1 complex-associated regulatory protein	1	1	1	0
PF15748	NA	Centriole, cilia and spindle-associated	1	4	1	0
PF15749	NA	Uncharacterised protein family UPF0544	1	1	1	0
PF15750	NA	Ubiquitin-binding zinc-finger	1	0	1	0
PF15753	NA	Biogenesis of lysosome-related organelles complex 1 subunit 3	1	1	8	0
PF15766	NA	Domain of unknown function (DUF4695)	1	2	1	0
PF15769	NA	Domain of unknown function (DUF4698)	1	3	8	1
PF15778	NA	Cation channel complex component UNC80	1	1	4	1

PF15780	NA	Abnormal spindle-like microcephaly-assoc'd, ASPM-SPD-2-Hydin	3	1	3	2
PF15784	GPS2_interact	G-protein pathway suppressor 2-interacting domain	1	1	10	0
PF15785	SMG1	Serine/threonine-protein kinase smg-1	1	2	1	2
PF15786	NA	PET assembly of cytochrome c oxidase, mitochondrial	1	0	0	0
PF15787	NA	Domain of unknown function (DUF4704)	1	15	20	0
PF15790	NA	E1A-binding protein p400, N-terminal	0	1	4	1
PF15795	NA	Ectodermal ciliogenesis protein	2	3	7	3
PF15796	NA	KELK-motif containing domain of MRCK Ser/Thr protein kinase	0	1	8	0
PF15797	NA	Domain of unknown function (DUF4706)	1	1	1	0
PF15798	PRAS	Proline-rich AKT1 substrate 1	1	1	2	0
PF15800	NA	Clock interacting protein circadian	1	1	2	1
PF15801	NA	zf-MYND-like zinc finger, mRNA-binding	1	1	2	0
PF15802	NA	DDB1- and CUL4-associated factor 17	1	1	2	0
PF15803	NA	Zinc-finger of sodium channel modifier 1	1	0	1	0
PF15805	NA	Acidic C-terminal region of sodium channel modifier 1 SCNM1	0	5	1	0
PF15806	NA	Domain of unknown function (DUF4707)	0	1	0	0
PF15810	NA	Coiled-coil domain-containing protein 117	0	4	0	0
PF15811	NA	Small VCP/p97-interacting protein	1	0	2	0
PF15813	NA	Domain of unknown function (DUF4708)	1	0	1	0
PF15814	NA	Protein family FAM199X	0	0	1	0
PF15818	NA	Coiled-coil domain-containing protein 73 family	1	0	1	0
PF15821	NA	Domain of unknown function (DUF4709)	1	1	10	0
PF15860	NA	Domain of unknown function (DUF4728)	1	0	1	0
PF15865	NA	Fanconi anaemia group A protein N terminus	1	1	0	0
PF15867	NA	Dynein attachment factor N-terminus	1	1	1	0
PF15870	NA	ElonginA binding-protein 1	1	1	3	1
PF15871	NA	Junction-mediating and -regulatory protein	1	2	1	1
PF15873	NA	Domain of unknown function (DUF4730)	0	0	0	1
PF15874	NA	Putative Interleukin 2 receptor, gamma chain	5	6	4	0
PF15877	NA	Transmembrane protein family 232	1	1	1	1
PF15880	NDUFV3	NADH dehydrogenase [ubiquinone] flavoprotein 3, mitochondrial	0	1	1	0
PF15882	NA	Domain of unknown function (DUF4735)	4	4	3	2
PF15884	NA	Protein QIL1	0	1	3	0
PF15886	CBM39	Carbohydrate binding domain (family 32)	1	0	2	0
PF15891	NA	Nucleoside 2-deoxyribosyltransferase like	1	1	4	0
PF15898	PRKG1_interact	cGMP-dependent protein kinase interacting domain	1	2	3	0
PF15901	Sortilin_C	Sortilin, neurotensin receptor 3, C-terminal	3	3	4	0
PF15902	Sortilin-Vps10	Sortilin, neurotensin receptor 3,	3	3	4	3
PF15903	NA	Filopodia upregulated, FAM65	1	10	11	1
PF15904	NA	LKB1 serine/threonine kinase interacting protein 1	0	1	1	0
PF15905	NA	Hyaluronan mediated motility receptor N-terminal	0	0	1	0
PF15906	NA	Zinc-finger of nitric oxide synthase-interacting protein	1	0	5	0
PF15907	NA	Integrin-alpha FG-GAP repeat-containing protein 2	1	2	1	0
PF15908	NA	Hyaluronan mediated motility receptor C-terminal	0	2	1	0
PF15911	NA	WD domain, G-beta repeat	1	1	0	0
PF15912	NA	Virilizer, N-terminal	1	1	4	0
PF15913	Furin-like_2	Furin-like repeat, cysteine-rich	1	6	2	0
PF15914	NA	FAM193 family C-terminal	0	4	1	0
PF15916	NA	Domain of unknown function (DUF4743)	1	4	1	0
PF15917	PIEZO	Piezo	1	1	0	0
PF15920	NA	N-terminal of Junction-mediating and WASP homolog-associated	1	0	1	1
PF15921	NA	Coiled-coil domain-containing protein 158	0	2	4	0
PF15924	NA	ALG11 mannosyltransferase N-terminus	1	2	1	0
PF15925	NA	SOSS complex subunit C	1	1	1	0
PF15926	NA	E3 ubiquitin-protein ligase RNF220	1	1	3	0
PF15927	NA	Cancer susceptibility candidate 1 N-terminus	1	1	4	0
PF15936	NA	Domain of unknown function (DUF4749)	2	8	12	1
PF15949	NA	Domain of unknown function (DUF4757)	0	1	1	1
PF15951	NA	MITF/TFEB/TFEC/TFE3 N-terminus	0	1	3	2
PF15961	NA	Domain of unknown function (DUF4764)	1	1	1	1
PF15963	Myb_DNA-bind_7	Myb DNA-binding like	0	1	1	0
PF15964	NA	Centrosomal colon cancer autoantigen protein family	0	4	3	0
PF15965	NA	TRAF-like zinc-finger	1	3	1	0
PF15966	NA	F-box	1	3	1	1
PF15967	Nucleoporin_FG2	Nucleoporin FG repeated region	1	3	1	1
PF15975	NA	Flotillin	2	2	5	0
PF15982	NA	N-terminal cysteine-rich region of Transmembrane protein 135	1	1	3	0
PF15985	KH_6	KH domain	1	2	3	1
PF15990	NA	UPF0767 family	1	1	1	0
PF15991	G_path_suppress	G-protein pathway suppressor	1	2	2	0
PF15993	NA	Fuseless	2	4	1	0
PF15996	NA	Arginine/serine-rich protein PNISR	1	3	4	1
PF15997	NA	Domain of unknown function (DUF4772)	1	1	1	1
PF15998	NA	Domain of unknown function (DUF4773)	1	4	5	1
PF16000	CARMIL_C	CARMIL C-terminus	2	1	16	0
PF16002	NA	Headcase protein	1	1	2	2
PF16004	EFTUD2	116 kDa U5 small nuclear ribonucleoprotein component N-terminus	1	1	2	0

PF16006	NA	Nucleolar and spindle-associated protein	1	1	1	0
PF16011	NA	Carbohydrate-binding family 9	1	0	0	0
PF16013	NA	Domain of unknown function (DUF4781)	0	4	0	0
PF16014	NA	Histone deacetylase complex subunit SAP130 C-terminus	2	1	2	0
PF16015	NA	Promethin	1	0	0	0
PF16016	VASt	Domain of unknown function (DUF4782)	1	6	2	0
PF16017	NA	BTB/POZ domain	1	1	2	1
PF16018	NA	Anillin N-terminus	0	1	4	0
PF16019	NA	Cysteine/serine-rich nuclear protein N-terminus	1	1	1	1
PF16020	NA	Deltamethrin resistance	0	2	0	0
PF16021	NA	Programmed cell death protein 7	1	2	1	0
PF16025	NA	Calcium-dependent calmodulin binding	1	5	0	0
PF16026	NA	Mitochondria-eating protein	2	4	140	4
PF16028	NA	Solute carrier family 3 member 2 N-terminus	1	1	4	2
PF16029	NA	Domain of unknown function (DUF4787)	0	1	1	1
PF16034	NA	JAKMIP CC3 domain	0	7	1	0
PF16038	NA	TMIE protein	1	2	0	0
PF16041	NA	Domain of unknown function (DUF4793)	1	3	0	2
PF16043	NA	Domain of unknown function (DUF4795)	1	1	8	0
PF16044	NA	Domain of unknown function (DUF4796)	0	1	2	0
PF16045	NA	LisH	1	2	2	0
PF16046	NA	FAM76 protein	1	1	2	0
PF16050	NA	Paf1 complex subunit CDC73 N-terminal	1	1	1	1
PF16053	MRP-S34	Mitochondrial 28S ribosomal protein S34	1	0	1	0
PF16054	NA	Transmembrane protein family 72	2	3	5	0
PF16057	NA	Domain of unknown function (DUF4800)	1	1	1	1
PF16058	NA	Mucin-like	1	2	5	0
PF16059	NA	Domain of unknown function (DUF4801)	1	0	4	0
PF16064	NA	Domain of unknown function (DUF4806)	0	0	10	0
PF16065	NA	Domain of unknown function (DUF4807)	1	1	0	0
PF16066	NA	Domain of unknown function (DUF4808)	1	2	2	1
PF16070	NA	Transmembrane protein family 132	2	1	1	1
PF16076	NA	Acyltransferase C-terminus	5	13	10	3
PF16077	Spaetzle	Spaetzle	0	0	0	1
PF16078	NA	2-oxoglutarate dehydrogenase N-terminus	3	2	5	0
PF16088	NA	UPF0693 family	0	0	2	0
PF16090	NA	Domain of unknown function (DUF4819)	1	3	1	1
PF16092	NA	Domain of unknown function (DUF4821)	1	1	1	1
PF16093	PAC4	Proteasome assembly chaperone 4	1	0	0	0
PF16094	NA	Proteasome assembly chaperone 4	1	1	1	0
PF16095	COR	C-terminal of Roc, COR, domain	5	18	41	2
PF16099	RMI1_C	Recq-mediated genome instability protein 1, C-terminal OB-fold	0	1	1	0
PF16100	RMI2	RecQ-mediated genome instability protein 2	1	1	0	0
PF16113	ECH_2	Enoyl-CoA hydratase/isomerase	1	1	4	1
PF16114	Citrate_bind	ATP citrate lyase citrate-binding	1	5	4	0
PF16116	NA	Domain of unknown function (DUF4832)	2	0	0	1
PF16121	40S_S4_C	40S ribosomal protein S4 C-terminus	1	1	1	0
PF16122	40S_SA_C	40S ribosomal protein SA C-terminus	1	1	1	0
PF16123	HAGH_C	Hydroxyacylglutathione hydrolase C-terminus	2	8	2	0
PF16124	RecQ_Zn_bind	RecQ zinc-binding	5	7	9	0
PF16134	NA	THO complex subunit 2 N-terminus	1	1	1	0
PF16158	NA	Ig-like domain from next to BRCA1 gene	2	1	4	0
PF16159	NA	FOXP coiled-coil domain	1	1	6	0
PF16165	NA	Ferlin C-terminus	2	2	20	1
PF16172	DOCK_N	DOCK N-terminus	3	9	6	0
PF16173	NA	Domain of unknown function (DUF4874)	2	0	0	0
PF16174	NA	Intracellular hyaluronan-binding protein 4 N-terminal	0	1	2	1
PF16177	ACAS_N	Acetyl-coenzyme A synthetase N-terminus	4	10	6	1
PF16178	Anoct_dimer	Dimerisation domain of Ca+-activated chloride-channel, anoctamin	4	21	30	0
PF16179	RHD_dimer	Rel homology dimerisation domain	2	3	8	0
PF16183	Kinesin_assoc	Kinesin-associated	2	13	14	1
PF16184	NA	Cadherin-like	4	6	7	19
PF16185	NA	Mitochondrial ABC-transporter N-terminal five TM region	1	3	2	2
PF16186	Arm_3	Atypical Arm repeat	3	3	4	1
PF16187	Peptidase_M16_M	Middle or third domain of peptidase_M16	2	5	5	1
PF16188	NA	C-terminal region of peptidase_M24	2	11	7	0
PF16189	NA	Creatinase/Prolidase N-terminal domain	2	11	5	0
PF16190	E1_FCCH	Ubiquitin-activating enzyme E1 FCCH domain	2	3	4	0
PF16191	E1_4HB	Ubiquitin-activating enzyme E1 four-helix bundle	2	3	3	0
PF16192	NA	C-terminal four TMM region of protein-O-mannosyltransferase	2	4	2	1
PF16193	AAA_assoc_2	AAA C-terminal domain	1	0	0	0
PF16197	KAsynt_C_assoc	Ketoacyl-synthetase C-terminal extension	3	5	3	2
PF16198	TruB_C_2	tRNA pseudouridylate synthase B C-terminal domain	1	1	1	0
PF16199	Radical_SAM_C	Radical_SAM C-terminal domain	1	3	1	0
PF16200	NA	C-terminal region of band_7	1	1	1	0
PF16201	NA	Nucleolar pre-ribosomal-associated protein 1	2	1	1	0
PF16203	ERCC3_RAD25_C	ERCC3/RAD25/XPB C-terminal helicase	1	1	1	0

PF16205	Ribosomal_S17_N	Ribosomal_S17 N-terminal	1	2	1	0
PF16206	NA	C-terminal region of Mon2 protein	2	2	3	4
PF16207	RAWUL	RAWUL domain RING finger- and WD40-associated ubiquitin-like	7	7	11	0
PF16209	PhoLip_ATPase_N	Phospholipid-translocating ATPase N-terminal	6	21	37	1
PF16211	Histone_H2A_C	C-terminus of histone H2A	30	4	14	1
PF16212	PhoLip_ATPase_C	Phospholipid-translocating P-type ATPase C-terminal	5	22	35	3
PF16213	NA	Dimerisation and cyclophilin-binding domain of Mon2	2	4	11	1
PF16214	NA	Adenylyl cyclase N-terminal extracellular and transmembrane region	4	5	6	3
PF16218	Peptidase_C101	Peptidase family C101	1	3	9	2
PF16273	NA	Nuclear distribution C domain	0	0	0	1
PF16275	SF1-HH	Splicing factor 1 helix-hairpin domain	1	1	2	0
PF16278	zf-C2HE	C2HE / C2H2 / C2HC zinc-binding finger	1	1	1	0
PF16282	SANT_DAMP1_like	SANT/Myb-like domain of DAMP1	1	1	1	0
PF16294	RSB_motif	RNSP1-SAP18 binding (RSB) motif	1	1	1	0
PF16300	NA	Type of WD40 repeat	3	5	16	1
PF16317	Glyco_hydro_99	Glycosyl hydrolase family 99	0	8	2	2
PF16320	Ribosomal_L12_N	Ribosomal protein L7/L12 dimerisation domain	1	3	1	0
PF16322	NA	Tubby N-terminal	1	0	0	0
PF16347	DUF4976	Domain of unknown function (DUF4976)	0	0	4	1
PF16350	FAO_M	FAD dependent oxidoreductase central domain	3	1	1	0
PF16360	GTP-bdg_M	GTP-binding GTPase Middle Region	1	1	3	0
PF16363	GDP_Man_Dehyd	GDP-mannose 4,6 dehydratase	3	9	5	5
PF16366	CEBP_ZZ	Cytoplasmic polyadenylation element-binding protein ZZ domain	2	2	2	1
PF16367	NA	RNA recognition motif	2	2	2	0
PF16368	NA	Cytoplasmic polyadenylation element-binding protein 1 N-terminus	2	1	1	0
PF16381	Coatomer_g_Cpla	Coatomer subunit gamma-1 C-terminal appendage platform	1	1	1	0
PF16399	Aquarius_N	Intron-binding protein aquarius N-terminus	1	1	2	1
PF16401	NA	Domain of unknown function (DUF5009)	3	2	2	0
PF16413	Mlh1_C	DNA mismatch repair protein Mlh1 C-terminus	1	1	1	1
PF16414	NPC1_N	Niemann-Pick C1 N terminus	0	3	2	2
PF16415	CNOT1_CAF1_bind	CCR4-NOT transcription complex subunit 1 CAF1-binding domain	1	1	3	0
PF16417	NA	CCR4-NOT transcription complex subunit 1 TTP binding domain	1	1	3	1
PF16418	NA	CCR4-NOT transcription complex subunit 1 HEAT repeat	1	1	3	0
PF16420	ATG7_N	Ubiquitin-like modifier-activating enzyme ATG7 N-terminus	1	1	2	0
PF16421	E2F_CC-MB	E2F transcription factor CC-MB domain	1	3	3	0
PF16422	COE1_DBD	Transcription factor COE1 DNA-binding domain	1	2	2	0
PF16423	COE1_HLH	Transcription factor COE1 helix-loop-helix domain	1	2	2	0
PF16453	IQ_SEC7_PH	PH domain	1	2	3	0
PF16454	PI3K_P85_iSH2	Phosphatidylinositol 3-kinase regulatory subunit P85 inter-SH2 domain	1	4	6	2
PF16455	NA	Ubiquitin-binding domain	1	1	2	1
PF16457	PH_12	Pleckstrin homology domain	2	4	7	0
PF16470	NA	Peptidase S8 pro-domain	4	8	16	2
PF16471	NA	JNK-interacting protein leucine zipper II	1	1	13	1
PF16472	NA	Domain of unknown function (DUF5050)	1	0	1	0
PF16474	KIND	Kinase non-catalytic C-lobe domain	1	2	5	1
PF16477	NA	Domain of unknown function (DUF5054)	1	4	2	0
PF16482	Staufen_C	Staufen C-terminal domain	1	10	3	0
PF16484	CPT_N	Carnitine O-palmitoyltransferase N-terminus	1	10	3	0
PF16486	ArgoN	N-terminal domain of argonaute	2	8	11	1
PF16487	ArgoMid	Mid domain of argonaute	1	5	7	0
PF16488	ArgoL2	Argonaute linker 2 domain	1	5	7	0
PF16489	NA	GPCR-Autoproteolysis INducing (GAIN) domain	2	11	30	1
PF16491	Peptidase_M48_N	CAAX prenyl protease N-terminal, five membrane helices	1	1	1	0
PF16493	NA	N-terminal of Homeobox Meis and PKNOX1	2	10	6	1
PF16494	NA	C-terminal extension of sodium/calcium exchanger domain	1	4	5	1
PF16495	SWIRM-assoc_1	SWIRM-associated region 1	1	3	6	1
PF16496	NA	SWIRM-associated domain at the N-terminal	1	4	6	1
PF16498	SWIRM-assoc_3	SWIRM-associated domain at the C-terminal	1	3	6	1
PF16499	Melibiase_2	Alpha galactosidase A	4	7	5	2
PF16500	Cyclin_N2	N-terminal region of cyclin_N	1	0	1	0
PF16501	NA	S phase cyclin A-associated protein in the endoplasmic reticulum	1	2	2	0
PF16503	NA	Zinc-ribbon	1	1	1	0
PF16507	BLM10_mid	Proteasome-substrate-size regulator, mid region	1	2	2	0
PF16508	NA	Second BRCT domain on Nijmegen syndrome breakage protein	1	1	2	1
PF16511	NA	N-terminal or F0 domain of Talin-head FERM	2	5	14	0
PF16512	NA	p190-A and -B Rho GAPs FF domain	1	5	5	0
PF16516	CC2-LZ	Leucine zipper of domain CC2 of NEMO, NF-kappa-B essential modulator	1	1	3	0
PF16517	NA	Novel Ras effector 1 C-terminal SARAH (Sav/Rassf/Hpo) domain	1	6	7	0
PF16519	TRPM_tetra	Tetramerisation domain of TRPM	1	4	12	0
PF16521	NA	Myosin VI cargo binding domain	1	3	5	2
PF16523	NA	betaPIX coiled coil	1	0	8	0
PF16526	CLZ	C-terminal leucine zipper domain of cyclic nucleotide-gated channels	2	1	5	0
PF16528	Exo84_C	Exocyst component 84 C-terminal	1	1	1	0
PF16529	NA	WD40 region of Ge1, enhancer of mRNA-decapping protein	1	5	4	0
PF16531	SAS-6_N	Centriolar protein SAS N-terminal	0	2	3	0
PF16533	SOAR	STIM1 Orai1-activating region	1	6	4	1
PF16534	ULD	Ubiquitin-like oligomerisation domain of SATB	1	3	1	0

PF16540	NA	Arf6-interacting domain of mitotic kinesin?릳ike protein 1	0	1	2	0
PF16543	NA	DRG Family Regulatory Proteins, Tma46	1	2	1	0
PF16544	STAR_dimer	Homodimerisation region of STAR domain protein	1	5	5	0
PF16545	CCM2_C	Cerebral cavernous malformation protein, harmonin-homology	1	1	3	0
PF16546	SGTA_dimer	Homodimerisation domain of SGTA	0	2	2	0
PF16550	RPN13_C	UCH-binding domain	1	1	1	1
PF16558	AZUL	Amino-terminal Zinc-binding domain of ubiquitin ligase E3A	1	1	0	1
PF16559	NA	GIT coiled-coil Rho guanine nucleotide exchange factor	1	0	4	0
PF16561	AMPK1_CBM	Glycogen recognition site of AMP-activated protein kinase	2	11	16	0
PF16562	NA	N-terminal domain of E3 ubiquitin-protein ligase HECW1 and 2	1	1	1	0
PF16563	NA	Coiled-coil and interaction region of P66A and P66B with MBD2	1	2	5	1
PF16564	NA	p55-binding region of Methyl-CpG-binding domain proteins MBD	1	1	3	0
PF16565	NA	Phospholipase D-like domain at C-terminus of MIT	1	9	6	0
PF16566	NA	Cell-cycle alteration and expression-elevated protein in tumour	2	2	3	1
PF16573	CLP1_N	N-terminal beta-sandwich domain of polyadenylation factor	1	2	1	0
PF16574	NA	Coiled-coil region of centrosome protein CE290	0	1	2	0
PF16575	CLP1_P	mRNA cleavage and polyadenylation factor CLP1 P-loop	2	3	4	0
PF16577	UBA_5	UBA domain	1	2	3	0
PF16579	AdenylateSensor	Adenylate sensor of SNF1-like protein kinase	1	2	13	0
PF16589	BRCT_2	BRCT domain, a BRCA1 C-terminus domain	2	10	12	0
PF16608	NA	TNRC6-PABC binding domain	0	0	0	2
PF16609	NA	SH3-RhoGEF linking unstructured region	1	0	0	0
PF16614	NA	Unstructured region two on RhoGEF 6 and 7	1	1	8	0
PF16622	zf-C2H2_11	zinc-finger C2H2-type	1	0	3	3
PF16641	NA	CLIP1 zinc knuckle	1	1	19	0
PF16646	AXIN1_TNKS_BD	Axin-1 tankyrase binding domain	0	4	3	1
PF16652	PH_13	Pleckstrin homology domain	3	4	37	0
PF16653	Sacchrp_dh_C	Saccharopine dehydrogenase C-terminal domain	1	1	1	2
PF16656	Pur_ac_phosph_N	Purple acid Phosphatase, N-terminal domain	4	12	5	1
PF16661	Lactamase_B_6	Metallo-beta-lactamase superfamily domain	4	3	3	0
PF16669	TTC5_OB	Tetratricopeptide repeat protein 5 OB fold domain	1	1	1	0
PF16672	LAMTOR5	Ragulator complex protein LAMTOR5	1	1	1	0
PF16673	NA	TNF receptor-associated factor BIRC3 binding domain	0	0	2	0
PF16676	NA	Transactivation domain of FOXO protein family	0	1	0	1
PF16678	NA	UB-associated-like domain of HOIP or E3 ubiquitin-protein ligase	1	1	1	1
PF16679	NA	DNA replication factor Cdt1 C-terminal domain	0	1	2	0
PF16680	Ig_4	T-cell surface glycoprotein CD3 delta chain	0	1	0	0
PF16682	MSL2-CXC	CXC domain of E3 ubiquitin-protein ligase MSL2	0	1	1	1
PF16685	zf-RING_10	zinc RING finger of MSL2	1	1	1	1
PF16686	POT1PC	ssDNA-binding domain of telomere protection protein	0	1	1	0
PF16687	NA	beta-propeller of ELYS nucleoporin	0	1	1	1
PF16689	NA	Coiled-coil N-terminus of APC, dimerisation domain	1	1	1	1
PF16690	MMACHC	Methylmalonic aciduria and homocystinuria type C family	0	1	1	1
PF16692	NA	Folliculin C-terminal domain	1	4	1	3
PF16696	NA	Zinc finger FYVE domain-containing protein 21 C-terminus	1	1	2	0
PF16698	ADAM17_MPD	Membrane-proximal domain, switch, for ADAM17	2	14	3	1
PF16699	NA	Cleavage stimulation factor subunit 1, dimerisation domain	1	1	1	0
PF16704	NA	Rab binding domain	1	2	2	0
PF16705	NA	NUDIX, or N-terminal NPxY motif-rich, region of KRIT	1	2	2	1
PF16717	NA	Ribosome-associated complex head domain	1	1	1	0
PF16725	NA	Nucleolin binding domain	1	1	1	0
PF16727	REV1_C	DNA repair protein REV1 C-terminal domain	1	0	1	0
PF16737	NA	PHD finger protein 12 MRG binding domain	1	2	2	0
PF16739	CARD_2	Caspase recruitment domain	3	12	11	1
PF16740	NA	Spindle and kinetochore-associated protein 2	1	2	2	0
PF16741	NA	mRNA-decapping enzyme C-terminus	0	0	1	0
PF16744	Zf_RING	KIAA1045 RING finger	2	2	4	1
PF16746	BAR_3	BAR domain of APPL family	4	20	17	1
PF16748	INSC_LBD	Inscuteable LGN-binding domain	0	0	1	0
PF16752	NA	Tubulin-specific chaperone C N-terminal domain	1	1	1	1
PF16757	Fucosidase_C	Alpha-L-fucosidase C-terminal domain	10	7	11	6
PF16759	LIG3_BRCT	DNA ligase 3 BRCT domain	3	1	7	0
PF16769	NA	MCM3AP domain of GANP	0	4	3	1
PF16770	RTT107_BRCT_5	Regulator of Ty1 transposition protein 107 BRCT domain	1	0	0	0
PF16776	NA	Type II inositol 1,4,5-trisphosphate 5-phosphatase PH domain	0	3	3	0
PF16783	FANCM-MHF_bd	FANCM to MHF binding domain	0	1	1	0
PF16794	NA	Fibronectin-III type domain	1	2	1	0
PF16808	PKcGMP_CC	Coiled-coil N-terminus of cGMP-dependent protein kinase	1	0	1	1
PF16835	SF3A2	Pre-mRNA-splicing factor SF3a complex subunit 2 (Prp11)	1	1	1	1
PF16837	SF3A3	Pre-mRNA-splicing factor SF3A3, of SF3a complex, Prp9	1	1	1	0
PF16845	SQAPI	Aspartic acid proteinase inhibitor	0	0	1	0
PF16858	NA	Condensin II complex subunit CAP-H2 or CNDH2, C-term	1	1	0	0
PF16860	NA	CHCH-CHCH-like Cx9C, IMS import disulfide relay-system,	1	1	0	0
PF16863	NtCtMGAM_N	N-terminal barrel of NtMGAM and CtMGAM, maltase-glucoamylase	7	4	4	0
PF16864	NA	Dimerisation domain	1	0	1	0
PF16865	GST_C_5	Glutathione S-transferase, C-terminal domain	0	0	1	0
PF16866	PHD_4	PHD-finger	1	1	1	1

PF16869	NA	PF16858	1	0	0	0
PF16870	OxoGdeHyase_C	2-oxoglutarate dehydrogenase C-terminal	3	3	6	1
PF16871	NA	Domain of unknown function (DUF5077)	0	0	1	0
PF16876	NA	Lipin/Ned1/Smp2 multi-domain protein middle domain	1	1	3	1
PF16878	SIX1_SD	Transcriptional regulator, SIX1, N-terminal SD domain	3	8	6	3
PF16879	NA	C-terminal domain of Sin3a protein	1	2	6	1
PF16880	NA	N-terminal EH-domain containing protein	1	1	1	1
PF16881	NA	N-terminal domain of lipoyl synthase of Radical_SAM family	2	1	2	0
PF16884	ADH_N_2	N-terminal domain of oxidoreductase	3	10	6	0
PF16885	NA	Voltage-gated calcium channel subunit alpha, C-term	1	0	13	0
PF16886	ATP-synt_ab_Xtn	ATPsynthase alpha/beta subunit N-term extension	1	1	3	0
PF16891	STPPase_N	Serine-threonine protein phosphatase N-terminal domain	3	9	7	0
PF16897	NA	C-terminal region of MMR_HSR1 domain	2	2	2	0
PF16898	TOPRIM_C	C-terminal associated domain of TOPRIM	1	4	1	1
PF16899	Cyclin_C_2	Cyclin C-terminal domain	2	5	4	0
PF16900	REPA_OB_2	Replication protein A OB domain	2	2	4	0
PF16901	DAO_C	C-terminal domain of alpha-glycerophosphate oxidase	1	1	2	0
PF16905	GPHH	Voltage-dependent L-type calcium channel, IQ-associated	2	3	44	2
PF16906	Ribosomal_L26	Ribosomal proteins L26 eukaryotic, L24P archaeal	1	3	1	0
PF16908	NA	Vacuolar sorting-associated protein 13, N-terminal	3	10	11	0
PF16909	NA	Vacuolar-sorting-associated 13 protein C-terminal	4	10	16	2
PF16910	NA	Repeating coiled region of VPS13	3	10	11	1
PF16916	ZT_dimer	Dimerisation domain of Zinc Transporter	2	2	1	6
PF16921	NA	Tex protein YqgF-like domain	1	1	1	0
PF16922	SLD5_C	DNA replication complex GINS protein SLD5 C-terminus	1	0	1	0
PF16923	NA	Glycosyl hydrolase family 63 N-terminal domain	1	4	1	1
PF16953	PRORP	Protein-only RNase P	1	1	1	0
PF16954	NA	Haem-transporter, endosomal/lysosomal, haem-responsive gene	1	1	1	0
PF16969	SRP68	RNA-binding signal recognition particle 68	1	1	1	1
PF16977	NA	C-terminal domain of apextrin	5	20	23	3
PF16978	CRIM	SAPK-interacting protein 1 (Sin1), middle CRIM domain	1	2	1	0
PF16979	SIN1_PH	SAPK-interacting protein 1 (Sin1), Pleckstrin-homology	1	2	1	1
PF17004	SRP_TPR_like	Putative TPR-like repeat	1	1	1	0
PF17034	NA	Zinc-ribbon like family	1	2	3	0
PF17035	BET	Bromodomain extra-terminal - transcription regulation	1	1	7	1
PF17039	NA	Fucosyltransferase, N-terminal	18	37	25	14
PF17045	NA	Centrosomal protein of 63 kDa	0	0	12	0
PF17047	NA	Synaptotagmin-like mitochondrial-lipid-binding domain	2	6	4	0
PF17048	Ceramidse_alk_C	Neutral/alkaline non-lysosomal ceramidase, C-terminal	1	2	1	0
PF17064	NA	Sleepless protein	1	0	2	0
PF17065	NA	Putative cytokine, C6ORF120	1	2	1	1
PF17066	NA	RBPJ-interacting and tubulin associated protein	0	2	1	0
PF17069	NA	Arginine/Serine-Rich protein 1	0	0	3	0
PF17092	PCB_OB	Penicillin-binding protein OB-like domain	0	1	0	0
PF17095	NA	Spectrin-binding region of Ca2+-Calmodulin	0	1	3	1
PF17098	NA	Pre-mRNA-splicing regulator WTAP	1	1	1	1
PF17101	NA	Stealth protein CR1, conserved region 1	1	1	3	0
PF17102	NA	Stealth protein CR3, conserved region 3	1	1	3	0
PF17103	NA	Stealth protein CR4, conserved region 4	1	1	3	0
PF17105	NA	C-terminal domain of bromodomain protein 4	1	1	7	0
PF17120	NA	Zinc-ribbon, C4HC2 type	1	1	3	0
PF17121	zf-C3HC4_5	Zinc finger, C3HC4 type (RING finger)	1	1	1	0
PF17123	NA	RING-like zinc finger	0	1	1	1
PF17125	Methyltr_RsmF_N	N-terminal domain of 16S rRNA methyltransferase RsmF	1	0	6	0
PF17172	GST_N_4	Glutathione S-transferase N-terminal domain	0	0	0	2
PF17207	MCM_OB	MCM OB domain	0	0	0	1
PF17213	NA	Hydin Adenylate kinase-like domain	0	0	0	1
PF17216	Rrp44_CSD1	Rrp44-like cold shock domain	0	0	0	2
PF17217	NA	UPA domain	0	0	0	3
PF17218	NA	CBX family C-terminal motif	0	0	0	3
PF17285	PRMT5_TIM	PRMT5 TIM barrel domain	0	0	0	1
PF17292	POB3_N	POB3-like N-terminal PH domain	0	0	0	1
PF17297	PEPCK_N	Phosphoenolpyruvate carboxykinase N-terminal domain	0	0	0	6
PF17450	Melibiase_2_C	Alpha galactosidase A C-terminal beta sandwich domain	0	0	0	1
PF17517	NA	IgGFc binding protein	0	0	0	25
